# Oxidative Rearrangement of Indoles Enabled by Promiscuous Cryptic Halogenation with Vanadium-Dependent Haloperoxidases

**DOI:** 10.1021/acscatal.5c07839

**Published:** 2026-01-14

**Authors:** Hyung Ji Lee, Carter U. Brzezinski, Sergio A. Solis, Raina S. Semenick, Ana Villalobos Galindo, Sophia G. Barthel, John Bacsa, Kyle F. Biegasiewicz

**Affiliations:** † Department of Chemistry, 1371 Emory University, Atlanta, Georgia 30322, United States; ‡ School of Molecular Sciences, Arizona State University, Tempe, Arizona 85281, United States

**Keywords:** biocatalysis, oxindole, spirooxindole, vanadium, haloperoxidase

## Abstract

The 2-oxindole class of heterocycles are privileged structural components in natural products and biologically active compounds. One of the most attractive methods for accessing 2-oxindoles is through direct oxidation of indoles, but current methods rely on the use of chemical oxidizing agents that lead to the generation of harmful waste products or biocatalytic methods using enzymes with a limited substrate scope. Herein, we describe the development and application of a general biocatalytic platform for the oxidative rearrangement of indoles using enzymatic halide recycling with vanadium-dependent haloperoxidases (VHPOs) facilitated by a catalytic quantity of halide salt and hydrogen peroxide as the terminal oxidant. This catalytic system is effective for the oxidative rearrangement of indoles into 2-oxindoles and 2-spirooxindoles. The developed protocol has been applied in multienzymatic and chemoenzymatic synthesis, late-stage functionalization of biologically active molecules, tryptophan-selective peptide modification, and gram-scale syntheses of coerulescine and horsfiline.

## Graphical abstract

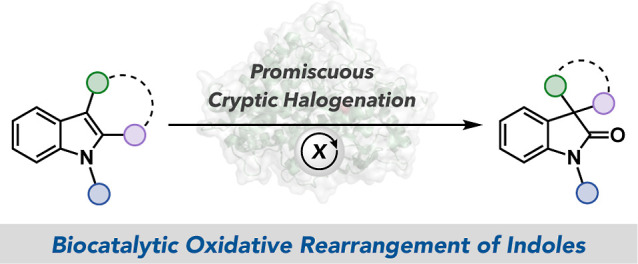

## Introduction

The 2-oxindole class of heterocycles are privileged motifs in pharmaceuticals and natural products with a diverse range of biological activities (Figure 1a).^1–9^ One of the most extensively studied methods for accessing 2-oxindoles is through the direct oxidation of indoles to generate oxindoles or spirooxindoles. Despite the logistical efficiency of this strategy, traditional methods for indole oxidation involve the use of chemical oxidants that are hazardous, lead to a host of undesired oxidized side products, or generate a stoichiometric quantity of downstream waste, all of which present significant challenges for conducting this reaction type at scale.^10–32^ In recent years, greener methods for indole oxidation have been developed using electrochemistry,^33–35^ catalytic halide with oxone as the terminal oxidant,^36^ or catalytic metal salts with hydrogen peroxide (H_2_O_2_) as the terminal oxidant.^37^ However, these protocols require significant quantities of organic solvents, limiting their potential applications in chemoenzymatic synthesis. Enzymes are an attractive alternative to current methods for indole oxidation because of their selectivity and sustainability parameters.^38,39^ While many enzymes have been explored for performing direct enzymatic oxidation of indoles including cytochrome P450 enzymes (P450),^40–42^ horseradish peroxidase (HRP),^43^ indoleamine 2,3-dioxygenase (IDO),^44^ and heme-dependent haloperoxidase enzymes (HP),^45–49^ these biocatalysts are limited to narrow substrate scopes and strictly catalyze direct indole oxidation. Similarly, while a small selection of enzymes has been reported to initiate the oxidative rearrangement of 2,3-disubstituted indole-containing scaffolds to generate spirooxindoles in the context of natural product biosynthesis,^50–54^ these examples are restricted to enzymes with limited substrate promiscuity for this single reaction type, leaving a general biocatalytic platform for oxidative rearrangement of indoles elusive and highly desirable (Figure 1b).

**Figure 1:**
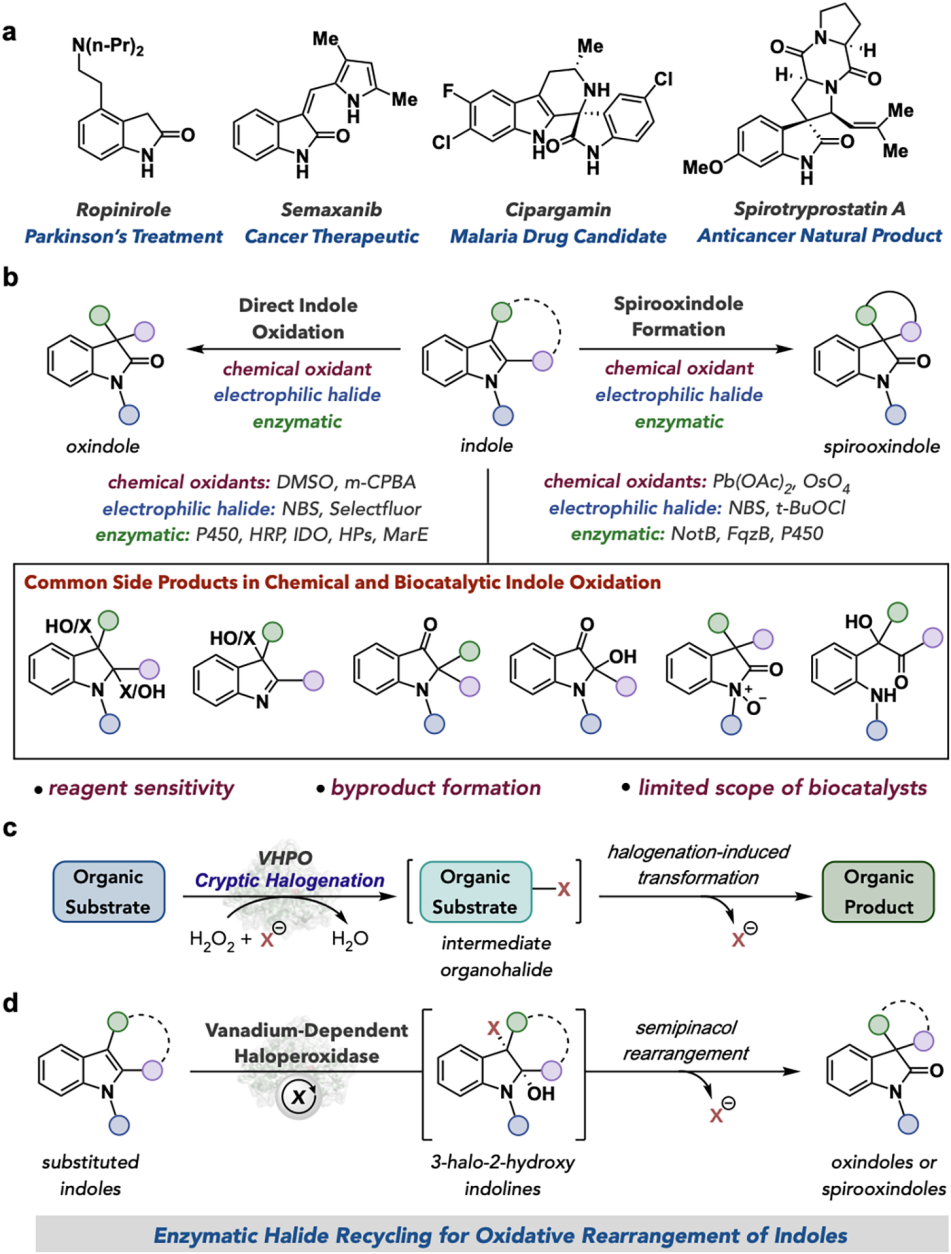
Oxidative rearrangement of indoles overview. (a) Biologically active indole and spirooxindoles.^1–9^ (b) Chemical and enzymatic strategies for oxidative rearrangement of indoles.^10–54,83^ (c) Cryptic halide recycling by VHPOs.^65,83,84^ (d) Proposed general biocatalytic strategy for oxidative rearrangement of indoles using enzymatic halide recycling.

We recently hypothesized that the vanadium-dependent haloperoxidase (VHPO) class of enzymes could address the above challenges on the basis of their recent emergence as a biocatalyst platform in synthetic organic chemistry.^55–60^ In nature, VHPOs are responsible for the selective halogenation of organic substrates through the generation of hypohalous acid (HOX) using simple halide salts and relying on hydrogen peroxide (H_2_O_2_) as the terminal oxidant.^61–64^ In addition to their more traditional reaction modes of performing direct halogenations, VHPOs are known for their ability to perform cryptic halogenation reactions. This reactivity regime refers to an enzyme-catalyzed halogenation of an organic substrate that activates a substrate for an ensuing halogenation-induced bond formation and loss of the halide (Figure 1c).^65^ Halogenating enzymes have used this strategy to perform a host of transformations including cyclopropanation,^66–72^ alkyne formation,^73–75^ alkylation,^76–80^ and biaryl coupling reactions.^81,82^ For reaction development, we were particularly intrigued by two important reports. First, studies by Butler and co-workers demonstrated that vanadium-dependent bromoperoxidases (VBPOs) could perform regioselective indole oxidation on 1,3-di-*tert*-butylindole to give the corresponding oxindole.^83^ In addition, studies by Moore and co-workers have revealed that vanadium-dependent chloroperoxidases (VCPOs) enable chlorination-induced α-hydroxyketone rearrangement in marinone natural product biosynthesis.^84^ We have recently capitalized on the catalytic capabilities of VHPOs to perform halogenation-mediated transformations using enzymatic halide recycling (EHR).^85–87^ Mechanistically, VHPO is responsible for the repetitive oxidation of a catalytic quantity of halide to generate HOX as the halogenating agent, activating the substrate for an ensuing chemical transformation with loss of halide. In the context of the oxidative rearrangement of indoles, we hypothesized that VHPO would be responsible for initiating the formation of the key 3-halo-2-hydroxyindoline through halogenation and subsequent trapping by water. This intermediate would proceed through semipinacol rearrangement to give the corresponding oxindole or spirooxindole, while the halide was released to be recycled by the enzyme. Herein, we report that VHPOs are a general biocatalyst platform for performing oxidative rearrangement of indoles to give oxindoles and spirooxindoles (Figure 1d).

## Methods and Results

Our studies commenced by interrogating our in-house library of VHPOs in both direct oxidation and spirooxindole formation reactions (Figure 2). We began by screening structurally diverse VHPOs, including chloroperoxidase from *Curvularia inaequalis* (*Ci*VCPO),^88^ and bromoperoxidases from *Corallina officinalis* (*Co*VBPO),^89^ *Corallina pilulifera* (*Cp*VBPO),^90^ and *Acaryochloris marina* (*Am*VBPO),^91^ for the conversion of 3-methyl-1*H*-indole (**1**) to 3-methylindolin-2-one (**2**). Subjection of **1** to each biocatalyst (0.0063 mol %), sodium orthovanadate (Na_3_VO_4_, 0.1 mM), potassium bromide (KBr, 1.0 equiv), and H_2_O_2_ (1.0 equiv) in citrate buffer (100 mM, pH = 5) and acetonitrile (MeCN, 30% v/v) for 2 h provided **2** in yields ranging from 7 to 95% (Figure 2, entries 1–4), with the most superior performance from *Ci*VCPO (Figure 2, entry 1). Gratifyingly, by simply increasing the reaction time to 4 h and increasing H_2_O_2_ loading to 2.0 equiv, the KBr loading could be decreased to 0.2 equiv to provide **2** in 96% yield (Figure 2, entry 5). To enhance the practical feasibility of the protocol, the reaction is run with *Escherichia coli* cells harboring *Ci*VCPO with an optical density (OD) of 18.5 to give **2** in 99% yield (Figure 2, entry 6). A series of control experiments excluding the addition of Na_3_VO_4_, H_2_O_2_, and KBr in turn confirmed the necessity of all reaction components (Figure 2, entries 7–9). Finally, a reaction was performed with an empty plasmid, confirming the necessity of *Ci*VCPO in the reaction (Figure 2, entry 10). Some other notable features of the reaction include tolerance of H_2_O_2_ loadings up to 4.0 equiv (Figure S8), optimal KBr loadings in the range of 0.1–0.3 equiv (Figure S9), and ideal reaction buffering with citrate buffer at pH = 5 (Figure S10) in the range of 5–400 mM (Figure S11). The reaction also performs well in a range of polar protic (MeOH, EtOH), polar aprotic (MeCN, DMSO, DMF, EtOAc, THF, 2-MeTHF), and nonpolar solvents (toluene, hexane) (Figure S12), with the most general compatibility with MeCN up to as much as 50% (v/v) (Figure S13). Importantly, when this reaction was conducted using chemical generation of hypobromous acid,^86^ no reactivity was observed.

**Figure 2:**
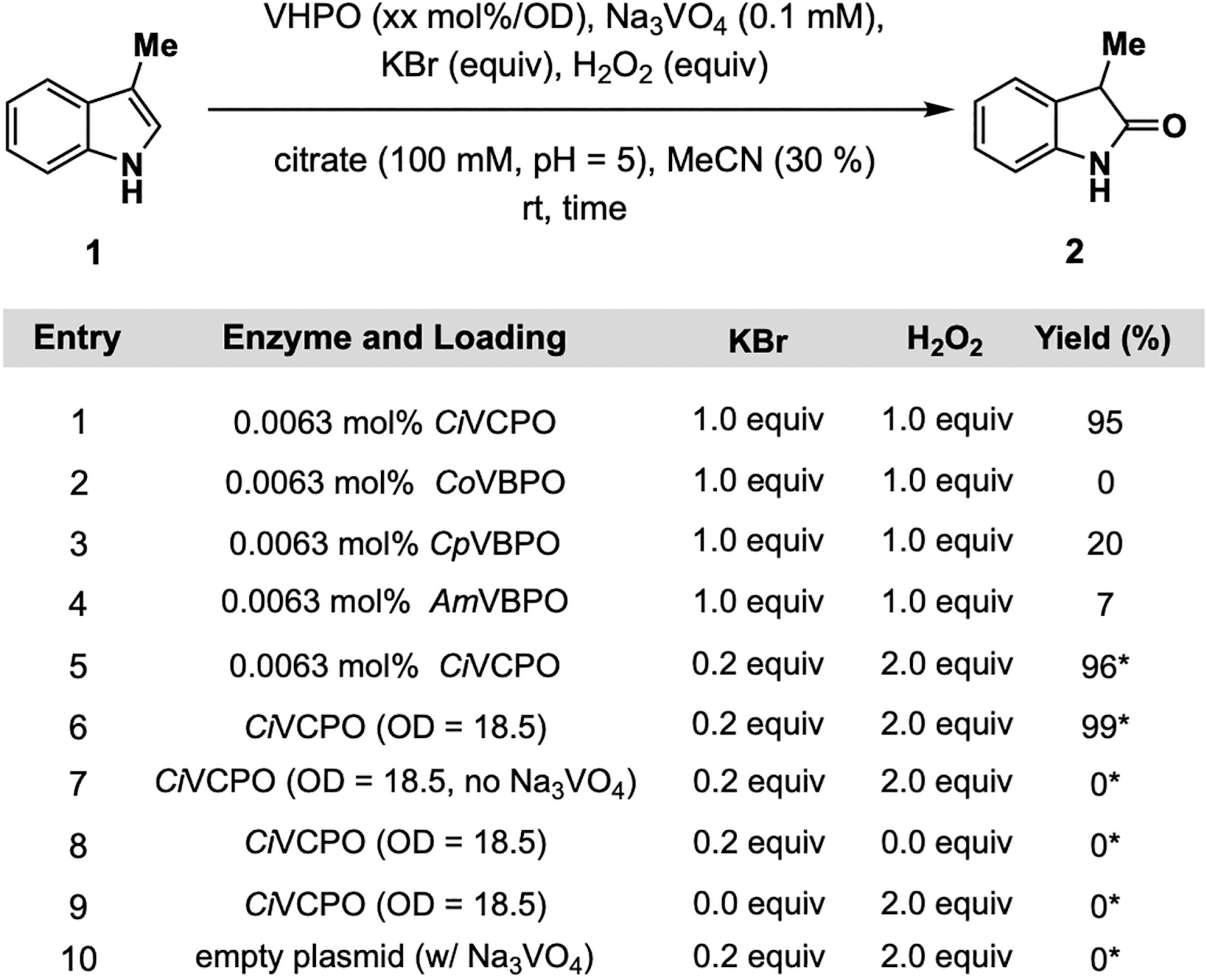
Optimization experiments for direct indole oxidation. Reaction conditions: **1** (4.0 μmol, 0.5 mg), VHPO (0.0022–0.0063 mol %), Na_3_VO_4_ (0.1 mM final concentration), KBr (0.2–1.0 equiv), H_2_O_2_ (1.0–2.0 equiv), citrate buffer (100 mM, pH = 5, 200 μL), MeCN (300 μL), 2 h, rt. 1 mL total reaction volume. *Reaction run for 4 h. Yields determined by HPLC based on a calibration curve. See the Supporting Information for details.

With optimized conditions for direct oxidation of indoles in hand, we next turned to spirooxindole formation in the conversion of 1-(1,3,4,9-tetrahydro-2*H*-pyrido­[3,4-*b*]­indol-2-yl)­ethan-1-one (**3**) to 1′-acetylspiro­[indoline-3,3′-pyrrolidin]-2-one (**4**). When the same set of biocatalysts (0.0016 mol %) was screened using similar conditions that featured Na_3_VO_4_ (1.0 mM), KBr (1.0 equiv), and H_2_O_2_ (1.0 equiv) in citrate buffer (50 mM, pH = 5) and *N*,*N*-dimethylformamide (DMF, 10% v/v) for 2 h (Figure 3, entries 1–4), three VHPOs (*Ci*VCPO, *Co*VBPO, and *Cp*VBPO) produced **4** in >99% yield (Figure 3, entries 1–3). Out of these well-performing biocatalysts, *Cp*VBPO was chosen as the featured enzyme for the transformation because of its comparatively higher expression levels and substrate promiscuity in the study. The reaction was readily conducted under EHR conditions with only 0.1 equiv of KBr by simply increasing the H_2_O_2_ loading to 2.0 equiv (Figure 3, entry 5). A series of control experiments excluding the addition of enzyme, Na_3_VO_4_, KBr, and H_2_O_2_ in turn confirmed the necessity of all reaction components (Figure 3, entries 6–9). Some other notable features of the reaction include tolerance of H_2_O_2_ loadings as high as 4.0 equiv (Figure S14), an optimal KBr loading of 0.1 equiv (Figure S15), and ideal reaction performance in both sodium acetate (NaOAc) and citrate buffers at pH = 5 (Figure S16) and in citrate buffer loadings from 50 to 500 mM (Figure S17). The reaction primarily performs well in DMF and dimethyl sulfoxide (DMSO) (Figure S18) and an optimal DMF loading of 10% (v/v) (Figure S19) because of substrate solubility. Similar to indole oxidation, no reactivity was observed using chemical generation of hypobromous acid.^86^

**Figure 3:**
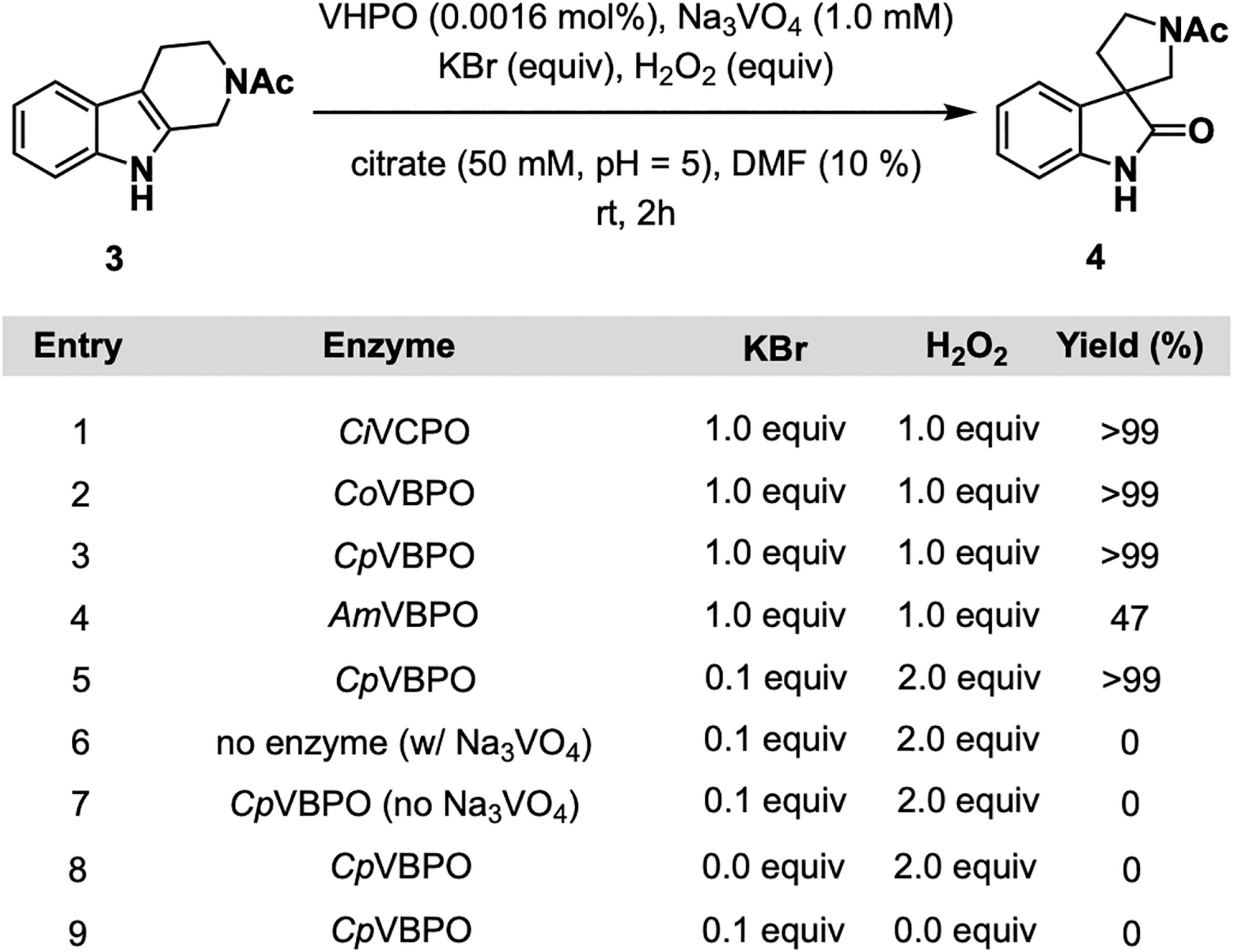
Optimization experiments for spirooxindole formation. Reaction conditions: **3** (4.0 μmol, 0.7 mg), VHPO (0.0016 mol %), Na_3_VO_4_ (1.0 mM final concentration), KBr (0.1–1.0 equiv), H_2_O_2_ (1.0–2.0 equiv), citrate buffer (50 mM, pH = 5, 100 μL), DMF (100 μL), 2 h, rt. 1 mL total reaction volume. Yields determined by HPLC based on a calibration curve. See the Supporting Information for details.

With our optimized conditions in hand for VHPO-catalyzed oxidative rearrangement of indoles, a series of 3-substituted and 2,3-substituted indoles were investigated for their performance in the catalyst system. For direct indole oxidation, the developed conditions for the conversion of indole **1** to oxindole **2** were tested at the preparative scale (0.4 mmol) in 97% yield and with a total turnover number (TTN) of 44,091 (Figure 4, **2**) without the need for further optimization. Notably, this reaction was demonstrated on a gram scale to generate **2** in 90% yield and 40,909 TTN. The catalyst system was applied to other 3-methyl-substituted N–H indoles to generate the corresponding 5-methoxy-, 6-bromo-, 5-bromo-, and 6-fluorooxindoles in 70–91% yield and a TTN range of 31,818–41,364 (Figure 4, **5–8**). Two- and three-carbon linkages on the 3-position of the indole containing methyl esters were well-tolerated in 81% yield and 36,818 TTN with no detected ester hydrolysis (Figure 4, **9–10**). A range of other functional groups were accommodated including an *N*-methoxy-*N*-methyl amide, phthalimide, *N*-acyl primary amine, ketone, and nitrile in 53–92% yield and a TTN range of 24,090–41,818 (Figure 4, **11–15**). To further highlight the functional group tolerance of the catalyst system, a 3-substituted indole containing both an arylmethoxy- and alkylnitrile substituent undergoes indole oxidation to give the corresponding oxindole in 67% yield and a TTN of 30,455 (Figure 4, **16**). To demonstrate the comparative utility of this catalyst system to established methods, reactions to generate the 3-benzyl- and 3-phenyl-substituted oxindoles were performed in 58–79% yield and a TTN range of 26,363–35,909 (Figure 4, **17–18**). These substrates are documented to be incompatible with electrochemical^35^ and other halide recycling systems,^36^ respectively, making this the first catalyst system to accommodate both substrate types. The reaction also tolerates an *N*-methyl substituent on the indole scaffold, giving the corresponding oxindole in 82% yield and TTN of 37,272 (Figure 4, **19**). Finally, the developed protocol could be used directly on the bioactive hormone melatonin to give the corresponding oxindole (**20**) in 79% yield and 35,909 TTN (Figure 4, **20**). Using our developed conditions for spirooxindole formation, the conversion of **3** to **4** is readily performed on a preparative scale in 90% yield and a TTN of 56,250 (Figure 4, **4**) as well as on a gram scale in 89% yield and a TTN of 55,625. The protocol is tolerant of analogous substrates containing *tert*-butyloxycarbonyl- (Boc), 2,2,2-trichloroethoxycarbonyl- (Troc), and methylcarbamate protecting groups in 83–92% yield and TTNs of 51,875–57,500 (Figure 4, **21–23**). Like the indole oxidation protocol, an *N*-methylindole containing substrate is tolerated, generating oxindole in 95% yield and a TTN of 59,375 (Figure 4, **24**). The catalyst system performs on a substrate directly derived from tryptophan to give the corresponding oxindole in 85% yield and a TTN of 53,125 as a single diastereomer (Figure 4, **25**). Gratifyingly, the developed conditions for aza-spirooxindole formation directly translate to the synthesis of oxa-spirooxindoles generating mono- and dispirooxindoles in 75–89% yield and a TTN range of 48,875–55,625 (Figure 4, **26–28**). The system can perform direct hydroxymethyl and acyl migration on acyclic 2,3-substituted indoles to give the corresponding acyclic spirooxindoles in 30–51% yield and 18,750–31,875 TTN (Figure 4, **29–30**). To further highlight the substrate tolerance of the catalyst system, the reaction performs selective oxidation on a tryptophan-containing dipeptide in >95% conversion (Figure 4, **31**). Notably, no enantioselectivity was observed across the substrate scope, and a detailed investigation of these findings is currently underway in our laboratory.

**Figure 4:**
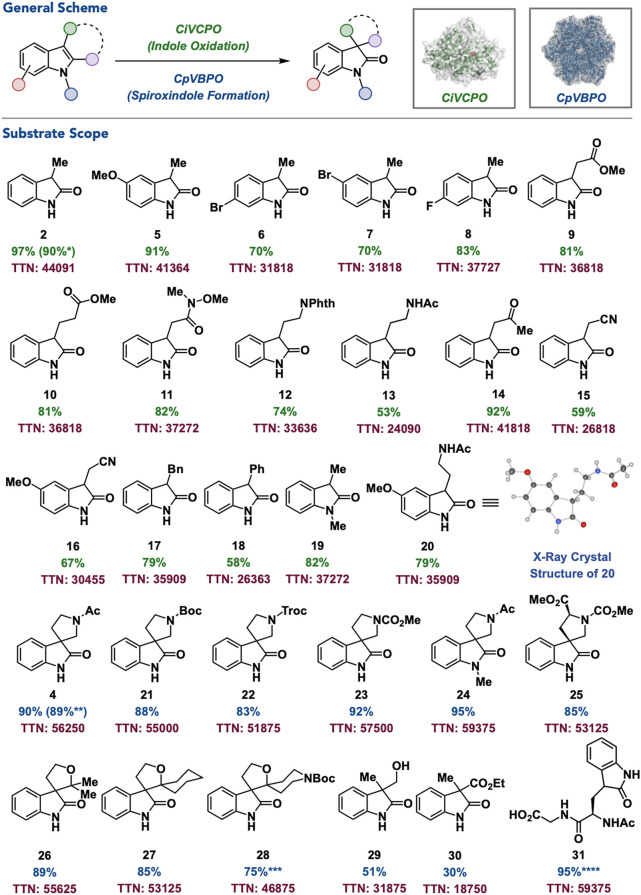
Substrate scope for oxidative rearrangement of indoles. Standard reaction conditions for indole oxidation: **substrate** (0.4 mmol), *Ci*VCPO whole cells (0.0022 mol %), Na_3_VO_4_ (0.1 mM final concentration), KBr (0.2 equiv), H_2_O_2_ (2.0 equiv), citrate buffer (100 mM, pH = 5), MeCN (30%), 4 h, rt. Yields determined by isolation. TTNs were determined by dividing the quantity of the resulting product by the concentration of the enzyme used. See the Supporting Information for more details. *Gram-scale reaction. Standard reaction conditions for spirooxindole formation: **substrate** (0.4 mmol), *Cp*VBPO (0.0016 mol %), Na_3_VO_4_ (1.0 mM final concentration), KBr (0.1 equiv), H_2_O_2_ (2.0 equiv), citrate buffer (50 mM, pH = 5), DMF (10%), 2 h, rt. Yields determined by isolation. TTNs were determined by dividing the quantity of the resulting product by the concentration of the enzyme used. See the Supporting Information for more details. **Gram-scale reaction. ***20% DMF (v/v) used as cosolvent. ****Percent represents conversion.

A proposed mechanism for the VHPO-catalyzed oxidative rearrangement of indoles is outlined in Figure 5. In analogy to previously proposed mechanisms,^62,63,92^ the vanadate cofactor is bound to a histidine residue in the enzyme active site (**I**). Exposure of **I** to H_2_O_2_ causes displacement of two water molecules to generate the corresponding peroxovanadium intermediate **II**. A subsequent nucleophilic attack of halide leads to the ring opening of **II**, leaving a vanadium-bound hypohalite (**III**) that can either participate directly in a halogenation event or be released from the coordination sphere as the corresponding hypohalous acid that would serve as the halogenating agent. We propose that one of these events is ultimately responsible for an indole (**IV**) halogenation event leading to the corresponding 3-halo-2-hydroxyindoline (**V**) after the trapping of the intermediate iminium ion with water from the aqueous solution. Like other established catalytic halogenating systems for oxidative rearrangement of indoles,^35,36^ the formation of **V** would lead to spontaneous semipinacol rearrangement, giving either the corresponding 3-substituted oxindole (**VI**) or 3-disubstituted oxindole (**VII**), completing a net oxidative rearrangement through a cryptic halogenation mechanism and releasing the starting halide to be recycled by the VHPO.

**Figure 5:**
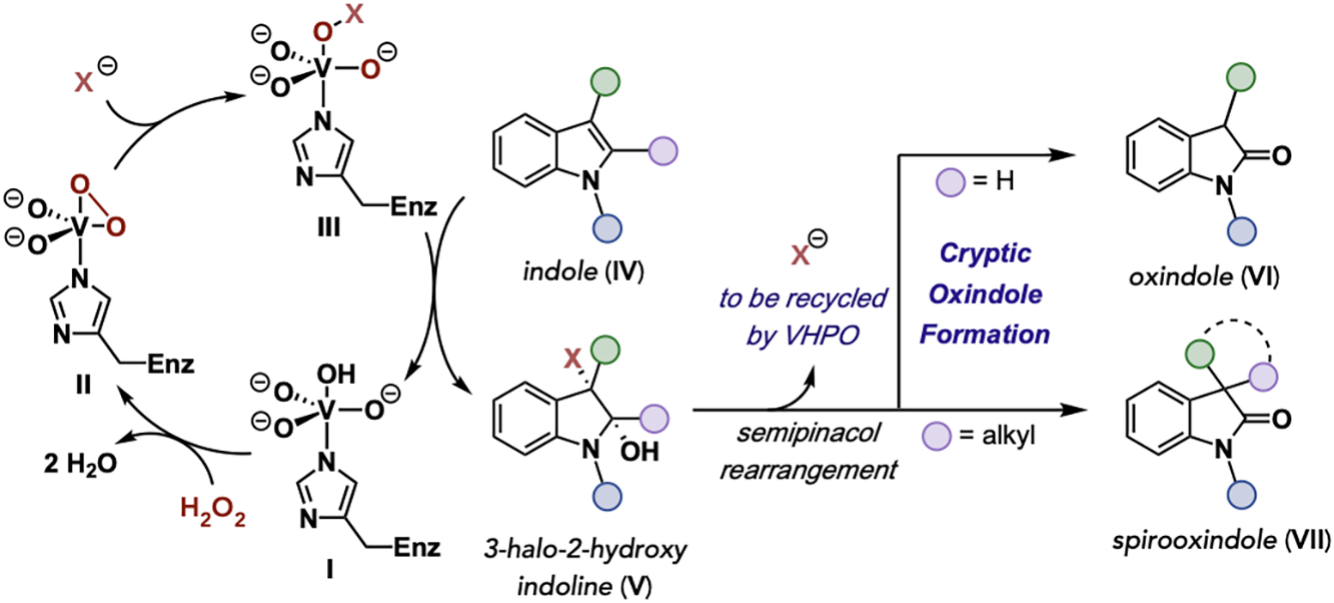
Proposed mechanism for oxidative rearrangement.

With an understanding of the substrate scope capabilities of the VHPO-catalyzed oxidative rearrangement of indoles, further exploration of its synthetic applicability was interrogated. When using purified *Ci*VCPO for indole oxidation, the catalyst loading could be decreased to as low as 2.5 × 10^–5^ mol % while increasing the loading of KBr to 0.3 equiv to give **2** in 96% yield and a TTN of 3.84 million, nearly doubling the best reported TTN of 2.0 million for a VHPO to date (Figure 6a).^93^ Similarly, *Cp*VBPO-catalyzed spirooxindole formation could be performed with as low as 1.0 × 10^–4^ mol % catalyst loading, providing spirooxindole **4** in 93% yield and a TTN of 930,000 (Figure 6b). By simply switching the reaction medium to deuterated solvents, the developed indole oxidation process can be used to perform an oxydeuteration of indoles, exemplified in the conversion of indole **1** to the corresponding oxindole **2-d** in 93% yield, a TTN of 1.86 million, and 82% deuterium incorporation (Figure 6c). With an interest in incorporating oxidative rearrangement of indoles into multienzyme sequences, VHPO-catalyzed indole oxidation is readily coupled to lipase-mediated ester hydrolysis in the conversion of methyl 2-(1*H*-indole-3-yl)­acetate (**32**) to the corresponding carboxylic acid-containing spirooxindole, 2-(2-oxoindolin-3-yl)­acetic acid (**33**), in 96% over two steps (Figure 6d). As an entryway into new chemoenzymatic heterocycle synthesis, VHPO-catalyzed indole oxidation was coupled to palladium-catalyzed cross-coupling and ring expansion to convert 2-(1*H*-indol-3-yl)­acetonitrile (**34**) to the corresponding quinoline, methyl 2-phenylquinoline-4-carboxylate (**35**), in 78% yield over two steps (Figure 6e).^94^ Finally, during reaction development, an interesting discovery was made while applying this protocol to a 2,3-disubstituted indole derived from tryptophan methyl ester and formaldehyde (**36**). When **36** was subjected to *Ci*VCPO under standard conditions with bromide as the halide, the corresponding spirooxindole (**37**) was generated in 76% yield and a TTN of 47,500. In contrast, when the same compound was subjected to conditions using the identical biocatalyst and chloride as the halide, methyl 9*H*-pyrido­[3,4-*b*]­indole-3-carboxylate (**38**) was generated in 71% yield and a TTN of 44,375 (Figure 6f). These results serve as a unique example of halide divergent EHR for a VHPO in organic synthesis. The mechanistic details of this reactivity divergence are currently under investigation in our laboratory.

**Figure 6:**
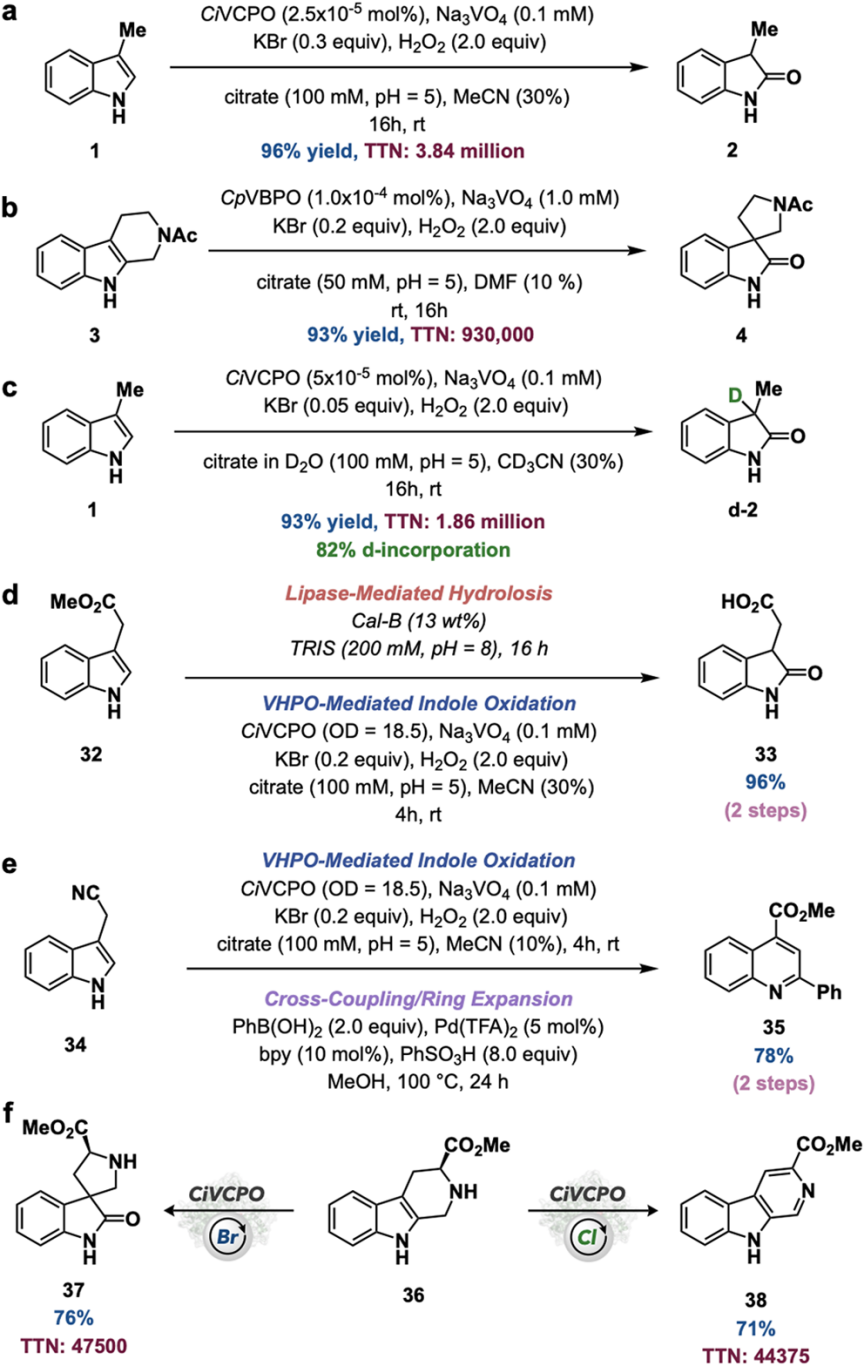
Synthetic applicability experiments. (a) High TTN indole oxidation experiment with purified *Ci*VCPO. (b) High TTN spirooxindole formation with *Cp*VBPO. (c) Oxydeuteration of indoles. (d) Multienzymatic ester hydrolysis/VHPO-mediated oxindole formation. (e) Chemoenzymatic synthesis of quinolines. (f) Halide divergent reactivity of *Ci*VCPO.

One of the major goals of this work is the development of a catalyst system for the late-stage functionalization or synthesis of biologically active molecules. Encouraged by the oxidation results for dipeptide **31**, we were interested in testing this reaction type on more complex peptides in tryptophan-selective peptide modification. Gratifyingly, using *Cp*VBPO as the biocatalyst, this desired transformation is performed on tyrosine- and histidine-containing tripeptides in 95% conversion, with a TTN of 59,375, and with complete tryptophan selectivity (Figure 7a, **39–40**). Remarkably, this protocol was extended to 8- and 9-mer peptides in 95% conversion, a TTN of 59,375, and complete selectivity over phenylalanine, tyrosine, lysine, asparagine, and guanidine residues (Figure 7a, **41–42**). Finally, *Cp*VBPO-catalyzed oxidative rearrangement was applied to the gram-scale synthesis of spirooxindole natural products coerulescine (conversion of **43** to **44**) in 73% yield and a TTN of 45,625 and horsfiline (conversion of **45** to **46**) in 82% yield and a TTN of 51,250 (Figure 7b).

**Figure 7:**
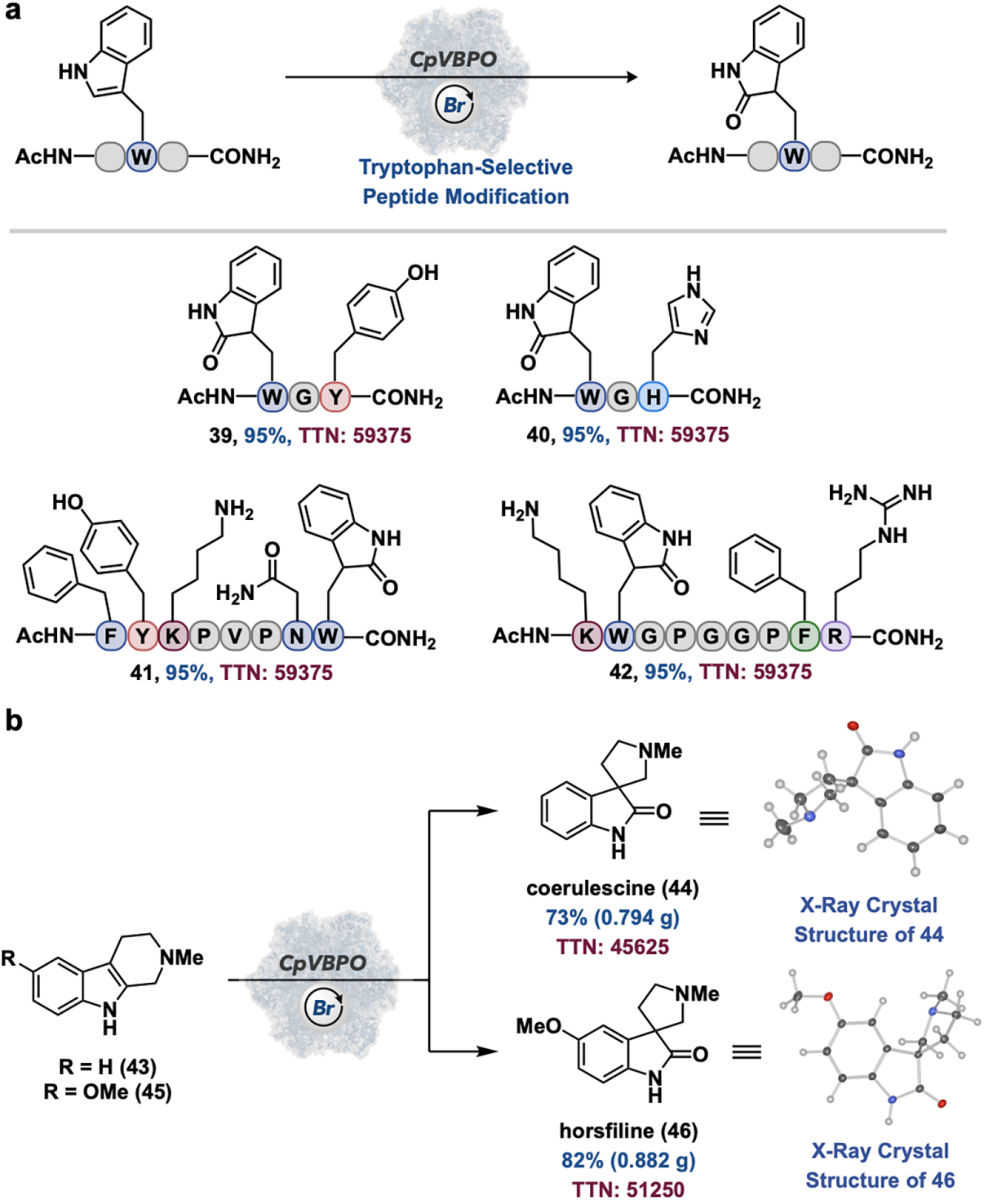
Tryptophan-selective peptide modification and biocatalytic synthesis of natural products. (a) Tryptophan-selective peptide modification (b) Gram-scale biocatalytic synthesis of coerulescine and horsfiline.

## Conclusion

In conclusion, VHPOs serve as a general biocatalytic platform for the oxidative rearrangement of indoles. This catalyst system is effective for both direct indole oxidation of 3-substituted indoles and spirooxindole formation from 2,3-substituted indoles in moderate to high yield, high TTN, and excellent regioselectivity. This biocatalytic protocol has been applied in enzymatic and chemoenzymatic cascades, oxydeuteration of indoles, tryptophan-selective peptide modification, and natural product synthesis. These studies not only demonstrate the versatility of VHPOs to perform oxidative rearrangements of indoles but also expand their synthetic application in chemoenzymatic synthesis.

## Supplementary Material



## Notes

Methods, experimental procedures, and characterization data, including supplementary figures and tables (PDF)

## References

[1] Sharma S., Monga Y., Gupta A., Singh S. (2023). 2-Oxindole and Related
Heterocycles: Synthetic Methodologies for Their Natural Products and
Related Derivatives. RSC Adv..

[2] Kaur M., Singh M., Chadha N., Silakari O. (2016). Oxindole: A Chemical
Prism Carrying Plethora of Therapeutic Benefits. Eur. J. Med. Chem..

[3] Khetmalis Y. M., Shivani M., Murugesan S., Chandra Sekhar K. V. G. (2021). Oxindole
and Its Derivatives: A Review on Recent Progress in Biological Activities. Biomed. Pharmacother..

[4] Norwood V. M., Huigens R. W. (2019). Harnessing the Chemistry
of the Indole Heterocycle
to Drive Discoveries in Biology and Medicine. ChemBioChem.

[5] Aguilar A., Sun W., Liu L., Lu J., McEachern D., Bernard D., Deschamps J. R., Wang S. (2014). Design of Chemically
Stable, Potent, and Efficacious MDM2 Inhibitors That Exploit the Retro-Mannich
Ring-Opening-Cyclization Reaction Mechanism in Spiro-Oxindoles. J. Med. Chem..

[6] Ziarani G. M., Gholamzadeh P., Lashgari N., Hajiabbasi P. (2013). Oxindole as
Starting Material in Organic Synthesis. Arkivoc.

[7] Yeung B. K., Zou B., Rottmann M., Lakshminarayana S. B., Ang S. H., Leong S. Y., Tan J., Wong J., Keller-Maerki S., Fischli C., Goh A., Schmitt E. K., Krastel P., Francotte E., Kuhen K., Plouffe D., Henson K., Wagner T., Winzeler E. A., Petersen F., Brun R., Dartois V., Diagana T. T., Keller T. H. (2010). Spirotetrahydro β-Carbolines
(Spiroindolones): A New Class of Potent and Orally Efficacious Compounds
for the Treatment of Malaria. J. Med. Chem..

[8] Galliford C. V., Scheidt K. A. (2007). Pyrrolidinyl-Spirooxindole
Natural Products as Inspirations
for the Development of Potential Therapeutic Agents. Angew. Chem., Int. Ed..

[9] Edmondson S. D., Danishefsky S. J., Sepp-Lorenzino L., Rosen N. (1999). Total Synthesis of
Spirotryprostatin A, Leading to the Discovery of Some Biologically
Promising Analogues. J. Am. Chem. Soc..

[10] Qian C., Li P., Sun J. (2021). Catalytic
Enantioselective Synthesis of Spirooxindoles
by Oxidative Rearrangement of Indoles. Angew.
Chem., Int. Ed..

[11] Gao B., Xu S., Du T., Li Y. (2020). Transition-Metal-Free Catalyzed Dearomatizative
Esterification of Indole. ChemistrySelect.

[12] Budovská M., Tischlerová V., Mojžiš J., Kozlov O., Gondová T. (2020). An Alternative
Approach to the Synthesis of Anticancer
Molecule Spirobrassinin and Its 2′-Amino Analogues. Monatsh. Chem..

[13] Peng X., Zeng Y., Liu H., Xu X., Zhang M., Liu Q. (2019). From Indoles to 3,3′-Biindolin-2-Ones:
Copper-Catalyzed Oxidative
Homocoupling of Indoles. New J. Chem..

[14] Seiler G. S., Hughes C. C. (2019). Progress toward
the Total Synthesis of Lymphostins:
Preparation of a Functionalized Tetrahydropyrrolo­[4,3,2-De]­Quinoline
and Unusual Oxidative Dimerization. J. Org.
Chem..

[15] Shelar S. V., Argade N. P. (2019). Regioselective Oxidation of Indoles to 2-Oxindoles. Org. Biomol. Chem..

[16] Talukdar R. (2019). A Study on
the Reactions of SeO2 with Pyrroles and N-Substituted Indoles in Non-Anhydrous
Ethanol under Non-Inert Atmosphere. Asian J.
Org. Chem..

[17] Jiang X., Zheng C., Lei L., Lin K., Yu C. (2018). Synthesis
of 2-Oxindoles from Substituted Indoles by Hypervalent-Iodine Oxidation. Eur. J. Org Chem..

[18] von
Drathen T., Hoffmann F., Brasholz M. (2018). Visible-Light Catalytic
Photooxygenation of Monoterpene Indole Alkaloids: Access to Spirooxindole-1,3-Oxazines. Chem.—Eur. J..

[19] Wang L., Qu X., Fang L., Li Z., Hu S., Wang F. (2016). Synthesis
of 3-Acetoxyoxindole Derivatives by Metal-Free PhI­(OAc)­2-Mediated
Oxidation of 3-Substituted Indoles. Eur. J.
Org Chem..

[20] Li G., Huang L., Xu J., Sun W., Xie J., Hong L., Wang R. (2016). Sodium Iodide/Hydrogen
Peroxide-Mediated Oxidation/Lactonization for the Construction of
Spirocyclic Oxindole-Lactones. Adv. Synth. Catal..

[21] Lin F., Chen Y., Wang B., Qin W., Liu L. (2015). Silver-Catalyzed
TEMPO Oxidative Homocoupling of Indoles for the Synthesis of 3,3′-Biindolin-2-Ones. RSC Adv..

[22] Bathula C., Dangi P., Hati S., Agarwal R., Munshi P., Singh A., Singh S., Sen S. (2015). Diverse Synthesis of
Natural Product Inspired Fused and Spiro-Heterocyclic Scaffolds via
Ring Distortion and Ring Construction Strategies. New J. Chem..

[23] Kolundzic F., Noshi M. N., Tjandra M., Movassaghi M., Miller S. J. (2011). Chemoselective and
Enantioselective Oxidation of Indoles
Employing Aspartyl Peptide Catalysts. J. Am.
Chem. Soc..

[24] Alamgir M., Mitchell P. S. R., Bowyer P. K., Kumar N., Black D. S. (2008). Synthesis
of 4,7-Indoloquinones from Indole-7-Carbaldehydes by Dakin Oxidation. Tetrahedron.

[25] Lazzaro F., Crucianelli M., De Angelis F., Neri V., Saladino R. (2004). A Novel Oxidative
Side-Chain Transformation of α-Amino Acids and Peptides by Methyltrioxorhenium/H2O2
System. Tetrahedron Lett..

[26] Peterson A. C., Cook J. M. (1994). Studies on the Enantiospecific
Synthesis of Oxindole
Alkaloids. Tetrahedron Lett..

[27] Zhang X., Foote C. S. (1993). Dimethyldioxirane
Oxidation of Indole Derivatives.
Formation of Novel Indole-2,3-Epoxides and a Versatile Synthetic Route
to Indolinones and Indolines. J. Am. Chem. Soc..

[28] Yoshida K., Goto J., Ban Y. (1987). Oxidation
of Cycloalkan [*b*] Indoles with Iodine Pentoxide­(I2O5). Chem. Pharm. Bull..

[29] Savige W. E., Fontana A. (1976). New Procedure for Oxidation of 3-Substituted Indoles
to Oxindoles: Modification of Tryptophan Residues in Peptides and
Proteins. J. Chem. Soc., Chem. Commun..

[30] Hinman R. L., Bauman C. P. (1964). Reactions of N-Bromosuccinimide and
Indoles. A Simple
Synthesis of 3-Bromooxindoles. J. Org. Chem..

[31] Finch N., Taylor W. I. (1962). Oxidative Transformations
of Indole Alkaloids. I. The
Preparation of Oxindoles from Yohimbine; the Structures and Partial
Syntheses of Mitraphylline, Rhyncophylline and Corynoxeine. J. Am. Chem. Soc..

[32] Finch N., Taylor W. I. (1962). The Conversion of Tetrahydro-β-Carboline Alkaloids
into Oxindoles. The Structures and Partial Syntheses of Mitraphylline
and Rhyncophylline. J. Am. Chem. Soc..

[33] Zheng Y., Cheung Y. T., Liang L., Qiu H., Zhang L., Tsang A., Chen Q., Tong R. (2022). Electrochemical
Oxidative
Rearrangement of Tetrahydro-β-Carbolines in a Zero-Gap Flow
Cell. Chem. Sci..

[34] Liu D., Xu H.-C. (2022). Electrochemical Rearrangement of Indoles to Spirooxindoles in Continuous
Flow. Eur. J. Org Chem..

[35] Arteaga
Giraldo J. J., Lindsay A. C., Seo R. C.-Y., Kilmartin P. A., Sperry J. (2023). Electrochemical Oxidation of 3-Substituted Indoles. Org. Biomol. Chem..

[36] Xu J., Liang L., Zheng H., Chi Y. R., Tong R. (2019). Green Oxidation
of Indoles Using Halide Catalysis. Nat. Commun..

[37] Zhao G., Liang L., Wang E., Lou S., Qi R., Tong R. (2021). Fenton Chemistry Enables the Catalytic
Oxidative Rearrangement of
Indoles Using Hydrogen Peroxide. Green Chem..

[38] Buller R., Lutz S., Kazlauskas R. J., Snajdrova R., Moore J. C., Bornscheuer U. T. (2023). From Nature
to Industry: Harnessing
Enzymes for Biocatalysis. Science.

[39] Bell E. L., Finnigan W., France S. P., Green A. P., Hayes M. A., Hepworth L. J., Lovelock S. L., Niikura H., Osuna S., Romero E., Ryan K. S., Turner N. J., Flitsch S. L. (2021). Biocatalysis. Nat. Rev. Methods
Primers.

[40] Li H., Mei L., Urlacher V. B., Schmid R. D. (2008). Cytochrome P450 BM-3 Evolved by Random
and Saturation Mutagenesis as an Effective Indole-Hydroxylating Catalyst. Appl. Biochem. Biotechnol..

[41] Nakamura K., Martin M. V., Guengerich F. P. (2001). Random
Mutagenesis of Human Cytochrome
P450 2A6 and Screening with Indole Oxidation Products1. Arch. Biochem. Biophys..

[42] Gillam E. M. J., Notley L. M., Cai H., De Voss J. J., Guengerich F. P. (2000). Oxidation
of Indole by Cytochrome P450 Enzymes. Biochemistry.

[43] Hinman R. L., Lang J. (1965). Peroxidase-Catalyzed
Oxidation of Indole-3-Acetic Acid. Biochemistry.

[44] Kuo H. H., Mauk A. G. (2012). Indole Peroxygenase
Activity of Indoleamine 2,3-Dioxygenase. Proc.
Natl. Acad. Sci. U.S.A..

[45] Krieg T., Hüttmann S., Mangold K.-M., Schrader J., Holtmann D. (2011). Gas Diffusion
Electrode as Novel Reaction System for an Electro-Enzymatic Process
with Chloroperoxidase. Green Chem..

[46] Bai C., Jiang Y., Hu M., Li S., Zhai Q. (2009). Improvement
of Chloroperoxidase Catalytic Activities by Chitosan and Thioglycolic
Acid. Catal. Lett..

[47] Lichtenecker R. J., Schmid W. (2009). Application of Various
Ionic Liquids as Cosolvents
for Chloroperoxidase-Catalysed Biotransformations. Monatsh. Chem..

[48] Alvarez R. G., Hunter I. S., Suckling C. J., Thomas M., Vitinius U. (2001). A Novel Biotransformation
of Benzofurans and Related Compounds Catalysed by a Chloroperoxidase. Tetrahedron.

[49] van
Deurzen M. P. J., van Rantwijk F., Sheldon R. A. (1996). Synthesis of Substituted
Oxindoles by Chloroperoxidase Catalyzed Oxidation of Indoles. J. Mol. Catal. B: Enzym..

[50] Nguyen T.-A. M., Grzech D., Chung K., Xia Z., Nguyen T.-D., Dang T.-T. T. (2023). Discovery of a Cytochrome P450 Enzyme
Catalyzing the
Formation of Spirooxindole Alkaloid Scaffold. Front. Plant Sci..

[51] Zhang Y., Zou Y., Brock N. L., Huang T., Lan Y., Wang X., Deng Z., Tang Y., Lin S. (2017). Characterization of
2-Oxindole Forming Heme Enzyme MarE, Expanding the Functional Diversity
of the Tryptophan Dioxygenase Superfamily. J.
Am. Chem. Soc..

[52] Tsunematsu Y., Ishikawa N., Wakana D., Goda Y., Noguchi H., Moriya H., Hotta K., Watanabe K. (2013). Distinct Mechanisms
for Spiro-Carbon Formation Reveal Biosynthetic Pathway Crosstalk. Nat. Chem. Biol..

[53] Li S., Finefield J. M., Sunderhaus J. D., McAfoos T., Williams R. M., Sherman D. H. (2012). Biochemical Characterization of NotB as an FAD-Dependent
Oxidase in the Biosynthesis of Notoamide Indole Alkaloids. J. Am. Chem. Soc..

[54] Chu D., Wang H., Nie Z., Li K.-L., Cao J., Yang M., Yin Q., Gu Y., Jiang Y. (2025). Collective
Biosynthesis of Plant Spirooxindole Alkaloids through Enzyme Discovery
and Engineering. J. Am. Chem. Soc..

[55] Zhao Q., Zhang R., Döbber J., Gulder T. (2025). Chemoenzymatic C,C-Bond
Forming Cascades by Cryptic Vanadium Haloperoxidase Catalyzed Bromination. Org. Lett..

[56] Krongyut C., Wiriya N., Saiyasombat W., Chansaenpak K., Sripattanakul S., Kamkaew A., Lai R. (2025). Chemoenzymatic
Cyclization
by Vanadium Chloroperoxidase for Synthesis of 4-Hydroxyisochroman-1-Ones. ChemBioChem.

[57] Zeides P., Bellmann-Sickert K., Zhang R., Seel C. J., Most V., Schoeder C. T., Groll M., Gulder T. (2025). Unraveling the Molecular
Basis of Substrate Specificity and Halogen Activation in Vanadium-Dependent
Haloperoxidases. Nat. Commun..

[58] Branham P. J., Saha N., Oyelere S. E., Agarwal V. (2025). Halogenase-Assisted
Biocatalytic Derivatization of Aminothiazoles and Cephalosporin Antibiotics. J. Org. Chem..

[59] Baumgartner J. T., McKinnie S. M. K. (2024). Regioselective Halogenation of Lavanducyanin by a Site-Selective
Vanadium-Dependent Chloroperoxidase. Org. Lett..

[60] Höfler G. T., But A., Hollmann F. (2019). Haloperoxidases
as Catalysts in Organic Synthesis. Org. Biomol.
Chem..

[61] Hegarty E., Büchler J., Buller R. (2023). Halogenases for the Synthesis of
Small Molecules. Curr. Opin. Green Sustainable
Chem..

[62] Latham J., Brandenburger E., Shepherd S. A., Menon B. R. K., Micklefield J. (2018). Development
of Halogenase Enzymes for Use in Synthesis. Chem. Rev..

[63] Agarwal V., Miles Z. D., Winter J. M., Eustáquio A. S., El Gamal A. A., Moore B. S. (2017). Enzymatic Halogenation
and Dehalogenation
Reactions: Pervasive and Mechanistically Diverse. Chem. Rev..

[64] Winter J. M., Moore B. S. (2009). Exploring the Chemistry and Biology of Vanadium-Dependent
Haloperoxidases. J. Biol. Chem..

[65] Adak S., Moore B. S. (2021). Cryptic Halogenation
Reactions in Natural Product Biosynthesis. Nat.
Prod. Rep..

[66] Khare D., Wang B., Gu L., Razelun J. R., Sherman D. H., Gerwick W. H., Håkansson K., Smith J. L. (2010). Conformational Switch
Triggered by α-Ketoglutarate in a Halogenase of Curacin a Biosynthesis. Proc. Natl. Acad. Sci. U.S.A..

[67] Neumann C. S., Walsh C. T. (2008). Biosynthesis of (−)-(1S,2R)-Allocoronamic
Acyl
Thioester by an FeII-Dependent Halogenase and a Cyclopropane-Forming
Flavoprotein. J. Am. Chem. Soc..

[68] Strieter E. R., Vaillancourt F. H., Walsh C. T. (2007). CmaE: A Transferase Shuttling Aminoacyl
Groups between Carrier Protein Domains in the Coronamic Acid Biosynthetic
Pathway. Biochemistry.

[69] Kelly W. L., Boyne M. T., Yeh E., Vosburg D. A., Galonić D. P., Kelleher N. L., Walsh C. T. (2007). Characterization of the Aminocarboxycyclopropane-Forming
Enzyme CmaC. Biochemistry.

[70] Vaillancourt F. H., Yeh E., Vosburg D. A., O’Connor S. E., Walsh C. T. (2005). Cryptic Chlorination
by a Non-Haem Iron Enzyme during Cyclopropyl Amino Acid Biosynthesis. Nature.

[71] Chang Z., Sitachitta N., Rossi J. V., Roberts M. A., Flatt P. M., Jia J., Sherman D. H., Gerwick W. H. (2004). Biosynthetic Pathway and Gene Cluster
Analysis of Curacin A, an Antitubulin Natural Product from the Tropical
Marine Cyanobacterium Lyngbya Majuscula. J.
Nat. Prod..

[72] Ullrich M., Bender C. L. (1994). The Biosynthetic
Gene Cluster for Coronamic Acid, an
Ethylcyclopropyl Amino Acid, Contains Genes Homologous to Amino Acid-Activating
Enzymes and Thioesterases. J. Bacteriol..

[73] Marchand J. A., Neugebauer M. E., Ing M. C., Lin C.-I., Pelton J. G., Chang M. C. Y. (2019). Discovery
of a Pathway for Terminal-Alkyne Amino Acid
Biosynthesis. Nature.

[74] Kolb H. C., Finn M. G., Sharpless K. B. (2001). Click Chemistry:
Diverse Chemical
Function from a Few Good Reactions. Angew. Chem.,
Int. Ed..

[75] Sanada M., Miyano T., Iwadare S. (1986). β -Ethynylserine,
an Antimetabolite
of L-Threonine, from Streptomyces Cattleya. J. Antibiot..

[76] Martins T. P., Rouger C., Glasser N. R., Freitas S., de Fraissinette N. B., Balskus E. P., Tasdemir D., Leão P. N. (2019). Chemistry,
Bioactivity and Biosynthesis of Cyanobacterial Alkylresorcinols. Nat. Prod. Rep..

[77] Nakamura H., Schultz E. E., Balskus E. P. (2017). A New Strategy for Aromatic Ring
Alkylation in Cylindrocyclophane Biosynthesis. Nat. Chem. Biol..

[78] Leão P. N., Nakamura H., Costa M., Pereira A. R., Martins R., Vasconcelos V., Gerwick W. H., Balskus E. P. (2015). Biosynthesis-Assisted
Structural Elucidation of the Bartolosides, Chlorinated Aromatic Glycolipids
from Cyanobacteria. Angew. Chem., Int. Ed..

[79] Moore B. S., Chen J.-L., Patterson G. M. L., Moore R. E. (1992). Structures of Cylindrocyphanes
A-f. Tetrahedron.

[80] Moore B. S., Chen J. L., Patterson G. M., Moore R. E., Brinen L. S., Kato Y., Clardy J. (1990). [7.7]­Paracyclophanes
from Blue-Green
Algae. J. Org. Chem..

[81] El
Gamal A., Agarwal V., Diethelm S., Rahman I. A., Schorn M., Sneed J. M., Louie G. V., Whalen K. E., Mincer T. J., Noel J. P., Paul V. J., Moore B. S. (2016). Biosynthesis
of Coral Settlement Cue Tetrabromopyrrole in Marine Bacteria by a
Uniquely Adapted Brominase–Thioesterase Enzyme Pair. Proc. Natl. Acad. Sci. U.S.A..

[82] Yamanaka K., Ryan K. S., Gulder T. A. M., Hughes C. C., Moore B. S. (2012). Flavoenzyme-Catalyzed
Atropo-Selective N,C-Bipyrrole Homocoupling in Marinopyrrole Biosynthesis. J. Am. Chem. Soc..

[83] Martinez J. S., Carroll G. L., Tschirret-Guth R. A., Altenhoff G., Little R. D., Butler A. (2001). On the Regiospecificity of Vanadium
Bromoperoxidase. J. Am. Chem. Soc..

[84] Murray L. A. M., McKinnie S. M. K., Pepper H. P., Erni R., Miles Z. D., Cruickshank M. C., López-Pérez B., Moore B. S., George J. H. (2018). Total Synthesis
Establishes the Biosynthetic
Pathway to the Naphterpin and Marinone Natural Products. Angew. Chem., Int. Ed..

[85] Wells C. E., Ramos L. P. T., Harstad L. J., Hessefort L. Z., Lee H. J., Sharma M., Biegasiewicz K. F. (2023). Decarboxylative
Bromooxidation of Indoles by a Vanadium Haloperoxidase. ACS Catal..

[86] Sharma M., Pascoe C. A., Jones S. K., Barthel S. G., Davis K. M., Biegasiewicz K. F. (2025). Intermolecular 1,2,4-Thiadiazole
Synthesis Enabled
by Enzymatic Halide Recycling with Vanadium-Dependent Haloperoxidases. J. Am. Chem. Soc..

[87] Sharma M., Li Y., Biegasiewicz K. F. (2025). Biocatalytic Thioketal Cleavage Enabled
by Enzymatic Bromide Recycling by Vanadium-Dependent Haloperoxidases. Org. Lett..

[88] van
Schijndel J. W. P. M., Vollenbroek E. G. M., Wever R. (1993). The Chloroperoxidase
from the Fungus Curvularia Inaequalis; a Novel Vanadium Enzyme. Biochim. Biophys. Acta.

[89] Yu H., Whittaker J. W. (1989). Vanadate Activation of Bromoperoxidase from Corallinaofficinalis. Biochem. Biophys. Res. Commun..

[90] Itoh N., Izumi Y., Yamada H. (1986). Characterization of
Nonheme Type
Bromoperoxidase in Corallina Pilulifera. J.
Biol. Chem..

[91] Frank A., Seel C. J., Groll M., Gulder T. (2016). Characterization of
a Cyanobacterial Haloperoxidase and Evaluation of Its Biocatalytic
Halogenation Potential. ChemBioChem.

[92] Wever R., Krenn B. E., Renirie R. (2018). Marine Vanadium-Dependent
Haloperoxidases,
Their Isolation, Characterization, and Application. Methods Enzymol..

[93] Fernández-Fueyo E., van Wingerden M., Renirie R., Wever R., Ni Y., Holtmann D., Hollmann F. (2015). Chemoenzymatic Halogenation of Phenols
by Using the Haloperoxidase from Curvularia Inaequalis. ChemCatChem.

[94] Zhao Z., Zeng G., Chen Y., Zheng J., Chen Z., Shao Y., Zhang F., Chen J., Li R. (2021). Palladium-Catalyzed
Three-Component Cascade Reaction of Nitriles: Synthesis of 2-Arylquinoline-4-Carboxylates. Org. Lett..

