# Supplementary material for: Oxidative Rearrangement of Indoles Enabled by Promiscuous Cryptic Halogenation with Vanadium-Dependent Haloperoxidases
Source: ACS Catal. 2026 Jan 14;16(3):2606–14. doi: 10.1021/acscatal.5c07839 (PMC12887939; doi:10.1021/acscatal.5c07839)
Supplement: Supplementary file 1 [file cs5c07839_si_001.pdf]

Supplementary Materials for

Oxidative Rearrangement of Indoles Enabled by Promiscuous Cryptic Halogenation with Vanadium-Dependent Haloperoxidases

Hyung Ji Lee[1], Carter U. Brzezinski[1], Sergio A. Solis[1], Raina S. Semenick[2], Ana Villalobos Galindo[1], Sophia G. Barthel[1], John Bacsa[1], Kyle F. Biegasiewicz[1,2*]

[1] Department of Chemistry, Emory University, Atlanta, GA 30322, United States
[2] School of Molecular Sciences, Arizona State University, Tempe, AZ 85281, United States

Corresponding Author E-mail: kbiegas@emory.edu

This PDF file includes:

Materials and Methods
Product Characterization
Supplementary Text and Figures
Figs. S1-S19
Spectroscopic Data
References

Table of Contents

General Experimental Information 3

Expression and Purification of CiVCPO and CpVBPO 4

Gel Electrophoresis of VHPO Enzymes 6

Preparation of Whole Cells Absent of CiVCPO for Control Experiments 7

Synthesis and Characterization of 3-Substituted Indole Substrates 10

Synthesis and Characterization of 2,3-Disubstituted Indole Substrates 11

General Analytical Procedures for Oxidation of 3-Monosubstituted Indoles 25

General Preparative Procedures for Oxidation of 3-Monosubstituted Indoles 28

General Analytical Procedure for Spirooxindole Formation 30

General Preparative Procedure for Spirooxdindole Formation 31

Product Characterization 32

Additional Reaction Experiments 61

Optimization Data 93

References 99

Spectroscopic Data 101

General Experimental Information

General: Unless specified, all reagents and solvents used in this study were purchased from commercial suppliers and used as received (Combi-Blocks, Sigma-Aldrich, Oakwood Chemicals, Fischer Scientific, VWR). CalB immo Plus™ was purchased from Strem Chemicals (Catalogue No. 07-3130). All nonaqueous reactions were performed using glassware that was flame-dried and capped with a rubber septum under nitrogen atmosphere using an inlet and outlet needle connected to a mineral oil bubbler. All aqueous reactions were conducted using glassware without flame-drying prior to experimental set up and without nitrogen atmosphere. For experiments requiring dried or degassed solvent, it was obtained from a solvent purification system from Pure Process Technology. Unless otherwise indicated, deionized water (H_2O) was used in any experiments where H_2O is included in the procedure. Ultrapure Milli-Q Water (Milli-Q H_2O) was accessed through a Milli-Q® EQ 7000 Ultrapure Water Purification System. Reagents including BugBuster® (Sigma-Aldrich, 70584-3), 4X Laemmli Buffer (Bio-Rad, 1610747), 2-mercaptoethanol (Sigma-Aldrich, M6250), Precision Plus Protein Dual Color Standards (Bio-Rad, 1610374), IPTG (Sigma-Aldrich, 16758), Kanamycin Sulfate (VWR, 0408-EU), Deoxyribonuclease from bovine pancreas (Sigma Aldrich, DN25) were purchased from indicated vendors.

Chromatography: Flash chromatography was performed on SiliaFlash® P60 (230-400 mesh, particle size 0.040-0.063 mm) using the listed solvent systems in each procedure. Thin-layer chromatography (TLC) was performed using Uniplate HLF 250 micron F254 precoated glass plates and preparative TLC was performed on Uniplate GF 1000 micron F254 precoated glass plates. For TLC analysis, a short-wave UV lamp and/or plate staining was used.

Spectroscopy and HRMS Analysis: ^{1}H- and ^{13}C-NMR were obtained on a Bruker AVIII or Bruker NEO (400 and 101 MHz, respectively). Chemical shifts are reported in ppm (δ) downfield from tetramethylsilane and are internally referenced to the internal deuterated solvent indicated. ^{1}H-NMR data is reported as follows: chemical shift [multiplicity, coupling constant (Hz), number of hydrogens]. Multiplicities are reported as follows: s (singlet), b (broad signal), d (doublet), dd (doublet of doublets), ddd (doublet of doublet of doublets), t (triplet), dt (doublet of triplets), tt (triplet of triplets), q (quartet), dq (doublet of quartets), p (pentet), m (multiplet). Infrared (IR) were acquired on a Thermo-Fisher Nicolet iS50 spectrometer taken neat, and peaks are reported in frequency of absorption (cm-1). High-resolution mass spectra were obtained on a Thermo Finnigan LTQ-FTMS spectrometer using APCI with an orbitrap mass analyzer.

Analytical: Analytical high-performance liquid chromatography (HPLC) was carried out using a Shimadzu LCMS-2020 System with a Kromasil EternityXT-2.5-C18 column (Dimensions: 4.6 x 50mm, Batch/Serial: 0000016627/A, Part No. XH2CLA05).

Expression and Purification of *Ci*VCPO and *Cp*VBPO

All protein expression and purifications were performed using previously reported procedures,[1] with the following modifications[1]:

The cell stocks of *E. coli* BL21(DE3) cells harboring a plasmid from the respective VHPO were stored at -80 °C as 25% glycerol stocks (comprising a 1:1 mixture of cell culture and sterile 50% glycerol in purified H_2O from a Milli-Q purification system). These glycerol stocks were used to inoculate primary cultures (~ 6 mL) in Turbo Broth™ prepared using purified H_2O from a Milli-Q purification system and containing 50 mg/L kanamycin in a sterile 10 mL culture tube overnight (~16-18 hours) at 26 °C and 260 rpm in an Infors HT Minitron incubator shaker. The primary cultures were used to inoculate Kan-Turbo Broth™ 1 L secondary cultures in 3L Fernbach flasks (6 mL of primary culture per 1 L of secondary culture, final kanamycin concentration of 50 mg/L) and the flasks were incubated in an Infors HT Multitron incubator shaker at 37 °C and 200 rpm until the culture reached an optical density (OD_{600}) between 0.8 and 1.0. After reaching the appropriate OD_{600}, the flask was placed in an ice bath and treated with 100 μL of a 1 M isopropyl β-D-1-thiogalactopyranoside (IPTG) stock [final concentration of 0.1 mM IPTG]. The liter culture was then allowed to shake overnight (~18-20 hours) at 18 °C, shaken at 200 rpm in an Infors HT Multitron incubator shaker. The cell cultures were pelleted using centrifugation at 3.5 krpm at 10 °C for 20 minutes in a Beckman Coulter Avanti JXN-26 centrifuge, and the broth supernatant was discarded. The cell pellets from three 1-L cultures for *Ci*VCPO or two 1-L cultures for *Cp*VBPO were combined into a single sterile 50 mL conical tube, which was then centrifuged at 13,000 rpm at 10 °C for 20 minutes in a Thermo Scientific Sorvall ST Plus Series centrifuge to remove any excess cell broth. These 3-L or 2-L pellets were stored at -80 °C until further use. The cell pellets were thawed in 30 °C water and suspended in Nickel-Nitrilotriacetic acid (Ni-NTA) binding buffer containing 50 mM pH 8 Tris-base/H_2SO_4, 50 mM imidazole, 100 mM Na_2SO_4, and 100 mM NaCl. The pellet was separated into two 50 mL conical tubes with equal volume and was suspended until a combined volume of 45 mL was reached in each conical tube, and the cells were vortexed until fully homogenized using a VWR Analog Vortex Mixer. Once the cells were fully suspended, a 200 mM phenylmethylsulfonyl fluoride (PMSF) stock was added to the suspended pellet solution for a final concentration of 1 mM for each conical tube. The suspended cells were then lysed on ice using a Branson SFX250 Sonifier with a ½ inch probe in 15 second pulses at 32% amplitude, 30 W in 10 bursts with 55 seconds between bursts. The lysed cell solution was then centrifuged at 13,000 rpm at 10 °C for 20 minutes in a Thermo Scientific Sorvall ST Plus Series centrifuge. After centrifugation, the clarified cell lysate was decanted into a 100 mL glass bottle. To reduce the viscosity and the DNA content of the solution, bovine DNase I was added to the lysate. These DNase stocks were prepared by adding 5.2 mg of DNase and ~17 mg of $MgSO_4$ to 10 mL of 5% glycerol and stored at -20 °C. After thawing at room temperature, 1000 μL of this DNase stock was added per ~35 mL of cell lysate (Final concentration of 15 μg/mL). The lysate solution was then placed in an ice bath for 10 minutes. Ni-NTA chromatography purification was performed on the clarified lysate using a 10 mL (2 x 5 mL linked) Cytiva Life Sciences Ni-NTA column with the GE AKTA Start FPLC system. The Ni- NTA method involved 5 column volume (CV) equilibration with binding buffer, loading of the supernatant onto the column, 13 CV of binding buffer (wash), and then a gradient elution from 0 to 100 % elution buffer containing 50 mM pH 7.8 Tris-base/H_2SO_4 and 500 mM imidazole over 6 CV. The flow rate was set to 4 mL/min. Fractions were pooled based on the 280 nm absorbance peak they were associated with. The pooled

fractions were prepared for gel electrophoresis, in addition to samples of diluted lysed cell pellet, lysed supernatant, and Ni-NTA column flow-through. The lysed cell pellet was prepared by mixing a portion of centrifuged pellet with 200 µL of BugBuster® Protein Extraction Reagent, vortexing the mixture, centrifuging for 1 minute, and adding 5 µL of the mixture to a clean microcentrifuge tube. The remaining samples were prepared by adding 5 µL of the lysed supernatant, column flowthrough, or pooled fractions to separate microcentrifuge tubes. To each tube was added 5 µL of 4X Laemmli Buffer and 10 µL of 2-mercaptoethanol. These samples were then shaken in an IKA Matrix orbital shaker at 95 ºC and 300 rpm for 15 minutes. Gel electrophoresis was performed on these samples using 10% sodium dodecyl sulfate Mini- PROTEAN TGX Stain-Free hand casted gels (Bio-Rad Laboratories). A sample gel of *Ci*VCPO is shown in SI Fig. 1 and a sample gel of *Cp*VBPO is shown in SI Fig.2. After this visual confirmation via gel electrophoresis, peak fractions containing protein of approximately the same weight as the Expasy Protparam predicted molecular weight for VHPO were pooled.[2] The pooled peak fractions containing VHPO were desalted using Cytiva desalting (DS) columns on the AKTA Start, loading 1.25 mL sample per 5 mL of DS resin. The desalting method involved 3 column-volume equilibration with storage buffer (For *Ci*VCPO: 25 mM pH 6 Citrate/H_2SO_4 buffer, 25 mM Na_2SO_4, 2.5 mM $Ca(NO_3)_2$; For *Cp*VBPO: 25 mM pH 6.5 PIPES/H_2SO_4 buffer, 25 mM Na_2SO_4, 2.5 mM $Ca(NO_3)_2$). After fractionation was complete, the fractions having high absorbance at 280 nm, determined from the AKTA chromatogram, were pooled. The absorbances of these combined fractions at 280 nm were determined using a storage buffer-blanked Nanodrop One C. The protein solution was then diluted with storage buffer to a concentration of 10 µM as calculated via the Beer-Lambert law using the Expasy Protparam extinction coefficient.[2] The diluted enzyme was then aliquoted, flash frozen using liquid nitrogen, and stored at -80 °C until further use.

Procedure for the Preparation of Wet Lysate Expressing *Ci*VCPO: A 3-L culture of *Ci*VCPO BL21(DE3) cells in Turbo Broth™ treated with 50 mg/L of kanamycin antibiotic was prepared using the previously described procedure. These cells were pelleted using centrifugation at 3.5 krpm for 20 minutes at 10 °C in a Sorvall ST Plus centrifuge, resuspended in buffer (25 mM pH 6 Citrate/H_2SO_4 buffer, 2.5 mM $Ca(NO_3)_2$, 25 mM Na_2SO_4) adjusted to an of OD_{600} = 18.5. Cells were lysed using a Branson SFX250 Sonifier with a ½ inch probe on ice in 15 s pulses at 32% amplitude, 30 W in 10 bursts with 55 s between bursts. The lysed cell solution was clarified using centrifugation at 13.0 krpm for 20 minutes at 10 °C to remove cell debris and subsequently transferred into a separate 50 mL conical tube. The 500 µL of clarified cell lysate solution was then aliquoted into 2 mL microcentrifuge tubes that were then flash frozen using liquid nitrogen and stored at -80 °C until further use. The measured A_{280} value of each aliquot was 236.

Procedure for the Preparation of Whole Cells Expressing *Ci*VCPO: A 3L culture of *Ci*VCPO BL21(DE3) cells in Turbo Broth™ treated with 50 mg/L of kanamycin antibiotic was prepared using the previously described procedure. These cells were pelleted using centrifugation at 3.5 krpm for 20 minutes at 10 °C in a Sorvall ST Plus centrifuge, resuspended in buffer (25 mM pH 6 Citrate/H_2SO_4 buffer, 2.5 mM $Ca(NO_3)_2$, 25 mM Na_2SO_4) adjusted to the OD_{600} = 18.5. The 500 µL of whole cell solution was then aliquoted into 2 mL microcentrifuge tubes and were then flash frozen using liquid nitrogen and stored at -80 °C until further use.

Gel Electrophoresis of VHPO Enzymes

Sodium dodecyl sulfate-polyacrylamide gel electrophoresis (SDS-PAGE) was carried out for VHPO of interest from the Ni-NTA chromatography purification fractions, in addition to samples of diluted lysed cell pellet, lysed supernatant, and Ni-NTA column flow-through. The lysed cell pellet was prepared by mixing a portion of centrifuged pellet with 200 µL of BugBuster® Protein Extraction Reagent, vortexing the mixture, centrifuging for 1 minute, and adding 5 µL of the mixture to a clean microcentrifuge tube. The remaining samples were prepared by adding 5 µL of the lysed supernatant, column flowthrough, or pooled fractions into separate microcentrifuge tubes. To each tube was added 5 µL of 4X Laemmli Buffer and 10 µL of 2-mercaptoethanol. These samples were then shaken in an IKA Matrix orbital shaker at 95 ºC and 300 rpm for 15 minutes. Gel electrophoresis was performed on these samples using 10% sodium dodecyl sulfate Mini-PROTEAN TGX Stain-Free hand casted gels (Bio-Rad Laboratories). A sample gel of *Ci*VCPO is shown in Figure S1 and a sample gel of *Cp*VBPO is shown in Figure S2.

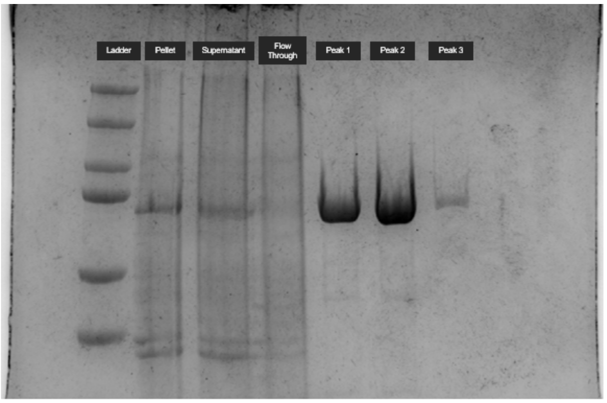

Figure S1. Example Gel Image of *Ci*VCPO

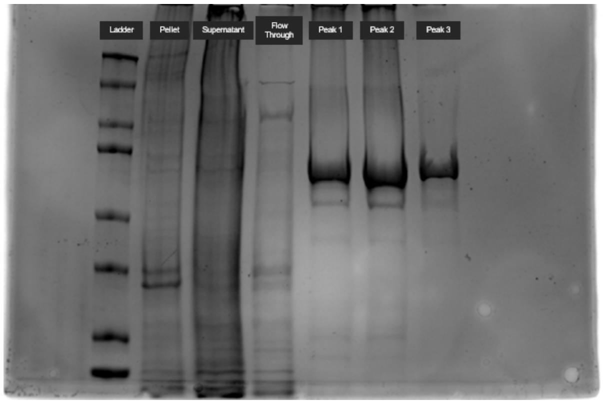

Figure S2. Example Gel Image of *Cp*VBPO

Preparation of Whole Cells Absent of *Ci*VCPO for Control Experiments

Preparation of Whole Cells Absent of *Ci*VCPO: Sample whole cells absent of *Ci*VCPO were prepared using the pUK21 cloning vector, which confers kanamycin resistance to the host cells but possesses an empty backbone so as to not encode for an enzymatic sequence. The pUK21 vector was a gift from Joachim Messing (Addgene plasmid # 49788 ; http://n2t.net/addgene:49788 ; RRID:Addgene_49788).[3] The pUK21 vector in *E. coli* DH5α bacterial cells was plated onto an LB agar plate containing 50 mg/L of kanamycin antibiotic and grown overnight (16 hours) at 37 °C. A single colony was then picked and used to grow a primary culture (9 mL) in Turbo Broth™ using Milli-Q sterile H_2O and containing 50 mg/L of kanamycin antibiotic in a sterile culture tube overnight (18 hours) at 32 °C and 200 rpm in an Infors HT Minitron incubator shaker. The pUK21 vector was then isolated via miniprep with a QIAprep Spin Miniprep Kit using the following protocol provided by QIAGEN: [[https://www.qiagen.com/na/resources/download.aspx?id=56b0162c-23b0-473c-9229-12e8b5c8d590&lang=en]] with only one change: in the final elution step, only 35 µL (rather than 60 µL) of elution buffer was added to the spin column, which was then left to stand for 2 minutes instead of 1 minute. The isolated plasmid was transformed into BL21(DE3) cells from New England Biolabs using the high-efficiency heat shock transformation method provided by NEB [[https://www.neb.com/en-us/protocols/0001/01/01/transformation-protocol-for-bl21-de3-competent-cells-c2527?srsltid=AfmBOops3k4vBIPbasy5Aej7TycYZLtYkPZ4T7RWJ-fA1rPB8ucpDfnQ]]. A sample of 100 µL of the transformed cells was plated onto an LB agar plate containing 50 mg/L of kanamycin antibiotic and grown overnight (18 hours) at 37 °C. A single colony was then picked and used to grow three primary cultures (6 mL) in Turbo Broth™ using purified H_2O from a Milli-Q purification system and containing 50 mg/L of kanamycin antibiotic in sterile culture tubes overnight (18 hours) at 26 °C and 260 rpm in an Infors HT Minitron incubator shaker. At this stage, 500 µL of the primary culture was used to prepare a 25% glycerol stock (comprising a 1:1 mixture of cell culture and sterile 50% glycerol in H_2O), which was flash-frozen in liquid nitrogen and stored at -80 °C. This cell stock was used to inoculate primary cell cultures in subsequent batches. The rest of the primary cultures were used to inoculate three kanamycin Turbo Broth™ 1 L secondary cultures in three 3L Fernbach flasks (6 mL of primary culture per 1 L of secondary culture, final kanamycin concentration of 50 mg/L) and the flasks were incubated in an Infors HT Multitron incubator shaker at 37 °C and 200 rpm until the cultures reached an optical density (OD_{600}) between 0.8 and 1. After reaching the appropriate optical density, the flasks were placed in an ice bath for 30 minutes before being treated with 100 µL of a 1 M isopropyl β-D-1-thiogalactopyranoside (IPTG) stock [final concentration of 0.1 mM IPTG]. The liter cultures were then allowed to shake overnight (20 hours) at 18 °C, shaken at 200 rpm in an Infors HT Multitron incubator shaker. These three 1-L cell cultures were pelleted using centrifugation at 3.5 krpm at 10 °C for 20 minutes in a Beckman Coulter Avanti JXN-26 centrifuge, and the broth supernatant was discarded. The cell pellets from the three 1-L cultures were then split evenly between two sterile 50 mL conical tubes, which were then centrifuged at 13,000 rpm at 10 °C for 20 minutes in a Thermo Scientific Sorvall ST Plus Series centrifuge to remove any excess cell broth. These two pUK21 pellets (each weighing approximately 14.75 g) were then each resuspended in *Ci*VCPO storage buffer (25 mM pH 6 Citrate/H_2SO_4, 25 mM Na_2SO_4, 2.5 mM $Ca(NO_3)_2$). One resuspended pellet was used to prepare the whole cell aliquots, while the other resuspended pellet was reserved to prepare the clarified lysate aliquots. The pUK21 pellet resuspended in *Ci*VCPO storage buffer was adjusted to an OD_{600} value of 19.7. This whole cell

solution was then separated into 500 µL aliquots contained in 2 mL microcentrifuge tubes, flash frozen using liquid nitrogen, and stored at -80 °C until further use.

Quantification of Whole Cells Harboring *Ci*VCPO: Six microcentrifuge tubes were prepared containing the materials outlined in Table S1.

Tube 1	Tube 2	Tube 3	Tube 4	Tube 5	Tube 6
10 µL *Ci*VCPO Whole Cells	5 µL *Ci*VCPO Whole Cells + 5 µL BugBuster®	2.5 µL of 10 µM purified *Ci*VCPO + 7.5 µL storage buffer (25 mM)	5.0 µL of 10 µM purified *Ci*VCPO + 5.0 µL storage buffer (50 mM)	7.5 µL of 10 µM purified *Ci*VCPO + 2.5 µL storage buffer (75 mM)	10.0 µL of 10 µM purified *Ci*VCPO (100 mM)

Table S1. Standard Curve Preparation of *Ci*VCPO Whole Cells

Upon addition of all contents indicated, each tube was vortexed for one minute and centrifuged for one minute. To each tube was added 5 µL of 4X Laemmli Buffer and 10 µL of 2-mercaptoethanol. These samples were then shaken in an IKA Matrix orbital shaker at 95 ºC and 300 rpm for 15 minutes. The SDS-PAGE was carried out on these samples following the protocol outlined in the Gel Electrophoresis of VHPO Enzymes section above. A total of 8 wells were filled including one lane of 4 µL of ladder, one empty lane, and 6 following lanes made up of 10 µL from each tube outlined in Table S1. An example of the gel result can be found in Figure S3. Then, intensity values for each lane were identified using Bio-Rad Image Lab software, where these intensity values are directly correlated to the protein concentration. These values were corrected by multiplying by a dilution factor of 2.5 to take into account that only 10 µL of the total 25 µL enzyme mix were loaded into the well. For tube 2, the value was multiplied by 2 to take into account that only 5 µL of the total 10 µL volume of *Ci*VCPO was added to the lane. Then, all the values were subtracted by intensity of the empty lane. The concentration (mM) vs. intensity graph was plotted using intensity values at 0 (empty lane), 25, 50, 75, and 100 mM. Using these data points, the linear regression was drawn as shown in Figure S4. Taking the corrected intensity value for tube 2 as y-value of the regression line, the concentration value was extrapolated (x-value), which was then used to calculate mol% of *Ci*VCPO whole cells. The average of three trials' mol% was used to calculate the total turnover number (TTN) of the substrates (Table S2).

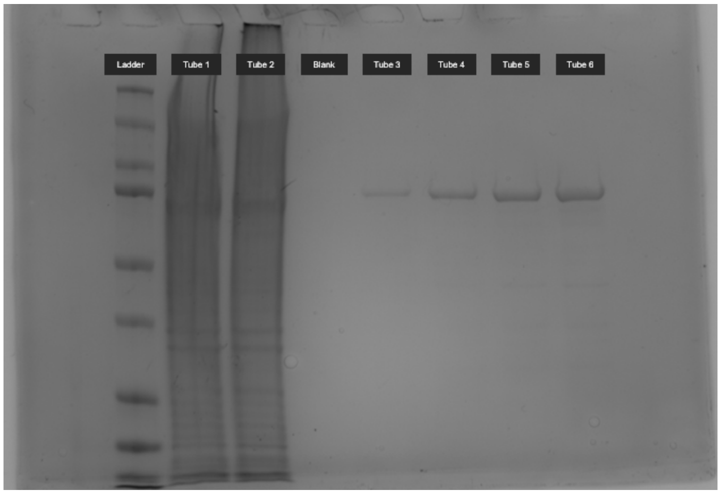

Figure S3. Example Gel Image for the Standard Curve of Whole Cells Harboring *Ci*VCPO

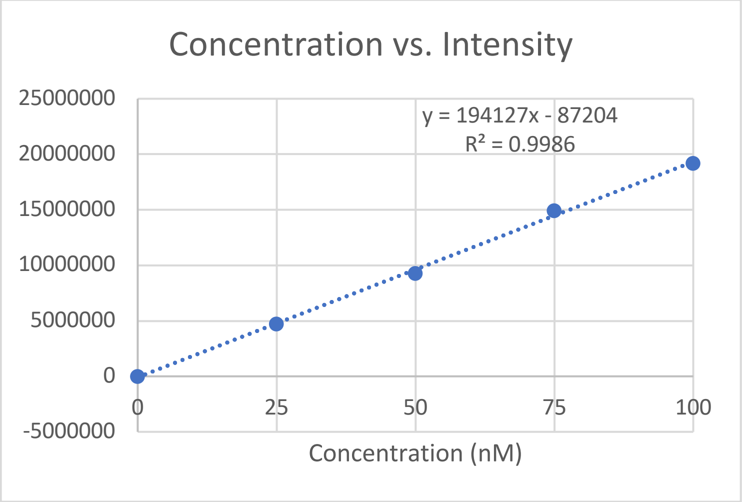

Figure S4. Standard Curve of Whole Cells Harboring *Ci*VCPO

	Concentration (mM)	Mol %
Trial 1	88.2	0.0022
Trial 2	90.5	0.0023
Trial 3	90.1	0.0023
Average	89.6	0.0022

Table S2. Concentration and Mol% Values for Three Trials and Average

Synthesis and Characterization of 3-Substituted Indole Substrates

Synthesis of N-(2-(1H-indol-3-yl)ethyl)acetamide (SM-13)

This synthesis was adapted from Vincent and co-workers[4] with the slight modifications indicated below:

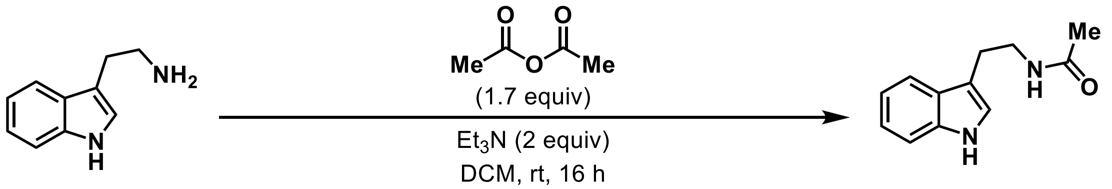

To a stirred suspension of tryptamine (0.50 g, 3.1 mmol) in dry DCM (7.5 mL) in a 50 mL round bottom flask, a solution of acetic anhydride (0.5 mL, 1.7 equiv, 5.3 mmol) and triethylamine (0.87 mL, 2 equiv, 6.2 mmol) in DCM (1.7 mL) was added dropwise. After 16 h stirring at room temperature, the reaction mixture was treated with 10 mL of saturated aqueous $NaHCO_3$ solution. The entire mixture was transferred to a 125 mL separatory funnel with 20 mL of DCM and 20 mL of saturated brine solution. The separated aqueous layer was extracted twice with 20 mL of DCM at a time, and combined organic layer was washed twice with 25 mL of saturated brine solution at a time. Then, the organic layer was dried over Na_2SO_4 for 10 minutes, concentrated under reduced pressure, and purified via flash chromatography to give N-(2-(1H-indol-3-yl)ethyl)acetamide (584 mg, 0.36 mmol) in 93% yield as a white solid.

Purification: Eluted with 5% MeOH in DCM (R_f = 0.30)

^{1}H NMR (400 MHz, $CDCl_3$) δ. 8.30 (br s, 1H), 7.63 (d, J = 7.9Hz, 1H), 7.41 (d, J = 7.9Hz, 1H), 7.24 (m, 1H), 7.16 (m, 1H), 6.97 (s, 1H,), 5.61 (br s, 1H), 3.63 (q, J = 6.5Hz, 2H), 3.00 (t, J = 6.7Hz, 2H), 1.95 (s,3H)

^{13}C NMR (101 MHz, $CDCl_3$) δ. 170.6, 136.4, 127.3, 122.2, 122.0, 119.3, 118.6, 112.6, 111.4, 39.9, 25.2, 23.3.

These NMR spectra were consistent with the literature precedent provided.

Synthesis and Characterization of 2,3-Disubstituted Indole Substrates

Synthesis of 1-(1,3,4,9-tetrahydro-2H-pyrido[3,4-b]indol-2-yl)ethan-1-one (3)

This synthesis was adapted from Ye and co-workers[5] with the slight modifications indicated below:

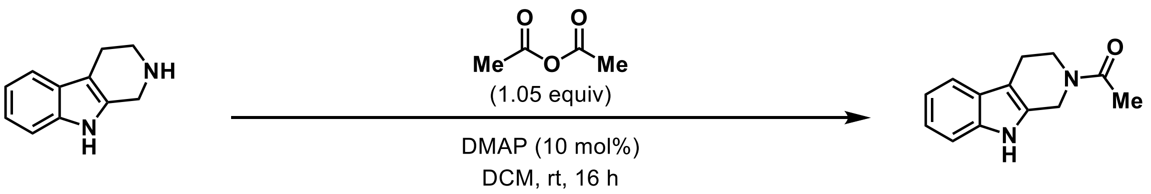

A 250 mL oven-dried round bottom flask was charged with 2,3,4,9-tetrahydro-1H-pyrido[3,4-b]indole (2.00 g, 11.6 mmol), and suspended in DCM (58.1 mL). Acetic anhydride (1.24 g, 1.15 mL, 1.05 equiv, 12.2 mmol) was added dropwise via syringe, and 4-dimethylaminopyridine (DMAP, 0.14 g, 10 mol%, 1.2 mmol) was added. The reaction was stirred for 16 hours and monitored by TLC (5% MeOH in DCM). Upon completion, the reaction was quenched with H_2O and the organic layer was concentrated under reduced pressure. This crude material was resuspended in EtOAc and filtered over a glass-fritted funnel, yielding 1-(1,3,4,9-tetrahydro-2H-pyrido[3,4-b]indol-2-yl)ethan-1-one (1.98 g, 9.24 mmol, 79.6%) as a dry, tan powder.

Purification: Eluted with 5% MeOH in DCM (R_f = 0.5)

^{1}H NMR (400 MHz, DMSO) δ 10.87 (d, J = 4.1 Hz, 1H), 7.40 (d, J = 7.7 Hz, 1H), 7.32 (t, J = 7.1 Hz, 1H), 7.05 (tdd, J = 8.0, 3.2, 1.3 Hz, 1H), 7.01 – 6.93 (m, 1H), 4.67 (d, J = 2.2 Hz, 2H), 3.84 – 3.68 (m, 2H), 2.83 – 2.59 (m, 2H), 2.13 (d, J = 7.9 Hz, 3H).

^{13}C NMR (101 MHz, DMSO) δ 169.06, 168.99, 136.09, 135.91, 131.33, 130.85, 126.56, 126.44, 120.82, 120.72, 118.48, 117.53, 117.47, 111.04, 111.00, 107.25, 106.61, 44.09, 43.61, 21.99, 21.48, 21.40, 20.64.

These NMR spectra were consistent with the literature precedent provided.

Synthesis of tert-butyl 1,3,4,9-tetrahydro-2H-pyrido[3,4-b]indole-2-carboxylate (SM-21)

This synthesis was adapted from Ye and co-workers[5] with the slight modifications indicated below:

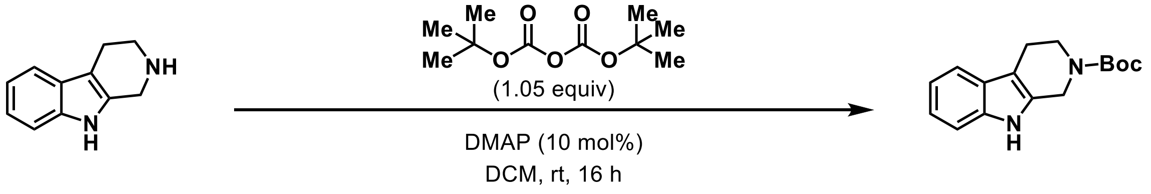

An oven-dried 100 mL round bottom flask was charged with 2,3,4,9-tetrahydro-1H-pyrido[3,4-b]indole (0.86 g 5.0 mmol) and suspended in DCM (25 mL). Boc_2O (1.10 g, 1.05 equiv, 5.3 mmol) was added, followed by DMAP (0.061 g, 10 mol%, 0.50 mmol). The reaction was stirred for 16 hours, and monitored by TLC (50% EtOAc in Hexanes). Upon completion, the reaction was quenched with 25 mL of H_2O and extracted with DCM (3 x 25 mL). The organic layer was separated, concentrated under reduced pressure, and purified via flash column chromatography (0 to 50% EtOAc /Hex gradient) yielding tert-butyl 1,3,4,9-tetrahydro-2H-pyrido[3,4-b]indole-2-carboxylate (923 mg, 3.39 mmol, 68%) as a white solid.

Purification: Eluted with 50% EtOAc in Hexanes (R_f = 0.45)

^{1}H NMR (600 MHz, DMSO) δ 10.84 (d, *J* = 17.8 Hz, 1H), 7.39 (dd, *J* = 7.6, 1.2 Hz, 1H), 7.30 (dt, *J* = 8.1, 0.9 Hz, 1H), 7.04 (ddd, *J* = 8.2, 7.0, 1.2 Hz, 1H), 6.97 (ddd, *J* = 8.0, 7.0, 1.0 Hz, 1H), 4.57 (s, 2H), 3.68 (t, *J* = 5.7 Hz, 2H), 2.69 (tt, *J* = 5.8, 1.6 Hz, 2H), 1.45 (s, 9H).

^{13}C NMR (151 MHz, DMSO) δ 154.43, 154.14, 135.90, 131.35, 131.10, 126.55, 120.75, 118.46, 117.48, 110.98, 106.75, 79.09, 42.29, 41.96, 41.35, 40.91, 28.08, 21.01, 20.73.

These NMR spectra were consistent with the literature precedent provided.

Synthesis of 2,2,2-trichloroethyl 1,3,4,9-tetrahydro-2H-pyrido[3,4-b]indole-2-carboxylate (SM-22)

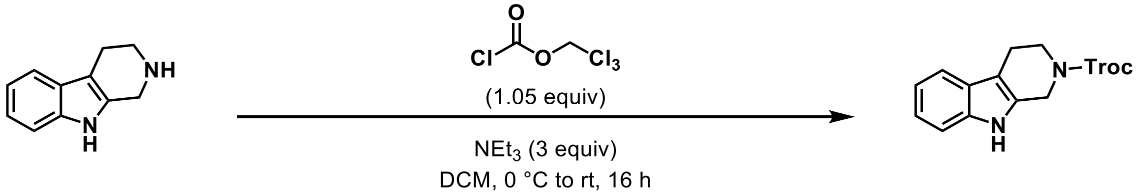

An oven-dried 100 mL round bottom flask was charged with 2,3,4,9-tetrahydro-1H-pyrido[3,4-b]indole (0.86 g, 5.0 mmol) and suspended in DCM (25 mL). The reaction was cooled to 0 °C via ice bath and 2,2,2-Trichloroethoxycarbonylchloride (1.1 g, 0.72 mL, 1.05 equiv, 5.3 mmol) was added dropwise via syringe, followed by triethylamine (1.5 g, 2.1 mL, 3 equiv, 15 mmol). The reaction was stirred for 16 hours, and monitored by TLC (50% EtOAc in Hexanes). Upon completion, the reaction was quenched with 25 mL of H_2O. The organic layer was separated, concentrated under reduced pressure, and purified via flash column chromatography (0 to 50% EtOAc /Hex gradient) yielding 2,2,2-trichloroethyl 1,3,4,9-tetrahydro-2H-pyrido[3,4-b]indole-2-carboxylate (1.450 g, 4.171 mmol, 83%) as a white solid.

Purification: Eluted with 50% EtOAc in Hexanes (R_f = 0.55)

^{1}H NMR (600 MHz, DMSO) δ 10.90 (d, J = 8.4 Hz, 1H), 7.40 (d, J = 7.8 Hz, 1H), 7.31 (t, J = 8.1 Hz, 1H), 7.05 (ddd, J = 8.1, 7.0, 1.2 Hz, 1H), 6.97 (ddd, J = 8.0, 7.0, 1.0 Hz, 1H), 4.92 (d, J = 4.6 Hz, 2H), 4.83 – 4.55 (m, 2H), 3.93 – 3.67 (m, 2H), 2.77 (dd, J = 13.7, 7.2 Hz, 2H).

^{13}C NMR (151 MHz, DMSO) δ 153.40, 153.22, 135.96, 135.91, 130.54, 130.20, 126.48, 126.42, 120.96, 120.92, 118.58, 117.58, 111.09, 111.06, 106.76, 106.59, 95.96, 95.93, 74.32, 74.25, 42.32, 42.29, 42.11, 41.84, 21.07, 20.52.

HRMS: calculated for $C_{14}H_{14}N_2O_2Cl_3$ $[M+H^+]^+$: 347.0115. Found $[M+H^+]^+$: 347.0122.

IR: (cm^{-1}) 3391, 3017, 2952, 1702, 1621, 1254, 790.

Synthesis of methyl 1,3,4,9-tetrahydro-2H-pyrido[3,4-b]indole-2-carboxylate (SM-23)

This synthesis was adapted from Li and co-workers[6] with the slight modifications indicated below:

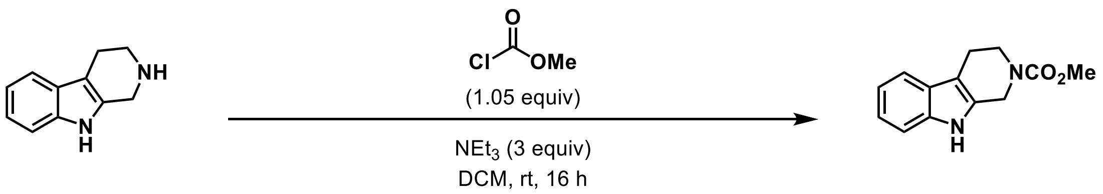

An oven-dried 100 mL round bottom flask was charged with 2,3,4,9-tetrahydro-1H-pyrido[3,4-b]indole (0.86 g, 5.0 mmol) and suspended in DCM (25 mL). The reaction was cooled to 0 °C via ice bath and methyl chloroformate (0.50 g, 1.05 equiv, 5.3 mmol) was added dropwise via syringe, followed by triethylamine (1.50 g, 2.1 mL, 3 equiv, 15 mmol). The reaction was stirred for 16 hours, and monitored by TLC (50% EtOAc in Hexanes). Upon completion, the reaction was quenched with 25 mL of H_2O. The organic layer was separated, concentrated under reduced pressure, and purified via flash column chromatography (0 --> 50% EtOAc /Hex gradient) yielding methyl 1,3,4,9-tetrahydro-2H-pyrido[3,4-b]indole-2-carboxylate (1.024 g, 4.447 mmol, 89%) as a tan solid.

Purification: Eluted with 50% EtOAc in Hexanes (R_f = 0.35)

^{1}H NMR (600 MHz, DMSO) δ 10.85 (s, 1H), 7.39 (d, *J* = 7.8 Hz, 1H), 7.32 (dt, *J* = 8.0, 0.9 Hz, 1H), 7.05 (ddd, *J* = 8.1, 7.0, 1.2 Hz, 1H), 6.97 (ddd, *J* = 7.9, 7.0, 1.0 Hz, 1H), 4.61 (s, 2H), 3.72 (t, *J* = 5.7 Hz, 2H), 3.67 (s, 3H), 2.71 (ddt, *J* = 7.0, 5.7, 1.6 Hz, 2H).

^{13}C NMR (151 MHz, DMSO) δ 156.17, 156.03, 136.40, 131.46, 131.27, 127.00, 121.28, 118.97, 117.98, 111.50, 107.10, 53.02, 42.37, 42.21, 21.48, 21.08.

These NMR spectra were consistent with the literature precedent provided.

Synthesis of 1-(9-methyl-1,3,4,9-tetrahydro-2H-pyrido[3,4-b]indol-2-yl)ethan-1-one (SM-24)

This synthesis was adapted from Ye and co-workers[7] with the slight modifications indicated below:

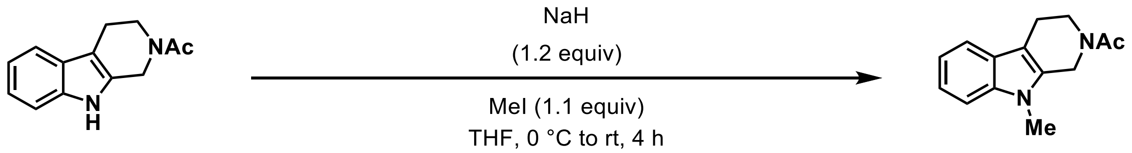

An oven-dried 50 mL round bottom flask was charged with 1-(1,3,4,9-tetrahydro-2H-pyrido[3,4-b]indol-2-yl)ethan-1-one (0.50 g, 2.33 mmol), and suspended in THF (11.7 mL). The flask was cooled to 0°C via ice bath, and sodium hydride (112 mg, 60% Wt, 1.2 equiv, 2.80 mmol) was slowly added (*caution: H_2 gas evolution*). The reaction was stirred at this temperature for 30 min, then methyl iodide (0.364 g, 161 μL, 1.1 equiv, 2.57 mmol) was added dropwise via syringe. The reaction was brought to room temperature and stirred for 4 hours and monitored by TLC (50% EtOAc/Hex). Upon completion, the reaction was carefully quenched with 10 mL of aqueous NH_4Cl and extracted with of EtOAc (3 x 10 mL). The organic layer was separated, concentrated under reduced pressure, and purified via flash column chromatography yielding 1-(9-methyl-1,3,4,9-tetrahydro-2H-pyrido[3,4-b]indol-2-yl)ethan-1-one (419 mg, 1.84 mmol, 78.7%) as a white solid.

Purification: Eluted with 30% EtOAc in Hexanes (R_f = 0.35)

^{1}H NMR (600 MHz, DMSO) δ 7.45 – 7.37 (m, 2H), 7.11 (dddd, *J* = 8.2, 7.0, 4.3, 1.2 Hz, 1H), 7.01 (ddd, *J* = 7.9, 7.0, 1.0 Hz, 1H), 4.73 (dt, *J* = 14.5, 1.6 Hz, 2H), 3.85 – 3.71 (m, 2H), 3.65 (d, *J* = 14.0 Hz, 3H), 2.91 – 2.58 (m, 2H), 2.15 (s, 0H).

^{13}C NMR (101 MHz, DMSO) δ 169.21, 169.08, 136.81, 136.68, 132.43, 132.13, 126.03, 125.92, 120.76, 120.69, 118.62, 117.65, 117.61, 109.19, 109.17, 106.92, 106.39, 43.96, 42.80, 29.20, 29.16, 22.05, 21.47, 21.45, 20.62.

These NMR spectra were consistent with the literature precedent provided.

Synthesis of dimethyl (S)-1,3,4,9-tetrahydro-2H-pyrido[3,4-b]indole-2,3-dicarboxylate (SM-25)

This synthesis was adapted from Ding and co-workers[8] with the slight modifications indicated below:

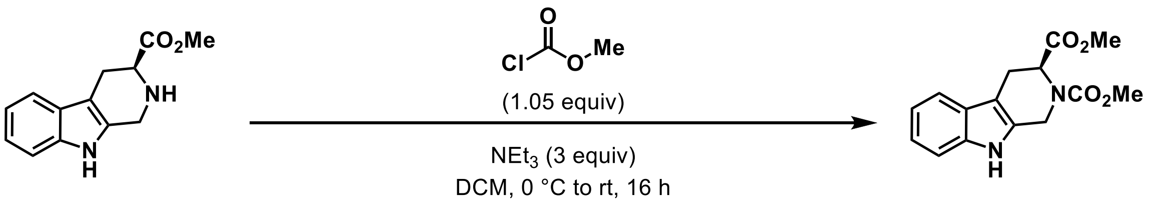

An oven dried 20 mL scintillation vial was charged with methyl (S)-2,3,4,9-tetrahydro-1H-pyrido[3,4-b]indole-3-carboxylate (0.50 g, 2.17 mmol) and dissolved in DCM (10.9 mL). The vial was cooled to 0°C, then methyl chloroformate (0.215 g, 1.05 equiv, 2.28 mmol) was added dropwise via syringe followed by triethylamine (0.659 g, 908 µL, 3 equiv, 6.51 mmol). The reaction was brough to room temperature, and stirred for 16 hours and monitored by TLC (5% MeOH in DCM). Upon completion, the reaction was quenched with 5 mL of H_2O and extracted with DCM (3 x 10 mL). The combined organic layers were washed with 25 mL of saturated brine, dried over Na_2SO_4, and concentrated under reduced pressure. The crude material was purified via flash column chromatography to yield dimethyl (S)-1,3,4,9-tetrahydro-2H-pyrido[3,4-b]indole-2,3-dicarboxylate (522 mg, 1.81 mmol, 83.4%).

Purification: Eluted with 1% MeOH in DCM (R_f = 0.55)

^{1}H NMR (600 MHz, DMSO) δ 10.90 (d, J = 14.2 Hz, 1H), 7.44 (d, J = 7.7 Hz, 1H), 7.31 (dd, J = 8.1, 1.0 Hz, 1H), 7.06 (ddd, J = 8.2, 7.0, 1.2 Hz, 1H), 6.98 (ddd, J = 8.0, 7.0, 1.0 Hz, 1H), 5.38 – 5.13 (m, 1H), 4.81 (dd, J = 16.2, 1.7 Hz, 1H), 4.56 – 4.24 (m, 1H), 3.84 – 3.66 (m, 3H), 3.58 (d, J = 1.6 Hz, 3H), 3.35 – 3.29 (m, 2H), 3.09 – 2.97 (m, 1H).

^{13}C NMR (151 MHz, DMSO) δ 172.00, 171.95, 156.88, 156.40, 136.68, 136.59, 130.17, 130.04, 126.63, 121.59, 121.56, 119.11, 118.14, 111.57, 104.63, 104.41, 53.54, 53.46, 53.28, 52.94, 52.86, 31.74, 28.85, 23.51, 23.18, 22.58, 14.43.

These NMR spectra were consistent with the literature precedent provided.

Synthesis of 2-(1H-indol-3-yl)ethan-1-ol (SM-A-26)

This synthesis was adapted from Bettoni and co-workers[9] with the slight modifications indicated below:

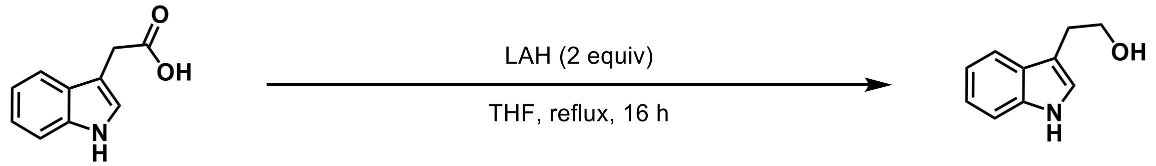

An oven-dried 250 mL round bottom flask was charged with 2-(1H-indol-3-yl)acetic acid (3.00 g, 1 equiv, 17.1 mmol) and dissolved in THF (85.6 mL). LAH (1.30 g, 2 equiv, 34.2 mmol) pellets were added at 0°C, then the reaction was slowly heated to reflux and stirred vigorously for 8 hours. The reaction was monitored via TLC (75% EtOAc/Hex), and when completed the reaction was brough to room temperature and *slowly* quenched with a few drops of H_2O followed by 30 mL of 1M NaOH. 50 mL of a saturated sodium tartrate solution was added, and the reaction vigorously stirred for 1-2 hours until the mixture readily separated. The product was extracted with EtOAc (3 x 50 mL), and the combined organic layer was washed with saturated brine, dried over Na_2SO_4, and concentrated under reduced pressure. This material was typically used without further purificaiton to yield 2-(1H-indol-3-yl)ethan-1-ol (2.32 g, 14.4 mmol, 84.0%) as a dark-brown solid. *Note: trace unreacted starting material can be removed via resuspension in 1:1 Hex / Et_2O and filtration, if needed.*

^{1}H NMR (600 MHz, DMSO) δ 10.77 (s, 1H), 7.50 (ddt, *J* = 7.9, 1.3, 0.7 Hz, 1H), 7.32 (dt, *J* = 8.1, 0.9 Hz, 1H), 7.12 (dd, *J* = 2.2, 1.1 Hz, 1H), 7.05 (ddd, *J* = 8.1, 6.9, 1.2 Hz, 1H), 6.96 (ddd, *J* = 8.0, 6.9, 1.0 Hz, 1H), 4.60 (t, *J* = 5.3 Hz, 1H), 3.64 (ddd, *J* = 7.7, 7.1, 5.3 Hz, 2H), 2.84 (ddd, *J* = 8.0, 7.1, 0.9 Hz, 2H).

^{13}C NMR (151 MHz, DMSO) δ 136.61, 127.89, 123.22, 121.22, 118.80, 118.57, 112.01, 111.74, 62.18, 29.32.

These NMR spectra were consistent with the literature precedent provided.

Synthesis of 1,1-dimethyl-1,3,4,9-tetrahydropyrano[3,4-b]indole (SM-B-26)

This synthesis was adapted from Zhou and co-workers[10] with the slight modifications indicated below:

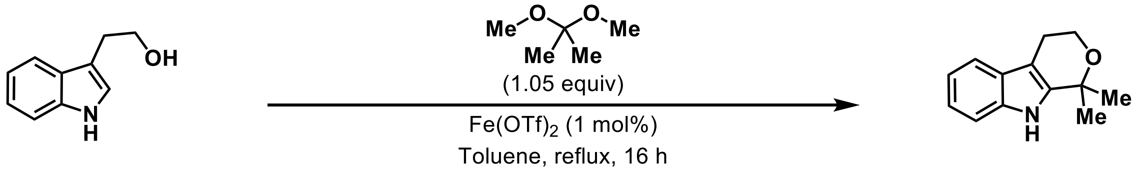

A 20 mL scintillation vial was charged with 2-(1H-indol-3-yl)ethan-1-ol (0.30 g, 1.86 mmol) and dissolved in toluene (9.31 mL) (significant sonication necessary to fully dissolve this material). 2,2-dimethoxypropane (0.204 g mg, 240 µL, 1.05 equiv, 1.95 mmol) was added via syringe, followed by Iron (II) bis(trifluoromethanesulfonate) (6.59 mg, 0.01 equiv, 18.6 µmol). The reaction was capped and placed into a pre-heated 80°C alumnium heating block and stirred vigorously for 4 hours. Upon completion, the reaction was quenched with H_2O (5 mL), and extracted with EtOAc (5 x 10 mL). The combined organic layers were washed with saturated brine, dried over Na_2SO_4, concentrated under reduced pressure and purified via flash column chromatography to yield 1,1-dimethyl-1,3,4,9-tetrahydropyrano[3,4-b]indole (345 mg, 1.71 mmol, 92.1%) as a white solid.

Purification: Eluted with 25% EtOAc in Hexanes (R_f = 0.45)

^{1}H NMR (600 MHz, DMSO) δ 10.83 (s, 1H), 7.39 (ddt, *J* = 7.8, 1.3, 0.7 Hz, 1H), 7.30 (dt, *J* = 8.1, 0.9 Hz, 1H), 7.04 (ddd, *J* = 8.1, 7.0, 1.2 Hz, 1H), 6.96 (ddd, *J* = 7.9, 7.0, 1.0 Hz, 1H), 3.91 (t, *J* = 5.4 Hz, 2H), 2.66 (t, *J* = 5.4 Hz, 2H), 1.49 (s, 6H).

^{13}C NMR (151 MHz, DMSO) δ 140.14, 136.09, 126.90, 121.09, 118.83, 118.20, 111.43, 104.92, 72.05, 60.00, 28.07, 22.61.

These NMR spectra were consistent with the literature precedent provided.

Synthesis of 4',9'-dihydro-3'H-spiro[cyclohexane-1,1'-pyrano[3,4-b]indole] (SM-27)

This synthesis was adapted from Zhou and co-workers[10] with the slight modifications indicated below:

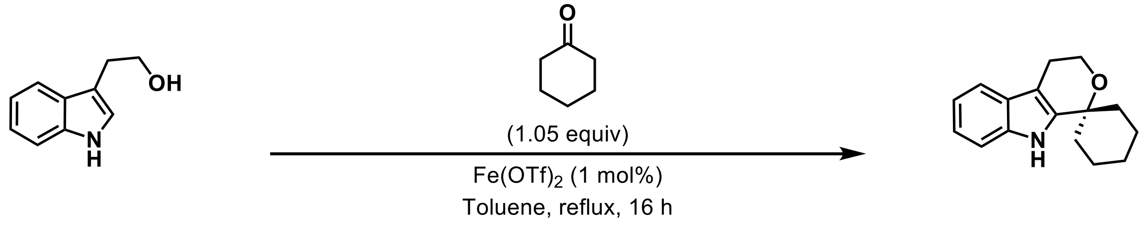

A 20 mL scintillation vial was charged with 2-(1H-indol-3-yl)ethan-1-ol (0.30 g, 1.86 mmol) and dissolved in toluene (9.31 mL) (significant sonication necessary to fully dissolve this material). Cyclohexanone (0.192 g, 1.05 equiv, 1.95 mmol) was added via syringe, followed by Iron (II) bis(trifluoromethanesulfonate) (6.59 mg, 0.01 equiv, 18.6 μmol). The reaction was capped and placed into a pre-heated 80°C alumnium heating block and stirred vigorously for 4 hours. Upon completion, the reaction was quenched with 5 mL of H_2O, and extracted with EtOAc (5 x10 mL). The combined organic layers were washed with saturated brine, dried over Na_2SO_4, concentrated under reduced pressure and purified via flash column chromatography to yield 4',9'-dihydro-3'H-spiro[cyclohexane-1,1'-pyrano[3,4-b]indole] (402 mg, 1.67 mmol, 89.5%) as a white solid.

Purification: Eluted with 25% EtOAc in Hexanes (R_f = 0.55)

^{1}H NMR (600 MHz, DMSO) δ 10.80 (s, 1H), 7.37 (ddt, *J* = 7.8, 1.3, 0.7 Hz, 1H), 7.29 (dt, *J* = 8.1, 0.9 Hz, 1H), 7.02 (ddd, *J* = 8.1, 7.0, 1.2 Hz, 1H), 6.95 (ddd, *J* = 8.0, 7.0, 1.0 Hz, 1H), 3.88 (t, *J* = 5.4 Hz, 2H), 2.65 (t, *J* = 5.4 Hz, 2H), 1.84 (dq, *J* = 11.9, 2.6 Hz, 2H), 1.79 (td, *J* = 13.2, 4.1 Hz, 2H), 1.73 – 1.60 (m, 3H), 1.55 (dt, *J* = 12.9, 3.3 Hz, 2H), 1.28 (dddd, *J* = 16.9, 13.1, 7.9, 4.0 Hz, 1H).

^{13}C NMR (151 MHz, DMSO) δ 140.14, 135.54, 126.44, 120.52, 118.30, 117.64, 110.99, 104.74, 72.05, 58.89, 34.65, 24.98, 22.18, 21.01.

These NMR spectra were consistent with the literature precedent provided.

Synthesis of tert-butyl 4',9'-dihydro-3'H-spiro[piperidine-4,1'-pyrano[3,4-b]indole]-1-carboxylate (SM-28)

This synthesis was adapted from Zhou and co-workers[10] with the slight modifications indicated below:

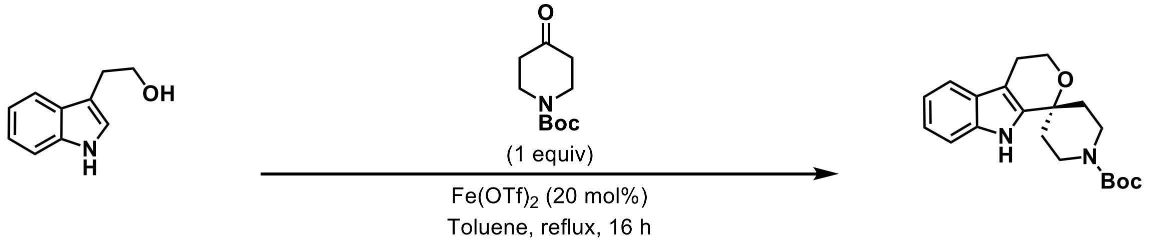

A 20 mL scintillation vial was charged with 2-(1H-indol-3-yl)ethan-1-ol (0.30 g, 1.86 mmol) and dissolved in toluene (9.31 mL) (significant sonication necessary to fully dissolve this material). Tert-butyl 4-oxopiperidine-1-carboxylate (0.371 g, 1 equiv, 1.86 mmol) was added via syringe, followed by Iron (II) bis(trifluoromethanesulfonate) (0.132 g, 0.2 equiv, 372 µmol). The reaction was capped and placed into a pre-heated 80°C alumnium heating block and stirred vigorously for 4 hours. Upon completion, the reaction was quenched with 5 mL of H_2O, and extracted with EtOAc (5 x 10 mL). The combined organic layers were washed with saturated brine, dried over Na_2SO_4, concentrated under reduced pressure and purified via flash column chromatography to yield tert-butyl 4',9'-dihydro-3'H-spiro[piperidine-4,1'-pyrano[3,4-b]indole]-1-carboxylate (0.321 g, 1.33 mmol, 71.5%) as a white solid.

Purification: Eluted with 25% EtOAc in Hexanes (R_f = 0.35)

^{1}H NMR (400 MHz, DMSO) δ 10.89 (s, 1H), 7.44 – 7.37 (m, 1H), 7.29 (dt, J = 8.1, 0.9 Hz, 1H), 7.09 – 6.93 (m, 2H), 3.94 (t, J = 5.4 Hz, 2H), 3.07 (s, 3H), 2.69 (t, J = 5.3 Hz, 2H), 2.02 – 1.88 (m, 2H), 1.81 (d, J = 13.5 Hz, 2H), 1.45 (s, 9H).

^{13}C NMR (101 MHz, DMSO) δ 153.78, 138.36, 135.64, 126.30, 120.81, 118.45, 117.79, 111.07, 105.41, 78.58, 70.60, 59.26, 28.17, 22.08.

HRMS: calculated for $C_{20}H_{26}N_2O_3$ [M+H$^+$]$^+$: 342.1938. Found [M+H$^+$]$^+$: 342.1935

IR (cm^{-1}): 3279, 2957, 1659, 1367, 1149, 1073, 764.

Synthesis of 6-methoxy-2-methyl-2,3,4,9-tetrahydro-1H-pyrido[3,4-b]indole (SM-29)

This synthesis was adapted from Jiang and co-workers[11] with the slight modifications indicated below:

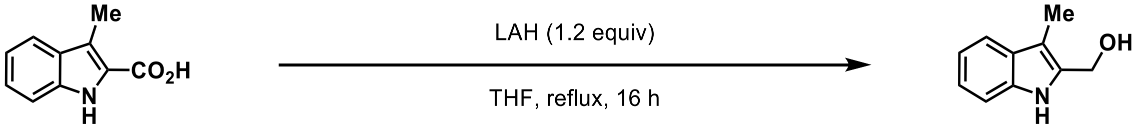

An oven-dried 250 mL round bottom flask was charged with 3-methyl-1H-indole-2-carboxylic acid (1.5 g, 8.562 mmol) and dissolved in THF (42.81 mL). LAH (0.390 g, 1.2 equiv, 10.27 mmol) pellets were added at 0°C, then the reaction was slowly brought to reflux and stirred for 8 hours. The reaction was monitored by TLC (5% MeOH in DCM), and upon completion the reaction was brought to room temperature and *slowly* quenched with a few drops of H_2O. The reaction was basified with of 1M NaOH (20 mL) followed by of saturated sodium tartrate solution (40 mL). EtOAc (40 mL) was added and the reaction was vigorously stirred until the mixture readily separated. The product was extracted with 3 x 40 mL of EtOAc, washed with saturated brine, dried over Na_2SO_4, and concentrated under reduced pressure. This crude material was purified via flash column chromatography using a 0 to 5% MeOH gradient yielding (3-methyl-1H-indol-2-yl)methanol (1.025 g, 6.359 mmol, 74.26%) as an orange-tan solid.

Purification: Eluted with 5% MeOH in DCM (R_f = 0.6)

^{1}H NMR (600 MHz, DMSO) δ 10.74 (s, 1H), 7.42 (ddt, *J* = 7.8, 1.3, 0.8 Hz, 1H), 7.29 (dt, *J* = 8.0, 0.9 Hz, 1H), 7.03 (ddd, *J* = 8.1, 7.0, 1.2 Hz, 1H), 6.95 (ddd, *J* = 7.9, 7.0, 1.0 Hz, 1H), 5.07 (t, *J* = 5.4 Hz, 1H), 4.59 (d, *J* = 5.3 Hz, 2H), 2.22 (s, 3H).

^{13}C NMR (151 MHz, DMSO) δ 135.82, 135.68, 128.94, 121.15, 118.48, 118.45, 111.34, 106.11, 55.12, 8.75.

These NMR spectra were consistent with the literature precedent provided.

Synthesis of methyl (S)-2,3,4,9-tetrahydro-1H-pyrido[3,4-b]indole-3-carboxylate (36)

This synthesis was adapted from Ye and co-workers[5] with the slight modifications indicated below:

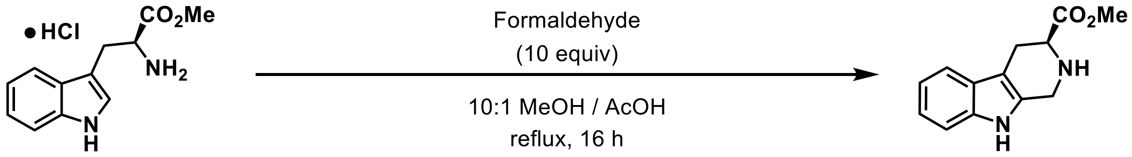

A 250 mL round bottom flask was charged with methyl L-tryptophanate hydrochloride (10 g, 39.26 mmol), and dissolved in a 10:1 mixture of MeOH (71.38 mL) and AcOH (7.138 mL). An aqueous solution of formaldehyde (31.86 g, 29.23 mL, 37% Wt, 10 equiv, 392.6 mmol) was then added over a 10 minute period via addition funnel. The addition funnel was removed and replaced with a reflux condenser, and the reaction was heated to reflux for 16 hours. The reaction was then brought to room temperature, slowly quenched with saturated sodium bicarbonate until pH = 9, and extracted with EtOAc (3 x 50 mL). The combined organic layer was washed with saturated brine, concentrated under reduced pressure, and purified via flash column chromatography to yield methyl (S)-2,3,4,9-tetrahydro-1H-pyrido[3,4-b]indole-3-carboxylate (3.043 g, 13.21 mmol, 33.66%) as a crystaline white solid.

Purification: Eluted with 5% MeOH in DCM (R_f = 0.35)

^{1}H NMR (600 MHz, DMSO) δ 10.72 (s, 1H), 7.37 (ddt, *J* = 7.9, 1.4, 0.7 Hz, 1H), 7.27 (dt, *J* = 8.1, 0.9 Hz, 1H), 7.01 (ddd, *J* = 8.1, 7.0, 1.2 Hz, 1H), 6.94 (ddd, *J* = 8.0, 7.0, 1.0 Hz, 1H), 4.00 (dt, *J* = 15.9, 1.2 Hz, 1H), 3.92 (dt, *J* = 15.9, 1.9 Hz, 1H), 3.73 (dd, *J* = 8.8, 4.8 Hz, 1H), 3.68 (s, 3H), 2.93 (dddd, *J* = 14.9, 4.9, 1.9, 0.9 Hz, 1H), 2.81 – 2.71 (m, 2H).

^{13}C NMR (151 MHz, DMSO) δ 174.11, 136.20, 133.95, 127.39, 120.89, 118.74, 117.67, 111.31, 105.96, 55.67, 52.09, 41.87, 25.47.

These NMR spectra were consistent with the literature precedent provided.

2-methyl-2,3,4,9-tetrahydro-1H-pyrido[3,4-b]indole (43)

This synthesis was adapted from Sathish and co-workers[12] with the slight modifications indicated below:

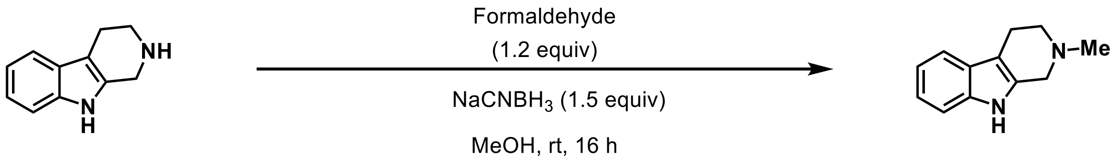

A 100 mL round bottom flask was charged with 2,3,4,9-tetrahydro-1H-pyrido[3,4-b]indole (1.00 g, 11.6 mmol) and dissolved in MeOH (58.1 mL). A 37% solution of formaldehyde (1.13 g, 1.04 mL, 37% Wt, 1.2 equiv, 13.9 mmol) was added, and the reaction was stirred for 30 minutes. Sodium cyanoborohydride (1.09 g, 1.5 equiv, 17.4 mmol) was then added in one portion, and left to stir for 2 hours at room temp and monitored by TLC (5% MeOH in DCM). Upon completion, the reaction was basified to pH 9.0 with saturated sodium bicarbonate, and extracted with 5 x 25 mL of EtOAc (*Note: this product is very water soluble and the recovered mass balance should be noted before discarding any aqueous portions*). The combined organic layer was washed with saturated brine, dried over Na_2SO_4, and concentrated under reduced pressure. This crude material was purified via flash column chromatography (0 to 2%MeOH) to yield 2-methyl-2,3,4,9-tetrahydro-1H-pyrido[3,4-b]indole (1.543 g, 8.284 mmol, 71.3%) as an off-white dense solid.

Purification: Eluted with the gradient of 0-2 % MeOH in DCM (R_f = 0.25)

^{1}H NMR (400 MHz, DMSO) δ 7.89 (br s, 1H), 7.47 (m, 1H), 7.29 (m, 1H), 7.11(dtd, J = 18.1, J = 7.2, J = 1.3 Hz, 2H), 3.59(s, 2H), 2.83(qd, J = 6.3, 2.8 Hz, 4H), 2.50 (s, 3H).

^{13}C NMR (101 MHz, DMSO) δ 136.00, 131.70, 127.18, 121.30, 119.29, 117.95, 52.98, 52.31, 45.68, 21.44

These NMR spectra were consistent with the literature precedent provided.

Synthesis of 6-methoxy-2-methyl-2,3,4,9-tetrahydro-1H-pyrido[3,4-b]indole (45)

This synthesis was adapted from Sathish and co-workers[12] with the slight modifications indicated below:

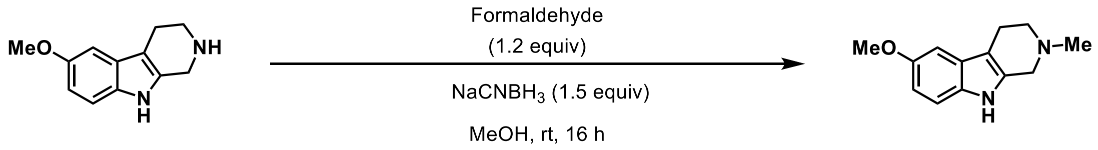

A 100ml round bottom flask was charged with 6-methoxy-2,3,4,9-tetrahydro-1H-pyrido[3,4-b]indole (2.00 g, 9.89 mmol) and dissolved in MeOH (49.4 mL). A 37% solution of formaldehyde (0.356 g, 327 µL, 1.2 equiv, 11.9 mmol) was added, and the reaction was stirred for 30 minutes. Sodium cyanoborohydride (0.932 g, 1.5 equiv, 14.8 mmol) was then added in one portion, and left to stir for 2 hours at room temp and monitored by TLC (DCM + 1% NEt3 w/ basified normal phase TLC plates). Upon completion, the reaction was basified to pH 9.0 with saturated sodium bicarbonate, and extracted 5 x 25 mL of EtOAc (*Note: This product is very water soluble and the recovered mass balance should be noted before discarding any aqueous portions*). The combined organic layer was washed with saturated brine, dried over Na_2SO_4, and concentrated under reduced pressure. This crude material was purified via flash column chromatography (0 to 2%MeOH / DCM+1%NEt3) to yield 6-methoxy-2-methyl-2,3,4,9-tetrahydro-1H-pyrido[3,4-b]indole (1.56 g, 7.21 mmol, 72.9 %) as an off-white dense solid.

Purification: Eluted with 2% MeOH in DCM (+1% NEt_3) (R_f = 0.45)

^{1}H NMR (400 MHz, DMSO) δ 10.53 (s, 1H), 7.15 (dd, J = 8.7, 0.5 Hz, 1H), 6.85 (d, J = 2.5 Hz, 1H), 6.64 (dd, J = 8.7, 2.5 Hz, 1H), 3.73 (s, 3H), 3.51 (s, 2H), 2.67 (d, J = 2.8 Hz, 4H), 2.40 (s, 3H).

^{13}C NMR (101 MHz, DMSO) δ 153.46, 134.01, 131.38, 127.48, 111.89, 110.31, 106.39, 100.22, 55.78, 53.13, 52.60, 45.89, 21.78.

These NMR spectra were consistent with the literature precedent provided.

General Analytical Procedures for Oxidation of 3-Monosubstituted Indoles

General Analytical Procedure for Oxidation of 3-Monosubstituted Indoles with Purified *Ci*VCPO (General Procedure A):

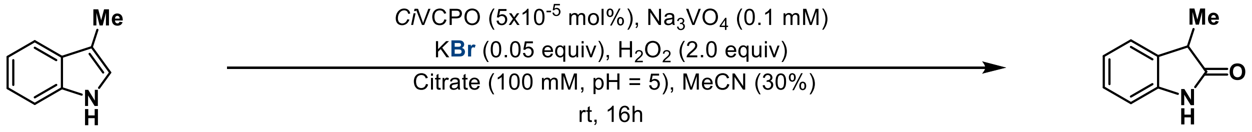

1 2

An enzyme aliquot of purified VHPO from *Curvularia inaequalis* (*Ci*VCPO, 10 µM, 50 µL) was removed from a -80 °C freezer and allowed to warm to room temperature over 5 min. After thawing, it was transferred to a microcentrifuge tube and diluted with *Ci*VCPO storage buffer (25 mM pH 6 Citrate/H_2SO_4 buffer, 25 mM Na_2SO_4, 2.5 mM $Ca(NO_3)_2$) (1.45 mL). The resulting 1.5 mL mixture was centrifuged for 30 seconds using a Chemglass Life Sciences MLX-108-CLS mini centrifuge to ensure complete mixing. After centrifugation, a portion of this solution (6 µL per 0.004 mmol starting material) was pipetted into a fresh PCR tube in addition to an aqueous solution of 50 mM Na_3VO_4 (2 µL, 0.025 equiv relative to starting material). To ensure mixing, the resulting solution was spun again in the mini centrifuge for 10 seconds and placed in the benchtop until further use. A 1-dram vial was then charged with purified H_2O (487 µL), 500 mM pH 5 citrate buffer (200 µL), 100 mM KBr (2 µL, 0.05 equiv) followed by the addition of MeCN (200 µL). A 40 mM solution of the starting 3-monosubstituted indole in a MeCN (100 µL, 1 equiv, 0.004 mmol starting material in reaction) was then added. The aliquot containing the diluted *Ci*VCPO (0.00005 mol%, 2 nM) and Na_3VO_4 (0.025 equiv) was added to the reaction mixture. Finally, a 10% stock of H_2O_2 (2.64 µL, 2 equiv) was added to the reaction mixture. The vial was capped and placed on a Heidolph Multireax Shaker set to 820 rpm at room temperature for overnight. After this time, the reaction mixture was diluted with MeCN (650 µL), transferred to a microcentrifuge tube, and spun in a Benchmark MC-24™ Touch Centrifuge at 13,000 rpm for 5 min. After centrifugation, 600 µL of the top layer of the reaction mixture was transferred to an LCMS vial then placed on an LCMS for analysis (*Note: 100 µL of a 8 mg/mL solution of 1,3,5-tribromobenzene was added as an internal standard for yield confirmation, where applicable*).

General Analytical Procedure for Oxidation of 3-Monosubstituted Indoles with *Ci*VCPO Cell Lysate (General Procedure B):

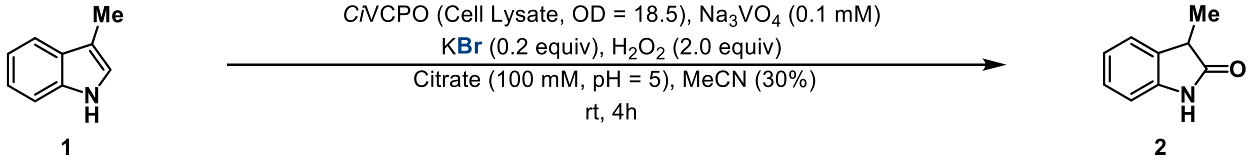

An enzyme aliquot of VHPO cell lysate from *Curvularia inaequalis* (*Ci*VCPO, OD_{600} = 18.5, A_{280} = 236, 500 µL) was removed from a -80 °C freezer and allowed to warm to room temperature over 10 min. A portion of this solution (10 µL per 0.004 mmol starting material) was pipetted into a fresh PCR tube in addition to an aqueous solution of 50 mM Na_3VO_4 (2 µL, 0.025 equiv relative to starting material). To ensure mixing, the resulting solution was spun in the mini centrifuge for 10 seconds and placed on the benchtop until further use. A 1-dram vial was then charged with purified H_2O (477 µL), 500 mM pH 5 citrate buffer (200 µL), 100 mM KBr (8 µL, 0.2 equiv) followed by the addition of MeCN (200 µL). A 40 mM solution of the corresponding 3-monosubstituted indole in a MeCN (100 µL, 1 equiv, 0.004 mmol starting material in reaction) was then added. The aliquot containing *Ci*VCPO and Na_3VO_4 was added to the reaction mixture. Finally, a 10% stock of H_2O_2 (2.64 µL, 2 equiv) was added to the reaction mixture. The vial was capped and placed on a shaker set to 820 rpm at room temperature for 4 h. After this time, the reaction mixture was diluted with MeCN (650 µL), transferred to a microcentrifuge tube, and spun down in a centrifuge at 12,500 rpm for 10 min. After centrifugation, 600µL of the top layer of the reaction mixture was transferred to an LCMS vial then placed on an LCMS for analysis (*Note: 100 µL of a 8 mg/mL solution of 1,3,5-tribromobenzene was added as an internal standard for yield confirmation, where applicable*).

General Analytical Procedure for Oxidation of 3-Monosubstituted Indoles with *Ci*VCPO in Whole Cells (General Procedure C):

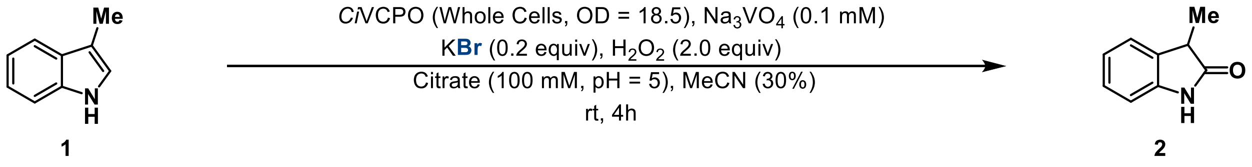

An enzyme aliquot of VHPO whole cell from *Curvularia inaequalis* (*Ci*VCPO, OD_{600} = 18.5, 500 µL) was removed from a -80 °C freezer and allowed to warm to room temperature over 10 min. A portion of this solution (10 µL per 0.004 mmol starting material) was pipetted into a fresh PCR tube in addition to an aqueous solution of 50 mM Na_3VO_4 (2 µL, 0.025 equiv relative to starting material). To ensure mixing, the resulting solution was spun in the mini centrifuge for 10 seconds and placed on the benchtop until further use. A 1-dram vial was then charged with purified H_2O (477 µL), 500 mM pH 5 citrate buffer (200 µL), 100 mM KBr (8 µL, 0.2 equiv) followed by the addition of MeCN (200 µL). A 40 mM solution of the corresponding 3-monosubstituted indole in a MeCN (100 µL, 1 equiv, 0.004 mmol starting material in reaction) was then added. The aliquot containing *Ci*VCPO and Na_3VO_4 was added to the reaction mixture. Finally, a 10% stock of H_2O_2 (2.64 µL, 2 equiv) was added to the reaction mixture. The vial was capped and placed on a shaker set to 820 rpm at room temperature for 4 h. After this time, the reaction mixture was diluted with MeCN (650 µL), transferred to a microcentrifuge tube, and spun down in a centrifuge at 13,000 rpm for 10 min. After centrifugation, 600 µL of the top layer of the reaction mixture was transferred to an LCMS vial then placed on an LCMS for analysis.

General Preparative Procedures for Oxidation of 3-Monosubstituted Indoles

General Preparative Procedure for Oxidation of 3-Monosubstituted Indoles with Purified *Ci*VCPO (General Procedure D): An enzyme aliquot of purified VHPO from *Curvularia inaequalis* (*Ci*VCPO, 10 μM, 20 μL) was removed from a -80 °C freezer and allowed to warm to room temperature over 5 min. The thawed aliquot was then combined with an aqueous solution of 50 mM Na_3VO_4 (200 μL, 0.025 equiv) in a microcentrifuge tube and stored at room temperature. To ensure mixing, the resulting solution was spun in the mini centrifuge for 10 seconds and placed on the benchtop until further use. To a 100 mL round bottom flask containing a magnetic stir bar, purified H_2O (49 mL), 500 mM pH 5 citrate buffer (20 mL), 50 mM KBr (400 μL, 0.05 equiv), and 20 mL MeCN was added. Then, the corresponding 3-monosubstituted indole (0.40 mmol,1 equiv) was dissolved in MeCN (10 mL) and added to the reaction mixture. The microcentrifuge tube containing *Ci*VCPO (0.00005 mol%, 2 nM) and Na_3VO_4 (0.025 equiv) was added to the reaction mixture. A 10% stock of H_2O_2 (264 μL, 2 equiv) was then added to the reaction mixture and the resulting solution was allowed to stir at room temperature for 16h. The reaction mixture was then diluted with EtOAc (30 mL), saturated brine (15 mL) and H_2O (15 mL) (additional EtOAc and H_2O used to rinse flask as needed) and transferred to a 250 mL separatory funnel. The aqueous layer was extracted with EtOAc (2 x 30 mL) and the combined organic layers were then washed with saturated brine (2 x 30 mL) and dried over sodium sulfate (Na_2SO_4) for 10 minutes. The dried crude organic layer was concentrated under reduced pressure, and purified via flash chromatography.

General Preparative Procedure for Oxidation of 3-Monosubstituted Indoles with *Ci*VCPO Cell Lysate (General Procedure E): Two enzyme aliquots of VHPO cell lysate from *Curvularia inaequalis* (*Ci*VCPO, A_{280} = 236, 500 μL) were removed from a -80 °C freezer and allowed to warm to room temperature over 10 min. The thawed aliquots were then combined with an aqueous solution of 50 mM Na_3VO_4 (200 μL, 0.025 equiv relative to starting material) in a microcentrifuge tube and stored at room temperature. To ensure mixing, the resulting solution was spun in a mini centrifuge for 10 seconds and placed in the benchtop until further use. To a 100 mL round bottom flask containing a magnetic stir bar, purified H_2O (48 mL), 500 mM pH 5 citrate buffer (20 mL), 100 mM KBr (800 μL, 0.2 equiv),and 20 mL MeCN was added. Then, the corresponding 3-monosubstituted indole (0.40 mmol,1 equiv) was dissolved in MeCN (10 mL) and added to the reaction mixture. The aliquot containing the *Ci*VCPO and Na_3VO_4 was added to the reaction mixture. A 10% stock of H_2O_2 (264 μL, 2 equiv) was then added to the reaction mixture and the reaction was allowed to stir at room temperature for 4 h. After this time, the reaction mixture was divided equally into four 50 mL falcon tubes and mixed with 25 mL of EtOAc. The organic and aqueous layers were then separated via centrifugation using Thermo Scientific Sorvall ST Plus Series centrifuge at 13 krpm at 10 °C for 15 min. After removal of the organic layers, two additional rounds of extraction were performed with 200 mL EtOAc and 50 mL of saturated brine each time. The combined organic layers were dried over Na_2SO_4 for 10 minutes, concentrated under reduced pressure, and dissolved in a minimal amount of EtOAc or DCM and silica. Then, it was concentrated again under reduced pressure to be dry loaded on to flash chromatography to be purified.

General Preparative Procedure for Oxidation of 3-Monosubstituted Indoles with *Ci*VCPO in Whole Cells (General Procedure F): Two enzyme aliquots of VHPO whole cell from *Curvularia inaequalis* (*Ci*VCPO, OD_{600} = 18.5, 500 µL) were removed from a -80 °C freezer and allowed to warm to room temperature over 10 min. The thawed aliquots were then combined with an aqueous solution of 50 mM Na_3VO_4 (200 µL, 0.025 equiv relative to starting material) in a microcentrifuge tube and stored at room temperature. To ensure mixing, the resulting solution was spun in the mini centrifuge for 10 seconds and placed in the benchtop until further use. To a 100 mL round bottom flask containing a magnetic stir bar, purified H_2O (48 mL), 500 mM pH 5 citrate buffer (20 mL), 100 mM KBr (800 µL, 0.2 equiv), and 20 mL MeCN was added. Then, the corresponding 3-monosubstituted indole (0.40 mmol,1 equiv) was dissolved in MeCN (10 mL) and added to the reaction mixture. The aliquot containing *Ci*VCPO and Na_3VO_4 was added to the reaction mixture. A 10% stock of H_2O_2 (264 µL, 2 equiv) was then added to the reaction mixture and the resulting solution was allowed to stir at room temperature for 4 h. After this time, the reaction mixture was divided equally into four 50 mL falcon tubes and mixed with 25 mL of EtOAc. The organic and aqueous layers were then separated via centrifugation using Thermo Scientific Sorvall ST Plus Series centrifuge at 13 krpm at 10 °C for 15 min. After removal of the organic layers, two additional rounds of extraction are performed with 200 mL EtOAc and 50 mL of saturated brine. The combined organic layers were dried over Na_2SO_4 for 10 minutes, concentrated under reduced pressure, and dissolved in a minimal amount of EtOAc or DCM and silica. Then, it was concentrated again under reduced pressure to be dry loaded on to flash chromatography to be purified.

General Analytical Procedure for Spirooxindole Formation

General Analytical Procedure for Spirooxindole Formation with Purified *Cp*VBPO (General Procedure G):

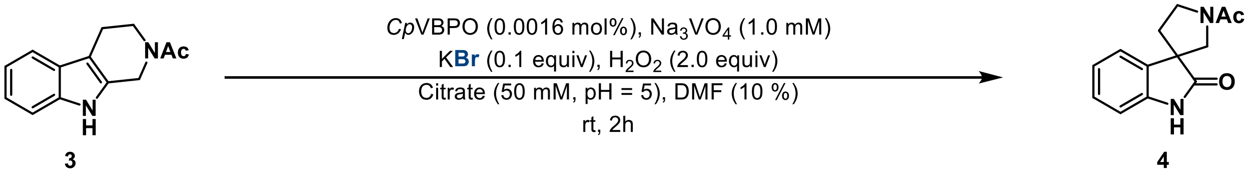

An enzyme aliquot of purified VHPO from *Corallina pilulifera* (*Cp*VBPO, 10 µM, 50 µL) was removed from a -80 °C freezer and allowed to warm to room temperature over 5 min. After thawing, 6.25 µL was transferred to a fresh PCR tube in addition to an aqueous solution of 250 mM Na_3VO_4 (4 µL, 0.25 equiv relative to starting material). To ensure mixing, the resulting solution was spun in the mini centrifuge for 10 seconds and placed in the benchtop until further use. A 1-dram vial was then charged with purified H_2O (783 µL), 500 mM pH 5 citrate buffer (100 µL), and 100 mM KBr (4 µL, 0.1 equiv). A 40 mM solution of the corresponding 2,3-disubstituted indole in a DMF (100 µL, 1 equiv, 0.004 mmol starting material in reaction) was then added. The aliquot containing *Cp*VBPO (0.0016 mol%, 62.5 nM) and Na_3VO_4 (0.25 equiv) was added to the reaction mixture. Finally, a 10% stock of H_2O_2 (2.64 µL, 2 equiv) was added to the reaction mixture. The vial was capped and placed on a Heidolph Multireax Shaker set to 820 rpm at room temperature for 2h. After this time, the reaction mixture was diluted with MeCN (650 µL), transferred to a microcentrifuge tube, and spun in a Benchmark MC-24™ Touch Centrifuge at 13,000 rpm for 5 min. After centrifugation, 600 µL of the top layer of the reaction mixture was transferred to an LCMS vial then placed on an LCMS for analysis (*Note: 100 µL of a 8 mg/mL solution of 1,3,5-tribromobenzene was added as an internal standard for yield confirmation, where applicable*).

General Preparative Procedure for Spirooxdindole Formation

General Preparative Procedure for Spirooxdindole Formation with Purified *Cp*VBPO (General Procedure H): An enzyme aliquot of purified VHPO from *Corallina pilulifera* (*Cp*VBPO, 10 μM, 625 μL) was removed from a -80°C freezer and allowed to warm to room temperature over 5 min. The thawed aliquot was then combined with an aqueous solution of 250 mM Na_3VO_4 (400 μL, 0.25 equiv) in an microcentrifuge tube and stored at room temperature. To ensure mixing, the resulting solution was spun in the mini centrifuge for 10 seconds and placed on the benchtop until further use. To a 100 mL round bottom flask containing a magnetic stir bar, purified H_2O (68 mL), 500mM pH 5 citrate buffer (10 mL) and 100mM KBr (400 μL, 0.1 equiv) was added. The corresponding 2,3-disubstituted indole (0.40 mmol, 1 equiv) was dissolved in DMF (10 mL) and added to the reaction mixture. The microcentrifuge tube containing the *Cp*VHPO (0.0016 mol%, 62.5 nM) and Na_3VO_4 (0.25 equiv) was then added to the reaction mixture. A 10% stock of H_2O_2 (264 μL, 2 equiv) was then added to the reaction mixture and the resulting solution was allowed to stir at room temperature for 2h. The reaction mixture was then diluted with EtOAc (50 mL) and transferred to a 250 mL separatory funnel. The aqueous layer was re-extracted 3-5X with additional EtOAc (50 mL) as needed, and the combined organic layer was washed with saturated brine 3 times to remove DMF. The organic layer was then dried over sodium sulfate (Na_2SO_4) for 10 minutes, concentrated under reduced pressure, and purified via flash chromatography. *(Note: many of the spirooxindole products are water soluble, especially in the presence of DMF. The recovered mass balance of the crude product should be carefully monitored, and residual DMF can be removed via additional saturated brine washes or azeotrope with heptanes)*.

Product Characterization

3-methylindolin-2-one (2)

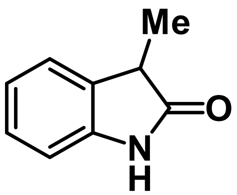

Synthesized from commercially available 3-methyl-1H-indole following General Procedure F

Yield: 97%

Purification: Eluted with the gradient of 10-50 % EtOAc in Hexanes (R_f = 0.38)

^{1}H NMR (400 MHz, $CDCl_3$) δ. 8.94 (br s, 1 H), 7.21 (m, 2 H), 7.03 (m, 1 H), 6.92 (d, J 7.2, 1 H), 3.48 (q, J 7.6, 1 H), 1.51 (d, J 7.6 Hz, 3 H)

^{13}C NMR (101 MHz, $CDCl_3$) δ. 181.6, 141.3, 131.3, 127.9, 123.8, 122.4, 109.8, 41.1, 15.3

These NMR spectra were consistent with the literature precedent[13].

Standard Curve for Analytical Runs:

Procedure for using standard curve is as follows: 1,3,5-bromobenzene (8mg/mL solution, 100 µL) is added to 900 µL of the reaction mixture and yield is determined by LCMS analysis based on the below standard curve. LCMS conditions: 2.5 µL injection volume, 0.5 mL/min mobile phase rate, 10-98% solvent B over 6.25 minutes. Mobile Phase: Solvent A- H_2O w/ 0.1% formic acid, Solvent B- MeCN w/ 0.1% formic acid.

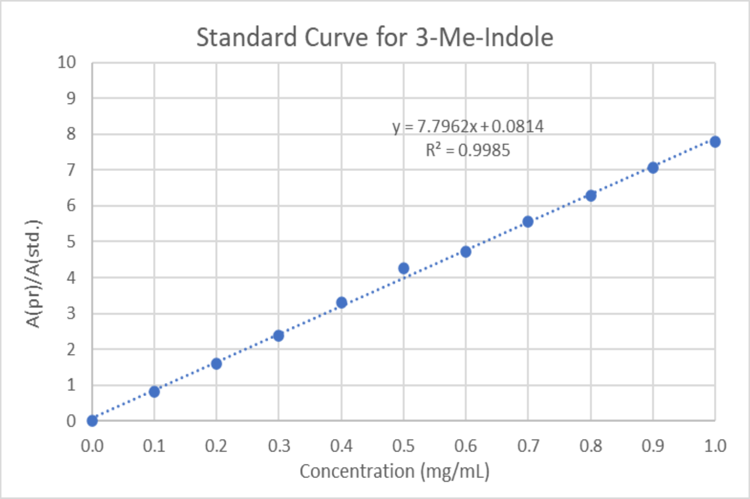

Figure S5. Standard Curve of 3-methyl-1H-indole

5-methoxy-3-methylindolin-2-one (5)

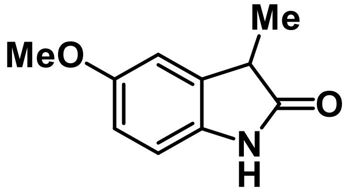

Synthesized from commercially available 5-methoxy-3-methyl-1H-indole following General Procedure F

Yield: 91%

Purification: Eluted with the gradient of 30-70 % EtOAc in Hexanes (R_f = 0.48)

^{1}H NMR (400 MHz, $CDCl_3$) δ. 8.66 (br s, 1 H), 6.82 (m, 2 H), 6.74 (dd, J = 8.4 Hz, 2.5 Hz, 1 H), 3.75 (s, 3 H), 3.45 (q, J = 7.7 Hz, 1 H), 1.49 (d, J = 7.7 Hz, 3 H)

^{13}C NMR (101 MHz, $CDCl_3$) δ. 181.8, 155.8, 134.9, 132.7, 112.3, 111.1, 110.2, 55.8, 41.7, 15.3

These NMR spectra were consistent with the literature precedent[13].

6-bromo-3-methylindolin-2-one (6)

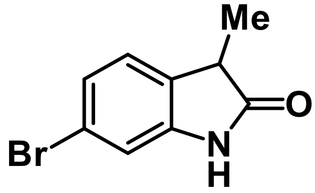

Synthesized from commercially available 6-bromo-3-methyl-1H-indole following General Procedure F

Yield: 70%

Purification: Eluted with the gradient of 10-50 % EtOAc in Hexanes (R_f = 0.50)

^{1}H NMR (400 MHz, $CDCl_3$) δ. 8.35 (br s, 1 H), 7.17 (dd, J = 7.9 Hz, 1.9 Hz, 1 H), 7.08 (m, 2 H), 3.41 (q, J = 7.6 Hz, 1 H), 1.48 (d, J = 7.7 Hz, 3 H)

^{13}C NMR (101 MHz, $CDCl_3$) δ. 180.6, 142.3, 130.1, 125.3, 125.2, 121.3, 113.0, 40.6, 15.12

These NMR spectra were consistent with the literature precedent. [13]

5-bromo-3-methylindolin-2-one (7)

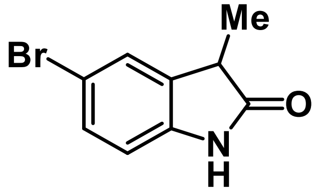

Synthesized from commercially available 5-bromo-3-methyl-1H-indole following General Procedure F

Yield: 70%

Purification: Eluted with the gradient of 10-50 % EtOAc in Hexanes (R_f = 0.52)

^{1}H NMR (400 MHz, $CDCl_3$) δ. 8.11 (br s, 1 H), 7.34 (m, 2 H), 6.77 (d, J = 8.7 Hz, 1 H), 3.47 (q, J = 7.7 Hz, 1 H), 1.49 (d, J = 7.7 Hz, 3 H)

^{13}C NMR (101 MHz, $CDCl_3$) δ. 180.5, 140.1, 133.3, 130.8, 127.2, 115.1, 111.1, 41.1, 15.1

These NMR spectra were consistent with the literature precedent[13].

6-fluoro-3-methylindolin-2-one (8)

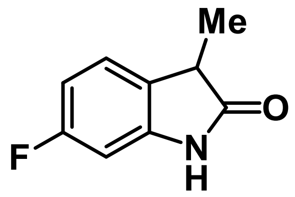

Synthesized from commercially available 6-fluoro-3-methyl-1H-indole following General Procedure F

Yield: 83%

Purification: Eluted with the gradient of 10-50 % EtOAc in Hexanes (R_f = 0.46)

^{1}H NMR (400 MHz, $CDCl_3$) δ. 8.12 (br s, 1 H), 7.14 (dd, J = 8.2 Hz, 5.3 Hz, 1 H), 6.69 (m, 2 H), 3.43 (q, J = 7.6 Hz), 1.48 (d, J = 7.6 Hz, 3 H)

^{13}C NMR (101 MHz, $CDCl_3$) δ. 181.8, 162.7 (d, J = 245 Hz, 1C), 142.4 (d, J = 12 Hz, 1C), 126.5 (d, J = 3 Hz, 1C), 124.7 (d, J = 10 Hz, 1C), 108.7 (d, J = 22 Hz, 1C), 98.4 (d, J = 27 Hz, 1C), 40.6, 15.3

These NMR spectra were consistent with the literature precedent[13].

methyl 2-(2-oxoindolin-3-yl)acetate (9)

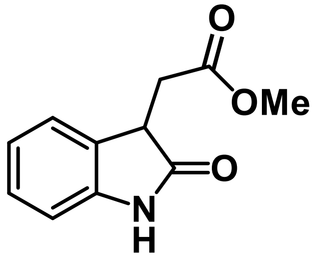

Synthesized from commercially available methyl 2-(1H-indol-3-yl)acetate following General Procedure F

Yield: 81%

Purification: Eluted with the gradient of 30-70% EtOAc in hexanes (R_f = 0.22)

^{1}H NMR (400 MHz, $CDCl_3$) δ. 8.68 (br s, 1 H), 7.19 (m, 2 H), 6.98 (td, J = 7.6 Hz, J = 1.0 Hz, 1 H), 6.89 (d, J = 7.7 Hz, 1 H), 3.89 (dd, J = 8.7 Hz, 3.5 Hz, 1 H), 3.25 (dd, J = 18.4 Hz, 3.6 Hz, 1 H), 2.90 (dd, J = 18.4 Hz , 8.6 Hz, 1 H), 2.23 (s, 3 H)

^{13}C NMR (101 MHz, $CDCl_3$) δ. 179.2, 171.6, 141.6, 128.7, 128.4, 124.1, 122.5, 109.9, 52.1, 42.3, 34.6

These NMR spectra were consistent with the literature precedent[13].

methyl 3-(2-oxoindolin-3-yl)propanoate (10)

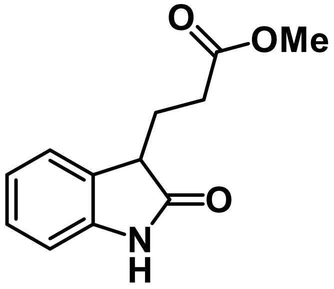

Synthesized from commercially available methyl 3-(1H-indol-3-yl)propanoate following General Procedure F

Yield: 81%

Purification: Eluted with the gradient of 50-70 % EtOAc in Hexanes (R_f = 0.47)

^{1}H NMR (400 MHz, $CDCl_3$) δ. 8.40 (br s, 1 H), 7.22 (m, 2 H), 7.06 (m, 1 H), 6.90 (d, J = 7.5 Hz, 1 H), 3.63 (s, 3 H), 2.55 (m, 1 H), 2.40 (m, 1 H), 2.28 (m, 2 H)

^{13}C NMR (101 MHz, $CDCl_3$) δ. 179.6, 173.4, 141.4, 128.6, 128.2, 124.3, 122.5, 109.8, 51.7, 44.8, 30.0, 25.5

These NMR spectra were consistent with the literature precedent[13].

N-methoxy-N-methyl-2-(2-oxoindolin-3-yl)acetamide (11)

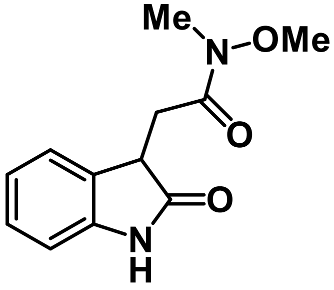

Synthesized from commercially available 2-(1H-indol-3-yl)-N-methoxy-N-methylacetamide following General Procedure F

Yield: 82%

Purification: Eluted with the gradient of 30-90 % EtOAc in Hexanes (R_f = 0.35)

^{1}H NMR (400 MHz, $CDCl_3$) δ. 8.76 (br s, 1 H), 7.28 (m, 1 H), 7.19 (m, 1 H), 6.98 (td, J = 7.5 Hz, J = 1.0 Hz, 1 H), 6.89 (d, J = 7.6 Hz, 1 H), 3.93 (dd, J = 8.9 Hz, 3.8 Hz, 1 H), 3.67 (s, 3 H), 3.28 (m, 1 H), 3.21 (s, 3 H), 2.93 (dd, J = 16.9 Hz, 8.8 Hz, 1 H)

^{13}C NMR (101 MHz, $CDCl_3$) δ. 180.1, 171.6, 141.6, 129.7, 128.0, 124.5, 122.4, 109.8, 61.4, 42.0, 33.2, 32.4

These NMR spectra were consistent with the literature precedent[13].

2-(2-(2-oxoindolin-3-yl)ethyl)isoindoline-1,3-dione (12)

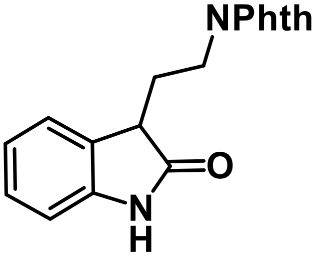

Synthesized from commercially available 2-(2-(1H-indol-3-yl)ethyl)isoindoline-1,3-dione following General Procedure F

Yield: 74%

Purification: Eluted with the gradient of 10-70 % EtOAc in Hexanes (R_f = 0.33)

^{1}H NMR (400 MHz, $CDCl_3$) δ. 7.90 (br s, 1 H), 7.77 (dd, J = 5.5 Hz, 3.0 Hz, 2 H), 7.66 (dd, J = 5.5 Hz, 3.0 Hz, 2 H), 7.30 (d, J 7.5 Hz, 1 H), 7.08 (t, J = 7.7 Hz, 1 H), 6.88 (t, J = 7.5 Hz, 1 H), 6.81 (d, J = 7.76 Hz, 1 H), 3.98 (m, 1 H), 3.82 (m, 1 H), 3.54 (t, J = 6.1 Hz, 1 H), 2.52 (m, 1 H), 2.33 (m, 1 H)

^{13}C NMR (101 MHz, $CDCl_3$) δ. 179.3, 168.2, 141.5, 133.8, 132.0, 128.6, 128.0, 124.0, 123.1, 122.4, 109.9, 43.9, 35.1, 28.4

These NMR spectra were consistent with the literature precedent[13].

N-(2-(2-oxoindolin-3-yl)ethyl)acetamide (13)

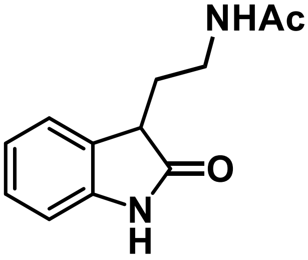

Synthesized from synthesized N-(2-(1H-indol-3-yl)ethyl)acetamide following General Procedure F

Yield: 53%

Purification: Eluted with 5% MeOH in DCM (R_f = 0.44)

^{1}H NMR (400 MHz, $CDCl_3$) δ. 8.22 (br s, 1H), 7.28 (d, J = 7.4 Hz, 1H), 7.22 (t, J = 7.7 Hz, 1H), 7.05 (t, J = 7.6 Hz, 1H), 6.88 (d, J = 7.8 Hz, 1H), 6.48 (br s, 1H), 3.49 (m, 3H), 2.26 (m, 1H), 2.05 (m, 1H), 1.97 (s, 3H)

^{13}C NMR (101 MHz, $CDCl_3$) δ. 180.0, 170.6, 141.0, 129.2, 128.3, 124.3, 122.8, 109.8, 44.4, 37.4, 29.9, 23.2

These NMR spectra were consistent with the literature precedent[14].

3-(2-oxopropyl)indolin-2-one (14)

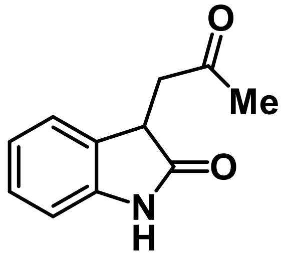

Synthesized from commercially available 1-(1H-indol-3-yl)propan-2-one following General Procedure F

Yield: 92%

Purification: Eluted with the gradient of 30-70% EtOAc in hexanes (R_f = 0.22)

^{1}H NMR (400 MHz, $CDCl_3$) δ.8.68 (br s, 1H), 7.19 (m, 2H), 6.98 (td, J = 7.6 Hz, 1.0 Hz, 1H), 6.89 (d, J = 7.7 Hz, 1H), 3.89 (dd, J = 8.7 Hz, 3.5 Hz, 1H), 3.25 (dd, J = 18.4 Hz, 3.6 Hz, 1H), 2.90 (dd, J = 18.4 Hz, 8.6 Hz, 1H), 2.23 (3H)

^{13}C NMR (101 MHz, $CDCl_3$) δ. 205.4, 180.0. 141.4, 129.5, 128.1, 124.4, 122.5, 109.8, 44.2, 41.5, 30.0

These NMR spectra were consistent with the literature precedent[13].

2-(2-oxoindolin-3-yl)acetonitrile (15)

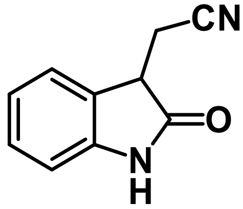

Synthesized from commercially available 2-(1H-indol-3-yl)acetonitrile following General Procedure F

Yield: 59%

Purification: Eluted with the gradient of 10-50 % EtOAc in Hexanes (R_f = 0.36)

^{1}H NMR (400 MHz, $CDCl_3$) δ. 7.96 (br s, 1H), 7.50 (d, J = 7.5 Hz, 1H), 7.31 (m, 1H), 7.12 (t, J = 8.2 Hz, 1H), 6.93 (d, J = 7.8 Hz, 1H), 3.71 (dd, J = 9.1 Hz, 4.8 Hz, 1H), 3.10 (dd, J = 16.8 Hz, 4.7 Hz, 1H), 2.75 (dd, J = 16.9 Hz, 9.1 Hz, 1H)
^{13}C NMR (101 MHz, $CDCl_3$) δ. 176.3, 141.1, 129.5, 126.2, 124.6, 123.3, 117.1, 110.3, 41.8, 18.9

These NMR spectra were consistent with the literature precedent[13].

2-(5-methoxy-2-oxoindolin-3-yl)acetonitrile (16)

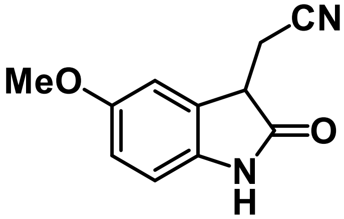

Synthesized from commercially available 2-(5-methoxy-1H-indol-3-yl)acetonitrile following General Procedure F

Yield: 67%

Purification: Eluted with the gradient of 30-70 % EtOAc in Hexanes (R_f = 0.32)

^{1}H NMR (400 MHz, $CDCl_3$) δ. 7.70 (br s, 1 H), 7.10 (s, 1 H), 6.83 (s, 2 H), 3.81 (s, 3 H), 3.68 (dd, J = 9.0 Hz, 4.7 Hz, 1 H), 3.10 (dd, J = 16.8 Hz, 4.7 Hz, 1 H), 2.74 (dd, J = 16.9 Hz, 9.1 Hz, 1 H)

^{13}C NMR (101 MHz, $CDCl_3$) δ. 176.2, 156.2, 134.4, 127.5, 117.1, 114.2, 111.5, 110.8, 55.9, 42.2, 19.0

These NMR spectra were consistent with the literature precedent[13].

3-benzylindolin-2-one (17)

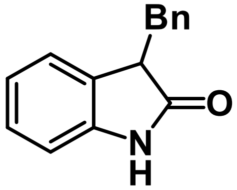

Synthesized from commercially available 3-benzyl-1H-indole following General Procedure F

Yield: 79%

Purification: Eluted with the gradient of 10-50 % EtOAc in Hexanes (R_f = 0.47)

^{1}H NMR (400 MHz, $CDCl_3$) δ. 7.95 (br s, 1H), 7.25 (m, 3H), 7.17 (m, 3H), 6.88 (m, 2H), 6.76 (d, J = 7.5 Hz, 1H), 3.75 (dd, J = 9.2, 4.6 Hz, 1H), 3.49(dd, J = 13.7 Hz, 4.6 Hz, 1H), 2.95 (dd, J = 13.7 Hz, 9.2 Hz, 1H)

^{13}C NMR (101 MHz, $CDCl_3$) δ. 179.6, 141.4, 137.8, 129.5, 129.0, 128.4, 128.0, 126.7, 124.9, 122.1, 109.7, 47.5, 36.6

These NMR spectra were consistent with the literature precedent[15].

3-phenylindolin-2-one (18)

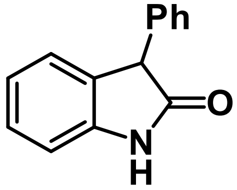

Synthesized from commercially 3-phenyl-1H-indole following General Procedure F

Yield: 58%

Purification: Eluted with the gradient of 10-50 % EtOAc in Hexanes (R_f = 0.50)

^{1}H NMR (400 MHz, $CDCl_3$) δ. 7.82 (br s, 1H), 7.38 (m, 3H), 7.23 (m, 3H), 7.14 (d, J = 7.4 Hz, 1H), 7.04 (t, J = 7.5 Hz, 1H), 6.93 (d, J = 7.8 Hz, 1H), 4.64 (s, 1H)

^{13}C NMR (101 MHz, $CDCl_3$) δ. 178.8, 141.7, 136.5, 129.6, 129.0, 128.5, 128.4, 127.7, 125.3, 122.7, 110.1, 52.7

These NMR spectra were consistent with the literature precedent[16].

1,3-dimethylindolin-2-one (19)

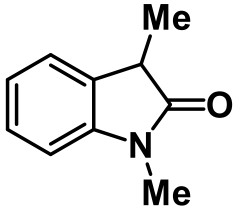

Synthesized from commercially available 1,3-dimethyl-1H-indole following General Procedure F

Yield: 82%

Purification: Eluted with the gradient of 10-50 % EtOAc in Hexanes (R_f = 0.67)

^{1}H NMR (400 MHz, $CDCl_3$) δ. 7.28 (m, 2 H), 7.07 (td, J = 7.5 Hz, 1.0 Hz, 1 H), 6.84 (d, J = 7.7 Hz, 1 H), 3.45 (q, J = 7.6 Hz, 1 H), 3.22 (s, 3 H), 1.49 (d, J 7.7 Hz, 3 H)

^{13}C NMR (101 MHz, $CDCl_3$) δ. 178.7, 144.0, 130.7, 127.9, 123.5, 122.4, 107.9, 40.6, 26.2, 15.4

These NMR spectra were consistent with the literature precedent[17].

N-(2-(5-methoxy-2-oxoindolin-3-yl)ethyl)acetamide (20)

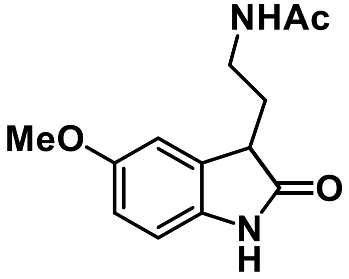

Synthesized from commercially available N-(2-(5-methoxy-1H-indol-3-yl)ethyl)acetamide following General Procedure F

Yield: 79%

Purification: Eluted with 5% MeOH in EtOAc (R_f = 0.27)

^{1}H NMR (400 MHz, $CDCl_3$) δ. 8.52 (br s, 1 H), 6.90 (s, 1 H), 6.79 (d, J = 8.4 Hz, 1 H), 6.74 (dd, J = 8.5, 3.1 Hz, 1 H), 6.49 (br s, 1 H), 3.78 (s, 3 H), 3.48 (m, 3 H), 2.23 (m, 1 H), 2.03 (m, 1 H), 1.95 (s, 3 H)

^{13}C NMR (101 MHz, $CDCl_3$) δ. 180.2, 170.6, 156.0, 134.5, 130.5, 112.9, 111.2, 110.2, 55.8, 44.9, 37.2, 30.0, 23.2

HRMS: calculated for $C_{13}H_{16}N_2O_3$ $[M+H^+]^+$: 249.1234. Found $[M+H^+]^+$: 249.1234.

IR: (cm^{-1}) 3293, 2987, 2900, 1690, 1637, 1213, 1034.

1'-acetylspiro[indoline-3,3'-pyrrolidin]-2-one (4)

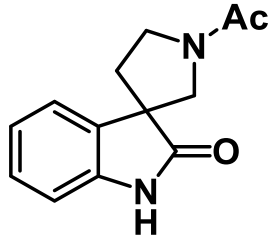

Synthesized from synthesized 1-(1,3,4,9-tetrahydro-2H-pyrido[3,4-b]indol-2-yl)ethan-1-one following General Procedure H

Yield: 90%

Purification: Eluted with the gradient of 10-50 % EtOAc in Hexanes (R_f = 0.67)

^{1}H NMR (400 MHz, DMSO) δ 10.53 (d, J = 4.9 Hz, 1H), 7.30 – 7.26 (m, 1H), 7.21 (tdd, J = 7.6, 2.2, 1.2 Hz, H), 6.98 (tdd, J = 7.5, 4.3, 1.1 Hz, 1H), 6.91 – 6.83 (m, 1H), 3.90 – 3.60 (m, 3H), 3.56 – 3.45 (m, 1H), 2.32 – 2.07 (m, 2H), 2.04 (s, 2H), 1.94 (s, 1H).

^{13}C NMR (101 MHz, DMSO) δ 179.63, 179.42, 168.92, 168.63, 141.98, 141.93, 132.56, 132.25, 128.78, 128.72, 123.28, 122.40, 122.37, 110.01, 54.89, 53.59, 53.11, 51.62, 46.39, 45.09, 36.30, 34.86, 22.80, 22.67.

HRMS: calculated for $C_{13}H_{15}N_2O_2$ [M+H$^+$]$^+$: 231.1128. Found [M+H$^+$]$^+$: 231.1124.

IR: (cm^{-1}) 3177, 2878, 1706, 1614, 1469, 1188, 750.

Standard Curve for Analytical Runs:

Procedure for using standard curve is as follows: 1,3,5-bromobenzene (8mg/mL solution, 100 µL) is added to 900 µL of the reaction mixture and yield is determined by LCMS analysis based on the below standard curve. LCMS conditions: 2.5 µL injection volume, 0.5 mL/min mobile phase rate, 10-98% solvent B over 6.25 minutes. Mobile Phase: Solvent A- H_2O w/ 0.1% formic acid, Solvent B- MeCN w/ 0.1% formic acid.

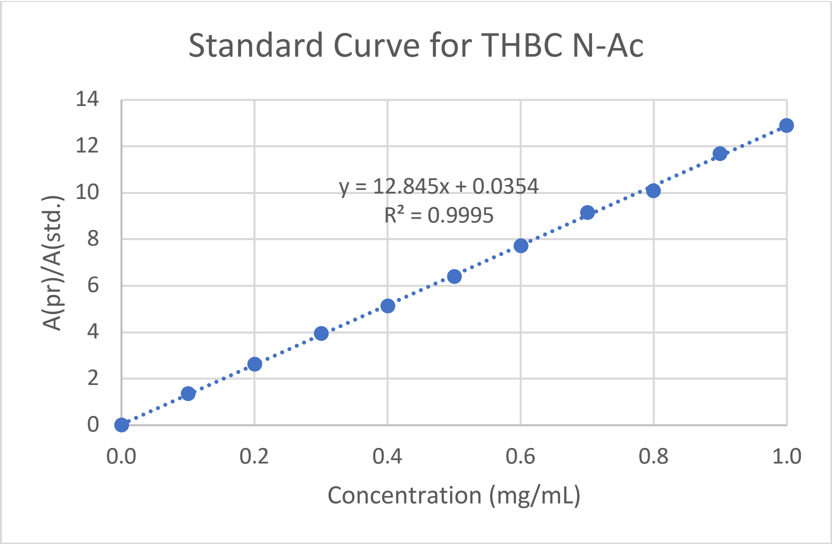

Figure S6. Standard Curve of 1-(1,3,4,9-tetrahydro-2H-pyrido[3,4-b]indol-2-yl)ethan-1-one

tert-butyl 2-oxospiro[indoline-3,3'-pyrrolidine]-1'-carboxylate (21)

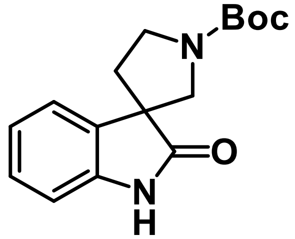

Synthesized from synthesized tert-butyl 1,3,4,9-tetrahydro-2H-pyrido[3,4-b]indole-2-carboxylate following General Procedure H

Yield: 88%

Purification: Eluted with the gradient of 0-2% MeOH in DCM (R_f = 0.5)

^{1}H NMR (400 MHz, $CDCl_3$) δ 8.96 – 8.43 (m, 1H), 7.33 – 7.24 (m, 1H), 7.21 – 7.14 (m, 1H), 7.08 (tdd, J = 7.6, 4.5, 1.0 Hz, 1H), 6.98 (ddt, J = 12.2, 7.8, 0.9 Hz, 1H), 4.08 – 3.95 (m, 1H), 3.94 – 3.81 (m, 2H), 3.81 – 3.62 (m, 1H), 2.55 – 2.41 (m, 1H), 2.27 (dt, J = 12.9, 7.2 Hz, 1H), 2.22 – 2.08 (m, 3H).

^{13}C NMR (101 MHz, $CDCl_3$) δ 180.11, 179.01, 169.57, 169.42, 140.32, 140.00, 132.52, 131.61, 128.76, 128.70, 123.19, 123.00, 122.71, 110.26, 110.14, 55.43, 53.56, 53.52, 51.77, 46.64, 45.14, 36.49, 35.12, 22.63, 22.48.

HRMS: calculated for $C_{16}H_{19}N_2O_3$ [M-H$^+$]$^-$: 287.1401. Found [M-H$^+$]$^-$: 287.1405.

IR: (cm^{-1}) 3289, 2969, 2888, 1720, 1692, 1617, 1395, 1164, 748.

2,2,2-trichloroethyl 2-oxospiro[indoline-3,3'-pyrrolidine]-1'-carboxylate (22)

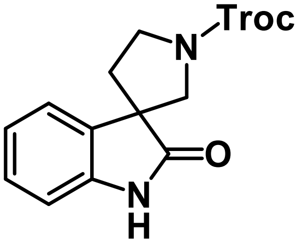

Synthesized from synthesized 2,2,2-trichloroethyl 1,3,4,9-tetrahydro-2H-pyrido[3,4-b]indole-2-carboxylate following General Procedure H

Yield: 83%

Purification: Eluted with the gradient of 10-50 % EtOAc in Hexanes (R_f = 0.45)

^{1}H NMR (400 MHz, $CDCl_3$) δ 8.07 (d, *J* = 15.5 Hz, 1H), 7.30 – 7.28 (m, 1H), 7.28 – 7.18 (m, 1H), 7.09 (tdd, *J* = 7.5, 2.8, 1.0 Hz, 1H), 6.96 (ddt, *J* = 7.7, 2.8, 0.8 Hz, 1H), 4.98 – 4.69 (m, 2H), 4.09 – 3.95 (m, 1H), 3.95 – 3.83 (m, 2H), 3.76 (dd, *J* = 11.2, 7.9 Hz, 1H), 2.57 – 2.44 (m, 1H), 2.20 (dddd, *J* = 17.7, 12.7, 7.6, 5.3 Hz, 1H). (mixture of rotamers)

^{13}C NMR (151 MHz, $CDCl_3$) δ 179.46, 179.28, 152.84, 140.00, 139.93, 132.11, 131.85, 128.78, 128.76, 123.20, 123.16, 122.82, 122.79, 110.17, 110.14, 95.72, 95.54, 74.95, 74.93, 54.42, 54.03, 53.03, 52.19, 45.92, 45.42, 36.22, 35.50. (peaks for both rotamers shown)

HRMS: calculated for $C_{14}H_{12}N_2O_3{}^{35}Cl_3$ $[M-H^+]^-$: 360.9919. Found $[M-H^+]^-$: 360.9921.

IR: (cm^{-1}) 3392, 3195, 2952, 1717, 1621, 1117, 748, 711.

methyl 2-oxospiro[indoline-3,3'-pyrrolidine]-1'-carboxylate (23)

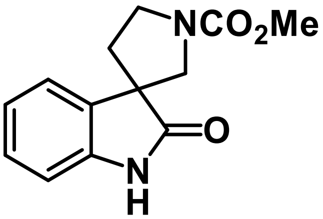

Synthesized from synthesized methyl 1,3,4,9-tetrahydro-2H-pyrido[3,4-b]indole-2-carboxylate following General Procedure H

Yield: 92%

Purification: Eluted with the gradient of 0-3 % MeOH in DCM (R_f = 0.5)

^{1}H NMR (400 MHz, DMSO) δ 10.54 (s, 1H), 7.29 – 7.17 (m, 2H), 6.98 (td, *J* = 7.6, 1.1 Hz, 1H), 6.87 (d, *J* = 7.7 Hz, 1H), 3.78 – 3.46 (m, 7H), 2.31 – 2.03 (m, 1H).

^{13}C NMR (151 MHz, $CDCl_3$) δ 179.46, 179.28, 152.84, 140.00, 139.93, 132.11, 131.85, 128.78, 128.76, 123.20, 123.16, 122.82, 122.79, 110.17, 110.14, 95.72, 95.54, 74.95, 54.42, 54.03, 53.03, 52.19, 45.92, 45.42, 36.22, 35.50.

HRMS: calculated for $C_{13}H_{13}N_2O_3$ $[M-H^+]^-$: 245.0932. Found $[M-H^+]^-$: 245.0932.

IR: (cm^{-1}) 3218, 1954, 1879, 1672, 1617, 1447, 1387, 1188, 747.

1'-acetyl-1-methylspiro[indoline-3,3'-pyrrolidin]-2-one (24)

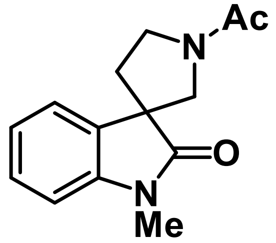

Synthesized from synthesized 1-(9-methyl-1,3,4,9-tetrahydro-2H-pyrido[3,4-b]indol-2-yl)ethan-1-one following General Procedure H

Yield: 95%

Purification: Eluted with the gradient of 10-75 % EtOAc in Hexanes (R_f = 0.55)

^{1}H NMR (400 MHz, $CDCl_3$) δ 7.32 (dtd, J = 9.0, 7.7, 1.3 Hz, 1H), 7.16 (dddd, J = 7.5, 6.2, 1.3, 0.6 Hz, 1H), 7.07 (tdd, J = 7.5, 5.5, 1.0 Hz, 1H), 6.88 (ddt, J = 10.6, 7.8, 0.8 Hz, 1H), 4.04 – 3.91 (m, 1H), 3.90 – 3.76 (m, 2H), 3.75 – 3.49 (m, 1H), 3.23 (d, J = 9.3 Hz, 3H), 2.50 – 2.38 (m, 1H), 2.24 – 2.17 (m, 1H), 2.17 – 2.00 (m, 3H). (mixture of rotamers)

^{13}C NMR (151 MHz, $CDCl_3$) δ 177.76, 176.66, 169.48, 169.36, 143.09, 142.72, 132.30, 131.32, 128.76, 128.69, 123.27, 123.05, 122.35, 108.52, 108.43, 55.50, 53.57, 53.15, 51.41, 46.68, 45.16, 36.44, 35.04, 26.57, 26.44, 22.66, 22.52. (peaks for both rotamers shown)

HRMS: calculated for $C_{14}H_{17}N_2O_2$ [M+H$^+$]$^+$: 245.1285. Found [M+H$^+$]$^+$: 245.1283.

IR: (cm^{-1}) 3391, 2904, 2839, 1704. 1625, 1437, 746.

dimethyl (5'S)-2-oxospiro[indoline-3,3'-pyrrolidine]-1',5'-dicarboxylate (25)

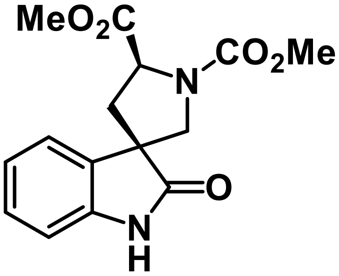

Synthesized from synthesized dimethyl (S)-1,3,4,9-tetrahydro-2H-pyrido[3,4-b]indole-2,3-dicarboxylate following General Procedure H

Yield: 85%

Purification: Eluted with the gradient of 10-75 % EtOAc in Hexanes (R_f = 0.33)

^{1}H NMR (600 MHz, DMSO) δ 10.66 (s, 1H), 7.29 – 7.21 (m, 1H), 7.18 – 7.06 (m, 1H), 6.99 (dd, *J* = 8.9, 6.3 Hz, 1H), 6.94 – 6.87 (m, 1H), 4.91 – 4.43 (m, 1H), 3.76 – 3.67 (m, 3H), 3.68 – 3.61 (m, 4H), 3.56 (dt, *J* = 14.1, 10.0 Hz, 1H), 2.52 – 2.38 (m, 1H), 2.36 – 2.22 (m, 1H).

^{13}C NMR (151 MHz, DMSO) δ 177.25, 177.00, 172.46, 172.29, 154.86, 154.51, 141.55, 133.49, 133.46, 128.90, 122.82, 122.73, 122.64, 122.61, 110.32, 110.30, 59.11, 58.54, 55.93, 55.41, 53.17, 53.11, 52.85, 52.72, 51.99, 21.24.

HRMS: calculated for $C_{15}H_{15}N_2O_5$ $[M-H^+]^-$: 303.0986. Found $[M-H^+]^-$: 303.0989.

IR: (cm^{-1}) 3267, 2954, 1698, 1618, 1446, 1178, 749.

$[\alpha]^{20}_D$ = -16.80 (c = 0.500, MeOH)

2,2-dimethyl-4,5-dihydro-2H-spiro[furan-3,3'-indolin]-2'-one (26)

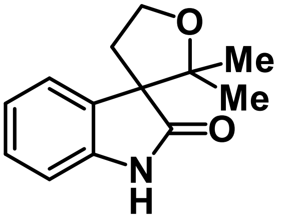

Synthesized from synthesized Synthesis of 1,1-dimethyl-1,3,4,9-tetrahydropyrano[3,4-b]indole following General Procedure H

Yield: 89%

Purification: Eluted with the gradient of 10-50 % EtOAc in Hexanes (R_f = 0.35)

^{1}H NMR (400 MHz, DMSO) δ 10.38 (s, 1H), 7.27 – 7.13 (m, 2H), 6.96 (td, J = 7.6, 1.1 Hz, 1H), 6.87 – 6.77 (m, 1H), 4.12 – 3.98 (m, 3H), 2.43 (ddd, J = 12.6, 9.0, 6.1 Hz, 1H), 2.24 (ddd, J = 12.6, 8.7, 6.0 Hz, 1H), 1.15 (s, 4H), 0.96 (s, 3H).

^{13}C NMR (101 MHz, $CDCl_3$) δ. 178.54, 141.78, 131.36, 127.87, 124.70, 121.25, 109.17, 83.76, 63.78, 59.20, 35.28, 24.37, 23.21.

HRMS: calculated for $C_{13}H_{14}NO_2$ [M-H$^+$]$^-$: 216.1030. Found [M-H$^+$]$^-$: 216.1035.

IR: (cm^{-1}) 3268, 3057, 2969, 2879, 1688, 1615, 1467, 1040, 740.

4',5'-dihydrodispiro[cyclohexane-1,2'-furan-3',3''-indolin]-2''-one (27)

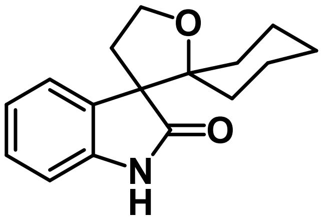

Synthesized from synthesized 4',9'-dihydro-3'H-spiro[cyclohexane-1,1'-pyrano[3,4-b]indole] following General Procedure H

Yield: 85%

Purification: Eluted with the gradient of 10-50 % EtOAc in Hexanes (R_f = 0.45)

^{1}H NMR (400 MHz, $CDCl_3$) δ 8.29 (s, 1H), 7.28 – 7.20 (m, 2H), 7.05 (td, *J* = 7.6, 1.1 Hz, 1H), 6.92 (dt, *J* = 7.8, 0.8 Hz, 1H), 4.27 (ddd, *J* = 9.3, 8.6, 5.7 Hz, 1H), 4.17 (td, *J* = 8.9, 5.8 Hz, 1H), 2.66 (ddd, *J* = 12.7, 9.3, 5.8 Hz, 1H), 2.32 (ddd, *J* = 12.7, 9.1, 5.7 Hz, 1H), 2.06 – 1.95 (m, 1H), 1.72 – 1.60 (m, 5H), 1.40 – 1.33 (m, 1H), 1.33 – 1.28 (m, 1H), 1.14 (dd, *J* = 13.2, 4.1 Hz, 1H), 1.10 (s, 1H).

^{13}C NMR (151 MHz, $CDCl_3$) δ 180.54, 140.82, 131.26, 128.05, 125.34, 122.18, 109.76, 86.25, 64.12, 60.93, 35.65, 32.00, 30.86, 25.51, 22.54, 22.40.

HRMS: calculated for $C_{16}H_{18}NO_2$ $[M-H^+]^-$: 256.1343 Found $[M-H^+]^-$: 256.1341.

IR: (cm^{-1}) 3276, 3060, 2926, 2877, 1725, 1696, 1614, 1089, 741.

tert-butyl 2-oxo-4',5'-dihydrodispiro[indoline-3,3'-furan-2',4''-piperidine]-1''-carboxylate (28)

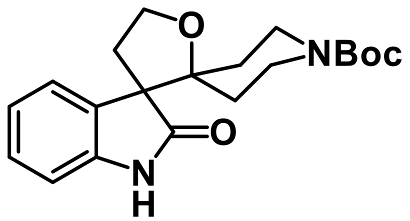

Synthesized from synthesized tert-butyl 4',9'-dihydro-3'H-spiro[piperidine-4,1'-pyrano[3,4-b]indole]-1-carboxylate following General Procedure H

Yield: 75%

Purification: Eluted with the gradient of 10-75 % EtOAc in Hexanes (R_f = 0.45)
z
^{1}H NMR (400 MHz, DMSO) δ 10.44 (s, 1H), 7.26 – 7.16 (m, 2H), 6.97 (td, *J* = 7.5, 1.1 Hz, 1H), 6.84 (dd, *J* = 7.7, 1.1 Hz, 1H), 4.10 (pd, *J* = 8.5, 6.0 Hz, 2H), 3.79 (s, 2H), 2.87 (s, 2H), 2.43 (ddd, *J* = 12.6, 8.8, 5.9 Hz, 1H), 2.28 (ddd, *J* = 12.6, 8.7, 6.2 Hz, 1H), 1.76 (dd, *J* = 13.7, 2.7 Hz, 1H), 1.50 (d, *J* = 13.6 Hz, 1H), 1.33 (s, 9H), 1.32 – 1.24 (m, 1H), 1.04 (td, *J* = 13.1, 4.9 Hz, 1H).

^{13}C NMR (101 MHz, DMSO) δ 178.59, 154.06, 142.29, 130.60, 128.55, 125.53, 121.75, 109.78, 83.35, 79.02, 64.49, 59.98, 35.13, 28.46.

HRMS: calculated for $C_{20}H_{25}N_2O_4$ [M-H^{+}]$^{-}$: 357.1820. Found [M-H^{+}]$^{-}$: 357.1818.

IR: (cm^{-1}) 3267, 2970, 2925, 1693, 1666, 1618, 1470, 1045, 745.

3-(hydroxymethyl)-3-methylindolin-2-one (29)

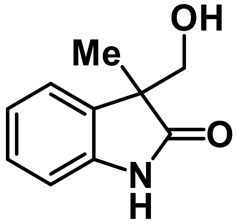

Synthesized from synthesized 6-methoxy-2-methyl-2,3,4,9-tetrahydro-1H-pyrido[3,4-b]indole following General Procedure H

Yield: 51%

Purification: Eluted with the gradient of 10-75 % EtOAc in Hexanes (R_f = 0.33)

^{1}H NMR (400 MHz, DMSO) δ 10.27 (s, 1H), 7.25 (dd, *J* = 7.4, 1.3 Hz, 1H), 7.15 (td, *J* = 7.7, 1.3 Hz, 1H), 6.94 (td, *J* = 7.5, 1.1 Hz, 1H), 6.81 (dt, *J* = 7.6, 0.9 Hz, 1H), 4.84 (t, *J* = 5.2 Hz, 1H), 3.59 (qd, *J* = 10.2, 5.3 Hz, 2H), 1.14 (s, 3H).

^{13}C NMR (151 MHz, DMSO) δ 181.10, 142.63, 134.14, 127.85, 123.82, 121.56, 109.43, 66.75, 50.77, 19.55.

HRMS: calculated for $C_{10}H_{12}NO_2$ $[M+H^+]^+$: 178.0863. Found $[M+H^+]^+$: 178.0862.

IR: (cm^{-1}) 3331, 3188, 2965, 2923, 1703, 1620, 1470, 1044, 744.

ethyl 3-methyl-2-oxoindoline-3-carboxylate (30)

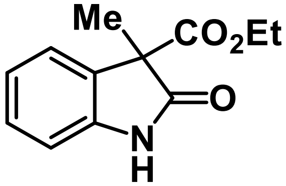

Synthesized from commercially available ethyl 3-methyl-1H-indole-2-carboxylate following General Procedure H

Yield: 30%

Purification: Eluted with the gradient of 10-50 % EtOAc in Hexanes (R_f = 0.5)

^{1}H NMR (400 MHz, DMSO) δ 10.68 (s, 1H), 7.25 (td, *J* = 7.7, 1.3 Hz, 1H), 7.22 – 7.19 (m, 1H), 6.99 (td, *J* = 7.6, 1.1 Hz, 1H), 6.90 (dt, *J* = 7.8, 0.9 Hz, 1H), 4.05 (qq, *J* = 10.9, 7.1 Hz, 2H), 1.49 (s, 3H), 1.05 (t, *J* = 7.1 Hz, 3H).

^{13}C NMR (151 MHz, DMSO) δ 176.37, 169.93, 142.61, 131.27, 129.37, 123.38, 122.44, 110.36, 61.75, 55.32, 20.13, 14.29.

HRMS: calculated for $C_{12}H_{12}NO_3$ $[M-H^+]^-$: 218.0823. Found $[M-H^+]^-$: 218.0817.

IR: (cm^{-1}) 3325, 2981, 2925, 1712, 1470, 1232, 1105, 751.

Additional Reaction Experiments

Gram-Scale Procedure for Oxidation of 3-methyl-1H-indole (1) with *Ci*VCPO in Whole Cells: An enzyme aliquots of VHPO whole cell from *Curvularia inaequalis* (*Ci*VCPO, OD_{600} = 18.5, 20 mL) was removed from a -80 °C freezer and allowed to warm to room temperature over 10 min. The thawed aliquots were then combined with an aqueous solution of 50 mM Na_3VO_4 (4 mL, 0.025 equiv relative to starting material) in a 50 mL falcon tube and stored at room temperature. To ensure mixing, the resulting solution was vortexed for 1 min and placed on the benchtop until further use. To a 4L Erlenmeyer flask, purified H_2O (953 mL), 500 mM pH 5 citrate buffer (400 mL), 100 mM KBr (16 mL, 0.2 equiv), and 400 mL MeCN was added. Then, 3-methyl-1H-indole (**1**) (1.0095 g, 7.70 mmol,1 equiv) was dissolved in MeCN (200 mL) and added to the reaction mixture. The falcon tube containing *Ci*VCPO and Na_3VO_4 was added to the reaction mixture as well. A 10% stock of H_2O_2 (5.28 mL, 2 equiv) was then added to the reaction mixture and the reaction was allowed to shake at room temperature for 4 h using Infors HT Multitron Incubator Shaker at 220 rpm. After this time, the reaction mixture was divided equally into four 1-L centrifugation bucket and mixed with 300 mL of EtOAc. The organic and aqueous layers were then separated using Beckman Coulter Avanti JXN-26 centrifuge at 3.5 krpm at 10 °C for 15 minutes. After removal of the organic layers, two additional rounds of extraction were performed with 400 mL EtOAc and 200 mL of saturated brine each time. The combined organic layers were dried over Na_2SO_4 for 10 minutes, concentrated under reduced pressure, and purified via flash chromatography to give 3-methylindolin-2-one (**2**) (90%, 1.026g, 6.935 mmol) as an off-white solid.

Gram-Scale Procedure for Spirooxdindole Formation with Purified *Cp*VBPO to Produce 4: An enzyme aliquot of purified VHPO from *Corallina pilulifera* (*Cp*VBPO, 10 µM, 7.25 mL) was removed from a -80°C freezer and allowed to warm to room temperature over 15 min. The thawed aliquot was then combined with an aqueous solution of 50mM Na_3VO_4 (580 uL, 0.25 equiv) in a 15 mL falcon tube and stored at room temperature. To ensure mixing, the resulting solution was vortexed for 1 min and placed on the benchtop until further use. To a 2L Erlenmeyer flask, purified H_2O (912 mL), 500mM pH 5 citrate buffer (116 mL), and 100mM KBr (4.66 mL, 0.1 equiv) was added. Then, 1-(1,3,4,9-tetrahydro-2H-pyrido[3,4-b]indol-2-yl)ethan-1-one (**3**) (1.0000 g, 4.67 mmol, 1 equiv) was dissolved in DMF (116 mL) and added to the reaction mixture. The falcon tube containing *Cp*VBPO (0.0016 mol%, 62.5 nM) and Na_3VO_4 (0.25 equiv) was then added to the reaction mixture. A 10% stock of H_2O_2 (3.01 mL, 2 equiv) was then added to the reaction mixture and the reaction was allowed to shake at room temperature for 2 h using Infors HT Multitron Incubator Shaker at 250 rpm. After this time, the reaction mixture was diluted with EtOAc (300 mL) and transferred to a 2L separatory funnel. The aqueous layer was re-extracted with 3X 300 mL EtOAc and the combined organic layer was washed with saturated brine (3x 250 mL) to remove residual DMF. The combined organic layer was then dried over sodium sulfate (Na_2SO_4) for 10 minutes, concentrated under reduced pressure, and purified via flash chromatography to give 1'-acetylspiro[indoline-3,3'-pyrrolidin]-2-one **(4)** (956 mg, 4.15 mmol, 89.0 %) as a clear oil.

Highest Total Turnover Number for 3-methylindolin-2-one (2) Synthesis

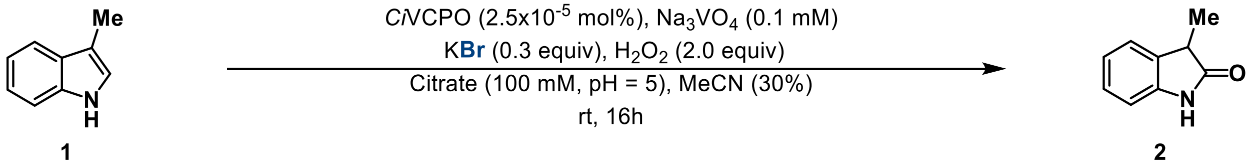

An enzyme aliquot of purified VHPO from *Curvularia inaequalis* (*Ci*VCPO, 10 µM, 20 µL) was removed from a -80 °C freezer and allowed to warm to room temperature over 5 min. The 10 µL of thawed aliquot was then combined with an aqueous solution of 50 mM Na_3VO_4 (200 µL, 0.025 equiv) in a microcentrifuge tube. To ensure mixing, the resulting solution was spun in the mini centrifuge for 10 seconds and placed on the benchtop until further use. To a 100 mL round bottom flask containing a magnetic stir bar, purified H_2O (49 mL), 500 mM pH 5 citrate buffer (20 mL) 100 mM KBr (1200 µL, 0.3 equiv), and 20 mL of MeCN was added. Then, the 3-methyl-1H-indole (**1**) (51.6 mg 0.40 mmol,1 equiv) was dissolved in MeCN (10 mL) and added to the reaction mixture. The microcentrifuge tube containing *Ci*VCPO (2.5×10^{-5} mol%, 1 nM) and Na_3VO_4 (0.025 equiv) was added to the reaction mixture. A 10% stock of H_2O_2 (264 µL, 2 equiv) was then added to the reaction mixture and the resulting solution was allowed to stir at room temperature for 16 h. The reaction mixture was then diluted with EtOAc (30 mL), saturated brine (15 mL) and H_2O (15 mL) (additional EtOAc and H_2O used to rinse flask as needed) and transferred to a 250 mL separatory funnel. The aqueous layer was extracted with EtOAc (2 x 30 mL) and the combined organic layers were then washed with H_2O/brine (2 x 30 mL) and dried over sodium sulfate (Na_2SO_4) for 10 minutes. The dried crude organic layer was concentrated under reduced pressure and purified via flash chromatography to give 3-methylindolin-2-one (**2**) (96%, 55.8 mg, 0.38 mmol) as an off-white solid.

Highest Total Turnover for 1'-acetylspiro[indoline-3,3'-pyrrolidin]-2-one (4) Synthesis

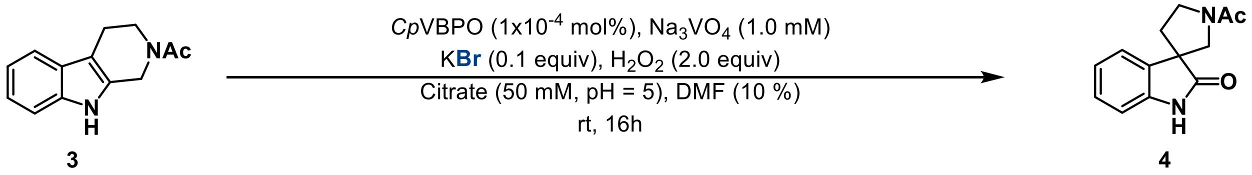

An enzyme aliquot of purified VHPO from *Corallina pilulifera* (*Cp*VBPO, 10 µM, 50 µL) was removed from a -80 °C freezer and allowed to warm to room temperature over 5 min. The 40 µL of thawed aliquot was then combined with an aqueous solution of 250 mM Na_3VO_4 (400 µL, 0.25 equiv) in a microcentrifuge tube. To ensure mixing, the resulting solution was spun in the mini centrifuge for 10 seconds and placed on the benchtop until further use. To a 100 mL round bottom flask containing a magnetic stir bar, purified H_2O (79 mL), 500 mM pH 5 citrate buffer (10 mL) and 100 mM KBr (400 µL, 0.1 equiv) was added. Then, the 1-(1,3,4,9-tetrahydro-2H-pyrido[3,4-b]indol-2-yl)ethan-1-one (**3**) (85.7 mg 0.40 mmol,1 equiv) was dissolved in DMF (10 mL) and added to the reaction mixture. The microcentrifuge tube containing *Cp*VBPO (1.0×10^{-5} mol%, 4 nM) and Na_3VO_4 (0.25 equiv) was added to the reaction mixture. A 10% stock of H_2O_2 (264 µL, 2 equiv) was then added to the reaction mixture and the resulting solution was allowed to stir at room temperature for 16 h. The reaction mixture was then diluted with EtOAc (50 mL) and saturated brine (50 mL) (additional EtOAc and H_2O used to rinse flask as needed) and transferred to a 250 mL separatory funnel. The aqueous layer was extracted with EtOAc (4 x 50 mL) and the combined organic layers were then washed with H_2O/brine (3 x 50 mL) and dried over sodium sulfate (Na_2SO_4) for 10 minutes. The dried crude organic layer was concentrated under reduced pressure and purified via flash chromatography to give 1'-acetylspiro[indoline-3,3'-pyrrolidin]-2-one (**4**) (93%, 86.9 mg, 0.38 mmol) as a clear oil.

Oxydeuteration Experiment

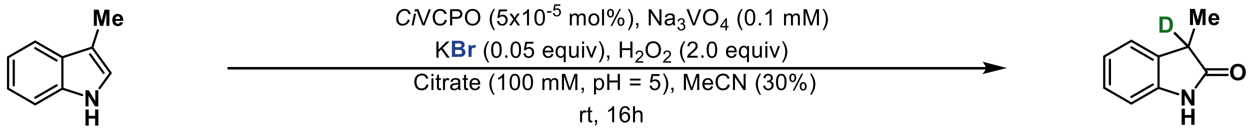

1 d-2

An enzyme aliquot of purified VHPO from *Curvularia inaequalis* (CiVCPO, 10 µM, 20 µL) was removed from a -80 °C freezer and allowed to warm to room temperature over 5 min. The 4 µL of thawed aliquot was then combined with an aqueous solution of 50 mM Na_3VO_4 (in D_2O, 40 µL, 0.025 equiv) in an Eppendorf tube and stored in room temperature. To ensure mixing, the resulting solution was spun in the mini centrifuge for 10 seconds and placed in the benchtop until further use. To a 50 mL round bottom flask containing a magnetic stir bar, D_2O (9.9 mL), 500 mM pH 5 citrate buffer (in D_2O, 4.0 mL), 50 mM KBr (in D_2O, 40 µL, 0.05 equiv), 4 mL of MeCN was added. Then, 3-methyl-1h-indole (1) (10.5 mg, 0.08 mmol,1 equiv) was dissolved in CD3CN (2.0 mL) and added to the reaction mixture. The Eppendorf tube containing the CiVCPO (0.00005 mol%, 2 nM) and Na_3VO_4 (0.025 equiv) was added to the reaction mixture. A 10% stock of H_2O_2 (in D_2O, 52.8 µL, 2 equiv) was then added to the reaction mixture and the reaction was allowed to stir at room temperature for 16h. After this time, the reaction mixture was diluted with ethyl acetate (5 mL), saturated brine (3 mL) and D_2O (3 mL) (additional ethyl acetate and D_2O used to rinse flask as needed) and transferred to a 125 mL separatory funnel. The aqueous layer was extracted with ethyl acetate (2 x 5 mL) and the combined organic layers are then washed with D_2O/Brine (2 x 5 mL) and dried over sodium sulfate for 10 minutes. The dried crude organic layer purified via flash chromatography to obtain 3-methylindolin-2-one (2) (11.0 mg, 0.08 mmol) in 93% yield.

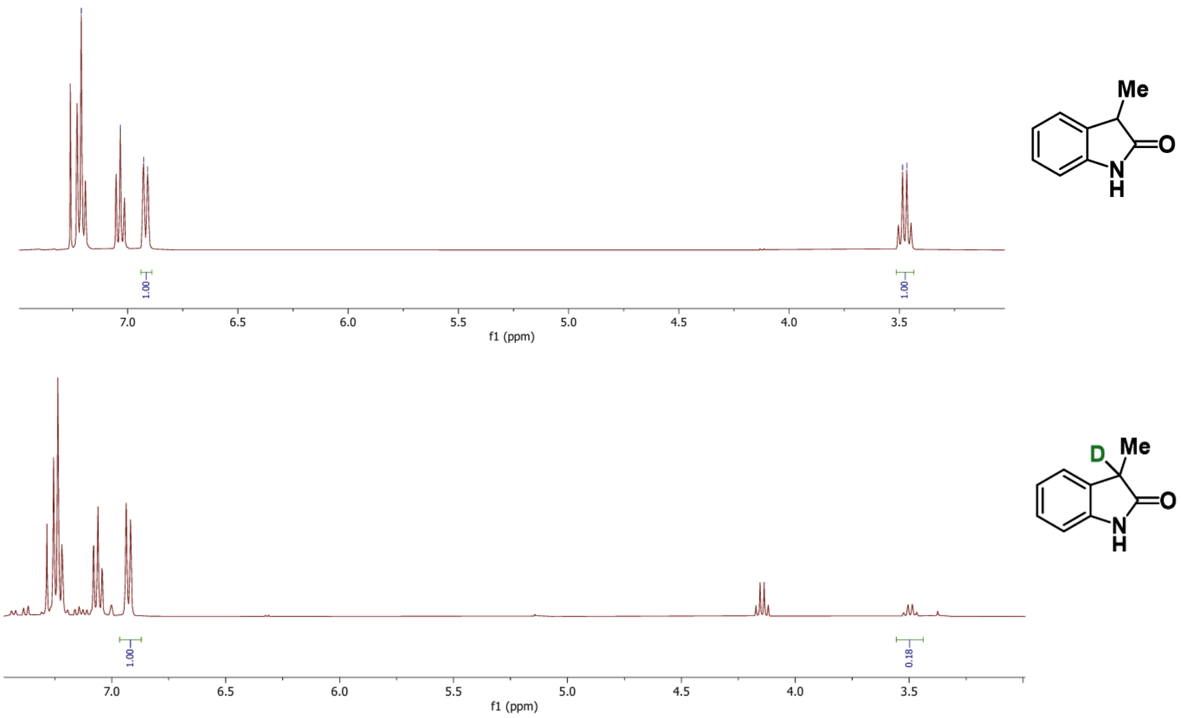

Figure S7. Deuterium Experiment

Biocatalytic Synthesis of 2-(2-oxoindolin-3-yl)acetic acid (33):

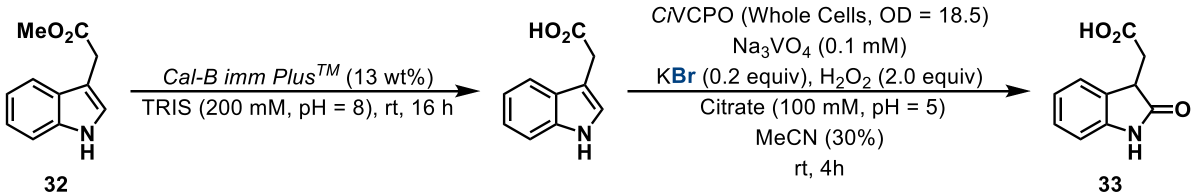

To a 20 mL scintillation vial, methyl 2-(1H-indol-3-yl)acetate (75.7 mg, 0.4 mmol) was added, followed by addition of 0.5 mL of MeCN, 3.5 mL of H_2O, 1.0 mL of 1M pH 8 Tris buffer, and Candida antarctica lipase B (CalB immo Plus^{TM}, 10.0 mg, 13 wt%). The reaction mixture was allowed to stir at room temperature for 16 h at 820 rpm on a shaker. Upon completion of reaction, mixture was acidified to pH 1 using 0.5 mL of 6 M HCl. Then, the reaction mixture was diluted with ethyl acetate (10 mL) and H_2O (10 mL) and filtered through a filter paper. Combined mixture was transferred to a 125 mL separatory funnel. The aqueous layer was extracted with ethyl acetate (2 x 10 mL) and the combined organic layers were then washed with H_2O/Brine (1 x 15 mL) and dried over sodium sulfate for 10 minutes. The resulting crude carboxylic acid was concentrated in a 100 mL round bottom flask under reduced pressure, which was then dissolved in 10 mL of MeCN. Two enzyme aliquots of VHPO whole cell from *Curvularia inaequalis* (*Ci*VCPO, OD600 = 18.5, 500 µL) was removed from a -80 °C freezer and allowed to warm to room temperature over 10 min. The thawed aliquots were then combined with an aqueous solution of 50 mM Na_3VO_4 (200 µL, 0.025 equiv) in a microcentrifuge tube and stored in room temperature. To ensure mixing, the resulting solution was spun in the mini centrifuge for 10 seconds and placed in the benchtop until further use. To a 100 mL round bottom flask containing crude carboxylic acid in MeCN and magnetic stir bar, purified H_2O (48 mL), 500 mM pH 5 citrate buffer (20 mL), 100 mM KBr (800 µL, 0.2 equiv), and 20 mL of MeCN was added. The aliquot containing the *Ci*VCPO and Na_3VO_4 was added to the reaction mixture. A 10% stock of H_2O_2 (264 µL, 2 equiv) was then added to the reaction mixture and the reaction was allowed to stir at room temperature for 4 h. After this time, the reaction mixture was divided equally into four 50 mL falcon tubes and mixed with 25 mL of EtOAc. The organic and aqueous layers were then separated via centrifugation. After removal of the organic layers, two additional rounds of extraction were performed with 200 mL of EtOAc and 50 mL of saturated brine each time. The combined organic layers were dried over Na_2SO_4 for 10 minutes, concentrated under reduced pressure, and dissolved in a minimal amount of EtOAc and silica. Then, it was concentrated again under reduced pressure to be dry loaded on to flash chromatography to give 2-(2-oxoindolin-3-yl)acetic acid (73.7 mg, 0.38 mmol) as off-white solid in 96% yield over two steps.

Yield: 96%

Purification: Eluted with 5% MeOH in DCM (R_f = 0.07)

^{1}H NMR (400 MHz, DMSO) δ. 12.35 (br s, 1H), 10.39 (s, 1H), 7.22 (d, J = 7.3 Hz, 1H), 7.16 (t, J = 7.6 Hz, 1H), 6.92 (t, J = 7.5 Hz, 1H), 6.81 (d, J = 7.5 Hz, 1H), 3.62 (t, J = 5.9 Hz, 1H), 2.90 (dd, J = 16.9, 4.7 Hz, 1H), 2.70 (m, 2H)

^{13}C NMR (101 MHz, DMSO) δ. 178.62, 172.67, 143.35, 129.85, 128.15, 124.06, 121.60, 109.61, 42.24, 34.43

These NMR spectra were consistent with the literature precedent.[18]

Chemoenzymatic Synthesis of methyl 2-phenylquinoline-4-carboxylate (35):

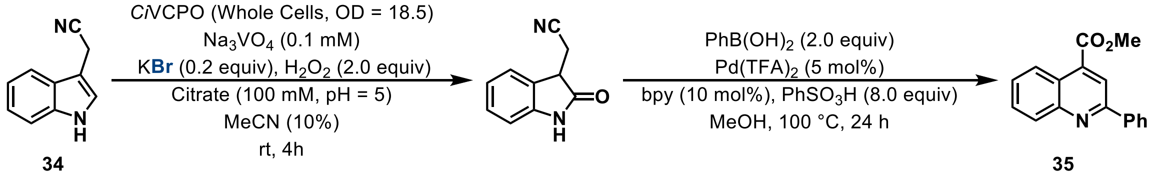

34 35

Two enzyme aliquots of VHPO whole cell from Curvularia inaequalis (*Ci*VCPO, OD600 = 18.5, 500 µL) was removed from a -80 °C freezer and allowed to warm to room temperature over 10 min. The thawed aliquots were then combined with an aqueous solution of 50 mM Na_3VO_4 (200 µL, 0.025 equiv) in a microcentrifuge tube and stored in room temperature. To ensure mixing, the resulting solution was spun in the mini centrifuge for 10 seconds and placed in the benchtop until further use. To a 100 mL round bottom flask containing a magnetic stir bar, purified H_2O (48 mL), 500 mM pH 5 citrate buffer (20 mL) and 100 mM KBr (800 µL, 0.2 equiv) was added. Then, 2-(1H-indol-3-yl)acetonitrile (0.40 mmol,1 equiv) was dissolved in MeCN (10 mL) and added to the reaction mixture. The aliquot containing the *Ci*VCPO and Na_3VO_4 was added to the reaction mixture. A 10% stock of H_2O_2 (264 µL, 2 equiv) was then added to the reaction mixture and the reaction was allowed to stir at room temperature for 4 h. After this time, the reaction mixture was divided equally into four 50 mL falcon tubes and mixed with 25 mL of EtOAc. The organic and aqueous layers were then separated via centrifugation. After removal of the organic layers, two additional rounds of extraction were performed with 200 mL of EtOAc and 50 mL of saturated brine each time. Combined organic layers were dried over sodium sulfate for 10 minutes. The resulting oxindole was concentrated into a 6-dram vial, then was added magnetic stir bar, MeOH (2 mL), benzene sulfonic acid (634 mg, 3.6 mmol, 8 equiv), phenyl boronic acid (110 mg, 0.09 mmol, 2 equiv), bipyridine (7 mg, 0.04 mmol, 0.1 equiv), $Pd(TFA)_2$ (8 mg, 0.02 mmol, 0.05 equiv) following the procedure adapted from Zhao and co-workers[19]. Then, the reaction was sealed with electrical tape and allowed to stir at 100 °C for 24 h at 1000 rpm. After the reaction time, saturated $NaHCO_3$ (5 mL) and ethyl acetate (10 mL) was added to the vial, which was then filtered through filter paper. The filtrate was extracted with ethyl acetate (3 × 30 mL) using a 125 mL separatory funnel, then combined organic layers were dried over sodium sulfate for 10 minutes, concentrated under reduced pressure, and dissolved in a minimal amount of EtOAc and silica. It was then concentrated again under reduced pressure to be dry loaded on to flash chromatography to give methyl 2-phenylquinoline-4-carboxylate (67.2 mg, 0.26 mmol) as light-yellow solid in 78% yield over two steps.

Yield: 78%

Purification: Eluted with 20% EtOAc in Hexane (R_f = 0.43)

^{1}H NMR (400 MHz, $CDCl_3$) δ. 8.76 (dd, J = 8.6, 1.4 Hz, 1H), 8.42 (s, 1H), 8.22 (td, J = 8.3, 1.3 Hz, 1H), 7.78 (ddd, J = 8.4, 6.8, 1.4 Hz, 1H), 7.64 (ddd, J = 8.3, 6.8, 1.3 Hz, 1H), 7.52 (m, 1H), 4.08 (s, 3H)

^{13}C NMR (101 MHz, $CDCl_3$) δ. 166.88, 156.75, 149.29, 138.81, 135.62, 130.35, 129.95, 129.77, 128.98, 127.85, 127.49, 125.44, 124.01, 120.39, 52.79

These NMR spectra were consistent with the literature precedent.[19]

Halide Divergent Experiment for Synthesis of methyl (5'S)-2-oxospiro[indoline-3,3'-pyrrolidine]-5'-carboxylate (37)

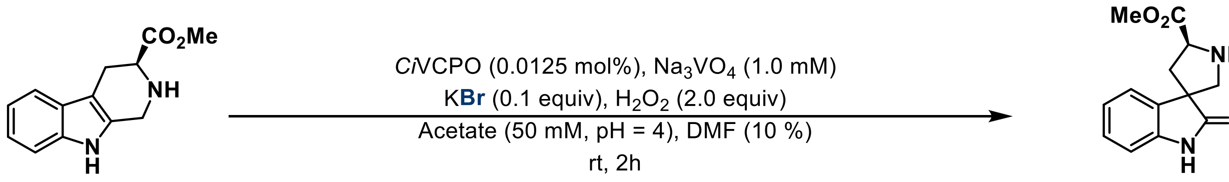

36 **37**

An enzyme aliquot of purified VHPO from *Curvularia inaequalis* (*Ci*VCPO, 10 µM, 5.0 mL) was removed from a -80 °C freezer and allowed to warm to room temperature over 15 min. The thawed aliquot was then combined with an aqueous solution of 250 mM Na_3VO_4 (400 µL, 0.25 equiv) in a 15 mL falcon tube. To ensure mixing, the resulting solution was vortexed for 1 min and placed on the benchtop until further use. To a 100 mL round bottom flask containing a magnetic stir bar, purified H_2O (79 mL), 500 mM pH 4 acetate buffer (10 mL) and 100 mM KBr (400 µL, 0.1 equiv) was added. Then, the methyl (S)-2,3,4,9-tetrahydro-1H-pyrido[3,4-b]indole-3-carboxylate (**36**) (92.0 mg 0.40 mmol,1 equiv) was dissolved in DMF (10 mL) and added to the reaction mixture. The falcon tube containing *Ci*VCPO (0.0125 mol%, 500 nM) and Na_3VO_4 (0.25 equiv) was added to the reaction mixture. A 10% stock of H_2O_2 (264 µL, 2 equiv) was then added to the reaction mixture and the resulting solution was allowed to stir at room temperature for 2 h. The reaction mixture was then diluted with EtOAc (50 mL) and transferred to a 250 mL separatory funnel. The organic layer was separated, and the aqueous layer was extracted with EtOAc (3 x 50 mL). The combined organic layers were then washed with saturated brine (3 x 50 mL) and dried over sodium sulfate (Na_2SO_4) for 10 minutes. The dried crude organic layer was concentrated under reduced pressure and purified via flash chromatography to give methyl (5'S)-2-oxospiro[indoline-3,3'-pyrrolidine]-5'-carboxylate **(37)** (76%, 75.0 mg, 0.30 mmol) as a thick yellow oil.

Yield: 76%

Purification: Eluted with the gradient of 0-10 % MeOH in DCM (R_f = 0.35)

^{1}H NMR (600 MHz, $CDCl_3$) δ 8.10 (d, J = 11.6 Hz, 1H), 7.31 (ddt, J = 8.2, 1.3, 0.7 Hz, 1H), 7.25 (tdd, J = 7.7, 4.2, 1.2 Hz, 1H), 7.09 (tdd, J = 7.6, 3.2, 1.0 Hz, 1H), 6.93 (ddt, J = 7.7, 1.6, 0.7 Hz, 1H), 4.37 – 4.15 (m, 1H), 3.83 (d, J = 5.3 Hz, 3H), 3.60 – 3.02 (m, 2H), 2.72 (dd, J = 13.6, 9.1 Hz, 1H), 2.54 – 2.43 (m, 1H), 2.37 – 2.29 (m, 1H).

^{13}C NMR (151 MHz, $CDCl_3$) δ 181.92, 181.67, 174.66, 173.53, 140.26, 140.19, 132.96, 132.46, 128.23, 128.17, 123.07, 123.05, 122.97, 122.87, 109.78, 109.73, 61.44, 61.08, 58.94, 58.50, 55.21, 54.46, 41.69, 41.37. (peaks for both diastereomers reported)

HRMS: calculated for $C_{13}H_{15}N_2O_3$ $[M+H^+]^+$: 247.1077. Found $[M+H^+]^+$: 247.1078.

IR: (cm^{-1}) 3195, 2950, 1701, 1618, 1470, 1176, 745.

$[\alpha]^{20}_D$ = +17.50 (c = 0.500, MeOH)

Halide Divergent Experiment for Synthesis of methyl 9H-pyrido[3,4-b]indole-3-carboxylate (38)

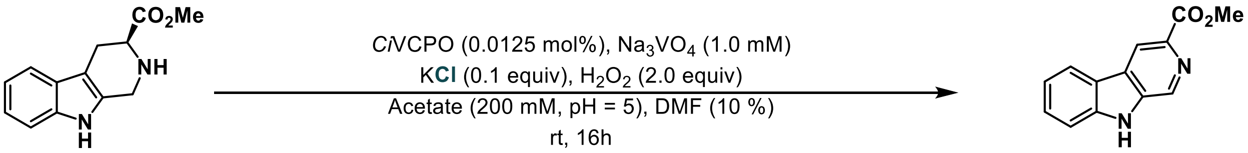

36 38

An enzyme aliquot of purified VHPO from *Curvularia inaequalis* (*Ci*VCPO, 10 µM, 5.0 mL) was removed from a -80 °C freezer and allowed to warm to room temperature over 15 min. The thawed aliquot was then combined with an aqueous solution of 250 mM Na_3VO_4 (400 µL, 0.25 equiv) in a 15 mL falcon tube. To ensure mixing, the resulting solution was vortexed for 1 min and placed on the benchtop until further use. To a 100 mL round bottom flask containing a magnetic stir bar, purified H_2O (79 mL), 500 mM pH 5 acetate buffer (20 mL) and 100 mM KCl (400 µL, 0.1 equiv) was added. Then, the methyl (S)-2,3,4,9-tetrahydro-1H-pyrido[3,4-b]indole-3-carboxylate (**36**) (92.0 mg 0.40 mmol,1 equiv) was dissolved in DMF (10 mL) and added to the reaction mixture. The falcon tube containing *Ci*VCPO (0.0125 mol%, 500 nM) and Na_3VO_4 (0.25 equiv) was added to the reaction mixture. A 10% stock of H_2O_2 (264 µL, 2 equiv) was then added to the reaction mixture and the resulting solution was allowed to stir at room temperature for 2 h. The reaction mixture was then diluted with EtOAc (50 mL) and transferred to a 250 mL separatory funnel. The organic layer was separated, and the aqueous layer was extracted with EtOAc (3 x 50 mL). The combined organic layers were then washed with saturated brine (3 x 50 mL) and dried over sodium sulfate (Na_2SO_4) for 10 minutes. The dried crude organic layer was concentrated under reduced pressure and purified via flash chromatography to give methyl 9H-pyrido[3,4-b]indole-3-carboxylate (**38**) (71%, 64.0 mg, 0.28 mmol) as a fine white solid.

Yield: 71%

Purification: Eluted with the gradient of 0-20% Acetone in DCM ($R_f = 0.2$)

^{1}H NMR (400 MHz, DMSO) δ 12.08 (s, 1H), 8.96 (dd, *J* = 15.5, 1.0 Hz, 2H), 8.42 (dt, *J* = 7.9, 1.0 Hz, 1H), 7.68 (dt, *J* = 8.2, 1.0 Hz, 1H), 7.61 (ddd, *J* = 8.2, 7.0, 1.2 Hz, 1H), 7.33 (ddd, *J* = 8.0, 7.0, 1.1 Hz, 1H), 3.92 (s, 3H).

^{13}C NMR (101 MHz, DMSO) δ 166.50, 141.42, 137.90, 136.94, 134.22, 129.18, 127.95, 122.72, 121.36, 120.71, 118.12, 112.87, 52.43.

HRMS: calculated for $C_{13}H_{11}N_2O_2$ $[M+H^+]^+$: 227.0815. Found $[M+H^+]^+$: 227.0813.

IR: (cm^{-1}) 3243, 2945, 1707, 1619, 1341, 1248, 1100, 728.

Analysis Data for Tryptophan-Selective Oxidation of Peptides

Purification

Purification of peptide starting materials was performed using high performance liquid chromatography (HPLC) on an Agilent 1100 series HPLC equipped with a C-18 reverse phase column with a particle size of 5 µm. All separations involved a mobile phase of water (solvent A) and acetonitrile (solvent B). The HPLC method used a linear gradient of 0- 80% solvent B over 30 minutes at ambient temperature with a flow rate of 1 mL/min . The eluent was monitored by absorbance at 220 nm.

Analytical HPLC

Analytical HPLC chromatography (HPLC) was performed on an Agilent 1100 series HPLC equipped with a 4.6 x 150 mm RediSep Prep C^{18} Aq, 100 A, 5 µm column. The reaction was monitored by analytical reverse phase HPLC using a gradient of water versus acetonitrile in linear gradients with a constant flow rate of 1 mL/min. Separations involved a mobile phase of 0.1% formic acid in water (solvent A) and 0.1 % formic acid in acetonitrile (solvent B) or mobile phase of water (solvent A) and acetonitrile (solvent B). The eluent was monitored with a detection wavelength of 220 nm.

HPLC Method A: Gradient: 0 to 80 % B (0.1% formic acid in ACN) in 30 min; 80-100 % B in 31-35 min at a flow rate of 1 mL/min.

LC/MS

High resolution LC-MS conditions for all purified peptides: Analyses were performed on an ultraperformance LC system (ACQUITY, Waters Corp., USA) coupled with a quadrupole 3 time-of-flight mass spectrometer (Q-ToF Premier, Waters) with electrospray ionization (ESI) in positive mode using Mass lynx software (V4.1) or high-performance LC system (Agilent, 1100 series) coupled with triple quadrupole.

LC-MS (Agilent technologies 6460) with electrospray ionization (ESI) in positive mode using Agilent mass hunter (10.0). Unless otherwise mentioned a sample was injected either onto a C4 column (Phenomenex Aeris™ 3.6 µm WIDEPORE C^4 200 Å, LC Column 50 x 2.1 mm) with a 400 µL/min flow rate of mobile phase of solution A (90 % H_2O, 10 % acetonitrile and 0.1 % formic acid (FA)) and solution B (95 % acetonitrile, 5 % H_2O, and 0.1 % formic acid) beginning gradient-Time- 0 min 10 % B; 5 min 28 % B; 20 min 38 % B; 22 min 90 % B; C18 column (ACQUITY UPLC BEH 1.7 µm 1x 50 mm) with a 200 µL/min flow rate of mobile phase of solution A (90 % H_2O, 10 % acetonitrile and 0.1 % formic acid) and solution B (90 % acetonitrile, 10 % H_2O, and 0.1 % formic acid) beginning gradient- Time- 1 min 0% B; 1-10 min 100% B for chromatography analysis (or) directly injected with mobile phase 90 % H_2O: 10 % ACN, 0.1% formic acid at 400 µL/min flow rate in ESI positive mode.

HRMS

High resolution MS data were acquired on Thermo Exactive Plus using a heated electrospray source. The solution was infused at a rate of 10-25 µL/min/electrospray using 3.3 KV. The typical settings were Capillary temp 320 ^{o}C. S-lens RF level was between 30-80 with an AGC setting of 1 E6. The maximum injection time as set to 50 ms. Spectra were taken at 140,000 resolutions at m/z 200 using Tune software and analyze with Thermo's Freestyle software.

Fmoc Solid-Phase Peptide Synthesis (Fmoc-SPPS)[20]

Peptides were synthesized manually on a 0.25 mm scale using Rink amide resin. Resin was swollen with DCM for 1 h at room temperature. Fmoc was deprotected using 20% piperidine–DMF for 5 min to obtain a deprotected peptide-resin. First, Fmoc protected amino acid (1.25 mm/5 equiv.) was coupled using HOAt (1.25 mm/5 equiv.) and DIC (1.25 mm/5 equiv.) in DMF for 15 min at room temperature. Fmoc-protected amino acids (0.75 mm/3 equiv.) were sequentially coupled on the resin using HBTU (0.75 mm/3 equiv.) and DIEA (1.5 mm/6 equiv.) in DMF for 5 min at room temperature. Peptides were synthesized using standard protocols. Peptides were cleaved from the resin using a cocktail of 95:5, trifluoroacetic acid: water for 2 h. The resin was removed by filtration and the resulting solution was concentrated. The residue was diluted with ACN/ H_2O mixture. The resulting solution was purified by HPLC.

General Analytical Scale Tryptophan-selective Oxidation of Peptides with Purified *Cp*VBPO

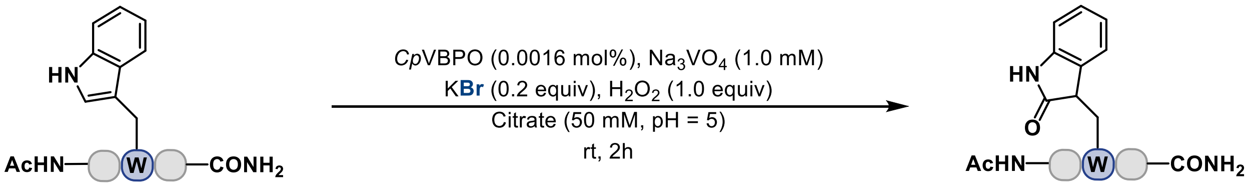

An enzyme aliquot of purified VHPO from *Corallina pilulifera* (*Cp*VBPO, 10 µM, 50 µL) was removed from a -80 °C freezer and allowed to warm to room temperature over 5 min. After thawing, 6.25 µL was transferred to a fresh PCR tube in addition to an aqueous solution of 250 mM Na_3VO_4 (4 µL, 0.25 equiv relative to starting material). To ensure mixing, the resulting solution was spun in the mini centrifuge for 10 seconds and placed in the benchtop until further use. A 1-dram vial was then charged with purified H_2O (725 µL), 500mM pH 5 citrate buffer (100 µL), and 100mM KBr (8 µL, 0.2 equiv). The 40 mM solution of the corresponding peptide in purified H_2O (100 µL, 1 equiv, 0.004 mmol starting material in reaction) was then added. The aliquot containing *Cp*VBPO (0.0016 mol%, 62.5 nM) and Na_3VO_4 (0.25 equiv) was added to the reaction mixture. Finally, a 10% stock of H_2O_2 (1.32 µL, 2 equiv) was added to the reaction mixture. The vial was capped and placed on a Heidolph Multireax Shaker set to 820 rpm at room temperature for 2h. After this time, the reaction mixture was diluted with MeCN (650 µL), transferred to a microcentrifuge tube, and spun in a Benchmark MC-24™ Touch Centrifuge at 13,000 rpm for 5 min. After centrifugation, 600 µL of the top layer of the reaction mixture was transferred to an LCMS vial then placed on an LCMS for analysis. The same vial was used to measure percent conversion on HPLC. A series of control experiments were then performed on the representative peptide Ac-WG-COOH (**31**) to confirm the necessity of all reaction components. These controls individually excluded the addition of *Cp*VBPO, Na_3VO_4, H_2O_2, and KBr.

.

Oxindole Ac-WG-COOH peptide (**31**)

Ac-WG-COOH peptide (SM-31). LCMS: *m/z* 304.1285 (calcd $[M+H]^+$ = 304.1292), *m/z* 607.2499 (calcd $[M*2+H]^+$ = 607.2511), (HPLC analysis at 220 nm). Retention time in HPLC: 8.4 min

HPLC Trace of Ac-WG-COOH peptide (SM-31) at 220 nm

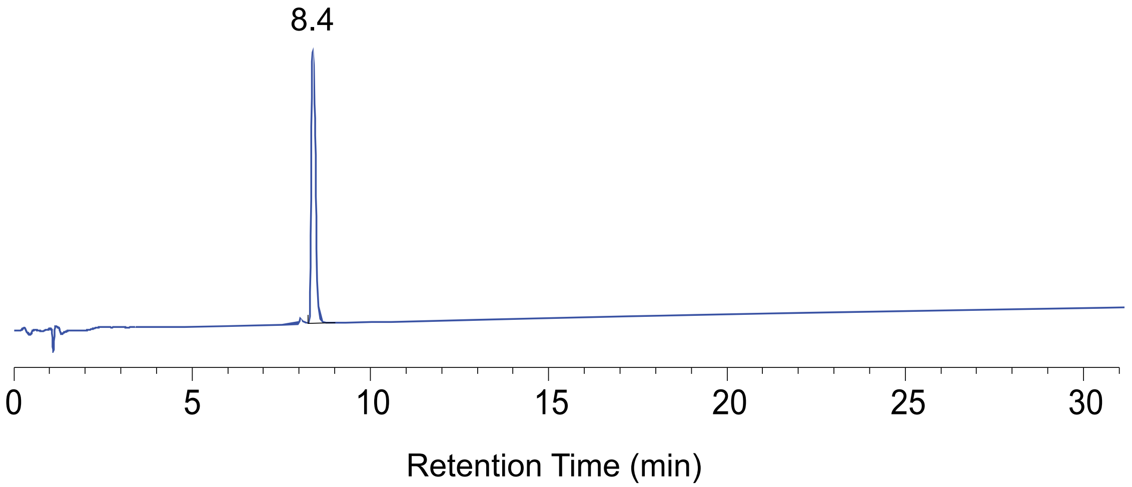

HRMS Trace of Ac-WG-COOH peptide (SM-31) Peak at 8.4 min

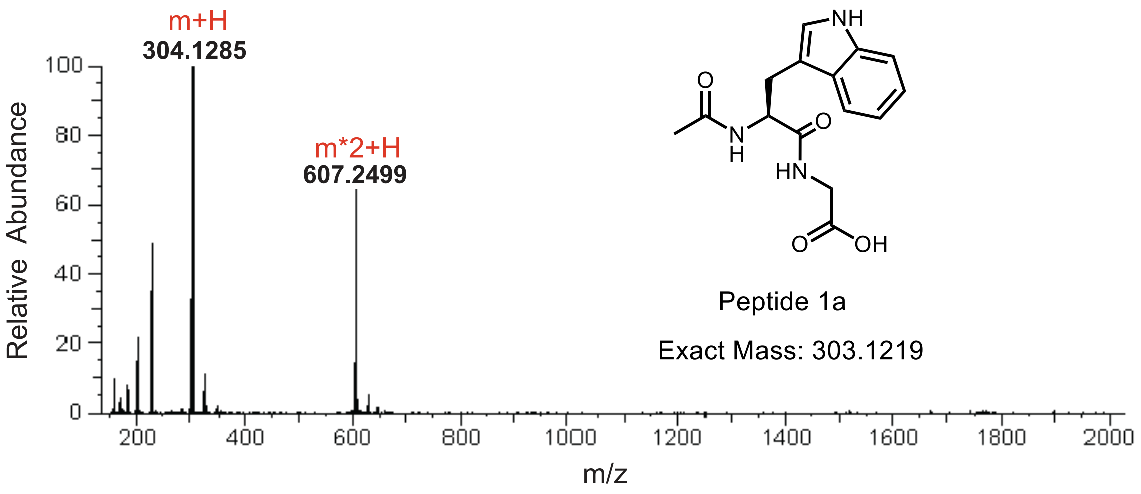

Ac-WG-COOH peptide in Standard Condition. LCMS: *m/z* 320.1233 and 320.1233 (calcd $[M+H]^+$ = 320.1241), *m/z* 639.2394 and 639.2393 (calcd $[M*2+H]^+$ = 639.2409) (HPLC analysis at 220 nm). Retention time in HPLC: 6.1 min and 6.5 min (> 95%)

HPLC Trace of Ac-WG-COOH peptide in Standard Condition at 220 nm

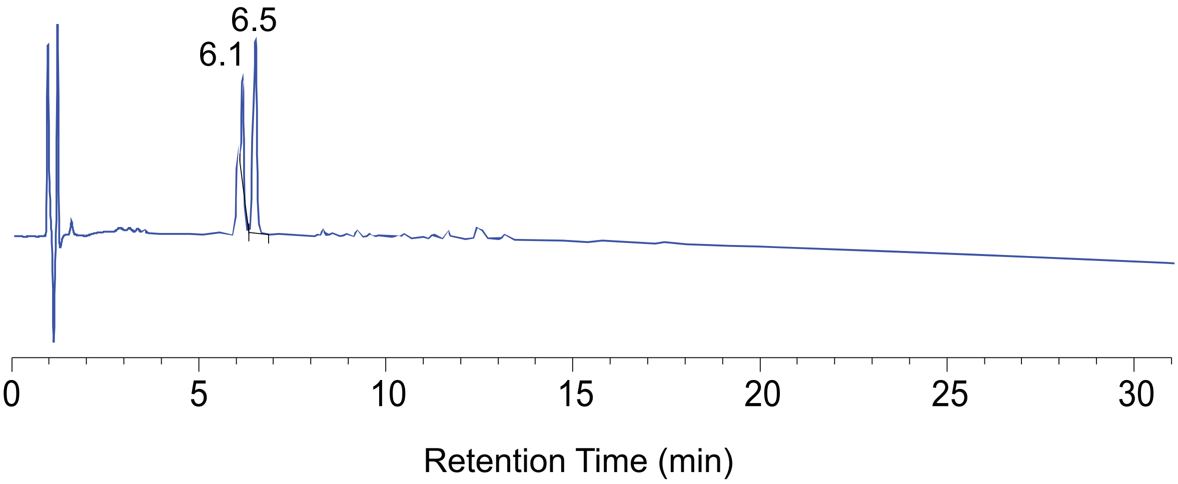

HRMS Trace of Ac-WG-COOH peptide in Standard Condition Peak at 6.1 min

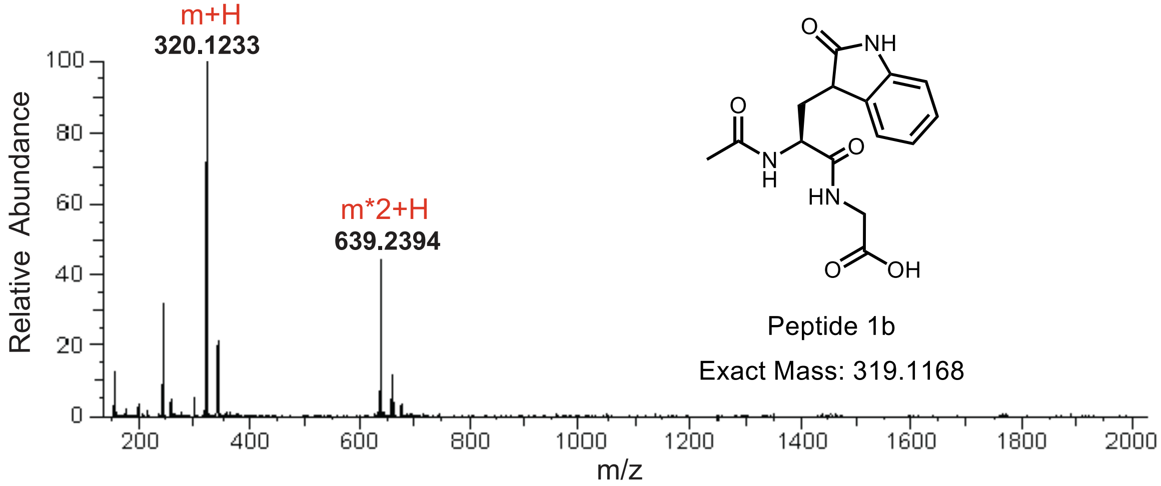

HRMS Trace of Ac-WG-COOH peptide in Standard Condition Peak at 6.5 min

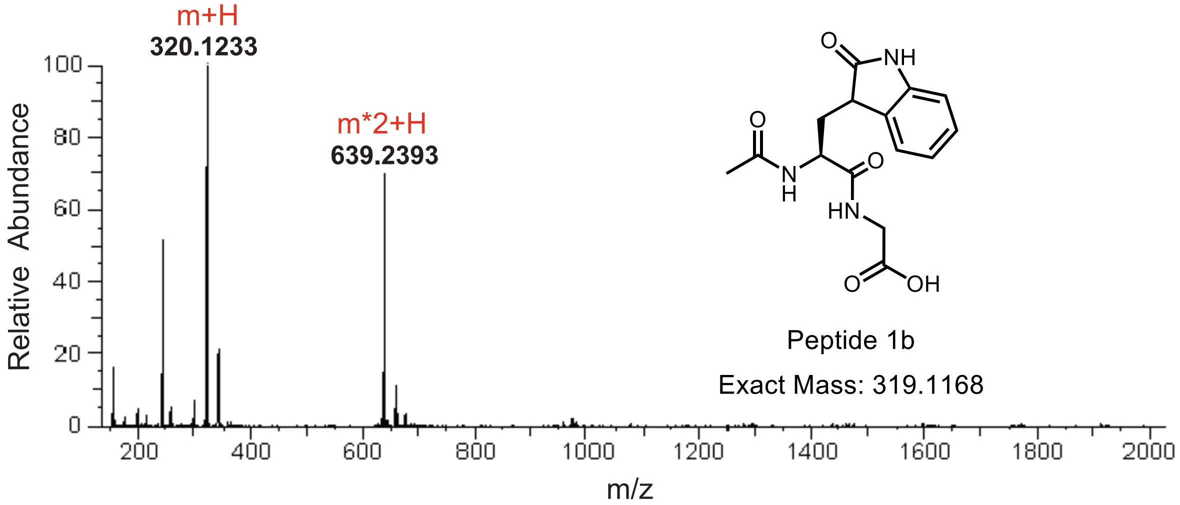

Ac-WG-COOH peptide in No *Cp*VBPO Condition. LCMS: *m/z* 304.1290 (calcd $[M+H]^+$ = 304.1292), *m/z* 607.2510 (calcd $[M*2+H]^+$ = 607.2511) (HPLC analysis at 220 nm). Retention time in HPLC: 8.4 min (no reaction)

HPLC Trace of Ac-WG-COOH peptide in no *Cp*VBPO Condition at 220 nm

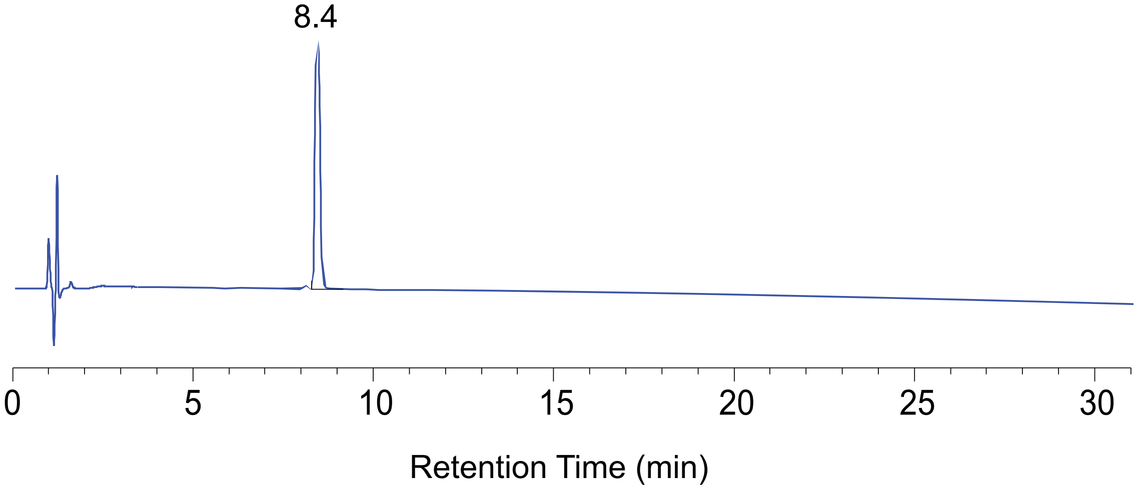

HRMS Trace of Ac-WG-COOH peptide in no *Cp*VBPO Condition Peak at 8.4 min

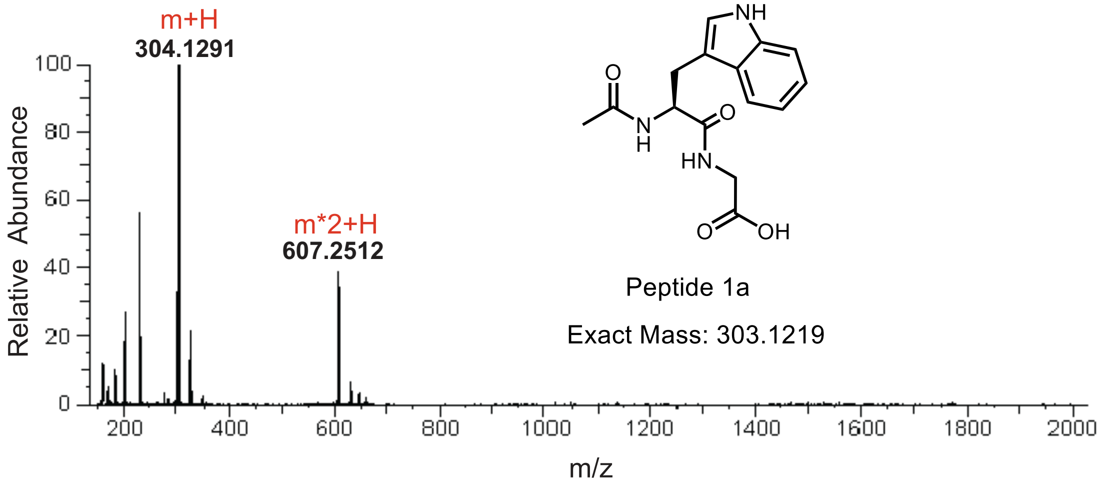

Ac-WG-COOH peptide in No Na_3VO_4 Condition. LCMS: *m/z* 304.1290 (calcd $[M+H]^+$ = 304.1292), *m/z* 607.2510 (calcd $[M*2+H]^+$ = 607.2511) (HPLC analysis at 220 nm). Retention time in HPLC: 8.4 min (no reaction)

HPLC Trace of Ac-WG-COOH peptide in no Na_3VO_4 Condition at 220 nm

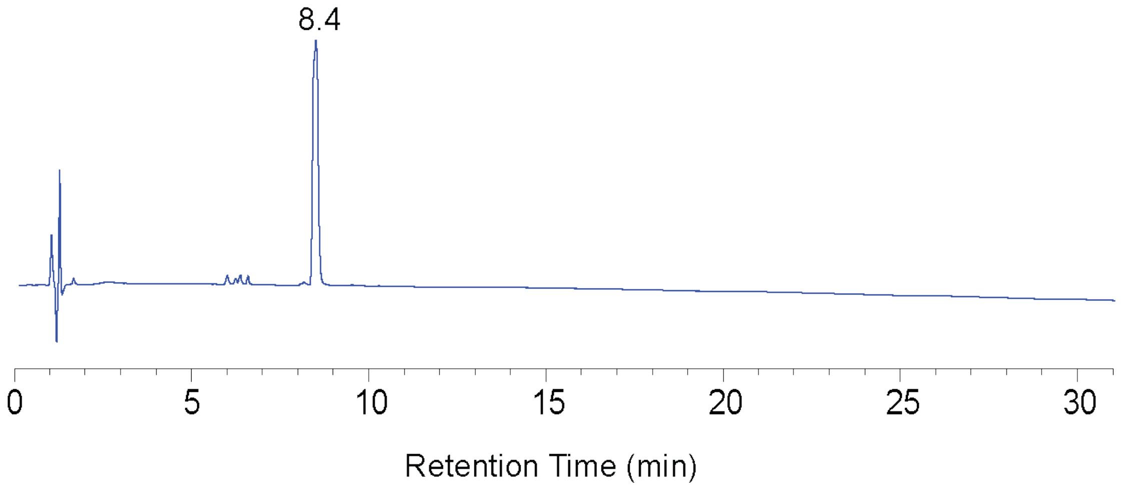

HRMS Trace of Ac-WG-COOH peptide in no Na_3VO_4 Condition Peak at 8.4 min

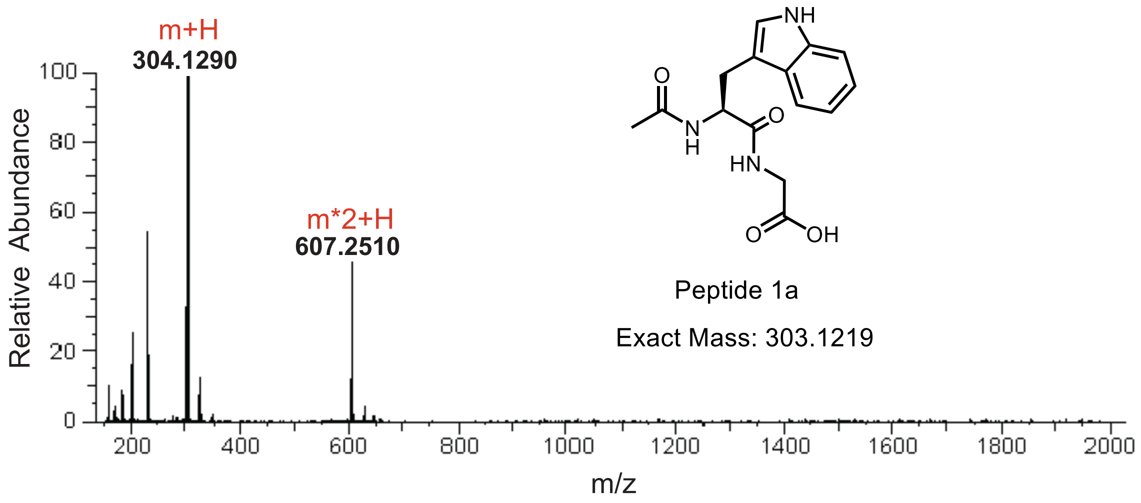

Ac-WG-COOH peptide in No KBr Condition. LCMS: *m/z* 304.1291 (calcd $[M+H]^+$ = 304.1292), *m/z* 607.2512 (calcd $[M*2+H]^+$ = 607.2511) (HPLC analysis at 220 nm). Retention time in HPLC: 8.4 min (no reaction)

HPLC Trace of Ac-WG-COOH peptide in no KBr Condition at 220 nm

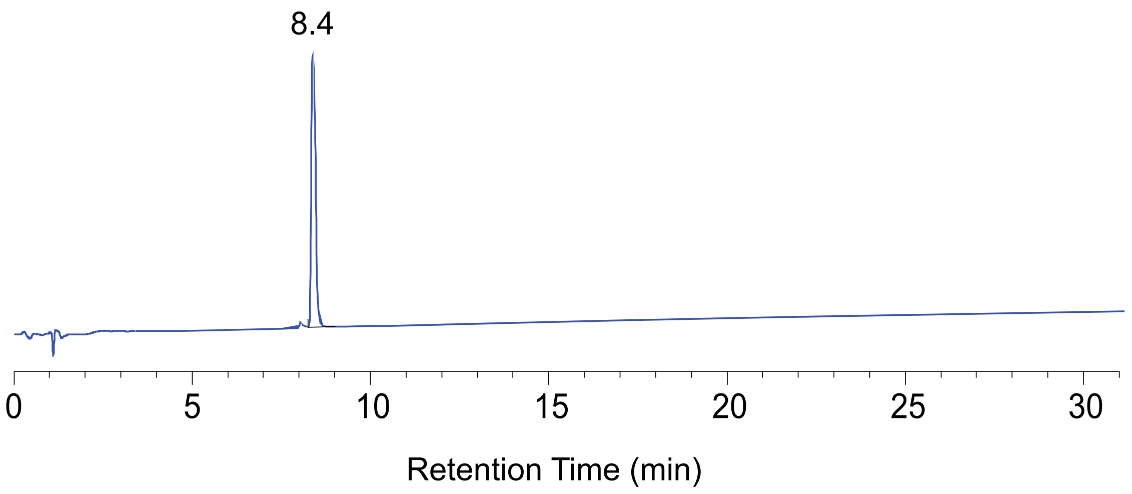

HRMS Trace of Ac-WG-COOH peptide in no KBr Condition Peak at 8.4 min

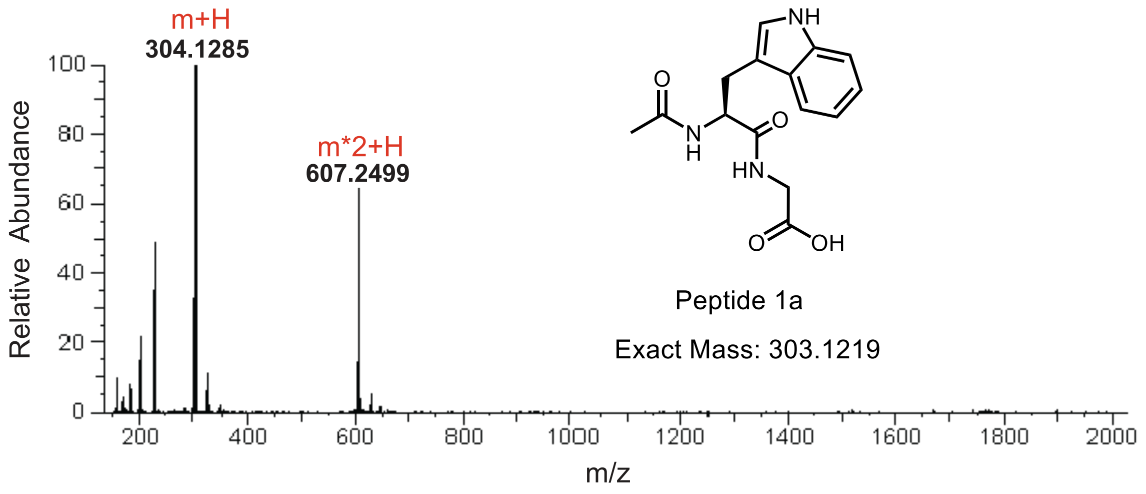

Ac-WG-COOH peptide in No H_2O_2 Condition. LCMS: *m/z* 304.1290 (calcd $[M+H]^+$ = 304.1292), *m/z* 607.2509 (calcd $[M*2+H]^+$ = 607.2511) (HPLC analysis at 220 nm). Retention time in HPLC: 8.4 min (no reaction)

HPLC Trace of Ac-WG-COOH peptide in no H_2O_2 Condition at 220 nm

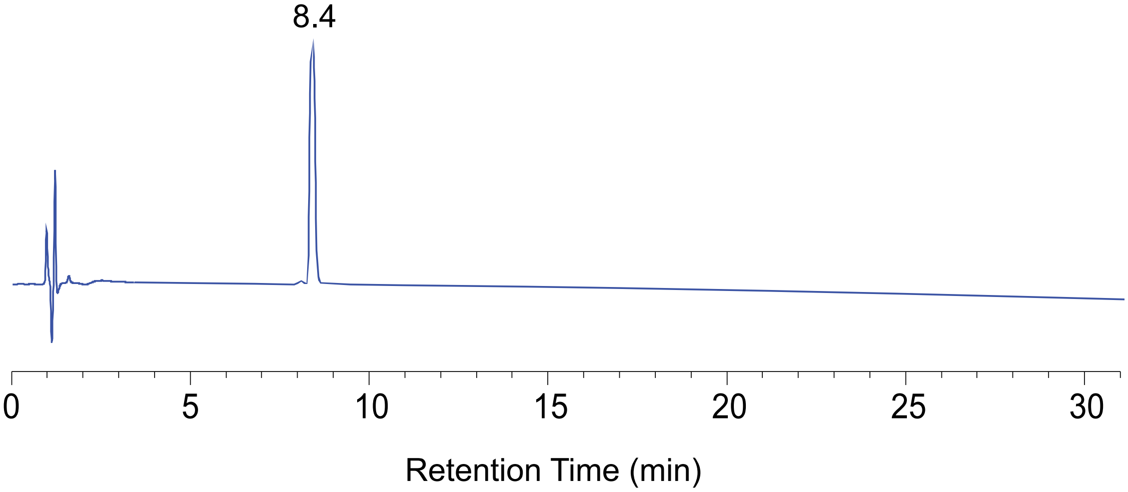

HRMS Trace of Ac-WG-COOH peptide in no H_2O_2 Condition Peak at 8.4 min

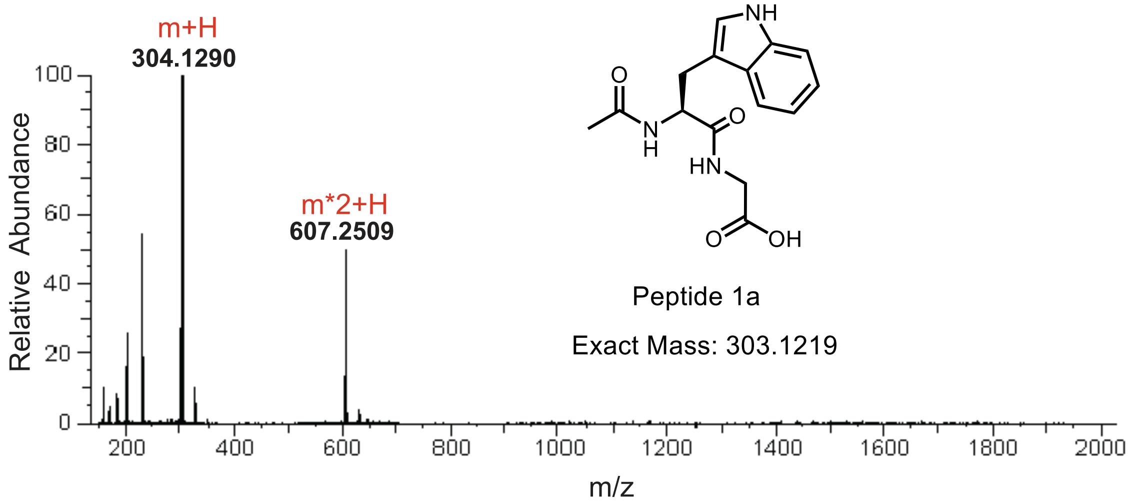

Oxindole Ac-WGY- $CONH_2$ peptide (39)

Ac-WGY- $CONH_2$ peptide (SM-39). LCMS: *m/z* 466.2085 (calcd $[M+H]^+$ = 466.2085), *m/z* 931.4101 (calcd $[M*2+H]^+$ = 931.4097), (HPLC analysis at 220 nm). Retention time in HPLC: 9.9 min

HPLC Trace of Ac-WGY- $CONH_2$ peptide (SM-39) at 220 nm

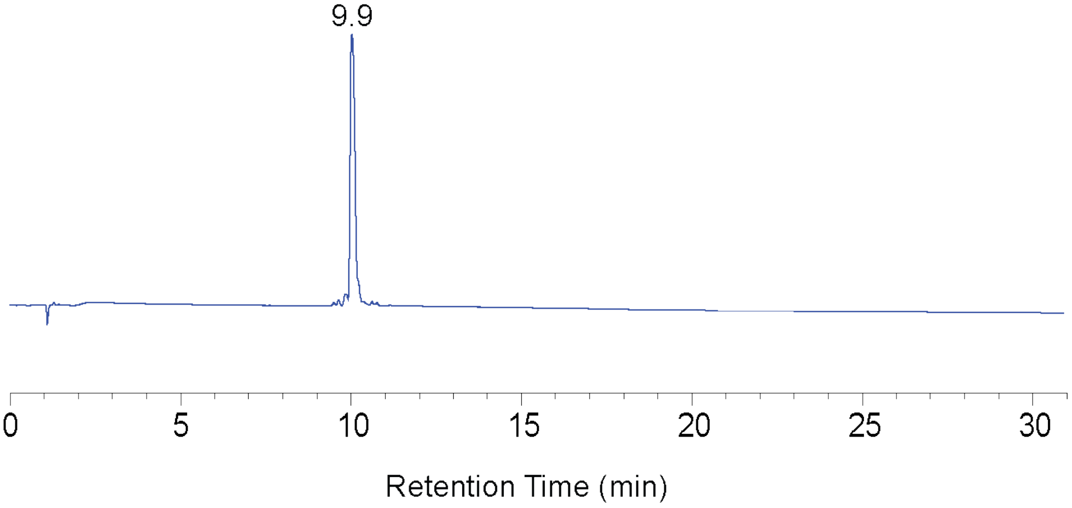

HRMS Trace of Ac-WGY- $CONH_2$ peptide (SM-39) at 9.9 min

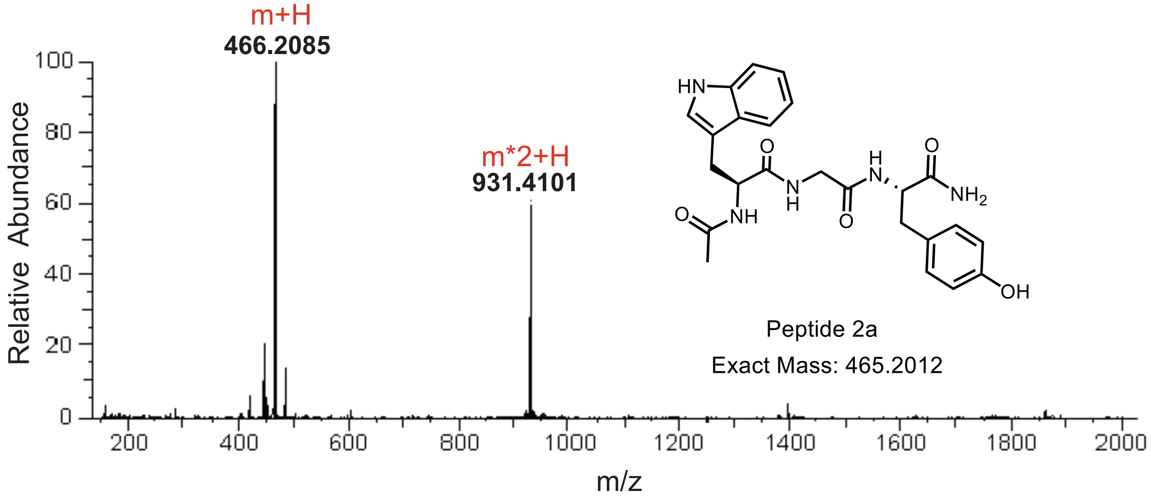

Ac-WGY- CONH2 peptide in Standard Condition. LCMS: *m/z* 482.2034 and 482.2033 (calcd $[M+H]^+$ = 482.2034), *m/z* 504.1853 (calcd $[M+Na]^+$ = 504.1859), LCMS: *m/z* 963.3998 (calcd $[M*2+H]^+$ = 963.3995) (HPLC analysis at 220 nm). Retention time in HPLC: 7.8 min and 8.1 min (> 95%)

HPLC Trace of Ac-WGY- $CONH_2$ peptide in Standard Condition at 220 nm

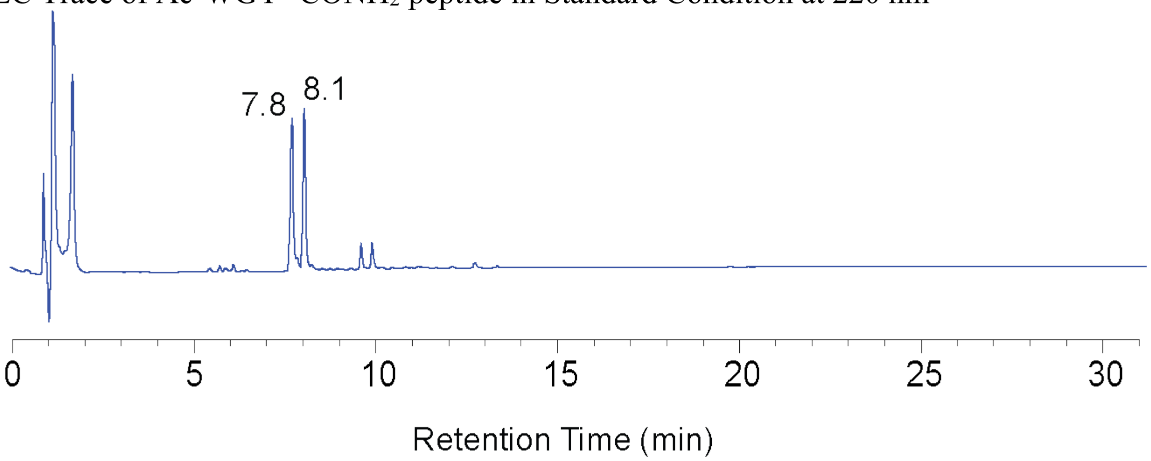

HRMS Trace of Ac-WGY- $CONH_2$ peptide in Standard Condition Peak at 7.8 min

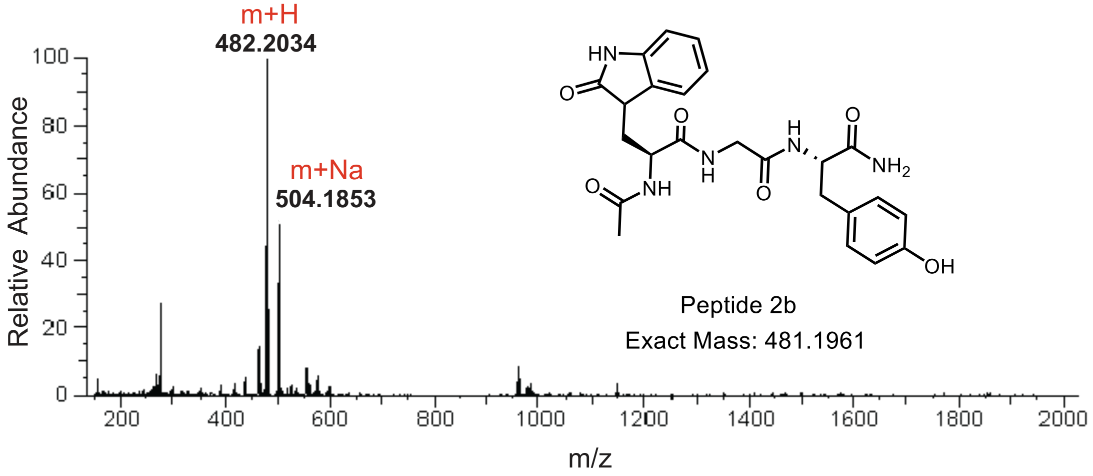

HRMS Trace of Ac-WGY- $CONH_2$ peptide in Standard Condition Peak at 8.1 min

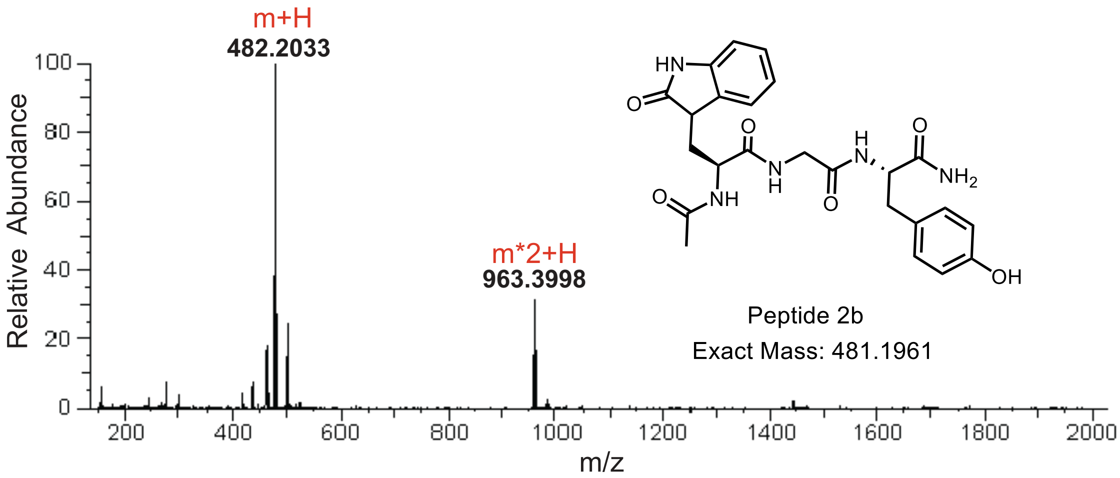

Oxindole Ac-WGH-$CONH_2$ peptide (40)

Ac-WGH-CONH₂ peptide (SM-40). LCMS: *m/z* 440.2038 (calcd $[M+H]^+$ = 440.2041) (HPLC analysis at 220 nm). Retention time in HPLC: 5.2 min

HPLC Trace of Ac-WGH-$CONH_2$ (SM-40) Peptide at 220 nm

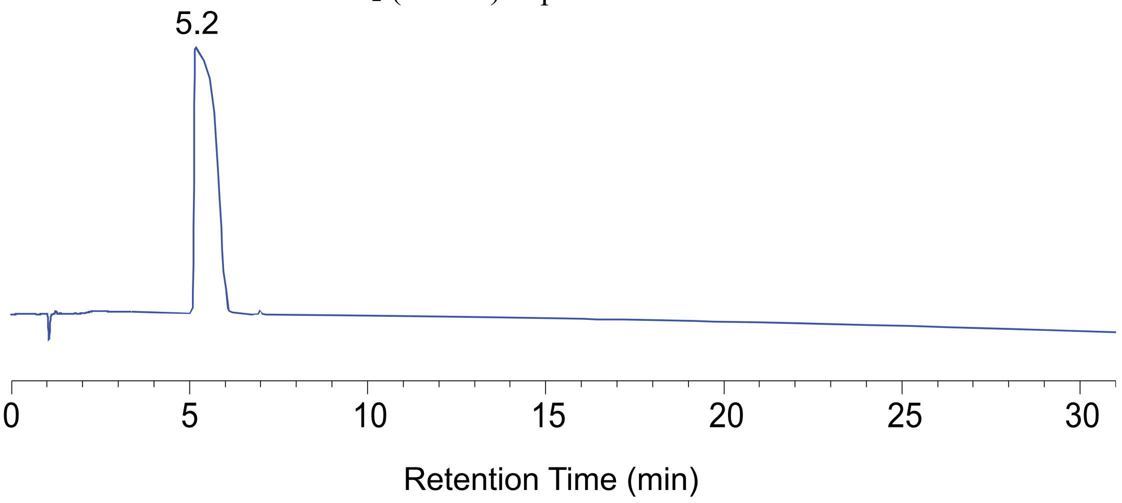

HRMS Trace of Ac-WGH-$CONH_2$ (SM-40) Peptide in Standard Condition Peak at 5.2 min

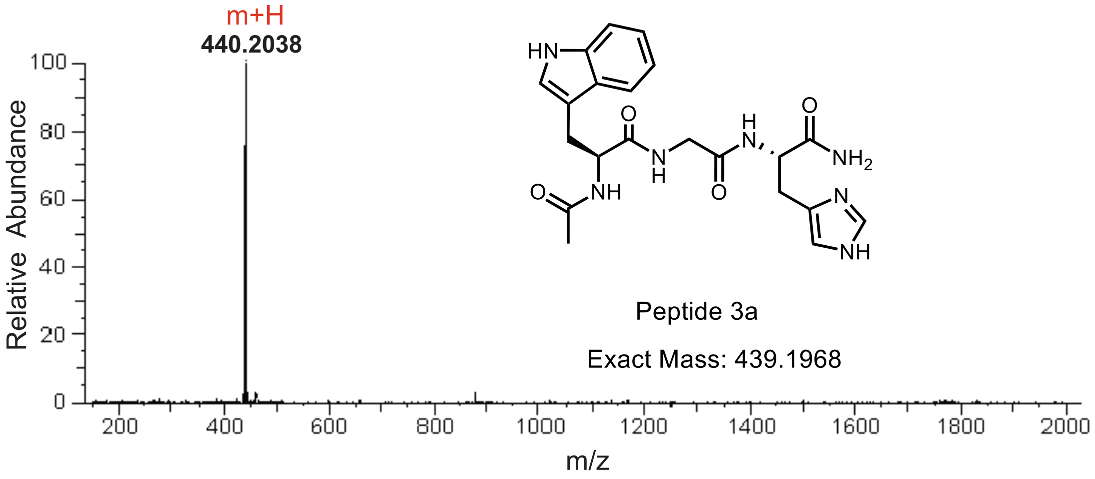

Ac-WGH-CONH$_2$ peptide in Standard Condition. LCMS: *m/z* 456.1986 and 456.1988 (calcd $[M+H]^+$ = 456.1990) (HPLC analysis at 220 nm). Retention time in HPLC: 4.3 min and 4.8 min (> 95%)

HPLC Trace of Ac-WGH-CONH$_2$ peptide in Standard Condition at 220 nm

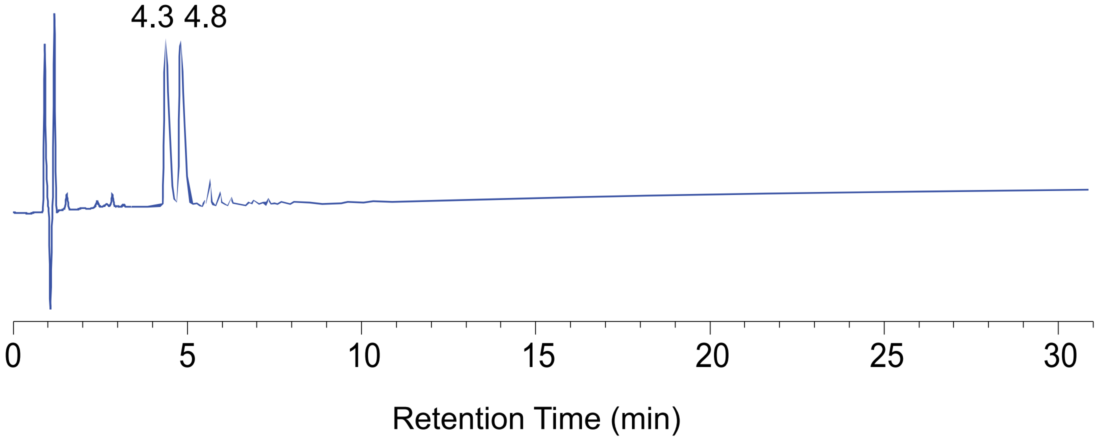

HRMS Trace of Ac-WGH-CONH$_2$ peptide Peak in Standard Condition at 4.3 min

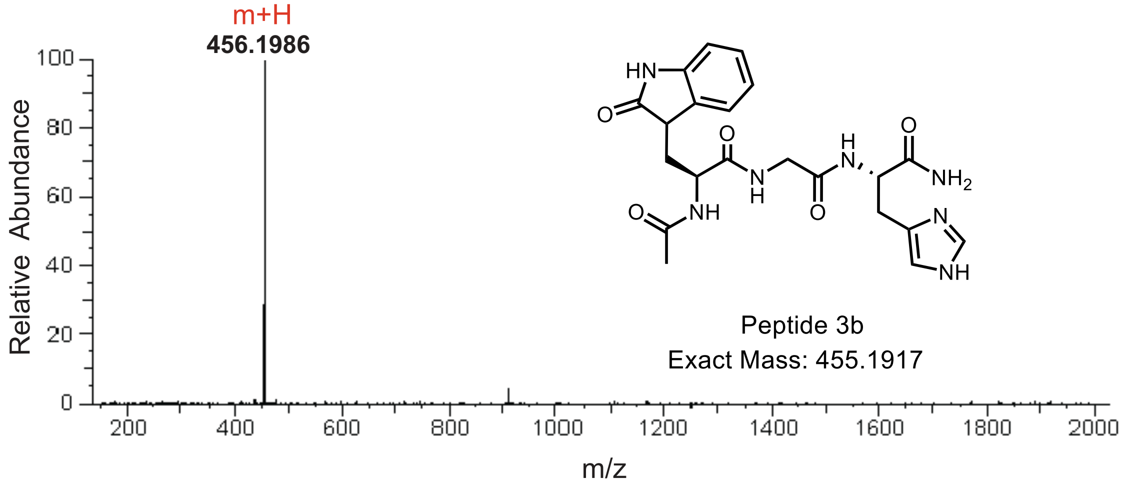

HRMS Trace of Ac-WGH-CONH$_2$ peptide Peak in Standard Condition Peak at 4.8 min

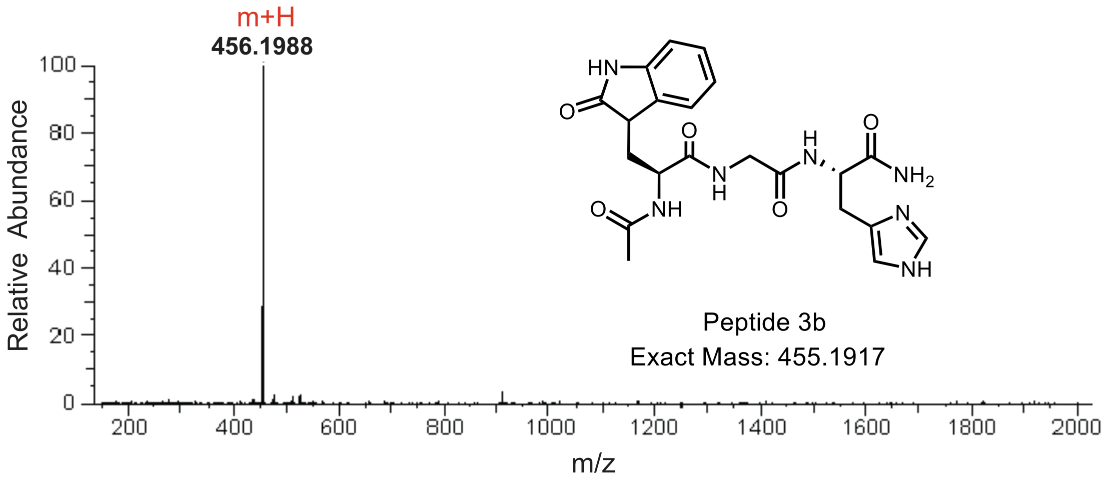

Oxindole Ac-FYKVPNW-CONH$_2$ peptide (41)

Ac-FYKVPNW-CONH$_2$ peptide (SM-41). LCMS: *m/z* 994.5142 (calcd [M+H]$^+$ = 994.5145) (HPLC analysis at 220 nm). Retention time in HPLC: 11.3 min

HPLC Trace of Ac-FYKVPNW-CONH$_2$ (SM-41) Peptide at 220 nm

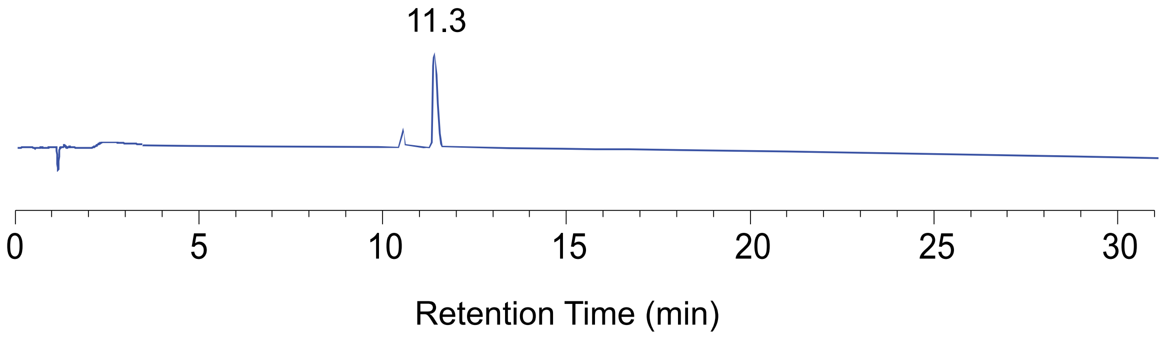

HRMS Trace of Ac-FYKVPNW-CONH$_2$ (SM-41) Peptide Peak at 11.3 min

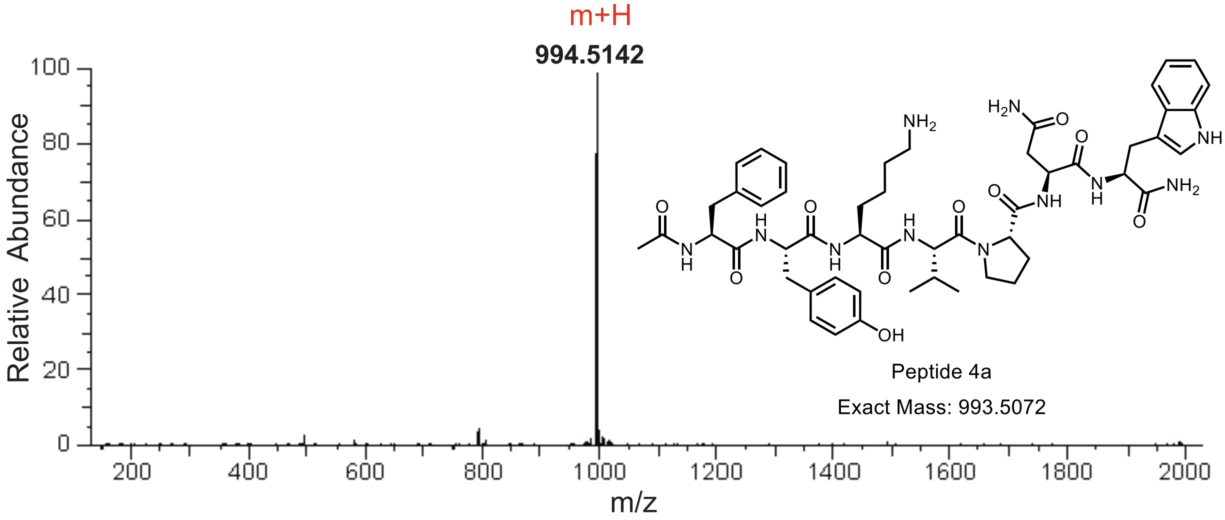

Ac-FYKVPNW-CONH2 peptide in Standard Condition. LCMS: *m/z* 1010.5070 and 1010.5073 (calcd $[M+H]^+$ = 1010.5094) (HPLC analysis at 220 nm). Retention time in HPLC: 10.2 min and 10.6 min (> 95%)

HPLC Trace of Ac-FYKVPNW-CONH2 peptide in Standard Condition at 220 nm

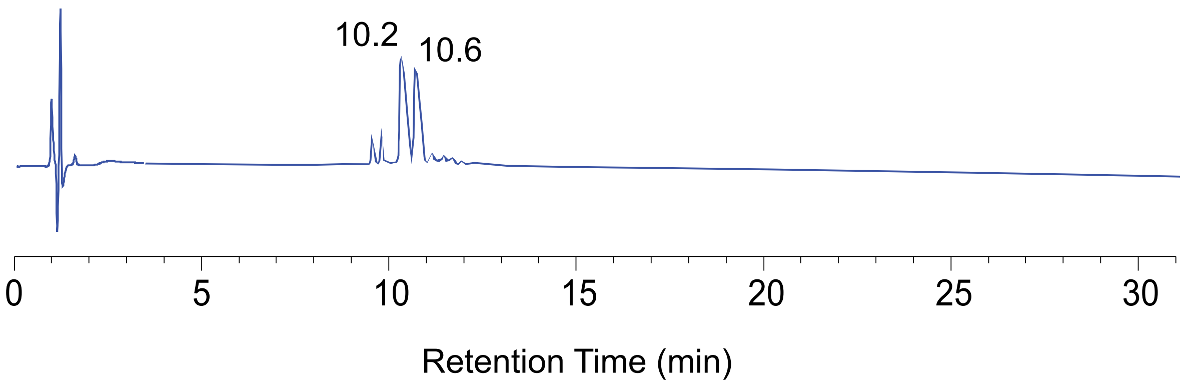

HRMS Trace of Ac-FYKVPNW-CONH2 peptide in Standard Condition Peak at 10.2 min

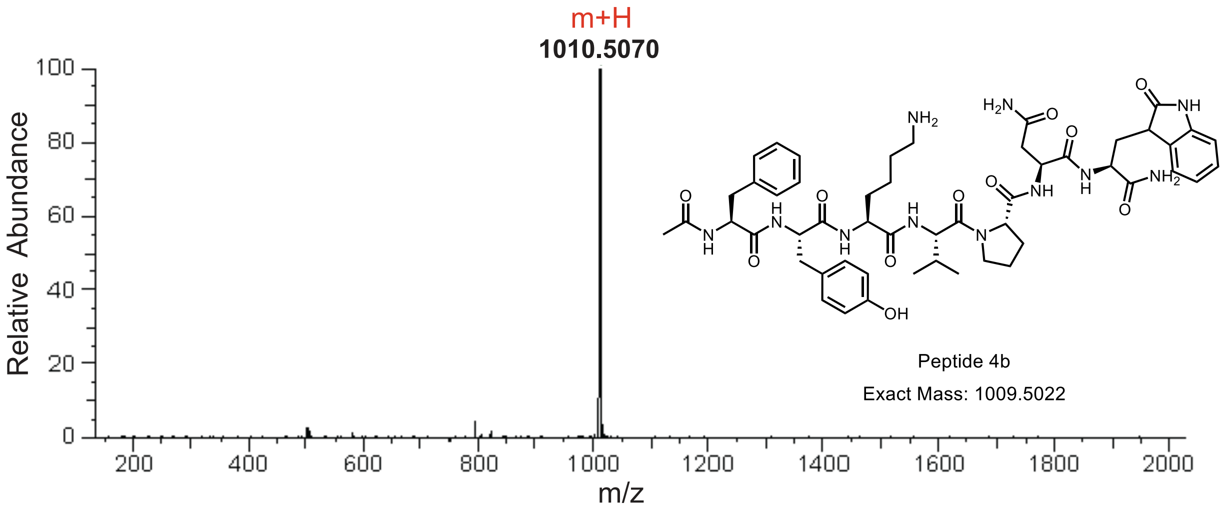

HRMS Trace of Ac-FYKVPNW-CONH$_2$ peptide in Standard Condition Peak at 10.6 min

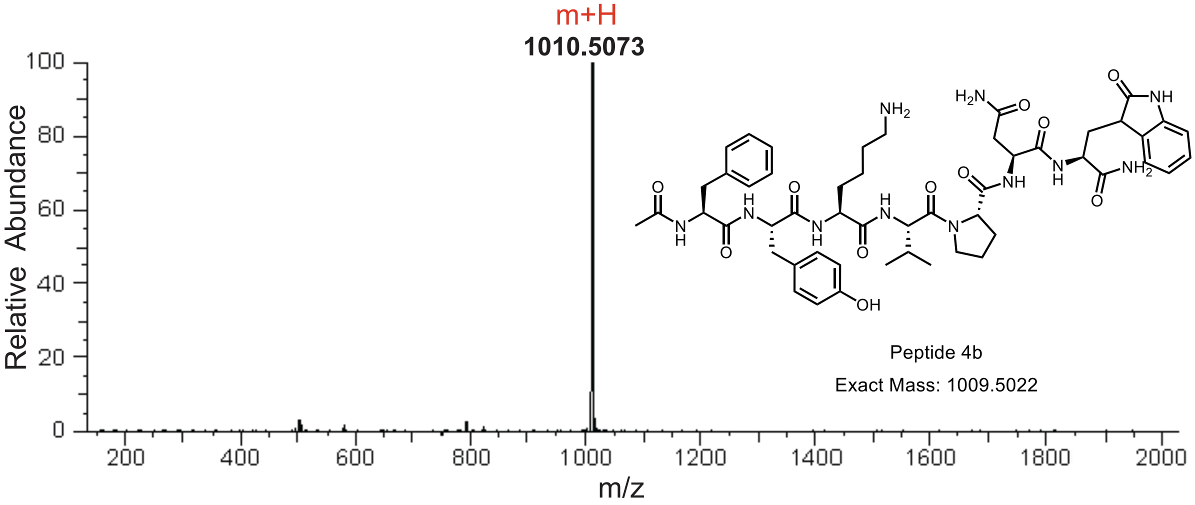

Oxindole Ac-KWGPGGPFR-CONH$_2$ peptide (42)

Ac-KWGPGGPFR-CONH$_2$ peptide (SM-42). LCMS: *m/z* 1042.6290 (calcd $[M+H]^+$ = 1042.5581), *m/z* 522.0375 (calcd $[M+2/2]^+$ = 521.7827), (HPLC analysis at 220 nm). Retention time in HPLC: 7.8 min

HPLC Trace of Ac-KWGPGGPFR-CONH$_2$ (SM-42) Peptide at 220 nm

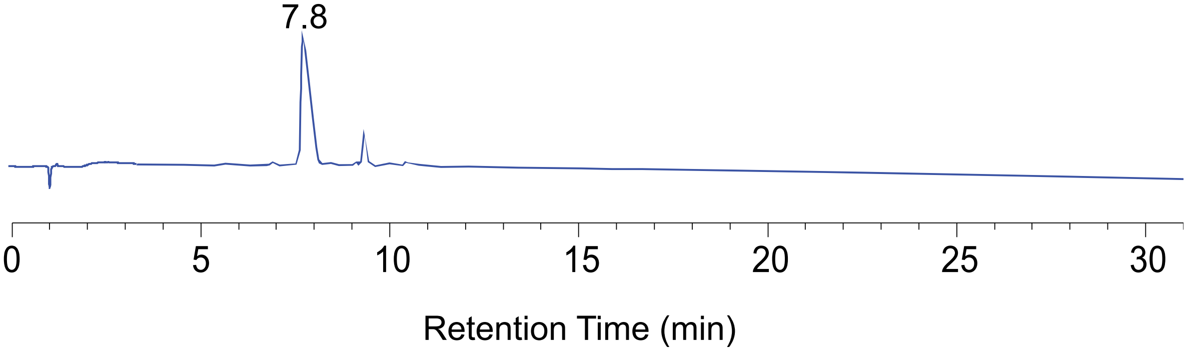

HRMS Trace of Ac-KWGPGGPFR-CONH$_2$ (SM-42) Peptide Peak at 7.8 min

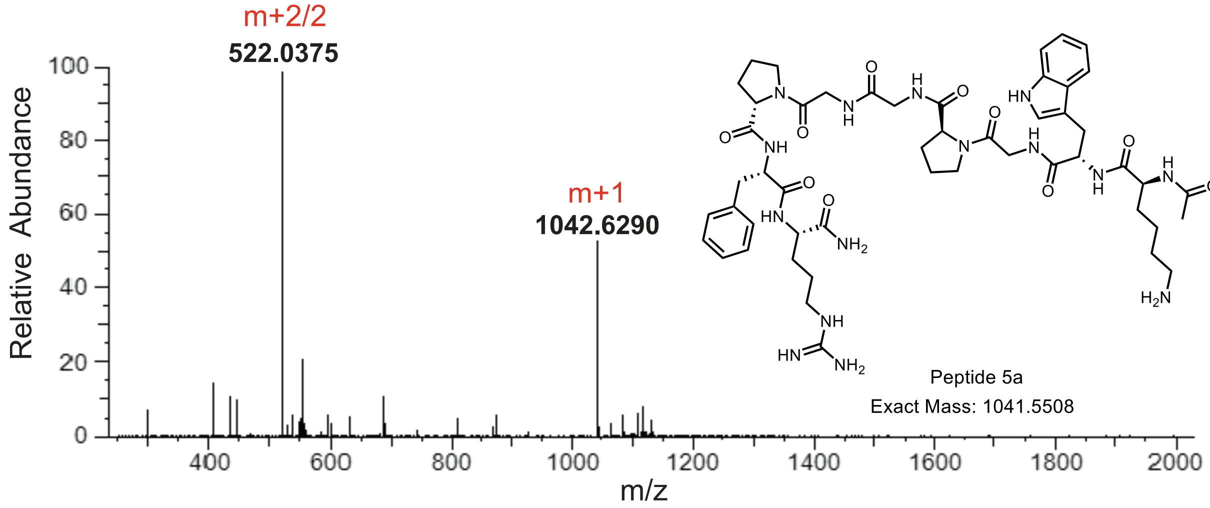

Ac-KWGPGGPFR-CONH$_2$ peptide in Standard Condition. LCMS: *m/z* 1058.5505 and 1058.5510 (calcd $[M+H]^+$ = 1058.5530), *m/z* 529.7790 and 529.7792 (calcd $[M+2/2]^+$ = 529.7802) (HPLC analysis at 220 nm). Retention time in HPLC: 7.2 min and 7.4 min (> 95%)

HPLC Trace of Ac-KWGPGGPFR-CONH$_2$ Peptide in Standard Condition at 220 nm

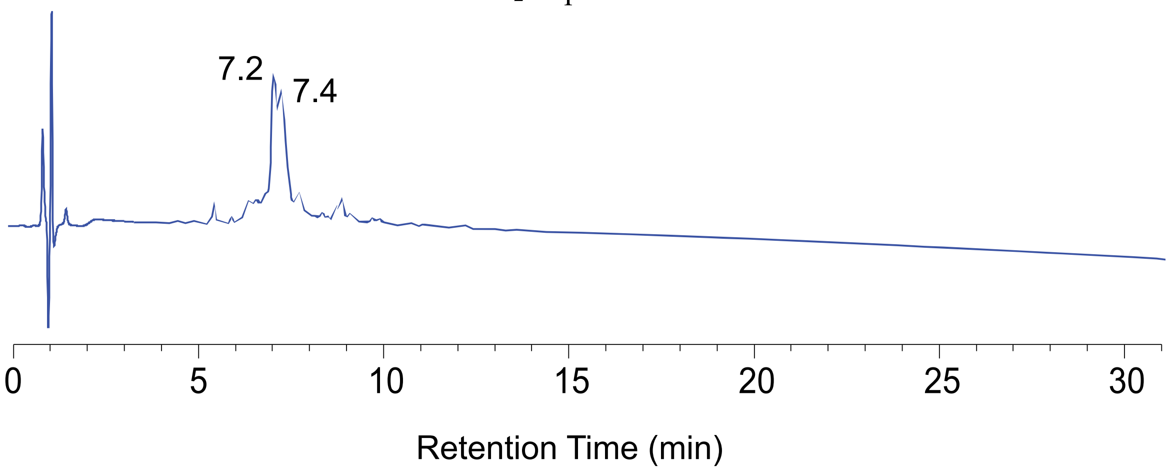

HRMS Trace of Ac-KWGPGGPFR-CONH$_2$ Peptide in Standard Condition Peak at 7.2 min

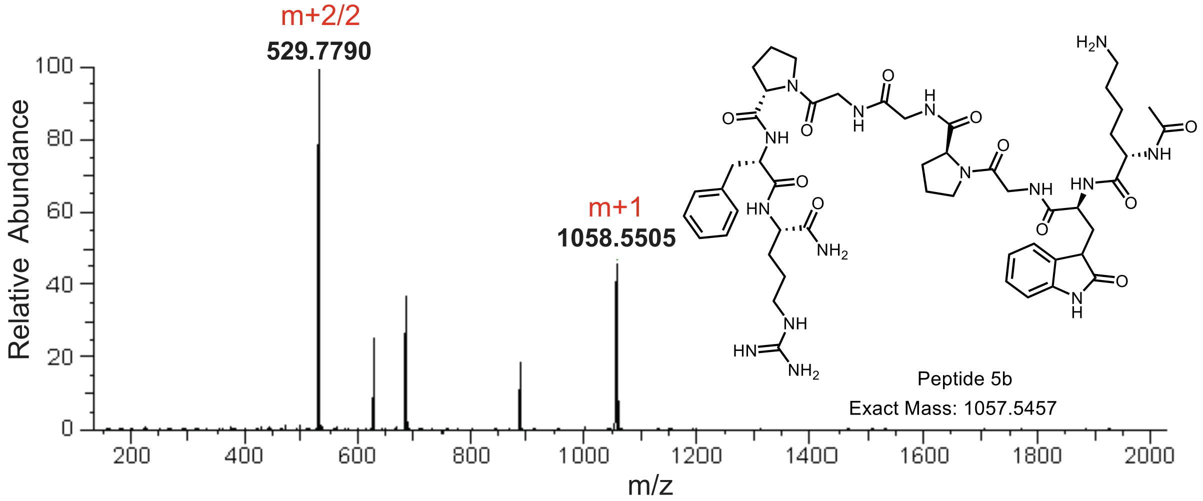

HRMS Trace of Ac-KWGPGGPFR-CONH$_2$ Peptide in Standard Condition Peak at 7.4 min

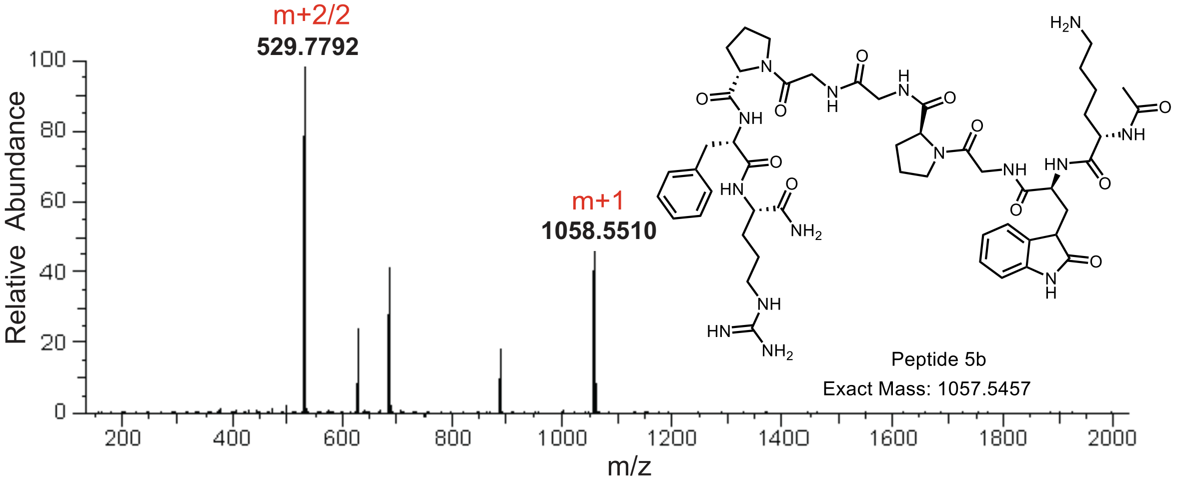

Gram Scale Synthesis of 1'-methylspiro[indoline-3,3'-pyrrolidin]-2-one (Coerulescine, 44):

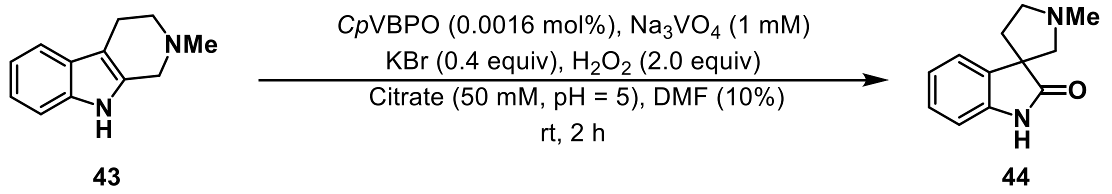

An enzyme aliquot of purified VHPO from *Corallina pilulifera* (*Cp*VBPO,10uM, 8.38 mL) was removed from a -80 °C freezer and allowed to warm to room temperature over 10 min. The thawed aliquot was then combined with an aqueous solution of 250 mM Na_3VO_4 (5.37 mL, 0.25 equiv) in a 15 mL falcon tube and stored at room temperature. To ensure mixing, the resulting solution was vortexed for 1 min and placed on the benchtop until further use. To a 2L Erlenmeyer flask, purified H_2O (1027 mL), 500 mM pH 5 citrate buffer (134 mL, 50 mM) and 100 mM KBr (21.5 mL, 0.4 equiv) was added. The 2-methyl-2,3,4,9-tetrahydro-1H-pyrido[3,4-b]indole (**43**) (1.002 g, 5.38 mmol) was dissolved in DMF (134 mL) and added to the reaction mixture. The falcon tube containing *Cp*VBPO (0.0016 mol%, 62.5 nM) and Na_3VO_4 (0.25 equiv) was added to the reaction mixture. A 10% stock of H_2O_2 (3.540 mL, 2 equiv) was then added to the reaction mixture and the reaction was allowed to shake at room temperature for 2 h using Infors HT Multitron Incubator Shaker at 220 rpm. After this time, the reaction mixture was extracted with EtOAc (5 × 150 mL) for every 500 mL of reaction mixture using 1L separatory funnel, and combined organic layer was concentrated down to 300 mL under reduced pressure. Then, this concentrated organic layer was washed with saturated brine (3 × 100 mL) to remove DMF. The resulting organic layer was concentrated again under the reduced pressure, dried over Na_2SO_4 for 10 minutes, concentrated under reduced pressure once more, and purified via flash chromatography to give 1'-methylspiro[indoline-3,3'-pyrrolidin]-2-one (**44**) (0.794 g, 3.90 mmol) as yellow oil in 73% yield.

Yield: 73%

Purification: Eluted with the gradient of 5 to 10% MeOH in DCM (R_f = 0.29)

^{1}H NMR (400 MHz, $CDCl_3$) δ. 9.32 (br s, 1H), 7.40 (d, J = 7.5 Hz, 1H), 7.18 (t, J = 7.7 Hz, 1H), 7.03 (t, J = 7.5 Hz, 1H), 6.91 (d, J = 7.7 Hz, 1H), 3.08 (m, J = 7.6 Hz, 1H), 2.94 (s, 2H), 2.89 (m, 1H), 2.51 (s, 3H), 2.41 (m, 1H), 2.15 (m, 1H).

^{13}C NMR (101 MHz, $CDCl_3$) δ. 183.10, 140.30, 135.47, 127.84, 123.27, 122.81, 109.72, 65.85, 56.65, 53.56, 41.75, 37.82

HRMS: calculated for $C_{12}H_{15}N_2O$ $[M+H^+]^+$: 203.1179. Found $[M+H^+]^+$: 203.1178.

IR: (cm^{-1}) 3201, 3055, 2916, 2850, 1660, 1606, 1308, 1150, 1034, 747.

Gram Scale Synthesis of 1'-methylspiro[indoline-3,3'-pyrrolidin]-2-one (Horsfiline, 46):

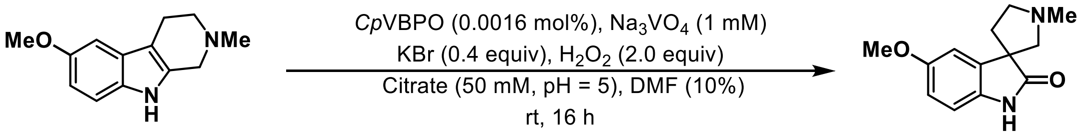

45 **46**

An enzyme aliquot of purified VHPO from *Corallina pilulifera* (*Cp*VBPO,10uM, 7.25 mL) was removed from a -80 °C freezer and allowed to warm to room temperature over 10 min. The thawed aliquot was then combined with an aqueous solution of 250 mM Na_3VO_4 (4.64 mL, 0.25 equiv) in a 15 mL falcon tube and stored at room temperature. To ensure mixing, the resulting solution was vortexed for 1 min and placed on the benchtop until further use. To a 2L Erlenmeyer flask, purified H_2O (910 mL), 500 mM pH 5 citrate buffer (115 mL, 50 mM) and 100 mM KBr (18.5 mL, 0.4 equiv) was added. The 6-methoxy-2-methyl-2,3,4,9-tetrahydro-1H-pyrido[3,4-b]indole (**45**) (1.000 g, 4.62 mmol) was dissolved in DMF (115 mL) and added to the reaction mixture. The falcon tube containing *Cp*VBPO (0.0016 mol%, 62.5 nM) and Na_3VO_4 (0.25 equiv) was added to the reaction mixture. A 10% stock of H_2O_2 (3.05 mL, 2 equiv) was then added to the reaction mixture and the reaction was allowed to shake at room temperature for 16 h using Infors HT Multitron Incubator Shaker at 220 rpm. After this time, reaction mixture was diluted with EtOAc (300 mL), and basified to pH = ~9.0 (measured via pH strip) with saturated NaHCO3, and transferred to a 2L separatory funnel. The aqueous layer was re-extracted 10X with additional EtOAc (300 mL) as needed, and the combined organic layer was washed with 100 mL of saturated brine 3 times to remove DMF. The resulting organic layer was dried over Na_2SO_4 for 10 minutes, concentrated under reduced pressure, and purified via flash chromatography to give 1'-methylspiro[indoline-3,3'-pyrrolidin]-2-one (**46**) (0.882 g, 4.62 mmol) as yellow oil in 82% yield.

Yield: 82%

Purification: Eluted with 1% MeOH in DCM with 1% NEt3 (R_f = 0.4)

^{1}H NMR (400 MHz, DMSO) δ 10.20 (s, 1H), 6.92 (t, *J* = 1.6 Hz, 1H), 6.73 (d, *J* = 1.5 Hz, 2H), 3.70 (s, 3H), 3.03 (td, *J* = 8.0, 3.9 Hz, 1H), 2.76 (d, *J* = 9.0 Hz, 1H), 2.58 (d, *J* = 9.0 Hz, 1H), 2.51 – 2.42 (m, 2H), 2.34 (s, 3H), 2.17 (ddd, *J* = 12.2, 8.0, 4.0 Hz, 1H), 1.91 (dt, *J* = 12.5, 7.7 Hz, 1H).

^{13}C NMR (101 MHz, DMSO) δ 181.23, 155.52, 138.24, 134.86, 112.56, 110.41, 109.90, 66.43, 56.56, 55.89, 53.90, 41.98, 37.79.

HRMS: calculated for $C_{13}H_{17}N_2O_2$ $[M+H^+]^+$: 233.1285. Found $[M+H^+]^+$: 233.1280.

IR: (cm^{-1}) 3161, 2960, 2900, 2781, 1697, 1600, 1477, 1034, 787.

Optimization Data

Oxidation of 3-Monosubstituted Indoles with *Ci*VCPO in Whole Cells Optimization Experiments: All optimization reactions were performed using the General Procedure C. The only variable changed was the one indicated in the Figures below. (*Note: 100 µL of a 8 mg/mL solution of 1,3,5-tribromobenzene was added as an internal standard for yield confirmation, where applicable)*

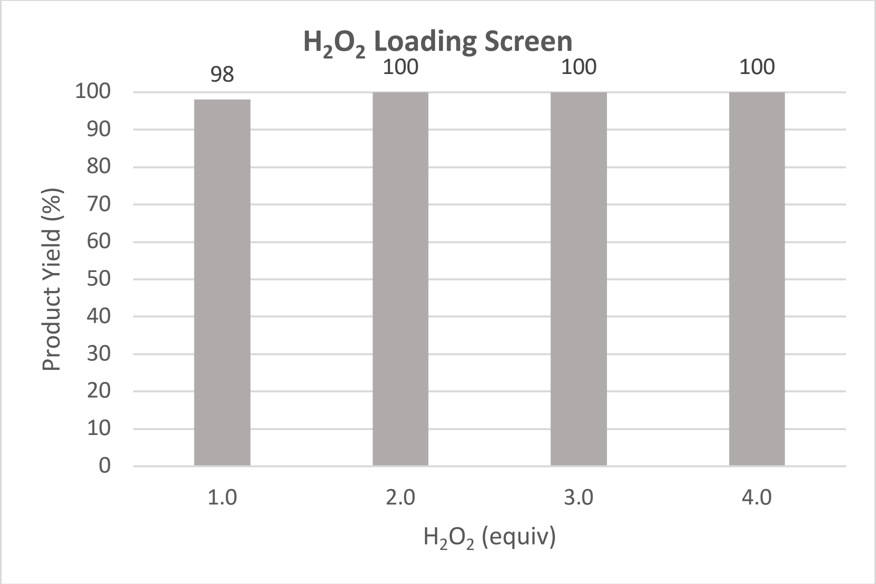

Figure S8. Hydrogen Peroxide (H_2O_2) Loading Screen

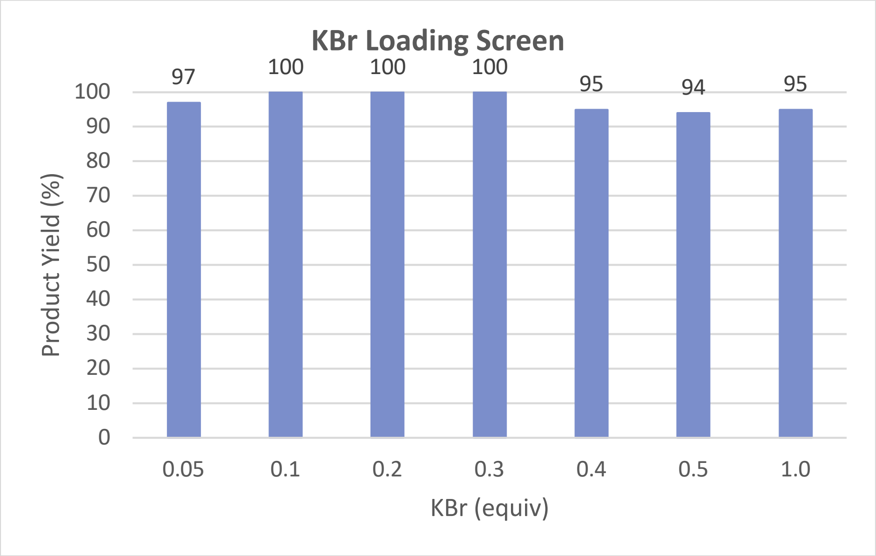

Figure S9. Potassium Bromide (KBr) Loading Screen

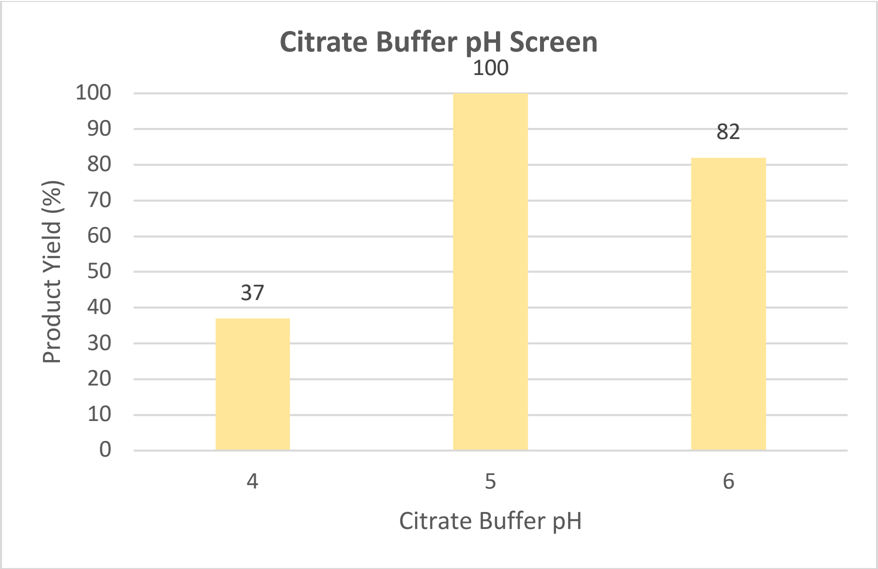

Figure S10. Citrate Buffer pH Screen

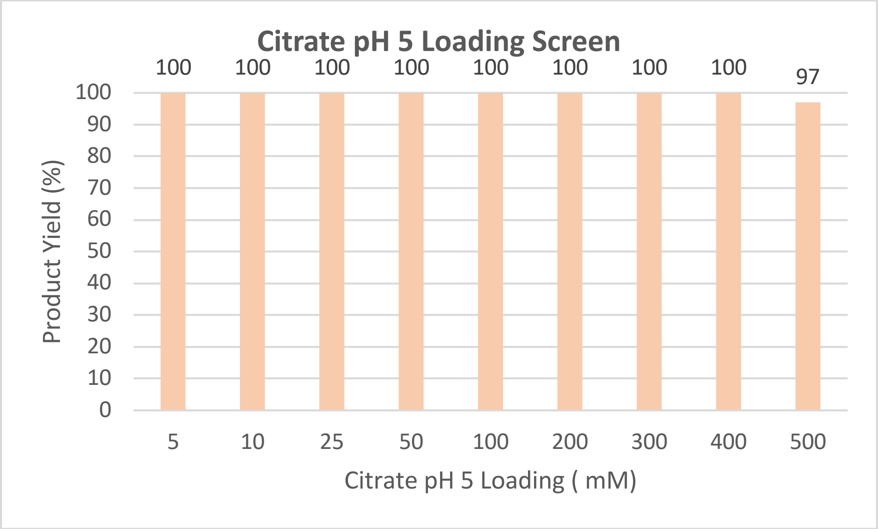

Figure S11. Citrate pH 5 Loading Screen

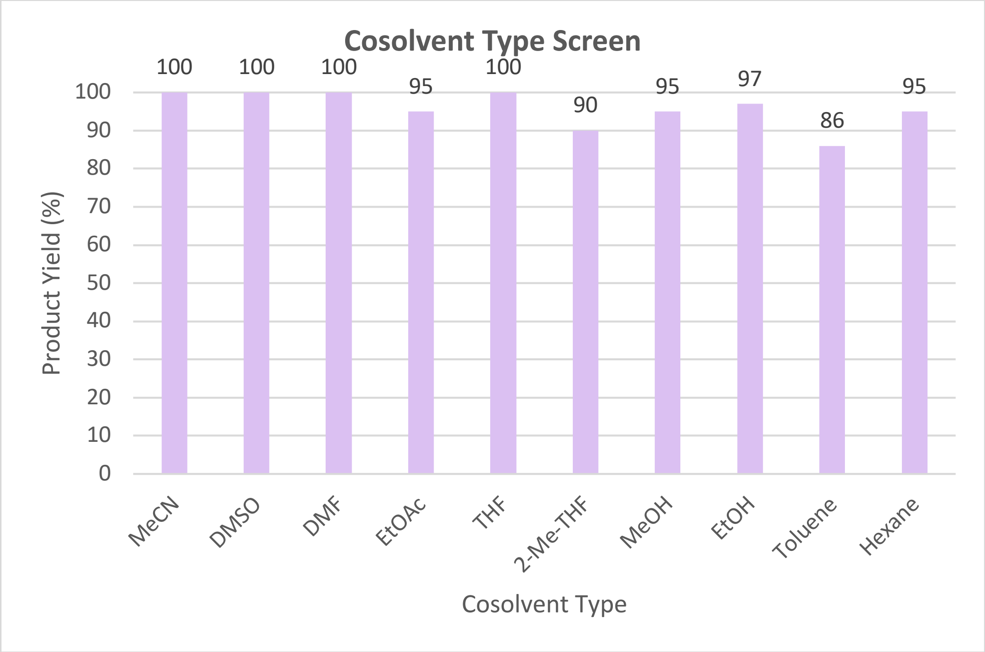

Figure S12. Cosolvent Type Screen

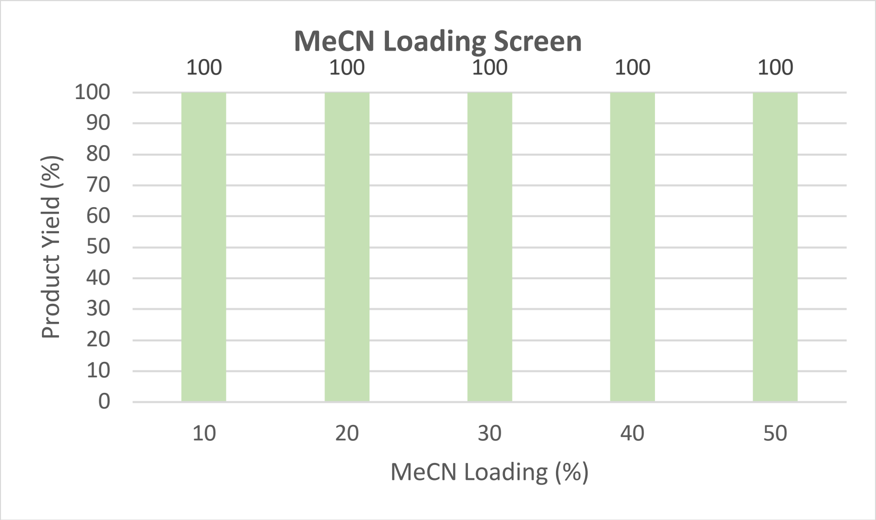

Figure S13. MeCN Loading Screen

Spirooxdindole Formation with Purified *Cp*VBPO Optimization Experiments: All optimization reactions were performed using the General Procedure G. The only variable changed was the one indicated in the Figures below. (*Note: 100 µL of a 8 mg/mL solution of 1,3,5-tribromobenzene was added as an internal standard for yield confirmation, where applicable)*

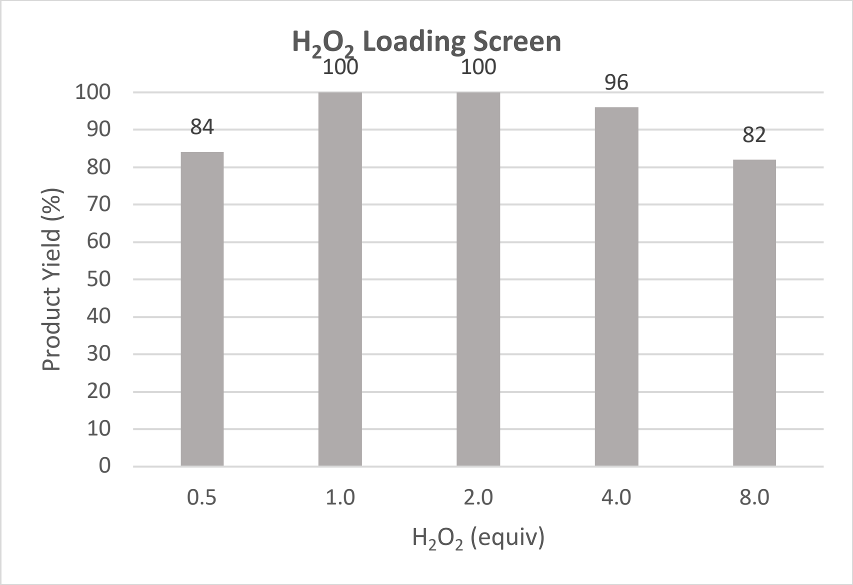

Figure S14. Hydrogen Peroxide (H_2O_2) Loading Screen

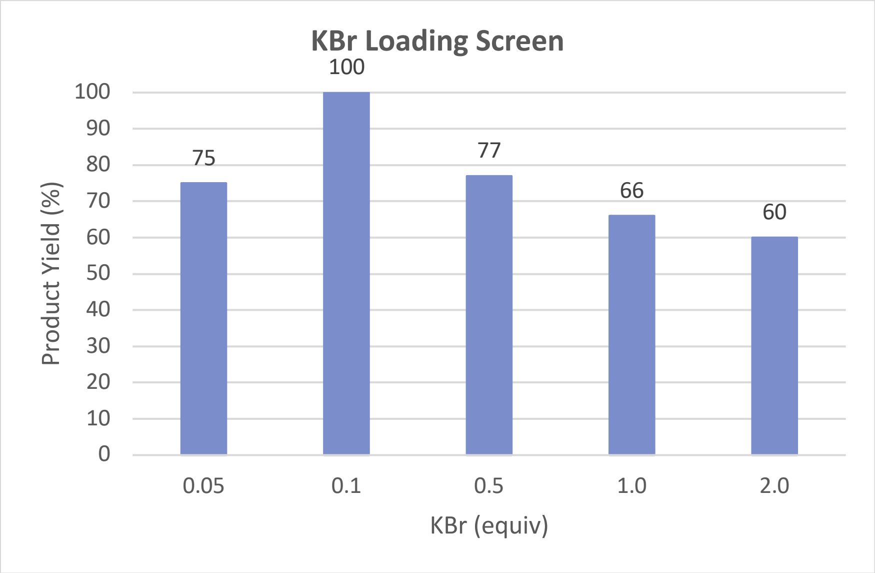

Figure S15. Potassium Bromide (KBr) Loading Screen

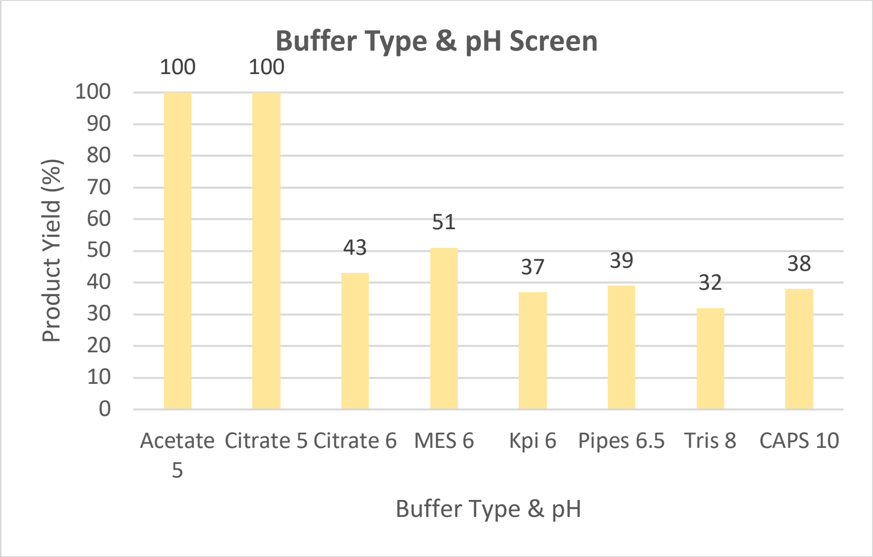

Figure S16. Buffer Type and pH Screen

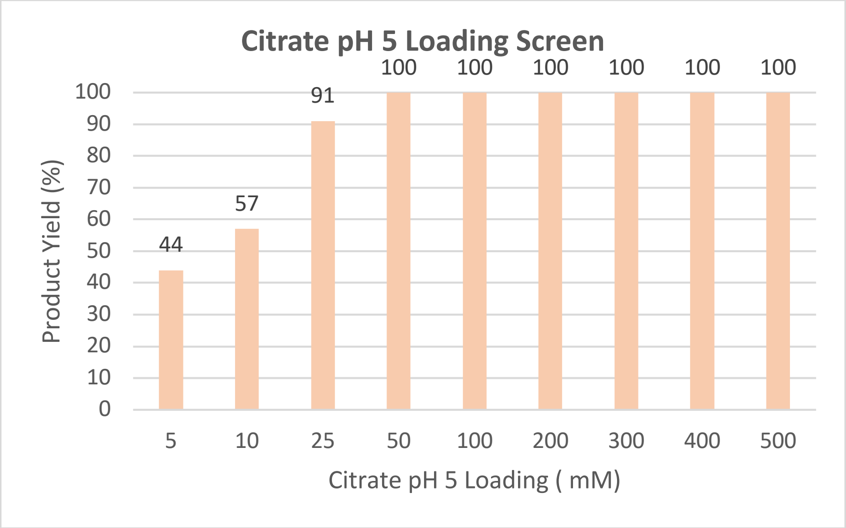

Figure S17. Citrate pH 5 Loading Screen

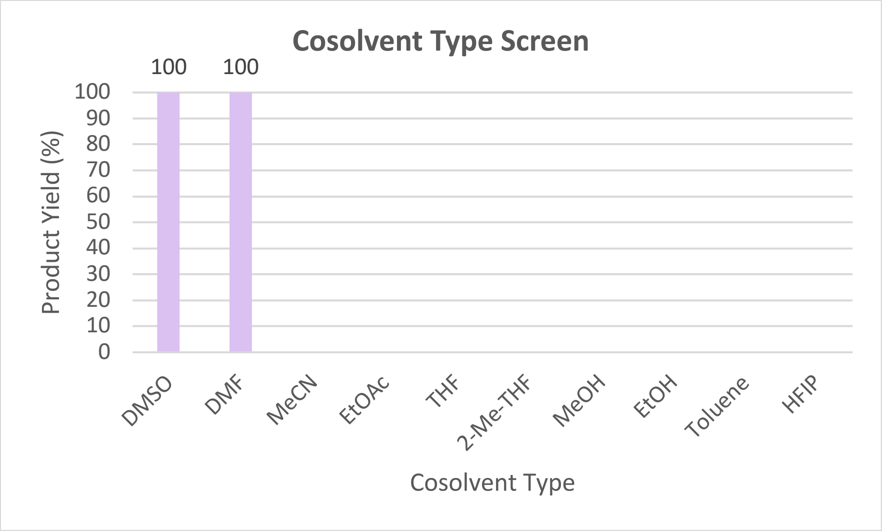

Figure S18. Cosolvent Type Screen

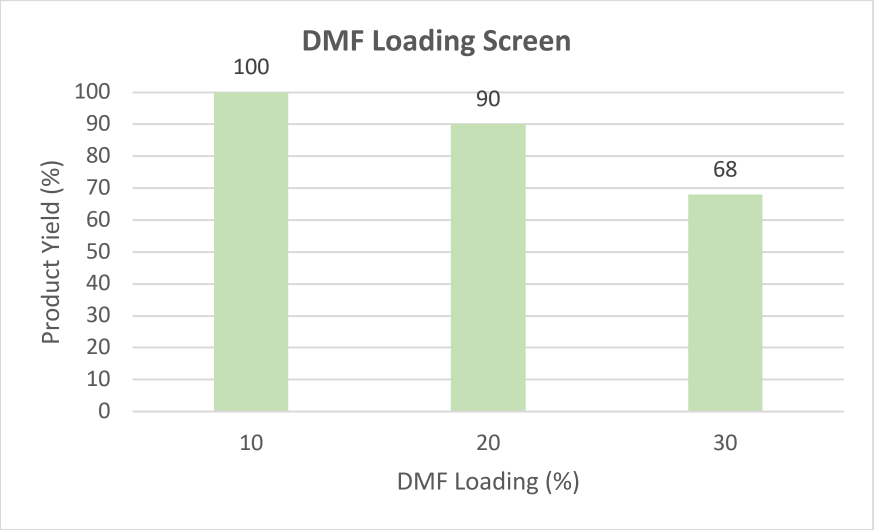

Figure S19. DMF Loading Screen

References

(1). Wells, C. E.; Ramos, L. P. T.; Harstad, L. J.; Hessefort, L. Z.; Lee, H. J.; Sharma, M.; Biegasiewicz, K. F. Decarboxylative Bromooxidation of Indoles by a Vanadium Haloperoxidase. *ACS Catalysis* **2023**, *13* (7), 4622–4628.

(2). Gasteiger, E.; Hoogland, C.; Gattiker, A.; Duvaud, S.; Wilkins, M. R.; Appel, R. D.; Bairoch, A. Protein Identification and Analysis Tools on the ExPASy Server. *The Proteomics Protocols Handbook* **2005**, 571–607.

(3). Jeffrey, V.; Joachim, M. New Puc-Derived Cloning Vectors with Different Selectable Markers and DNA Replication Origins. *Gene* **1991**, *100*, 189–194.

(4). Vincent, G.; Tomakinian, T.; Kouklovsky, C. Investigation of the Synthesis of Benzofuroindolines from N-Hydroxyindoles: An O-Arylation/[3,3]-Sigmatropic Rearrangement Sequence. *Synlett* **2015**, *26* (09), 1269–1275.

(5). Ye, J.; Wu, J.; Lv, T.; Wu, G.; Gao, Y.; Chen, H. Oxidative Rearrangement Coupling Reaction for the Functionalization of Tetrahydro-β-carbolines with Aromatic Amines. *Angew. Chem., Int. Ed.* **2017**, *129* (47), 15164–15168.

(6). Li, C.; Chan, C.; Heimann, A. C.; Danishefsky, S. J. On the Rearrangement of an Azaspiroindolenine to a Precursor to Phalarine: Mechanistic Insights. *Angew. Chem., Int. Ed.* **2007**, *46* (9), 1444–1447.

(7). Ye, J.; Lin, Y.; Liu, Q.; Xu, D.; Wu, F.; Liu, B.; Gao, Y.; Chen, H. Biomimetic Oxidative Coupling Cyclization Enabling Rapid Construction of Isochromanoindolenines. *Org. Lett.* **2018**, *20* (17), 5457–5460.

(8). Ding, Y.; Shen, L.; Liang, K.; Xia, C. Synthesis of C2-Carbonyl Indoles via Visible Light-Induced Oxidative Cleavage of an Aminomethylene Group. *J. Org. Chem.* **2022**, *87* (24), 16644–16654.

(9). Bettoni, L.; Gaillard, S.; Renaud, J.-L. Iron-Catalyzed β-Alkylation of Alcohols. *Org. Lett* **2019**, *21* (20), 8404–8408.

(10). Zhou, J.; Wang, C.; Xue, D.; Tang, W.; Xiao, J.; Li, C. Synthesis of Isochromans via Fe(OTF)2-Catalyzed Oxa-Pictet–Spengler Cyclization. *Tetrahedron* **2018**, *74* (49), 7040–7046.

(11). Jiang, X.; Yang, J.; Zhang, F.; Yu, P.; Yi, P.; Sun, Y.; Wang, Y. Synthesis of Quaternary 3,3-Disubstituted 2-Oxindoles from 2-Substituted Indole Using Selectfluor. *Org. Lett* **2016**, *18* (13), 3154–3157.

(12). Sathish, M.; Nachtigall, F. M.; Santos, L. S. Bifunctional Thiosquaramide Catalyzed Asymmetric Reduction of Dihydro-β-Carbolines and Enantioselective Synthesis of (−)-Coerulescine and (−)-Horsfiline by Oxidative Rearrangement. *RSC Adv.* **2020**, *10* (63), 38672–38677.

(13). Arteaga Giraldo, J. J.; Lindsay, A. C.; Seo, R. C.-Y.; Kilmartin, P. A.; Sperry, J. Electrochemical Oxidation of 3-Substituted Indoles. *Organic & Biomolecular Chemistry* **2023**, *21* (27), 5609–5615.

(14). Shelar, S. V.; Argade, N. P. Regioselective Oxidation of Indoles to 2-Oxindoles. *Org. Biomol. Chem* **2019**, *17* (27), 6671–6677.

(15). Song, D.; Chen, R.; Huang, W.; Yang, P.; Chen, L.; Zhang, J.; Ling, F.; Zhong, W. Room-temperature Synthesis of *α*-alkylated Indolin-2-ones via a Photo Assisted Iron Catalyzed Borrowing Hydrogen Reaction. *Adv. Synth. Catal.* **2024**, *366* (8), 1893–1898.

(16). Khan, J.; Yadav, N.; Tyagi, A.; Hazra, C. K. Silyl Cation-Initiated, Brønsted Acid-Catalyzed Strategy toward Unsymmetrical 3,3-Disubstituted 2-Oxindoles and Azonazine Cores. *J. Org. Chem.* **2022**, *87* (16), 11097–11111.
(17). Wang, J.; Chen, Y.; Du, W.; Chen, N.; Fu, K.; He, Q.; Shao, L. Green Oxidative Rearrangement of Indoles Using Halide Catalyst and Hydrogen Peroxide. *Tetrahedron* **2022**, *127*, 133101.
(18). Eremeev, R. O.; Efremov, A. M.; Zakharova, D. V.; Beznos, O. V.; Sokolova, E. V.; Kalitin, K. Y.; Mukha, O. Y.; Vinogradova, D. V.; Veselov, I. M.; Shevtsov, P. N.; Dubova, L. G.; Babkov, D. A.; Spasov, A. A.; Shevtsova, E. F.; Lozinskaya, N. A. Discovery of Novel 2-oxindoles as Compounds with Antiglaucoma Activity. *ChemMedChem* **2025**, *20* (10).
(19). Zhao, Z.; Zeng, G.; Chen, Y.; Zheng, J.; Chen, Z.; Shao, Y.; Zhang, F.; Chen, J.; Li, R. Palladium-Catalyzed Three-Component Cascade Reaction of Nitriles: Synthesis of 2-Arylquinoline-4-Carboxylates. *Org. Lett.* **2021**, *23* (20), 7955–7960.
(20). Chan, W. C.; White, P. D. *Fmoc Solid Phase Peptide Synthesis: A Practical Approach*; Oxford University Press, 2020.

<u>**Spectroscopic Data**</u>

Starting Materials

N-(2-(1H-indol-3-yl)ethyl)acetamide (SM-13)

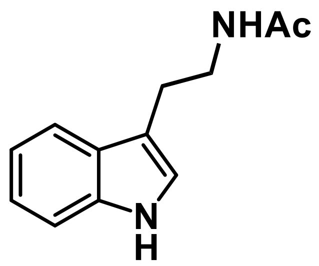

^{1}H-NMR ($CDCl_3$)

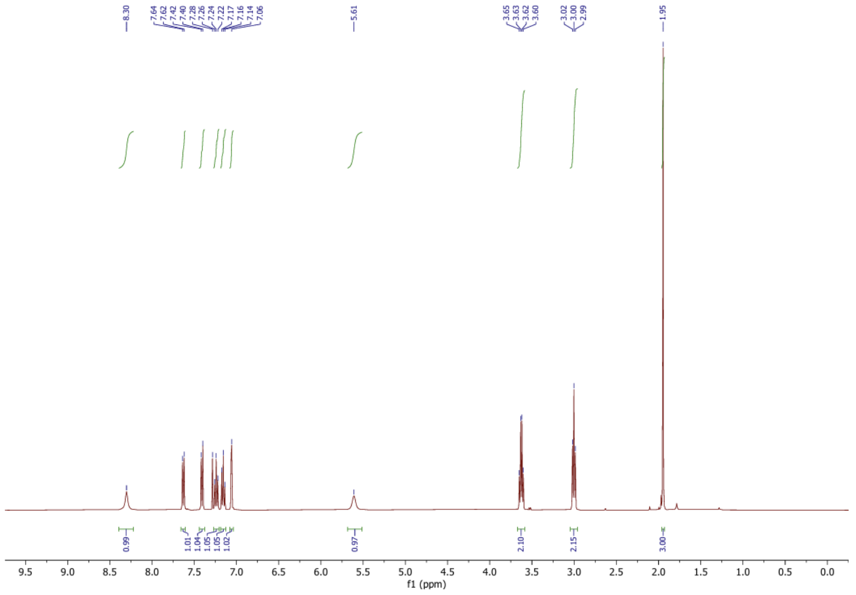

^{13}C-NMR ($CDCl_3$)

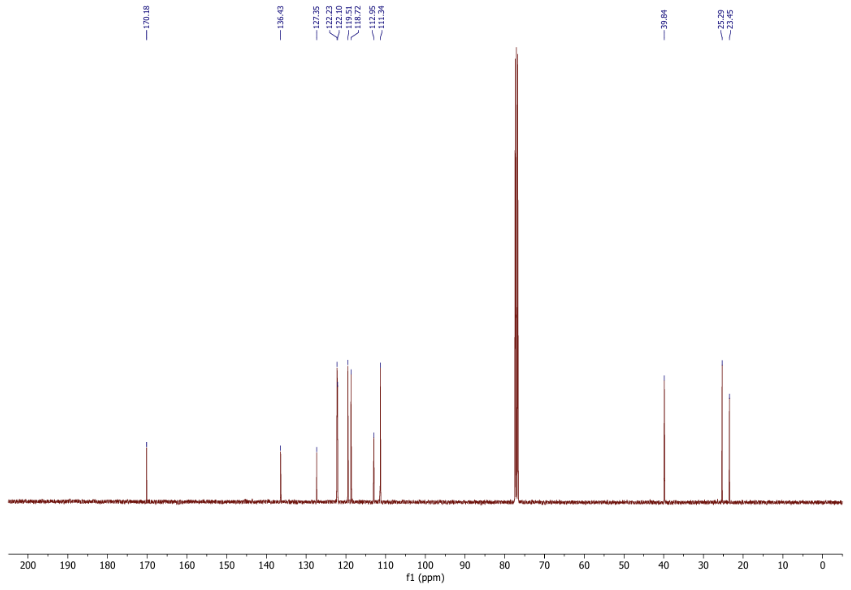

1-(1,3,4,9-tetrahydro-2H-pyrido[3,4-b]indol-2-yl)ethan-1-one (3)

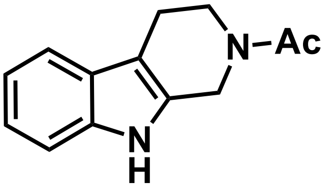

^{1}H-NMR (DMSO-d6)

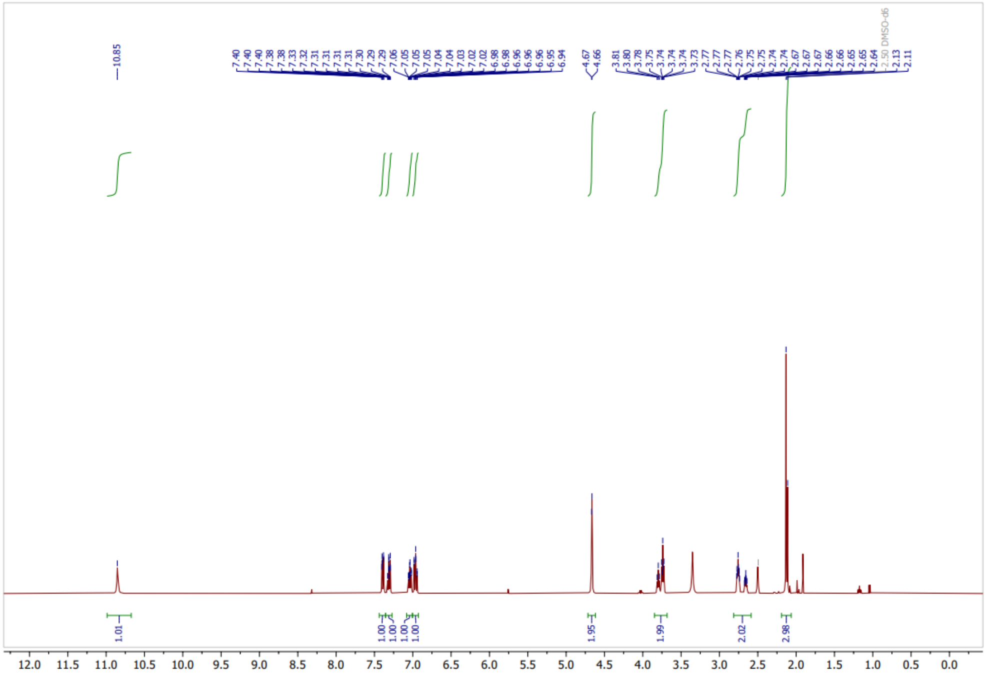

^{13}C-NMR (DMSO-d6)

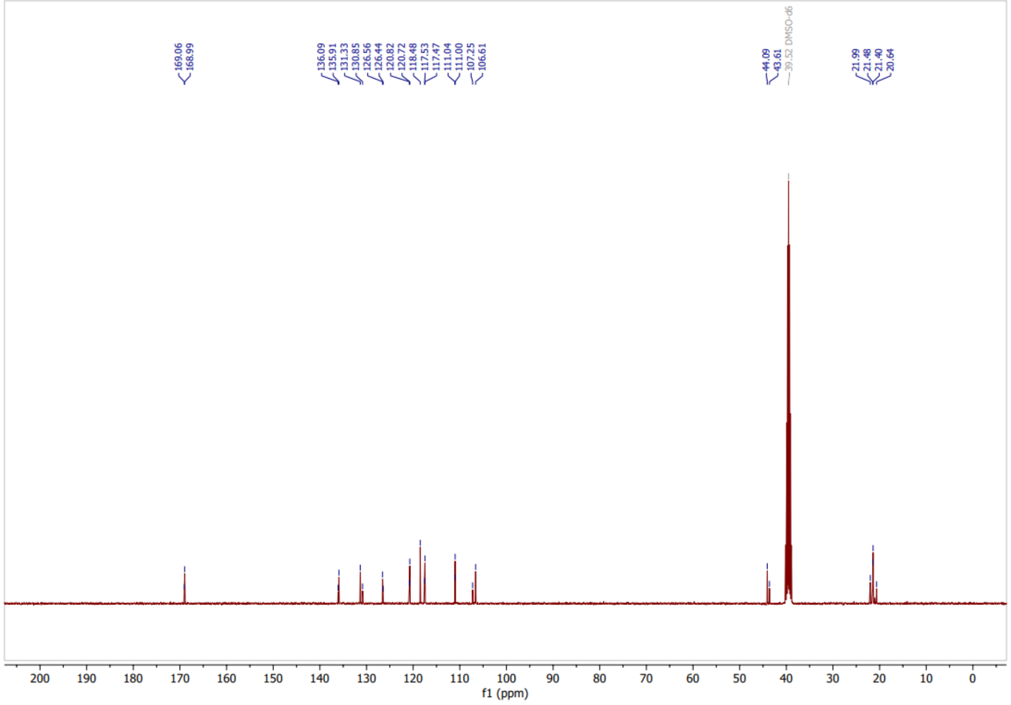

tert-butyl 1,3,4,9-tetrahydro-2H-pyrido[3,4-b]indole-2-carboxylate (SM-21)

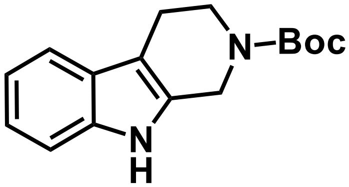

^{1}H-NMR (DMSO-d6)

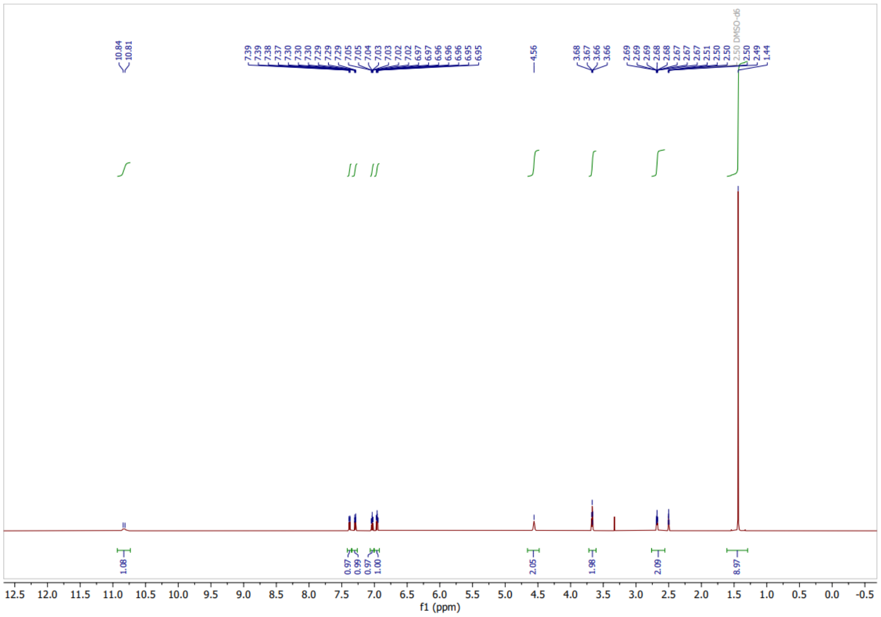

^{13}C-NMR (DMSO-d6)

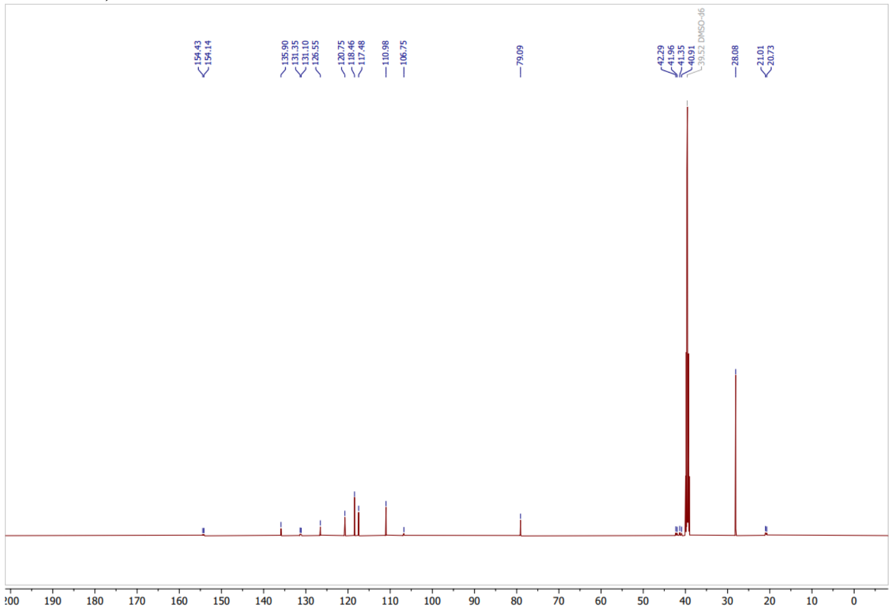

2,2,2-trichloroethyl 1,3,4,9-tetrahydro-2H-pyrido[3,4-b]indole-2-carboxylate (SM-22)

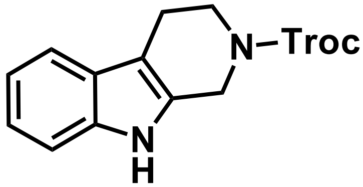

^{1}H-NMR (DMSO-d6)

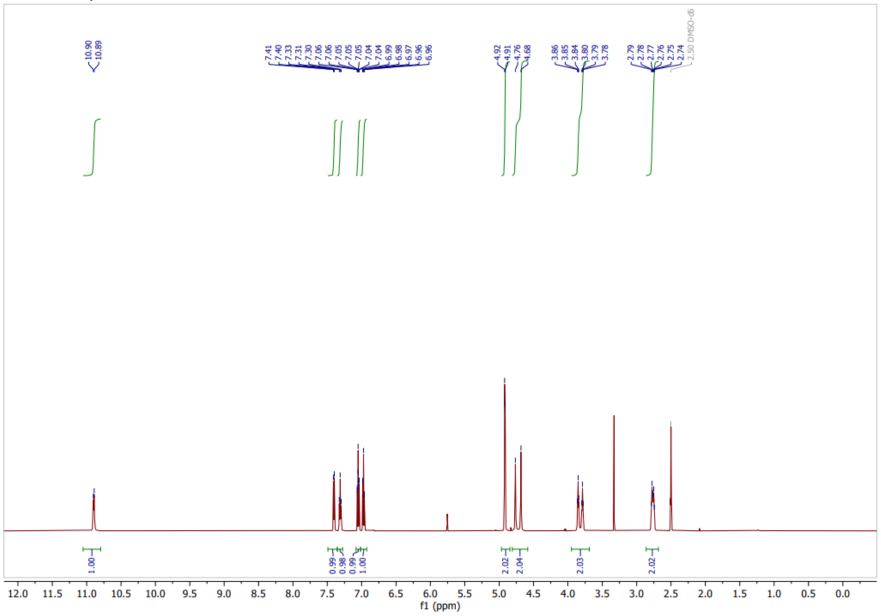

^{13}C-NMR (DMSO-d6)

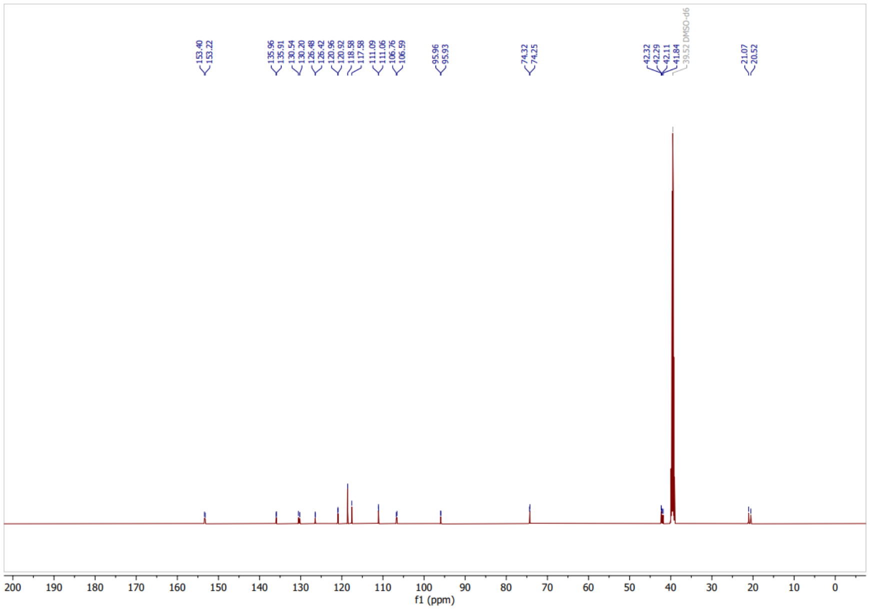

methyl 1,3,4,9-tetrahydro-2H-pyrido[3,4-b]indole-2-carboxylate (SM-23)

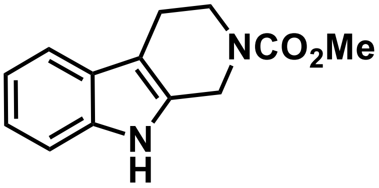

^{1}H-NMR (DMSO-d6)

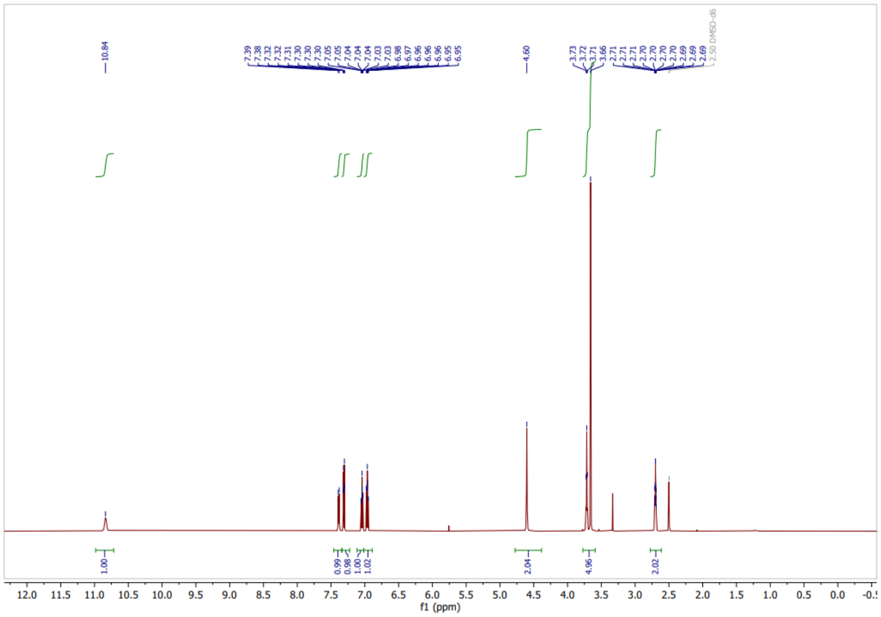

^{13}C-NMR (DMSO-d6)

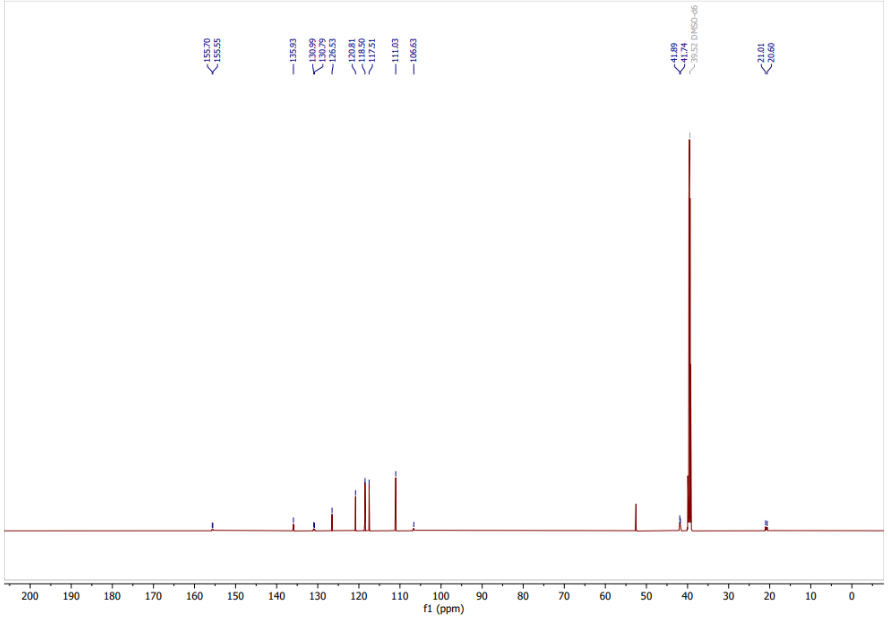

1-(9-methyl-1,3,4,9-tetrahydro-2H-pyrido[3,4-b]indol-2-yl)ethan-1-one (SM-24)

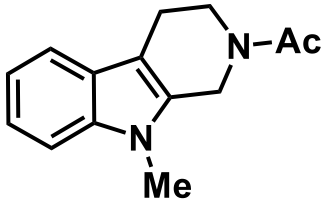

^{1}H-NMR (DMSO-d6)

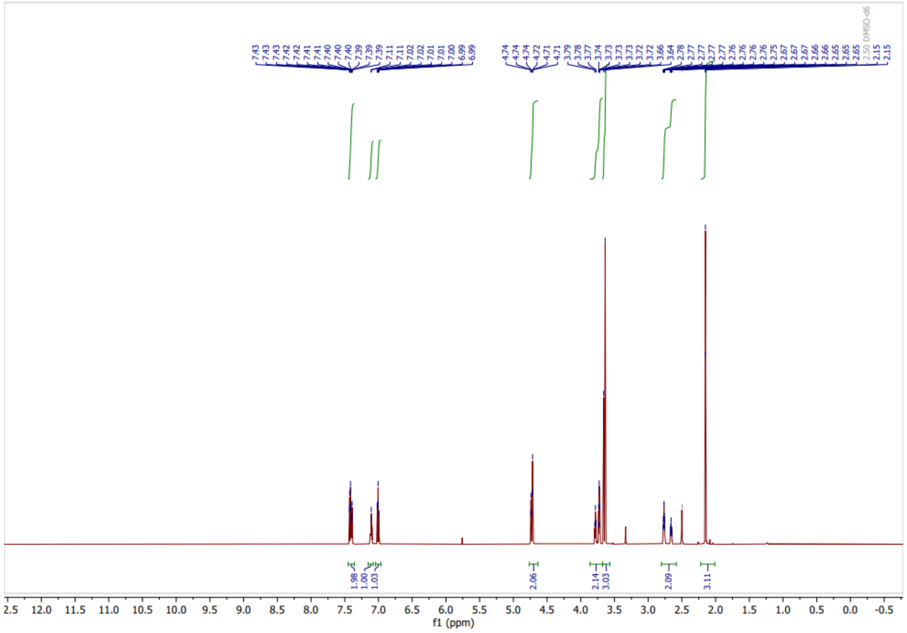

^{13}C-NMR (DMSO-d6)

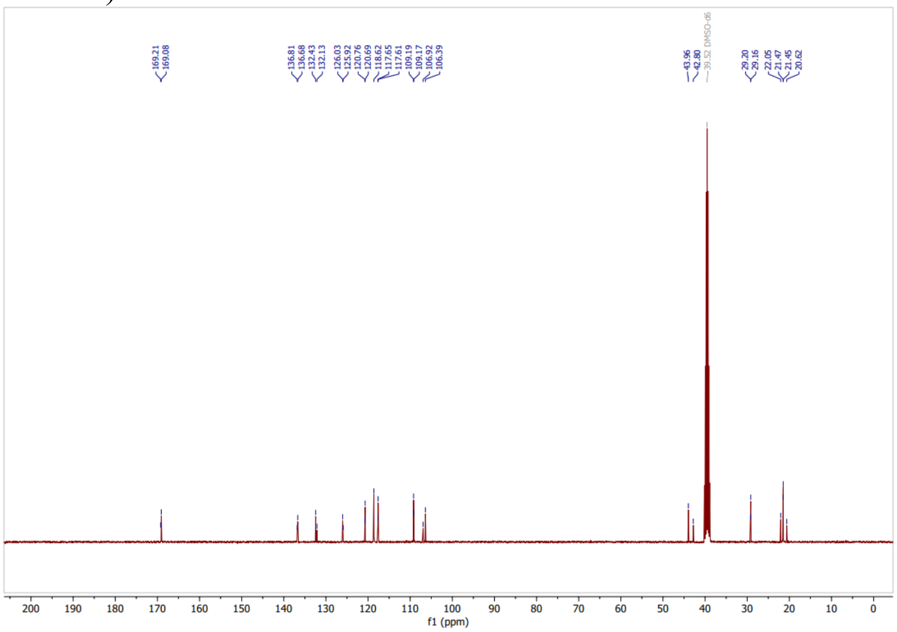

dimethyl (S)-1,3,4,9-tetrahydro-2H-pyrido[3,4-b]indole-2,3-dicarboxylate (SM-25)

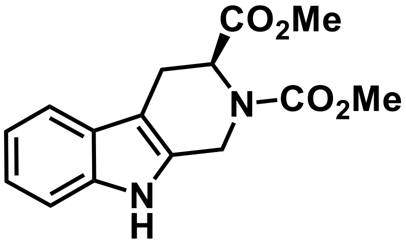

^{1}H-NMR (DMSO-d6)

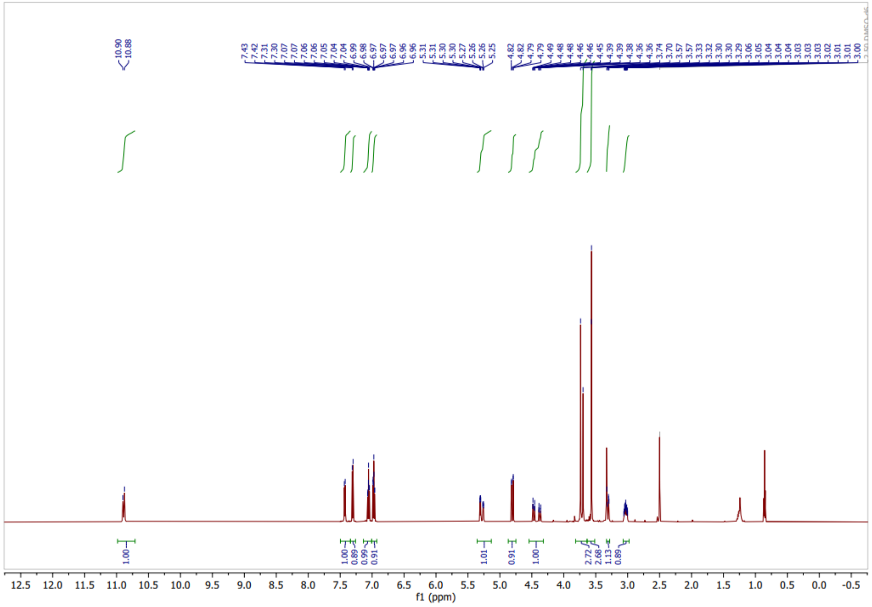

^{13}C-NMR (DMSO-d6)

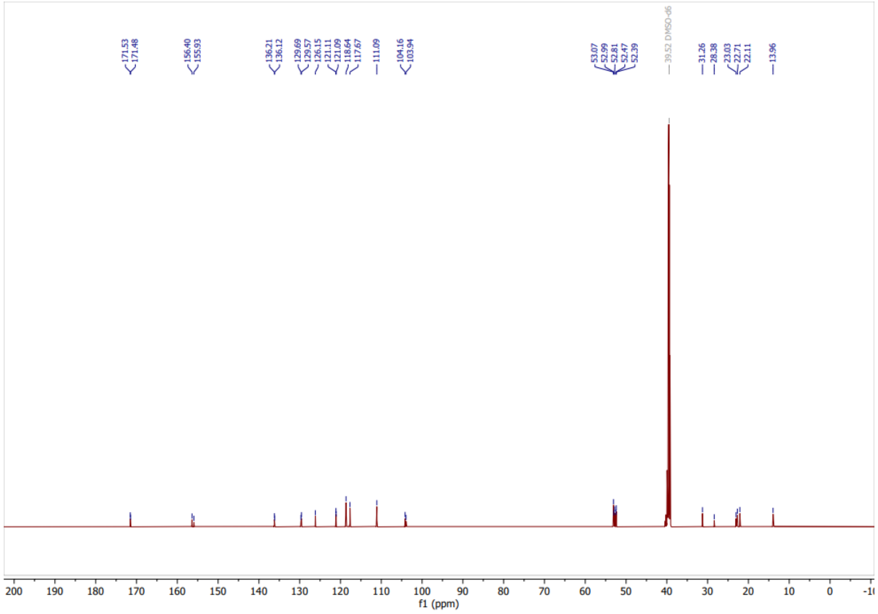

1,1-dimethyl-1,3,4,9-tetrahydropyrano[3,4-b]indole (SM-A-26)

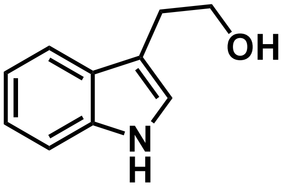

^{1}H-NMR (DMSO-d6)

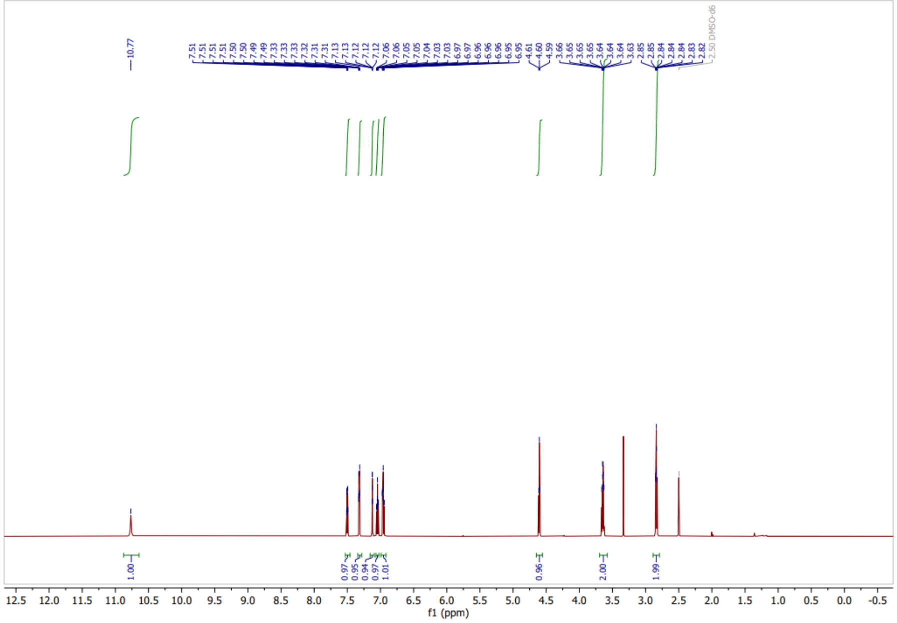

^{13}C-NMR (DMSO-d6)

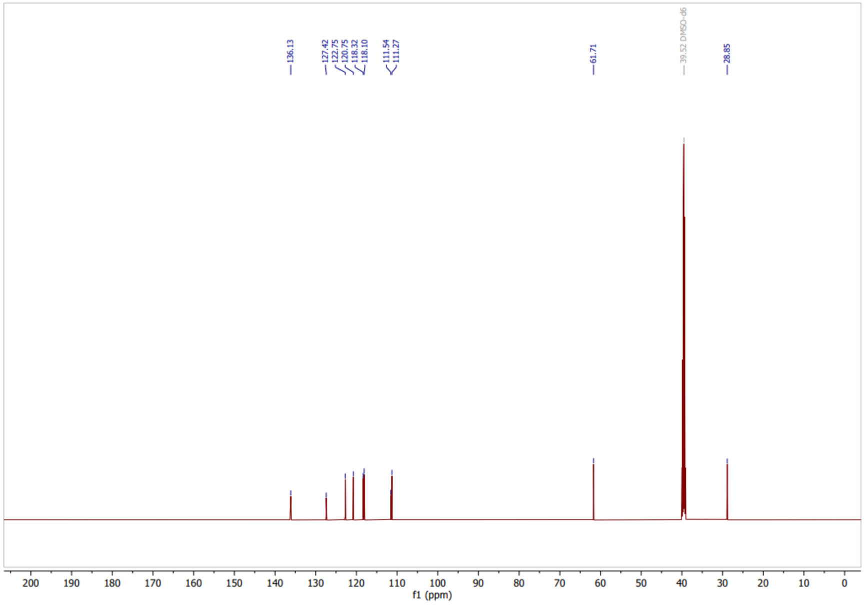

1,1-dimethyl-1,3,4,9-tetrahydropyrano[3,4-b]indole (SM-B-26)

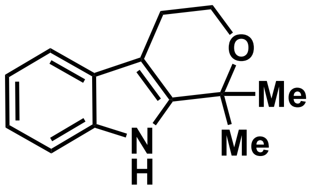

^{1}H-NMR (DMSO-d6)

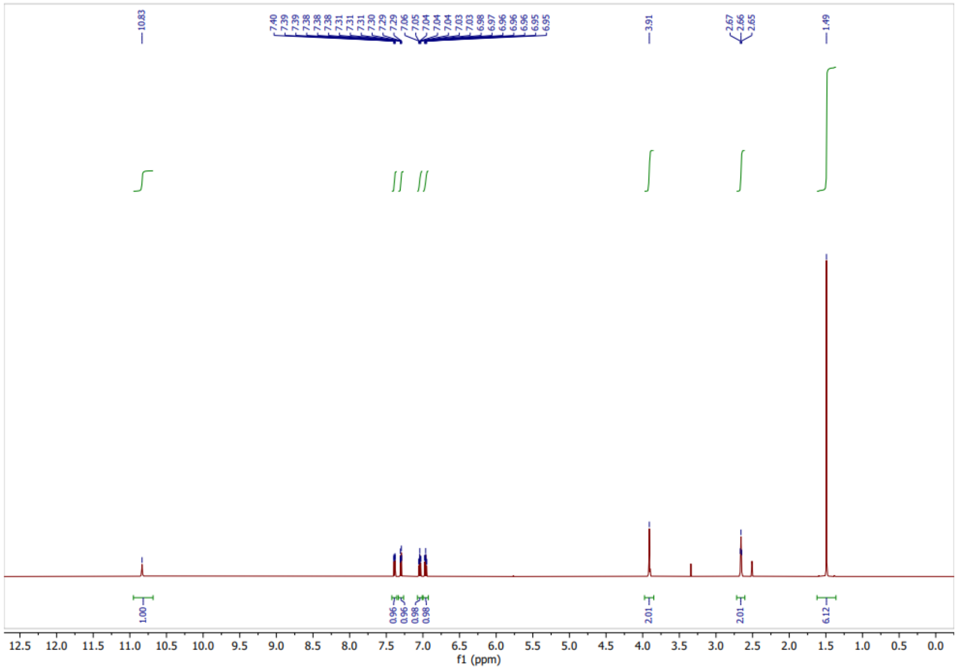

^{13}C-NMR (DMSO-d6)

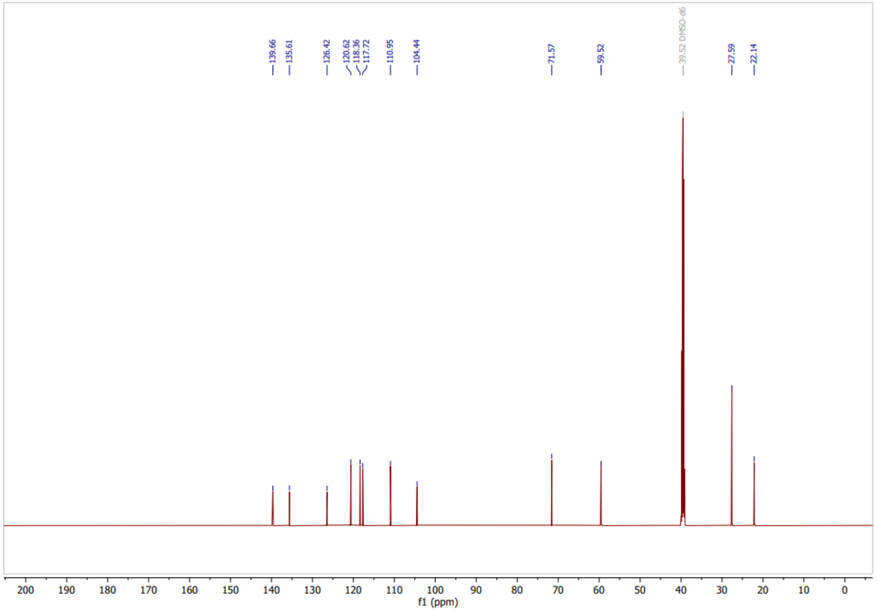

4',9'-dihydro-3'H-spiro[cyclohexane-1,1'-pyrano[3,4-b]indole] (SM-27)

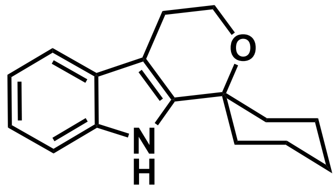

^{1}H-NMR (DMSO-d6)

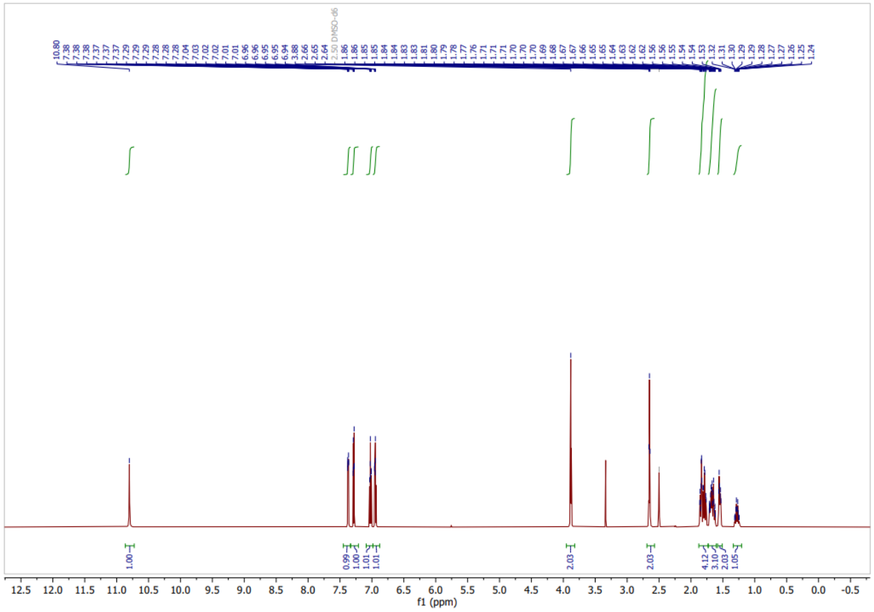

^{13}C-NMR (DMSO-d6)

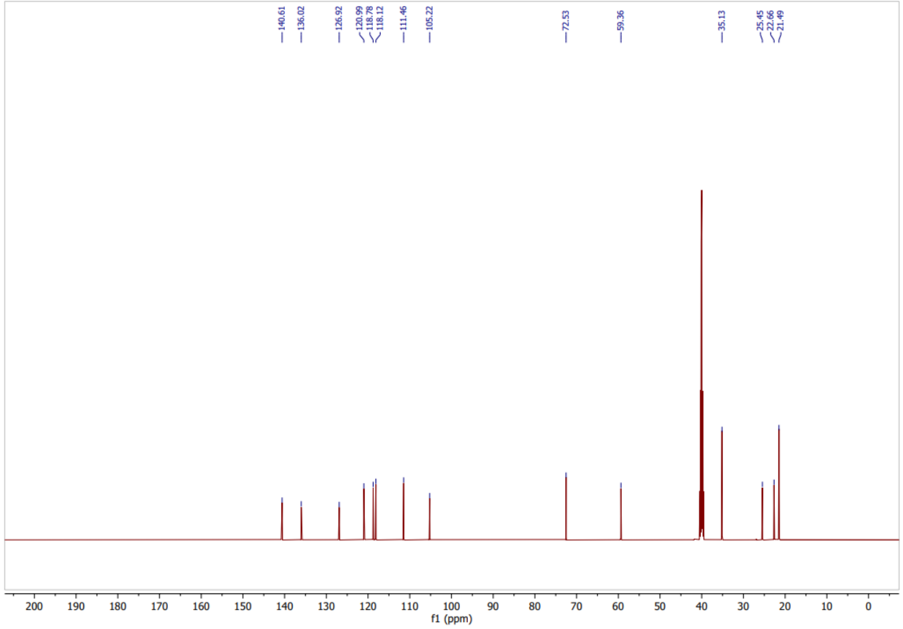

tert-butyl 4',9'-dihydro-3'H-spiro[piperidine-4,1'-pyrano[3,4-b]indole]-1-carboxylate (SM-28)

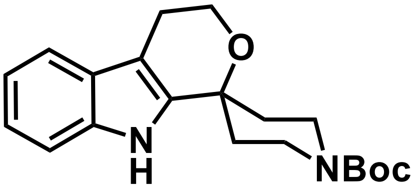

^{1}H-NMR (DMSO-d6)

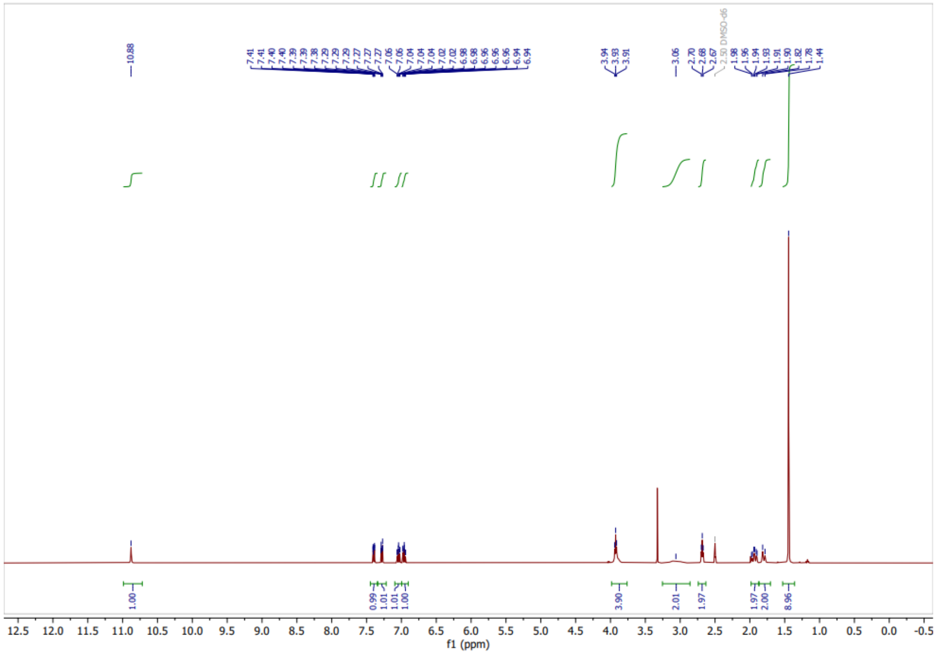

^{13}C-NMR (DMSO-d6)

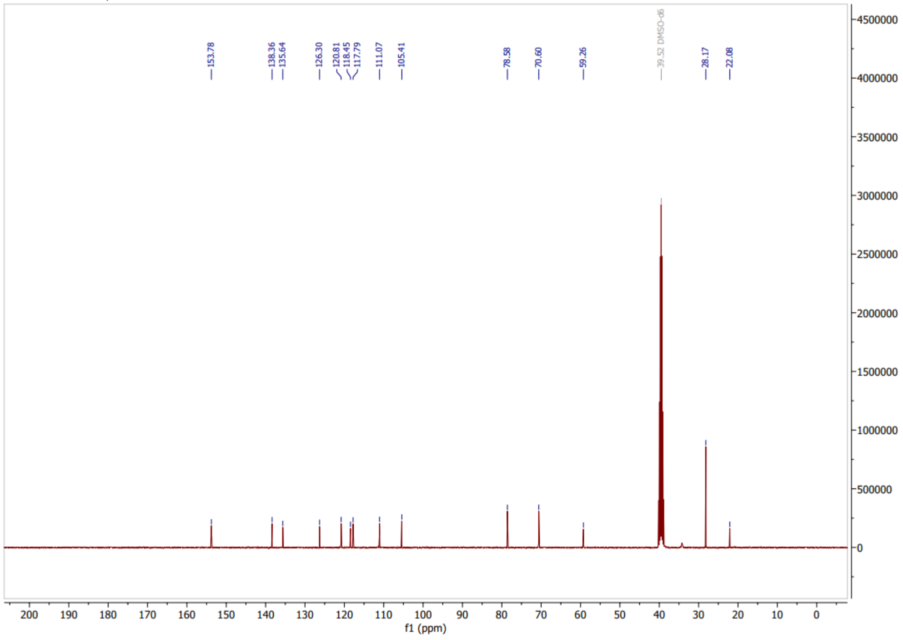

(3-methyl-1H-indol-2-yl)methanol (SM-29)

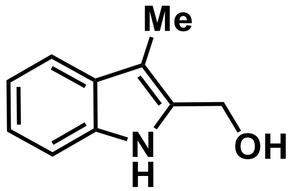

^{1}H-NMR (DMSO-d6)

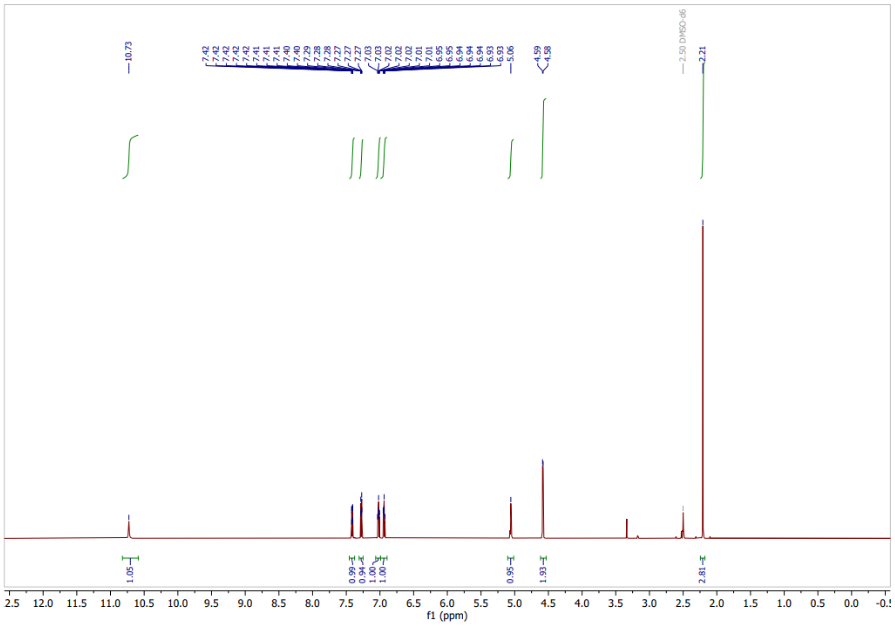

^{13}C-NMR (DMSO-d6)

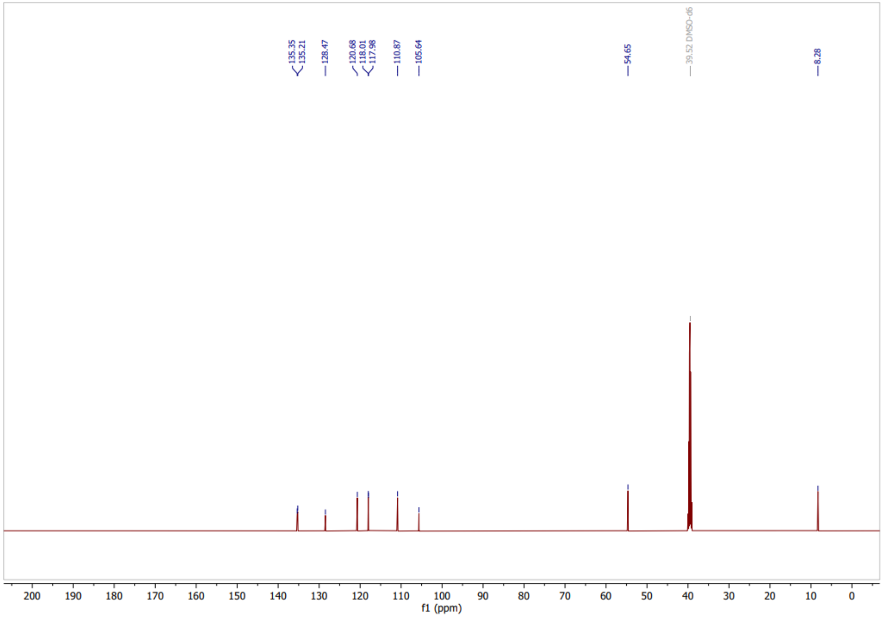

2-methyl-2,3,4,9-tetrahydro-1H-pyrido[3,4-b]indole (43)

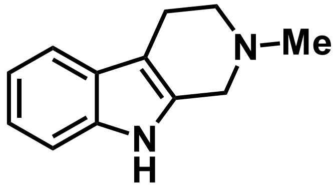

^{1}H-NMR ($CDCl_3$)

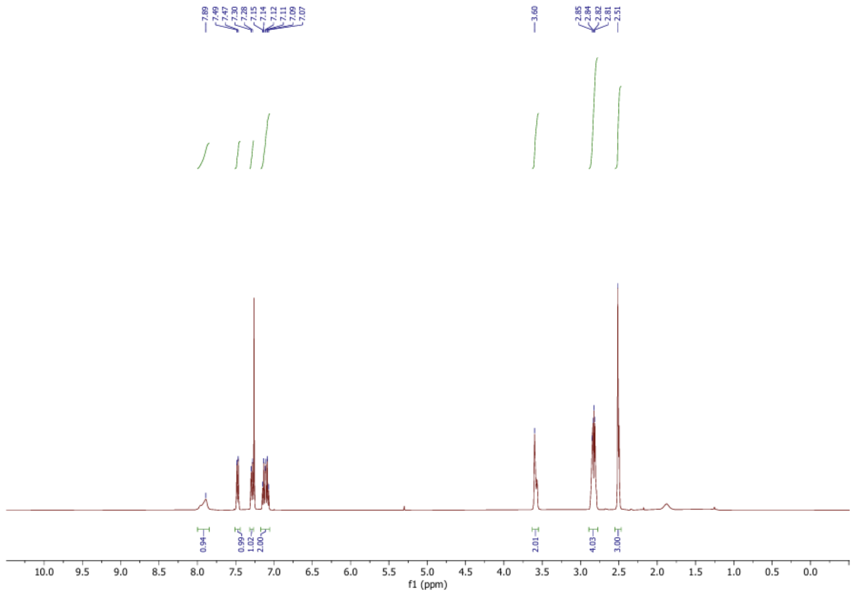

^{13}C-NMR ($CDCl_3$)

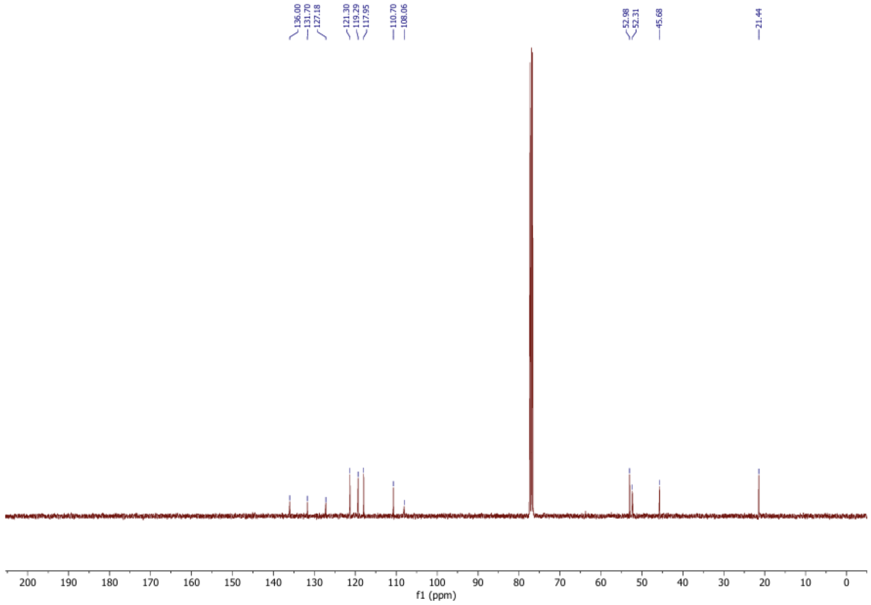

6-methoxy-2-methyl-2,3,4,9-tetrahydro-1H-pyrido[3,4-b]indole (45)

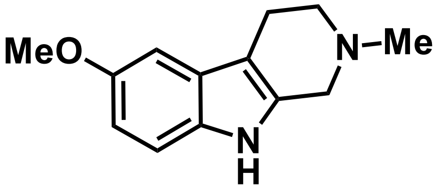

^{1}H-NMR (DMSO-d6)

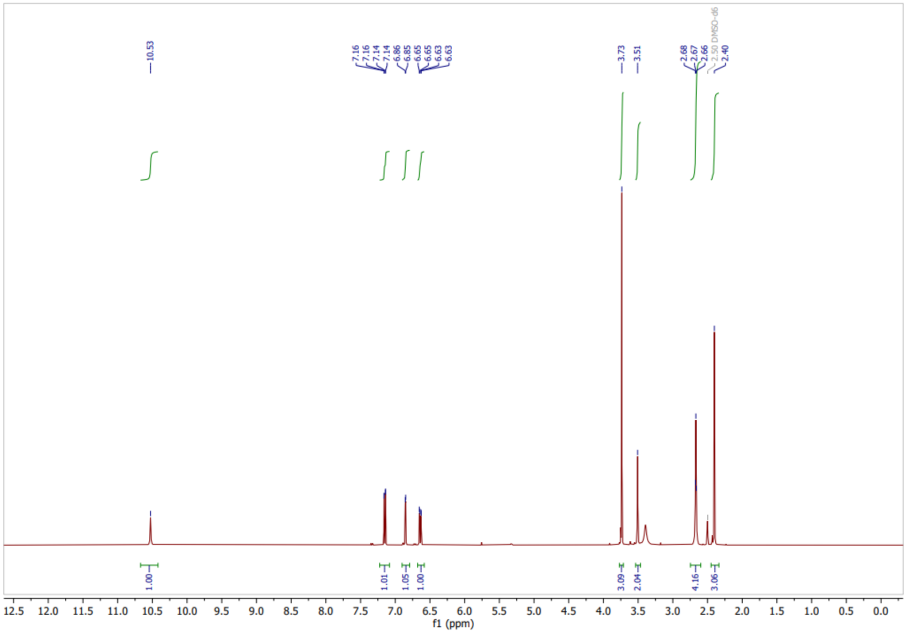

^{13}C-NMR (DMSO-d6)

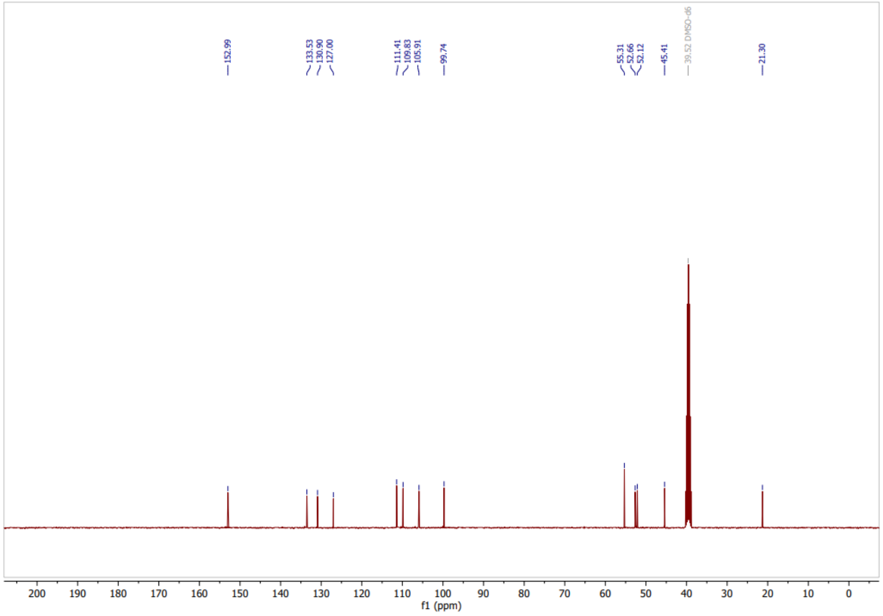

Products
3-methylindolin-2-one (2)

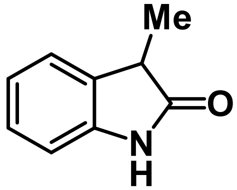

^{1}H-NMR ($CDCl_3$)

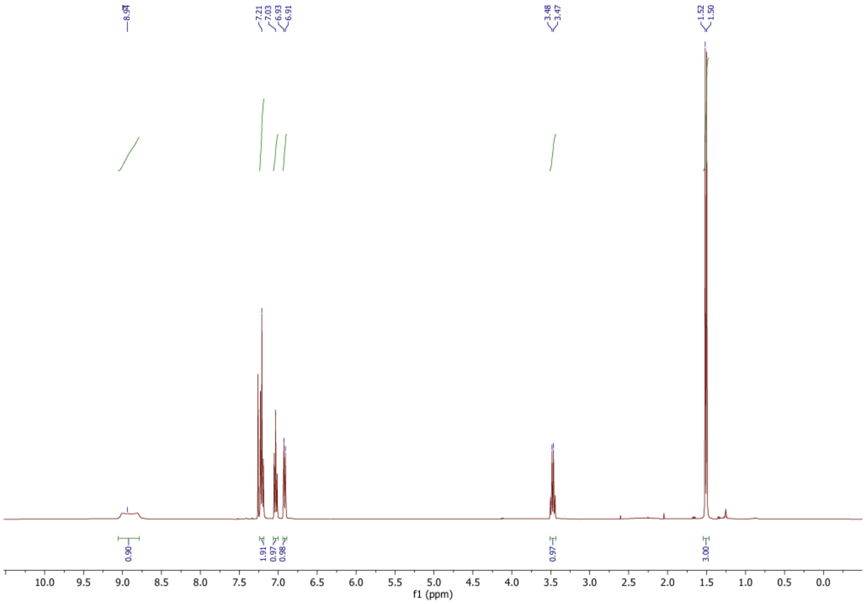

^{13}C-NMR ($CDCl_3$)

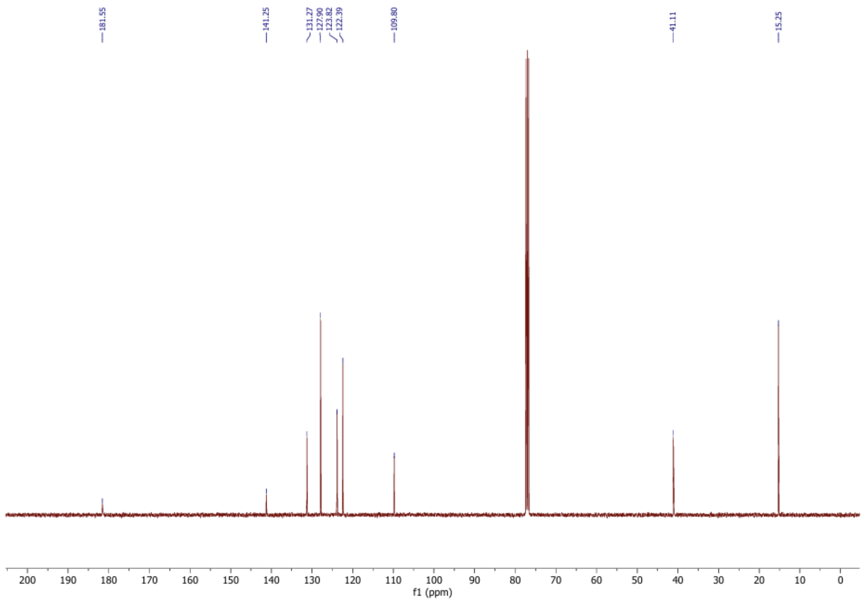

5-methoxy-3-methylindolin-2-one (5)

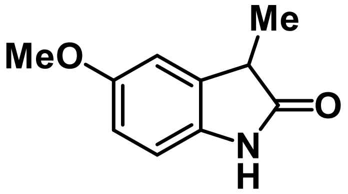

^{1}H-NMR ($CDCl_3$)

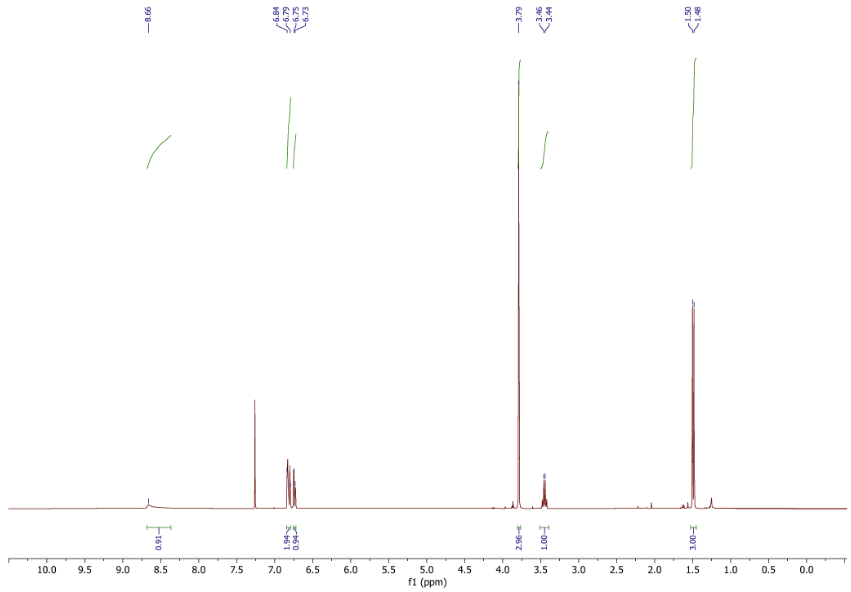

^{13}C-NMR ($CDCl_3$)

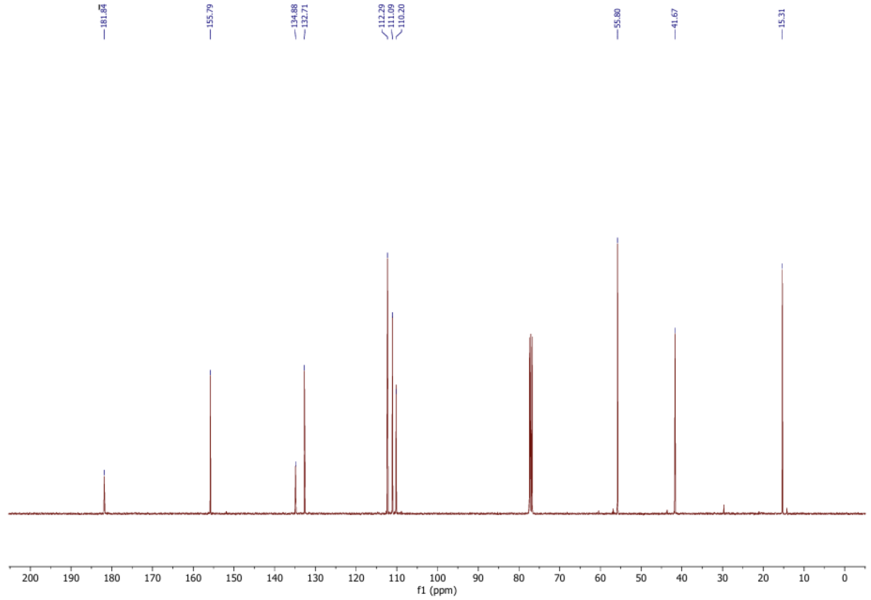

6-bromo-3-methylindolin-2-one (6)

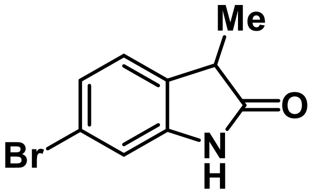

^{1}H-NMR ($CDCl_3$)

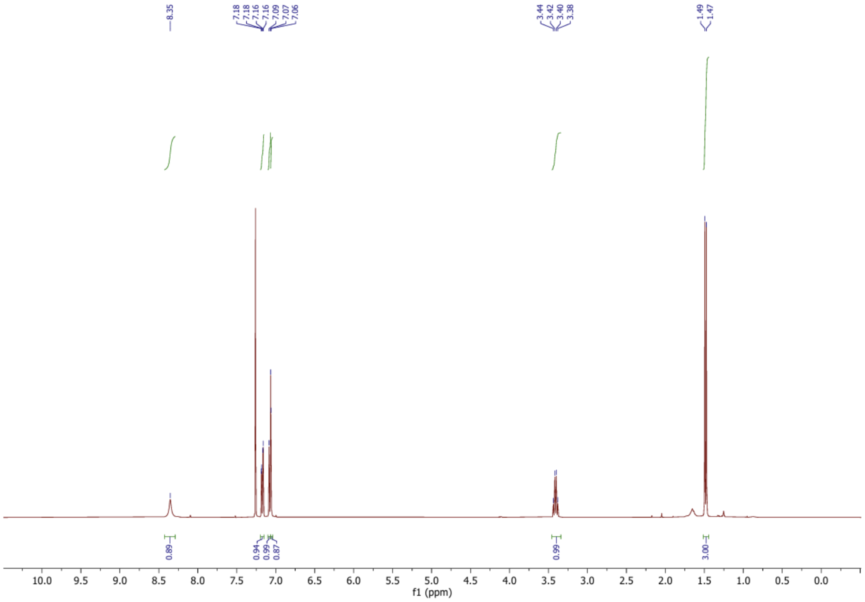

^{13}C-NMR ($CDCl_3$)

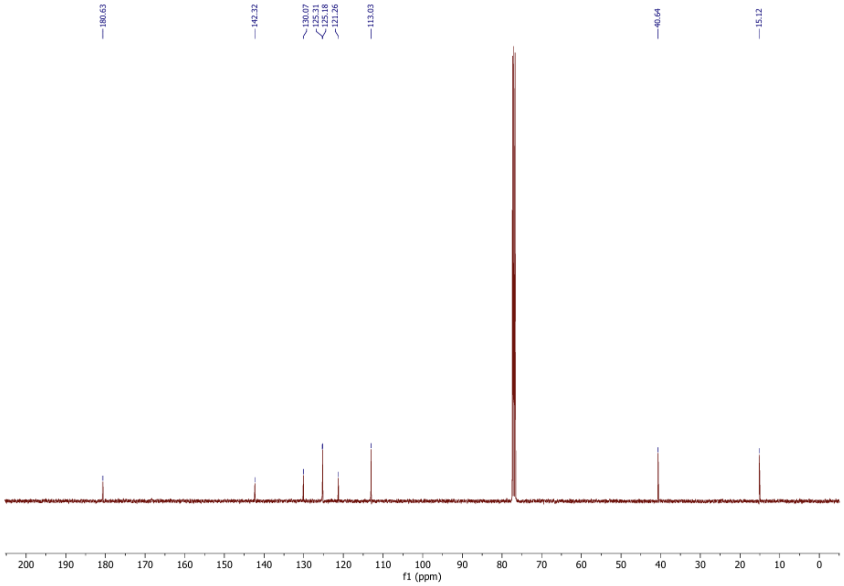

5-bromo-3-methylindolin-2-one (7)

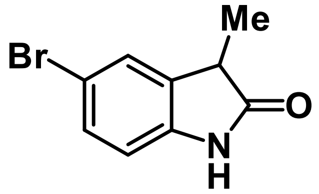

^{1}H-NMR ($CDCl_3$)

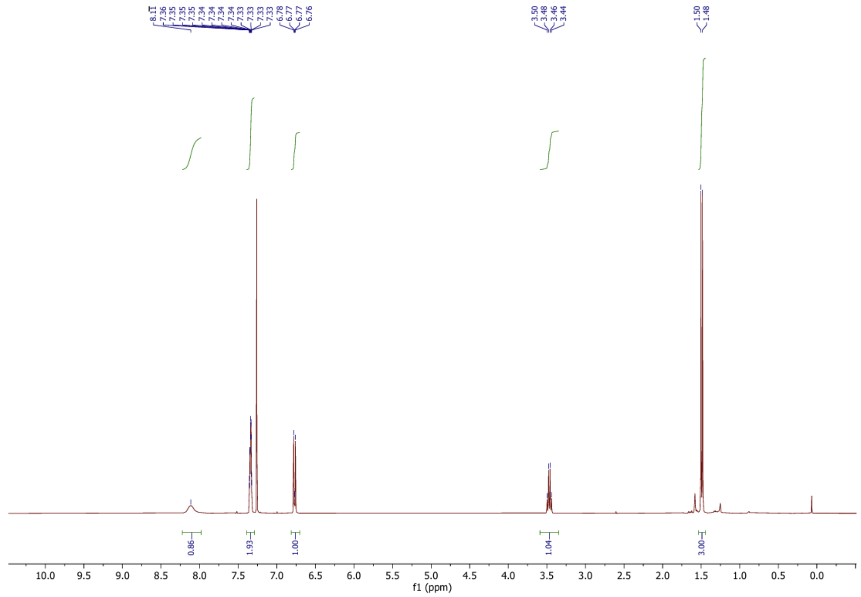

^{13}C-NMR ($CDCl_3$)

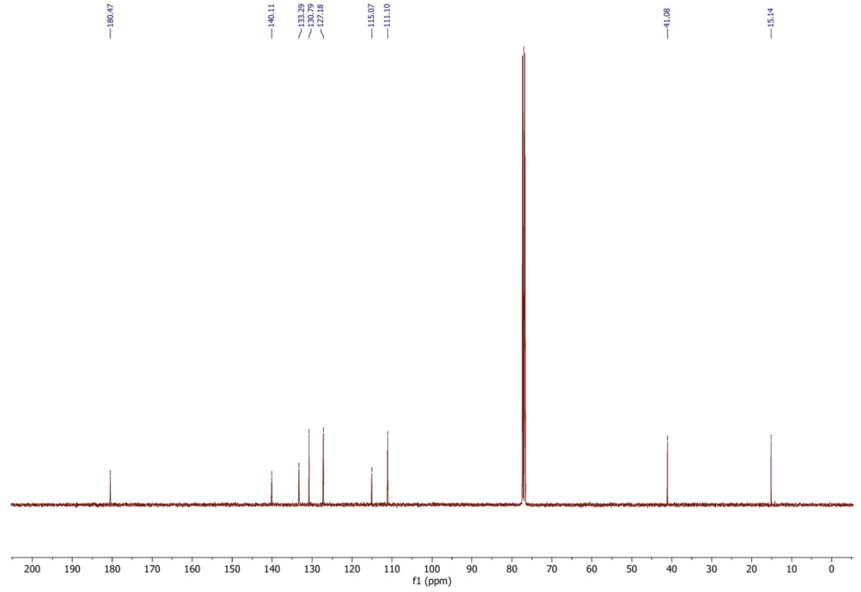

6-fluoro-3-methylindolin-2-one (8)

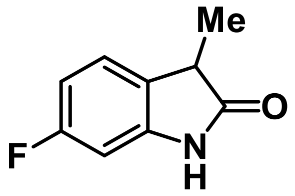

^{1}H-NMR ($CDCl_3$)

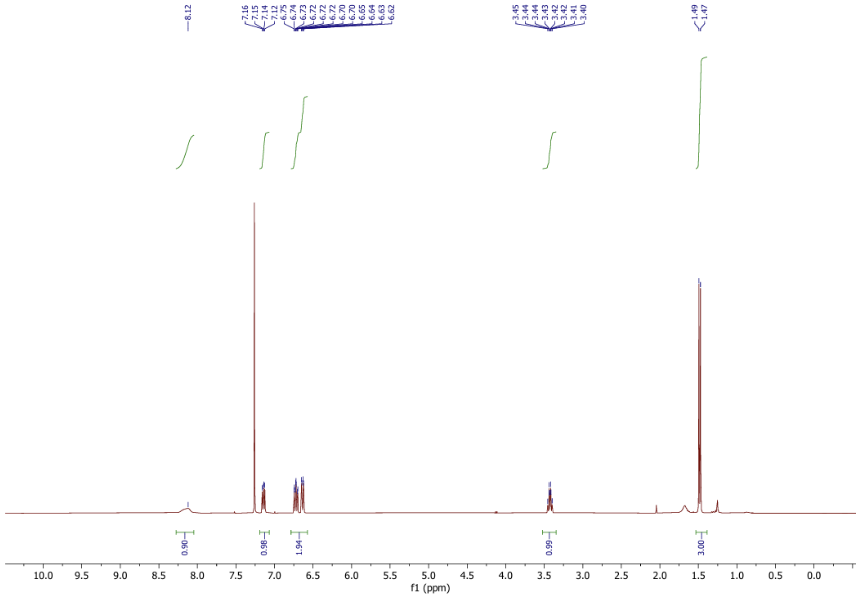

^{13}C-NMR ($CDCl_3$)

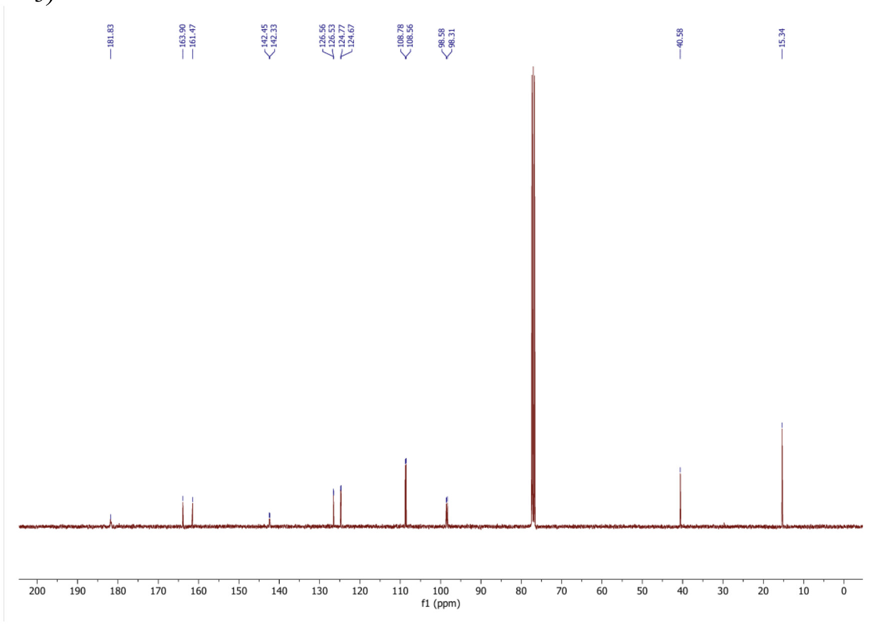

methyl 2-(2-oxoindolin-3-yl)acetate (9)

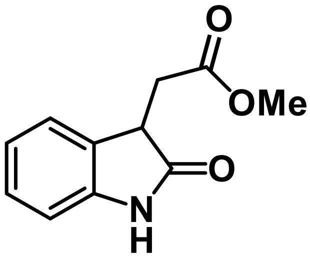

^{1}H-NMR ($CDCl_3$)

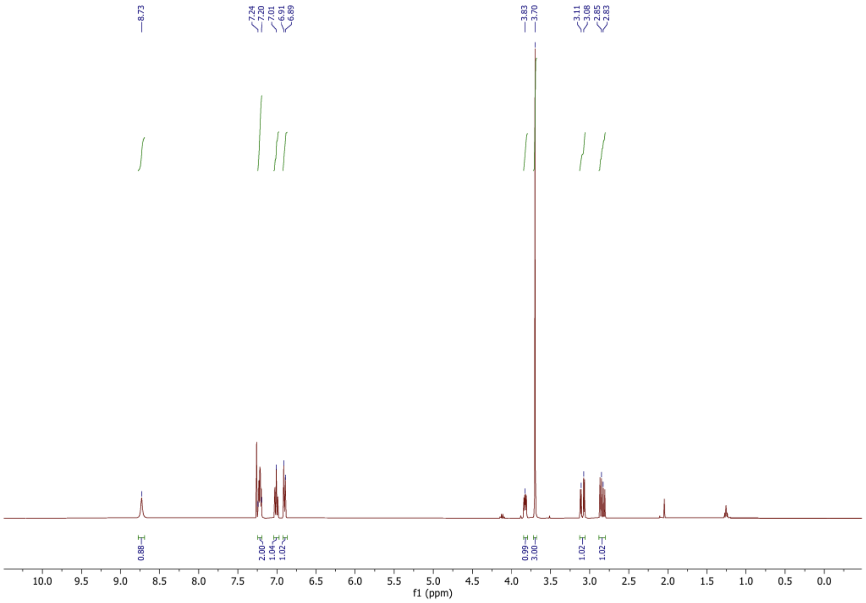

^{13}C-NMR ($CDCl_3$)

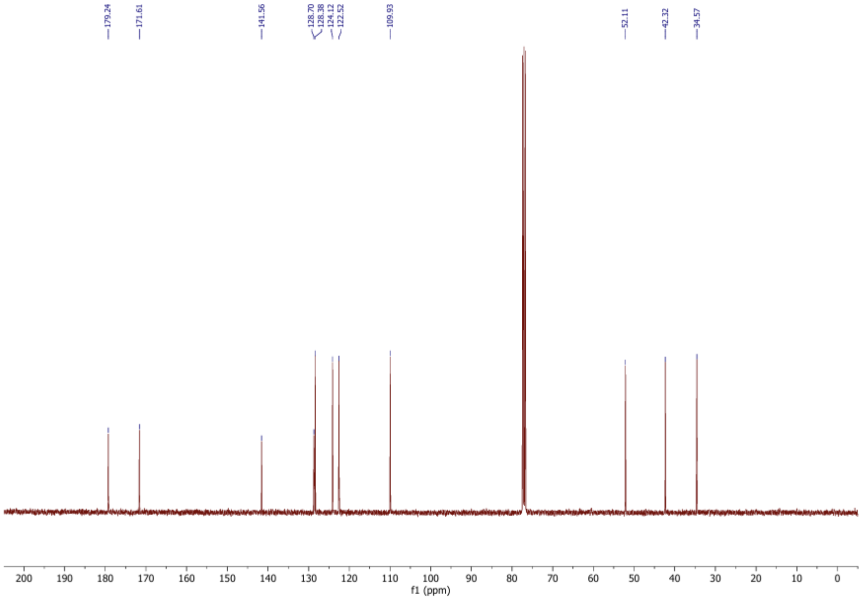

methyl 3-(2-oxoindolin-3-yl)propanoate (10)

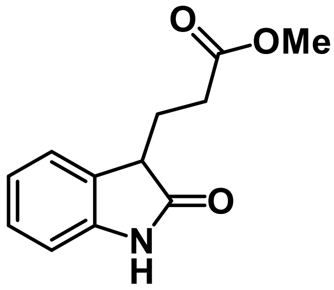

^{1}H-NMR (CDCl$_3$)

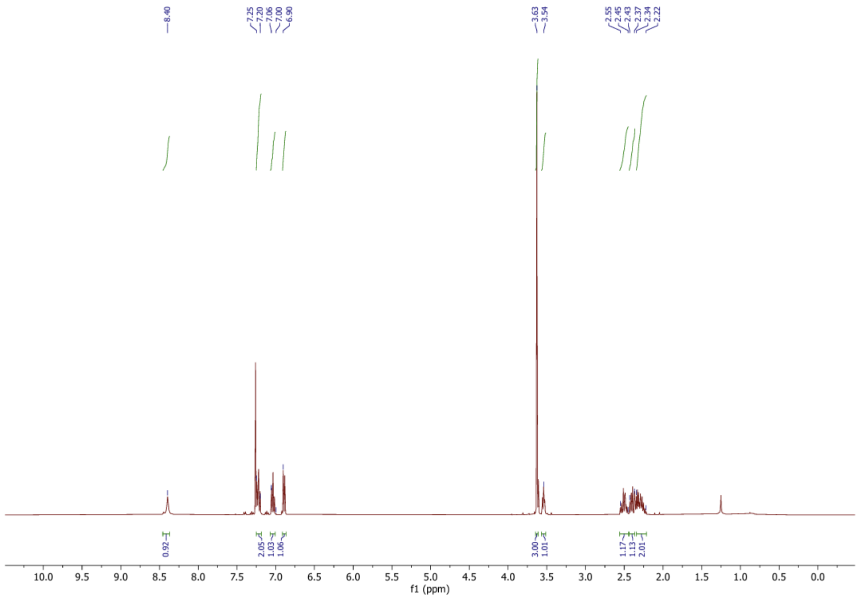

^{13}C-NMR (CDCl$_3$)

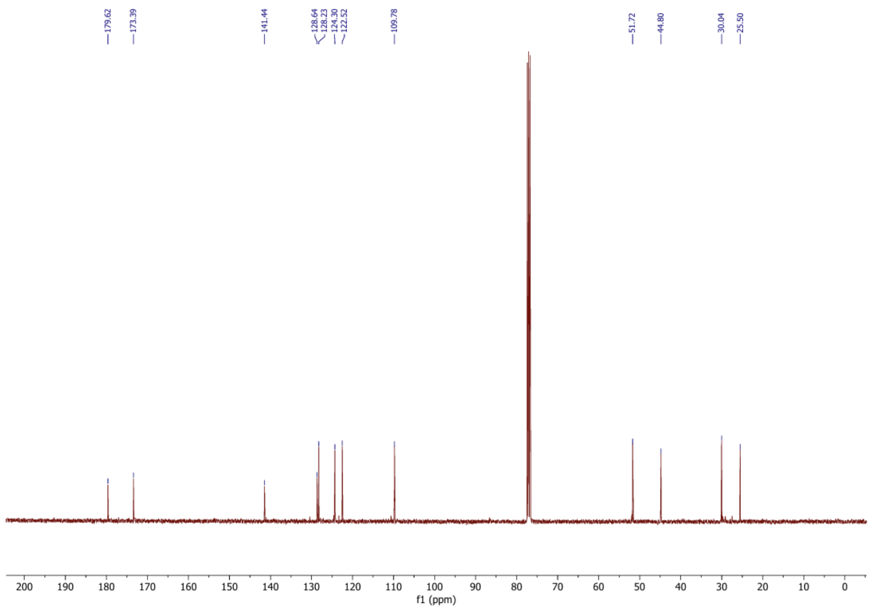

N-methoxy-N-methyl-2-(2-oxoindolin-3-yl)acetamide (11)

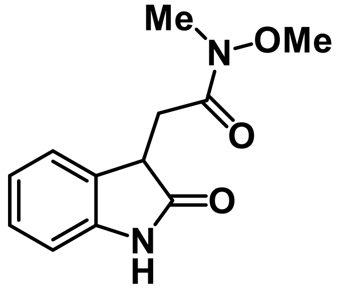

^{1}H-NMR (CDCl$_3$)

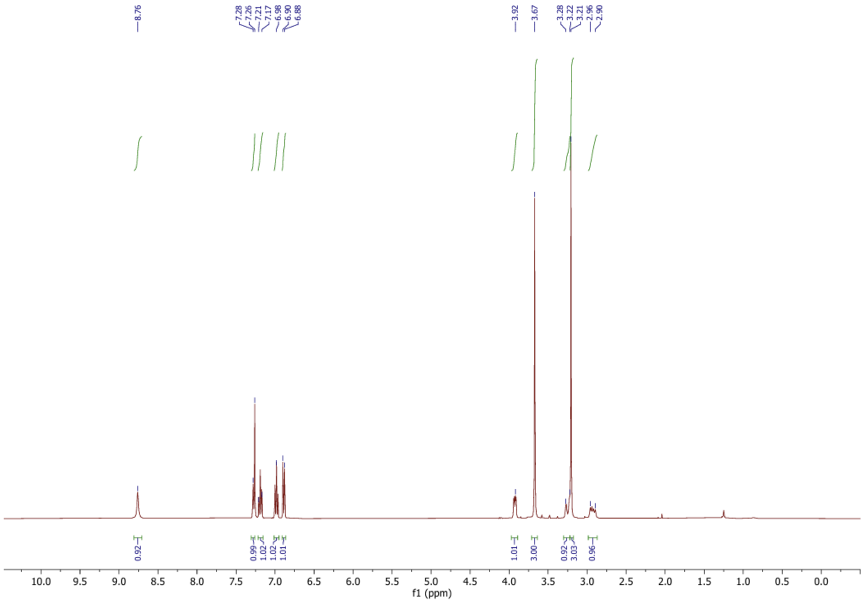

^{13}C-NMR (CDCl$_3$)

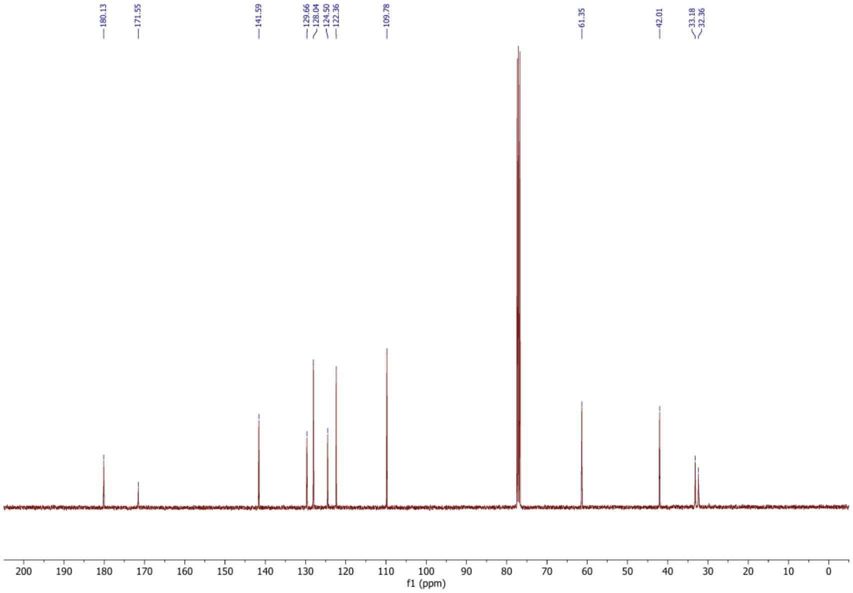

2-(2-(2-oxoindolin-3-yl)ethyl)isoindoline-1,3-dione (12)

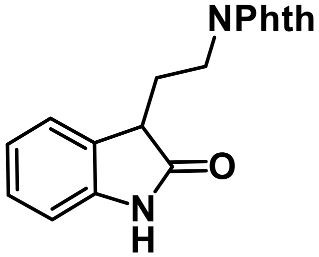

^{1}H-NMR ($CDCl_3$)

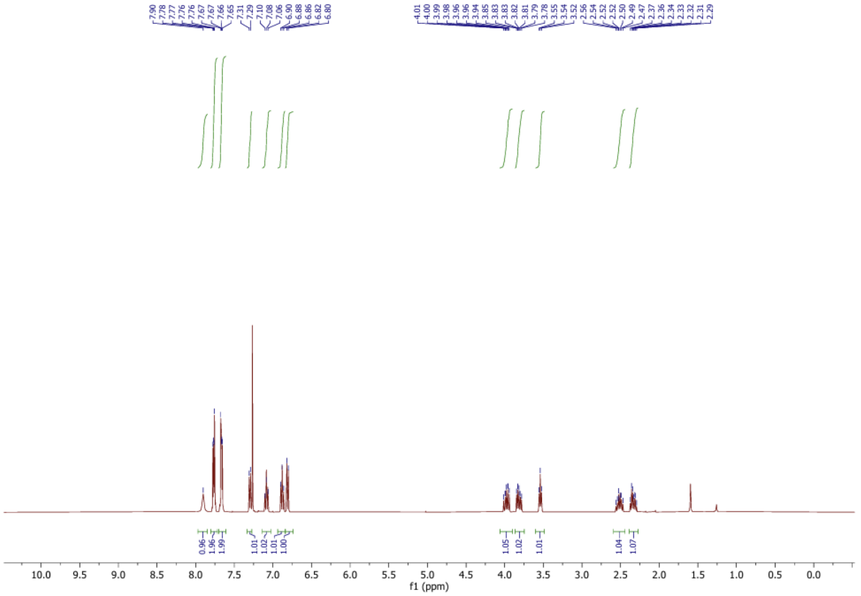

^{13}C-NMR ($CDCl_3$)

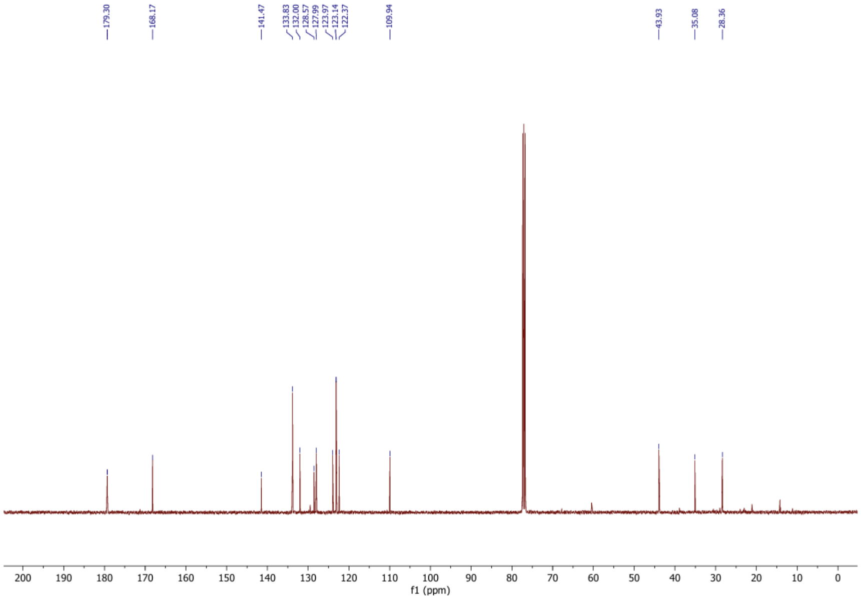

N-(2-(2-oxoindolin-3-yl)ethyl)acetamide (13)

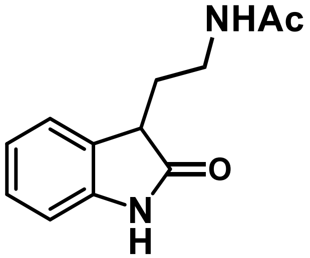

^{1}H-NMR ($CDCl_3$)

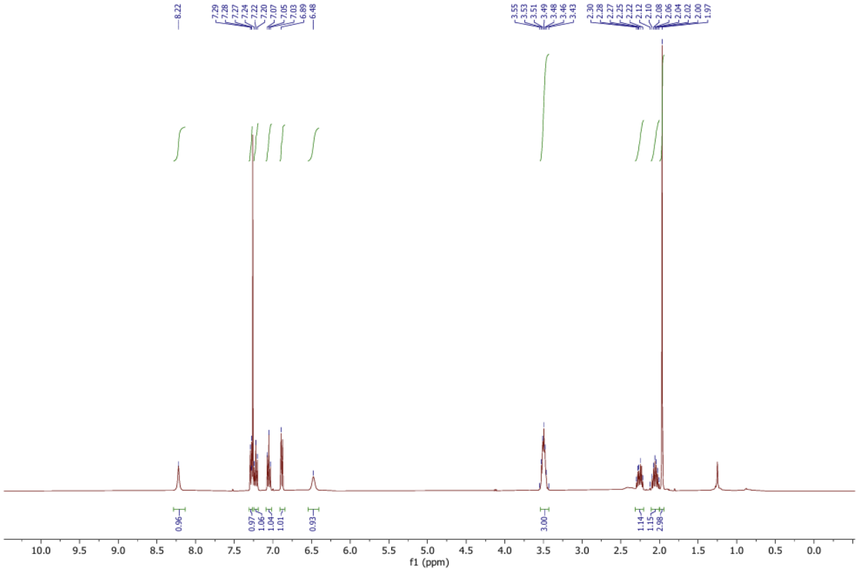

^{13}C-NMR ($CDCl_3$)

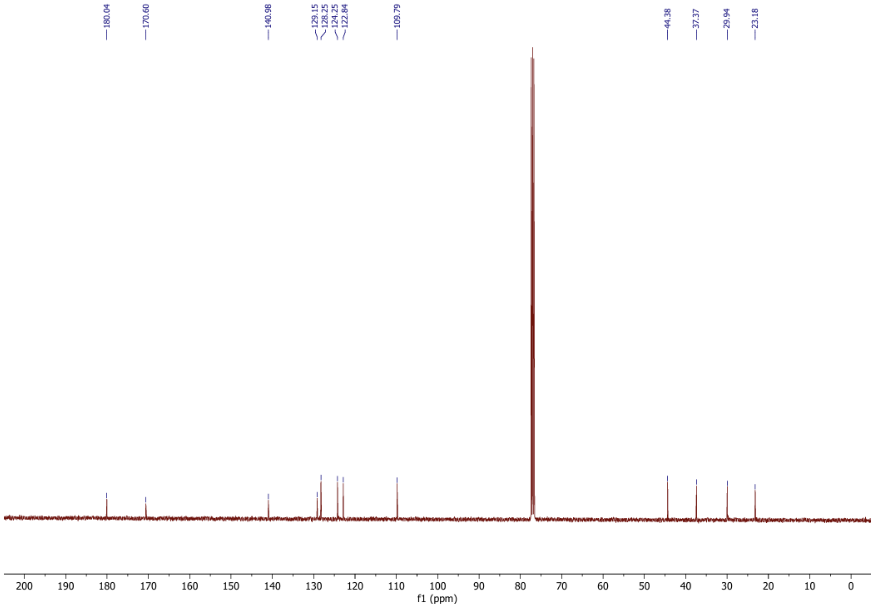

3-(2-oxopropyl)indolin-2-one (14)

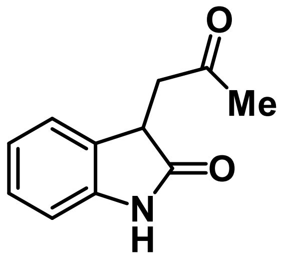

^{1}H-NMR ($CDCl_3$)

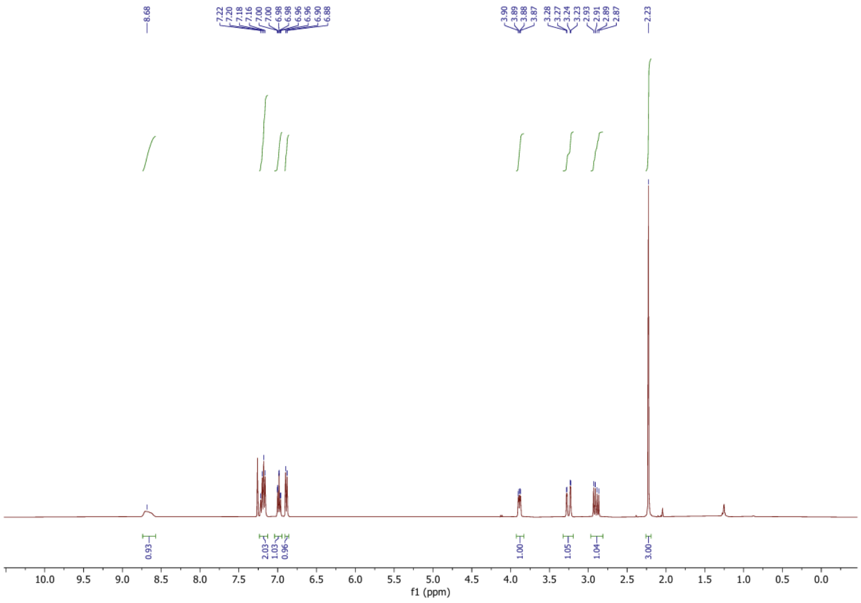

^{13}C-NMR ($CDCl_3$)

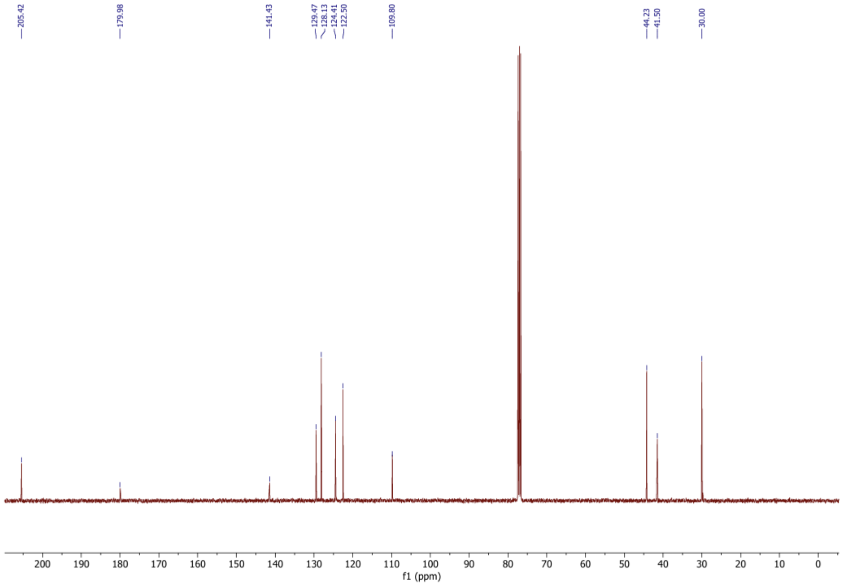

2-(2-oxoindolin-3-yl)acetonitrile (15)

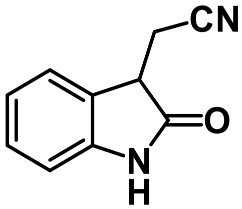

^{1}H-NMR ($CDCl_3$)

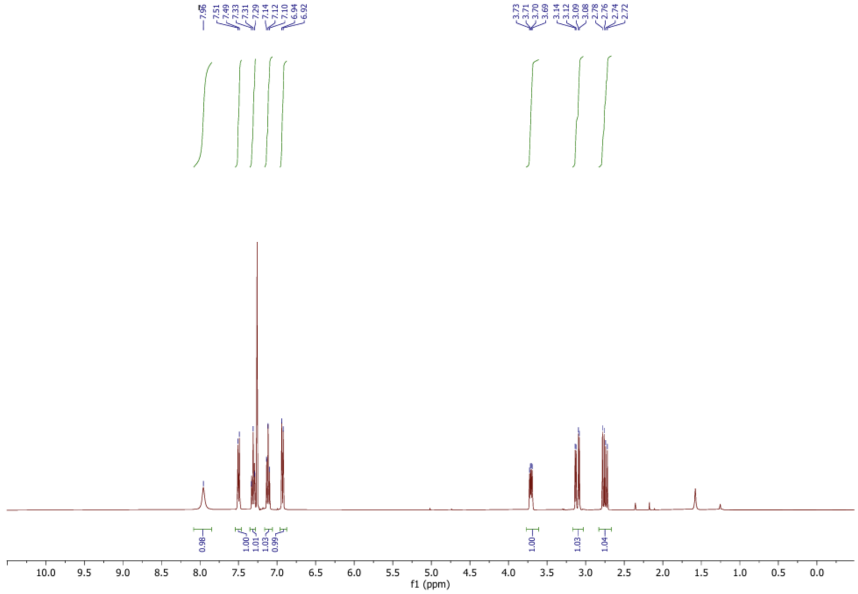

^{13}C-NMR ($CDCl_3$)

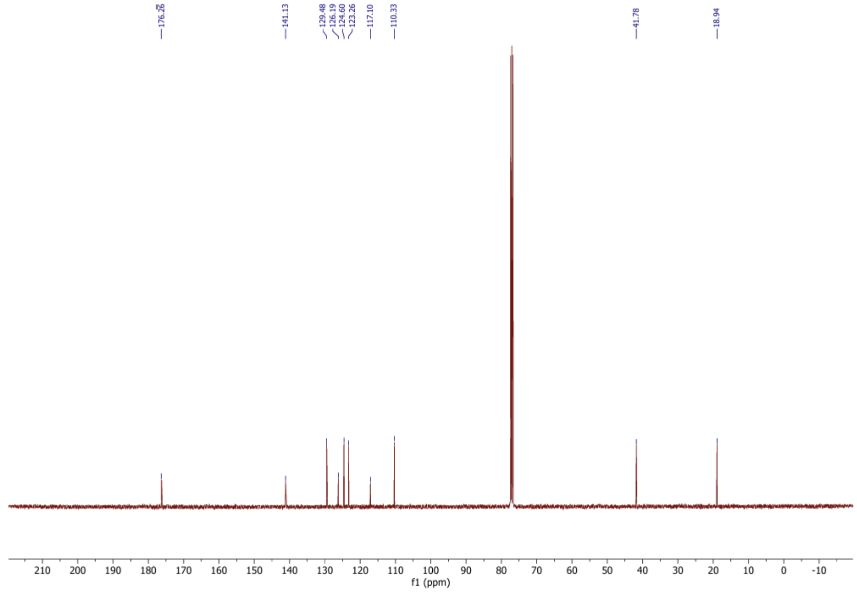

2-(5-methoxy-2-oxoindolin-3-yl)acetonitrile (16)

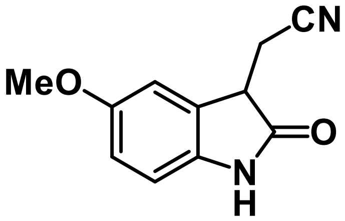

^{1}H-NMR ($CDCl_3$)

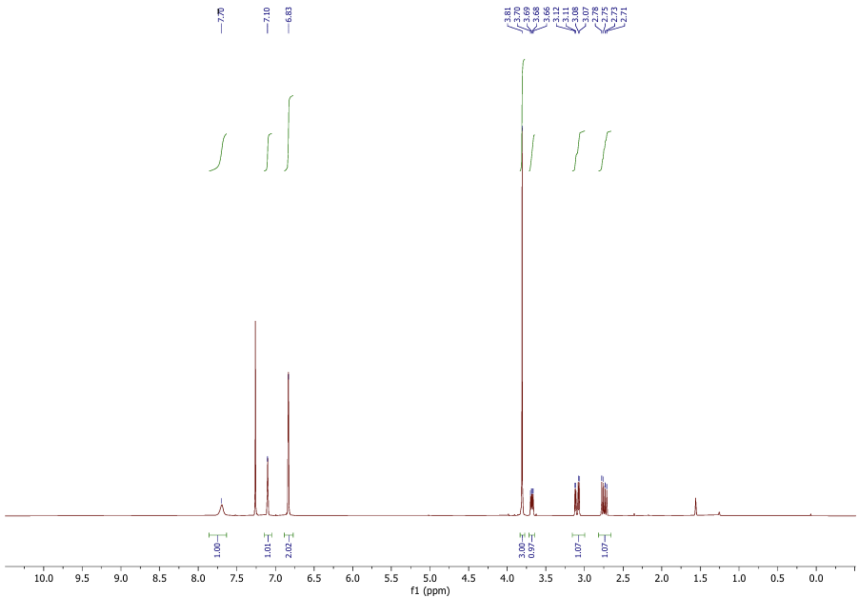

^{13}C-NMR ($CDCl_3$)

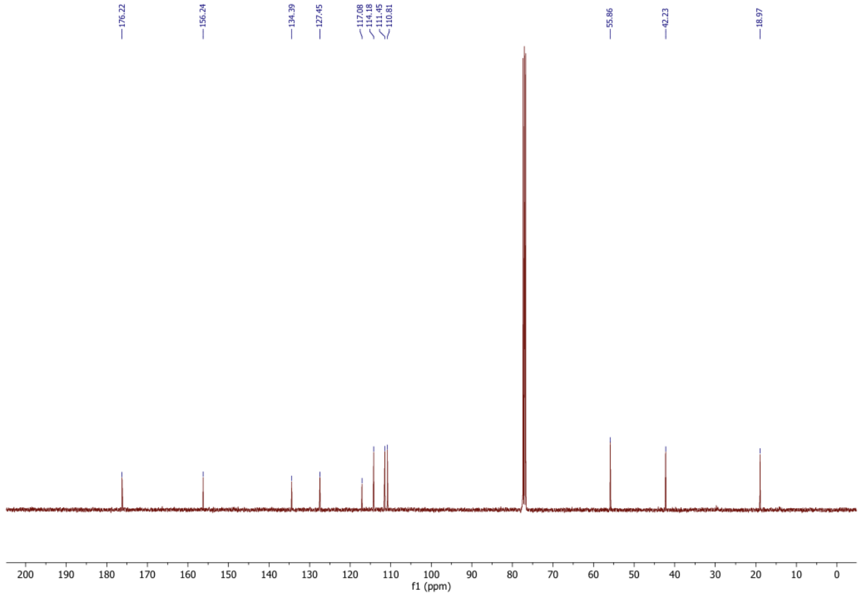

3-benzylindolin-2-one (17)

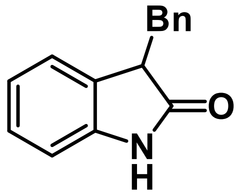

^{1}H-NMR ($CDCl_3$)

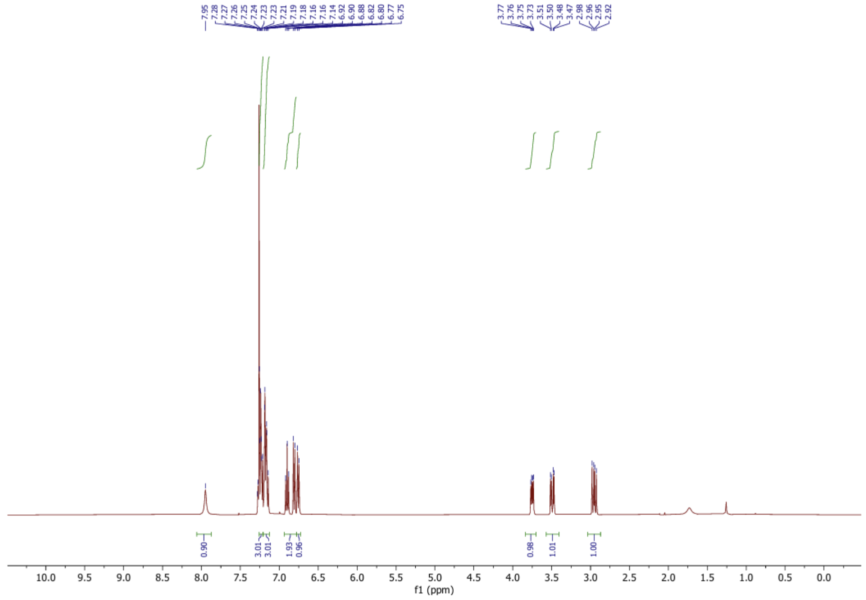

^{13}C-NMR ($CDCl_3$)

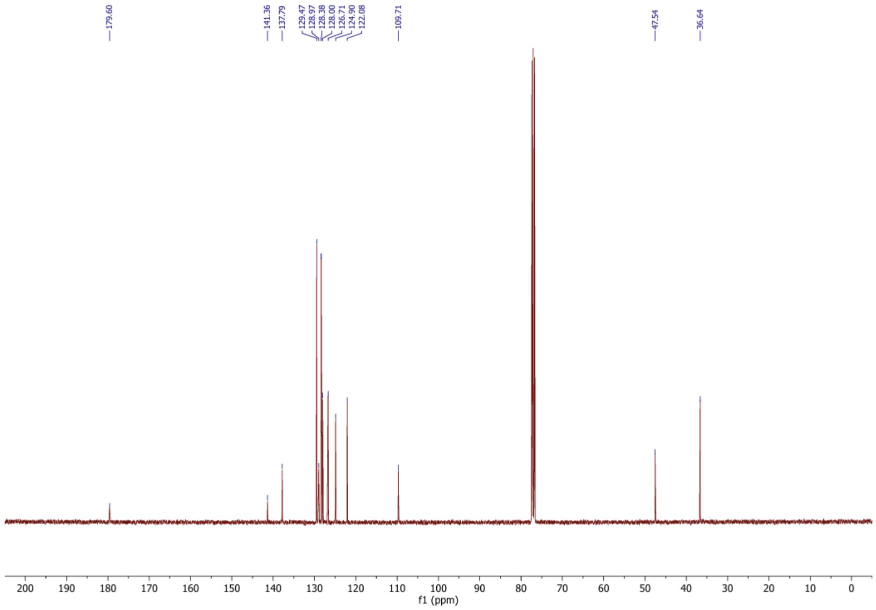

3-phenylindolin-2-one (18)

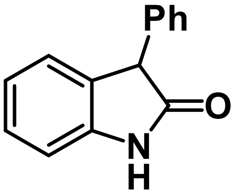

^{1}H-NMR (CDCl$_3$)

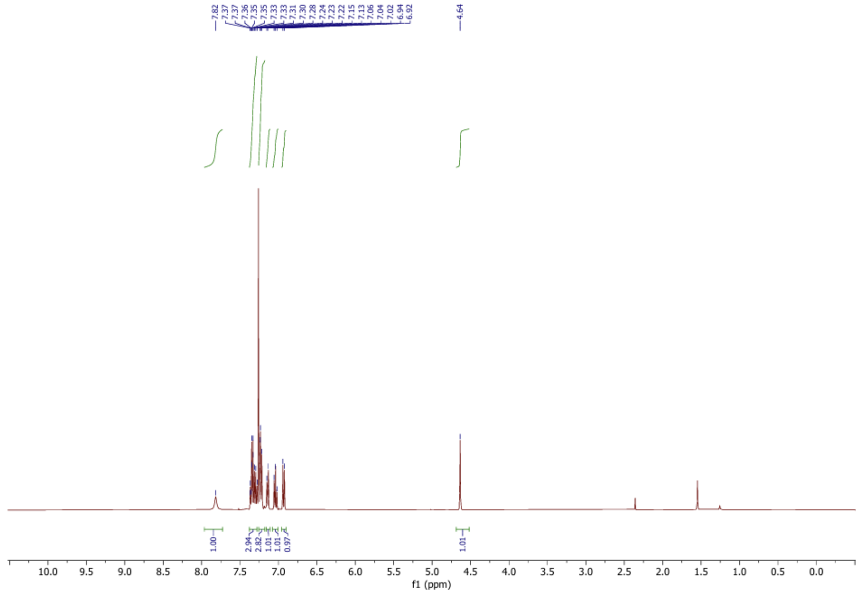

^{13}C-NMR (CDCl$_3$)

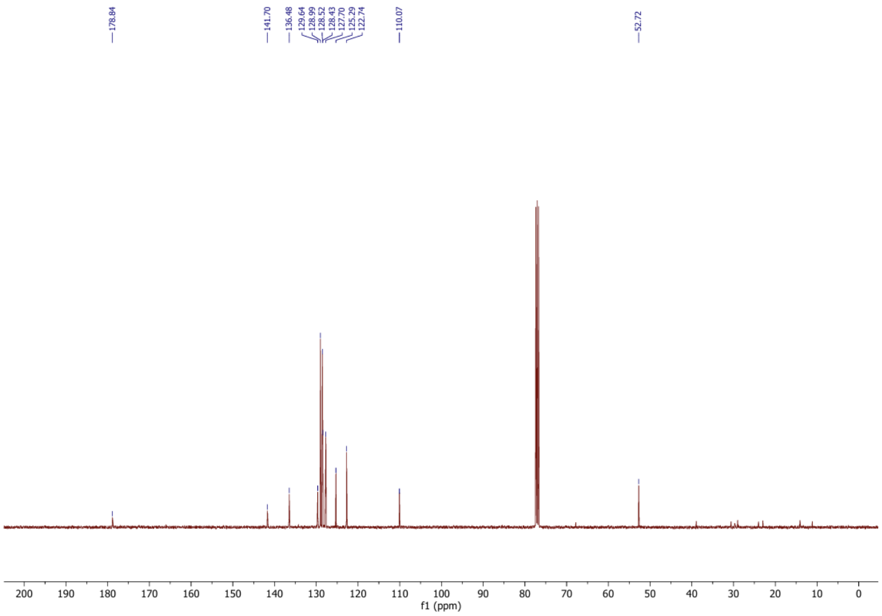

1,3-dimethylindolin-2-one (19)

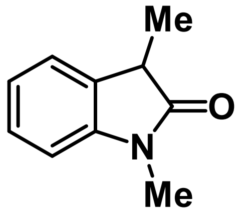

^{1}H-NMR ($CDCl_3$)

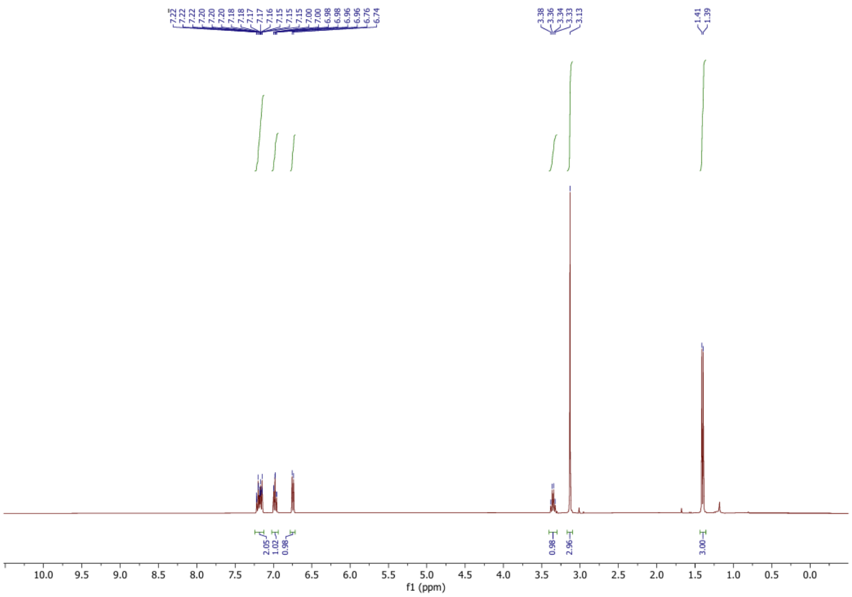

^{13}C-NMR ($CDCl_3$)

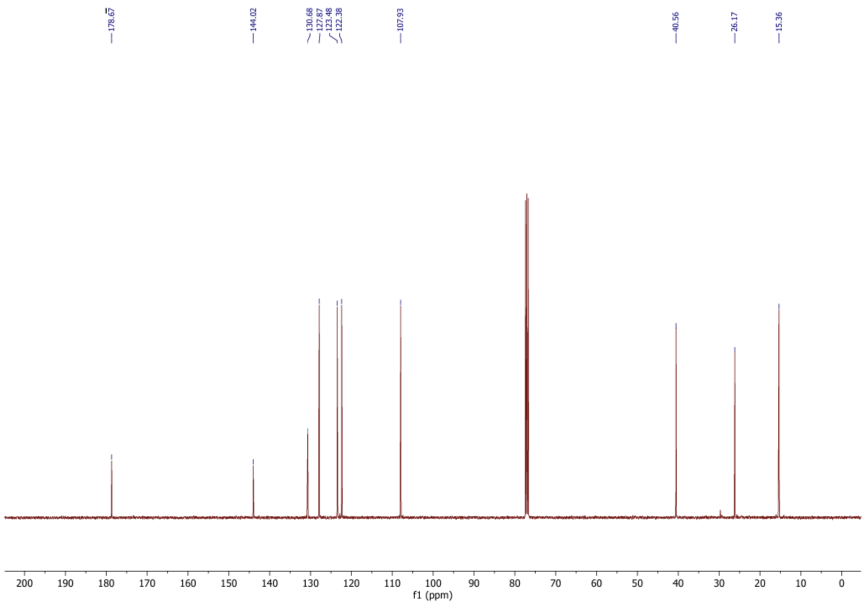

N-(2-(5-methoxy-2-oxoindolin-3-yl)ethyl)acetamide (20)

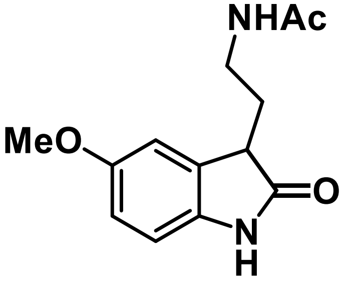

^{1}H-NMR ($CDCl_3$)

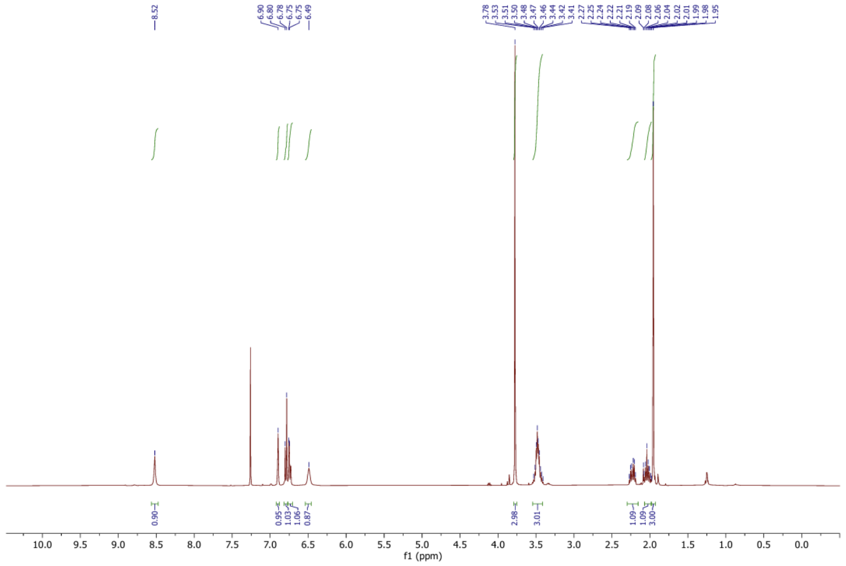

^{13}C-NMR ($CDCl_3$)

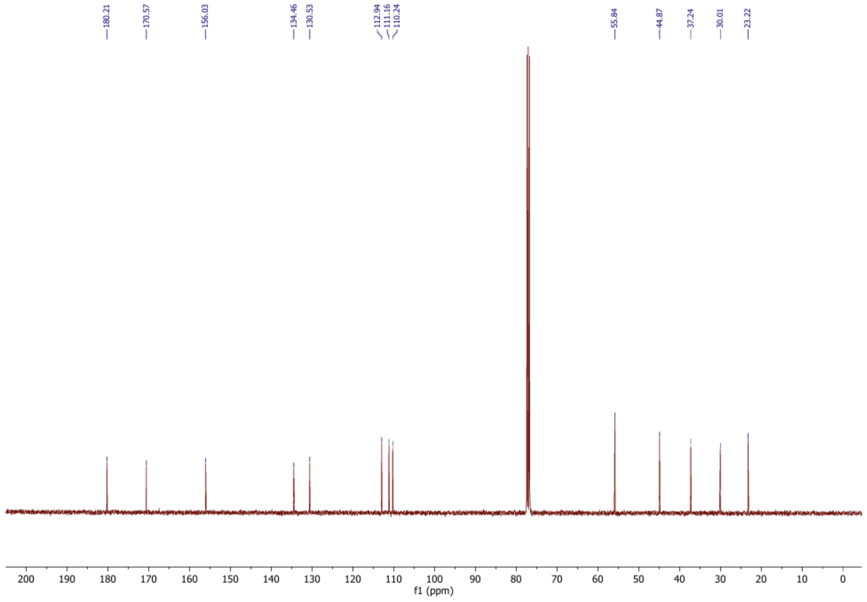

1'-acetylspiro[indoline-3,3'-pyrrolidin]-2-one (4)

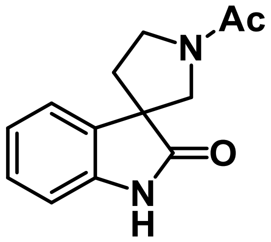

^{1}H-NMR (DMSO-d6)

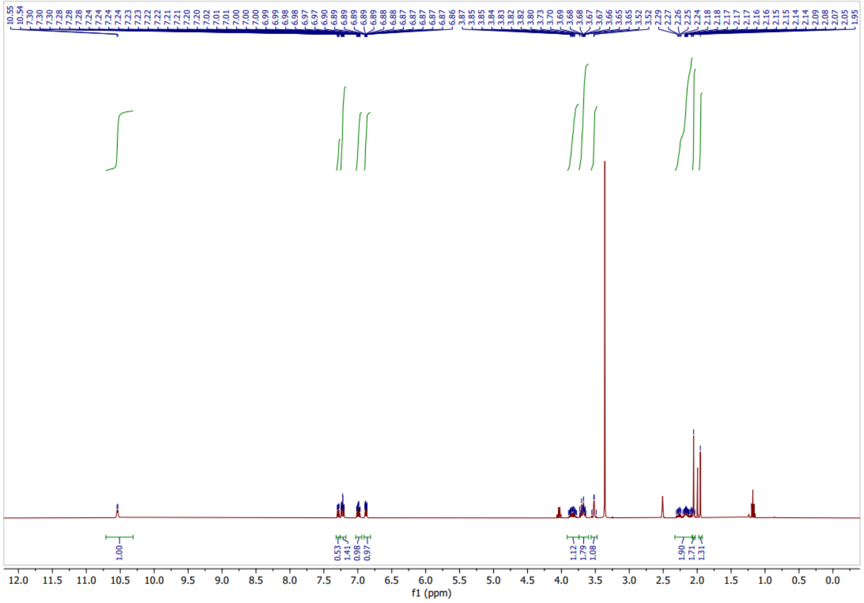

^{13}C-NMR (DMSO-d6)

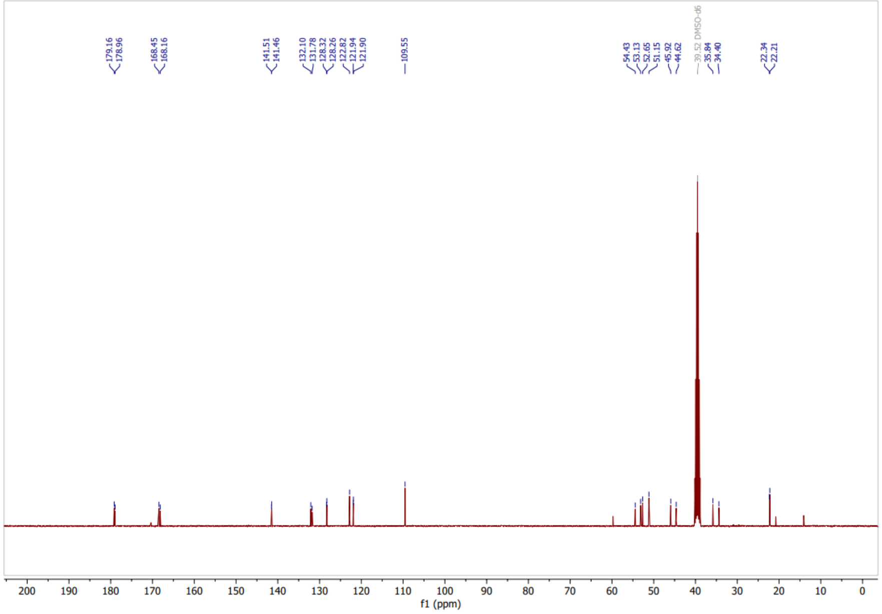

tert-butyl 2-oxospiro[indoline-3,3'-pyrrolidine]-1'-carboxylate (21)

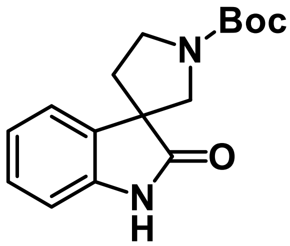

^{1}H-NMR ($CDCl_3$)

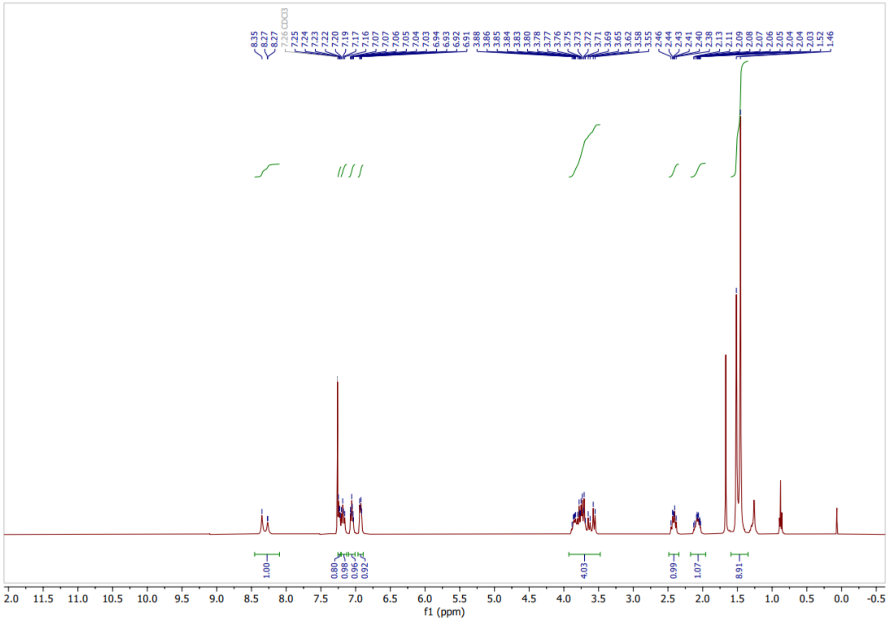

^{13}C-NMR ($CDCl_3$)

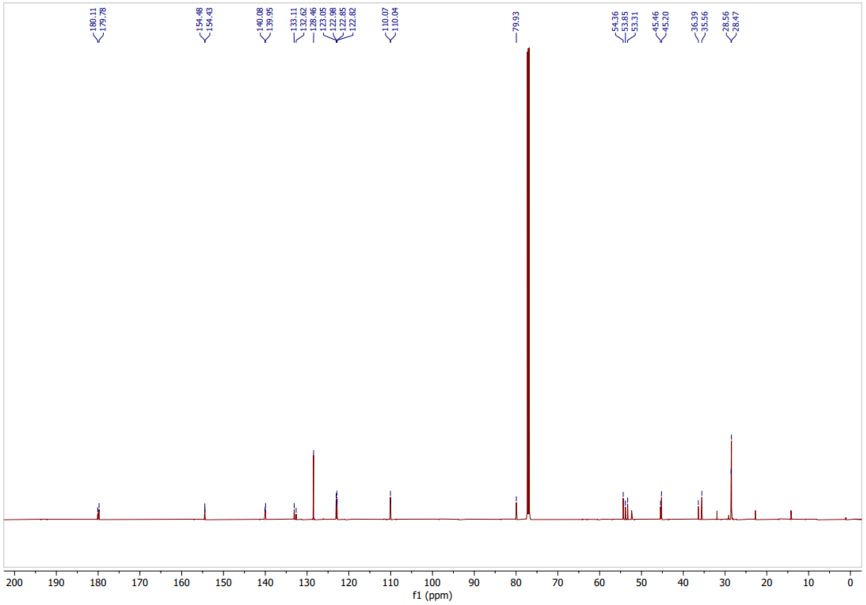

2,2,2-trichloroethyl 2-oxospiro[indoline-3,3'-pyrrolidine]-1'-carboxylate (22)

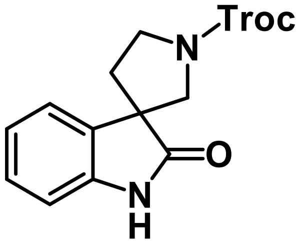

^{1}H-NMR ($CDCl_3$)

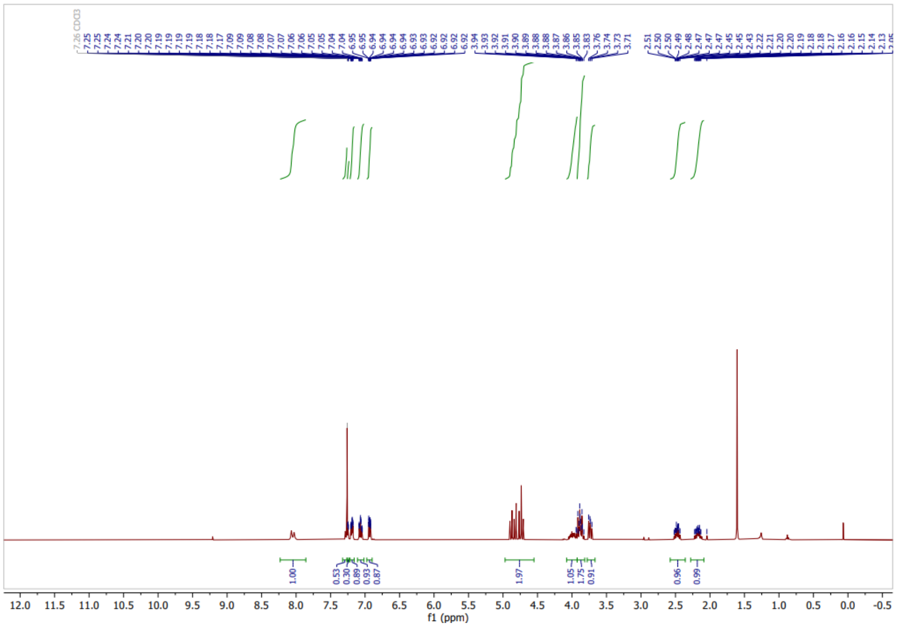

^{13}C-NMR ($CDCl_3$)

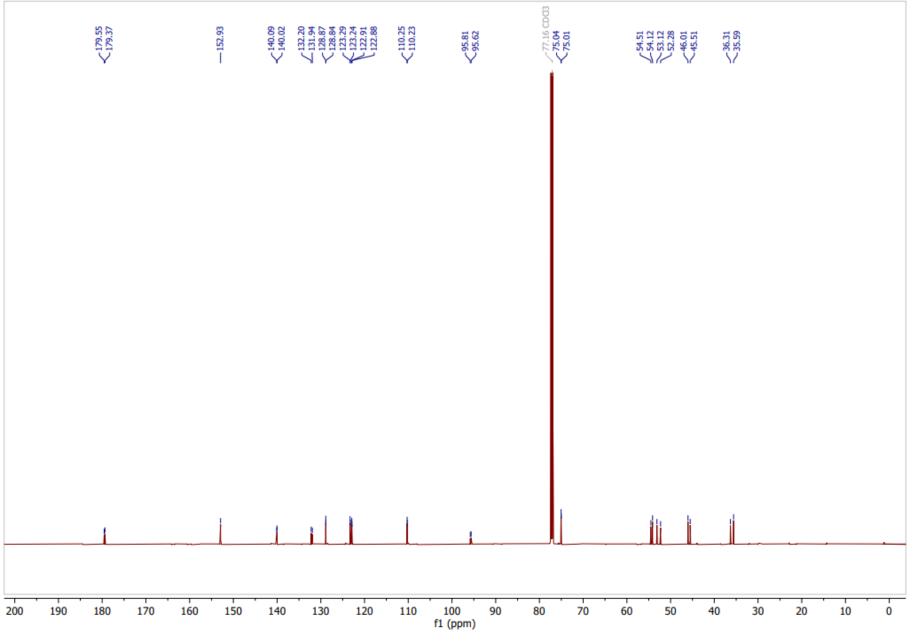

methyl 2-oxospiro[indoline-3,3'-pyrrolidine]-1'-carboxylate (23)

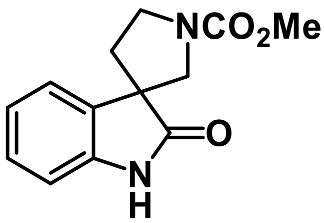

^{1}H-NMR (DMSO-d6)

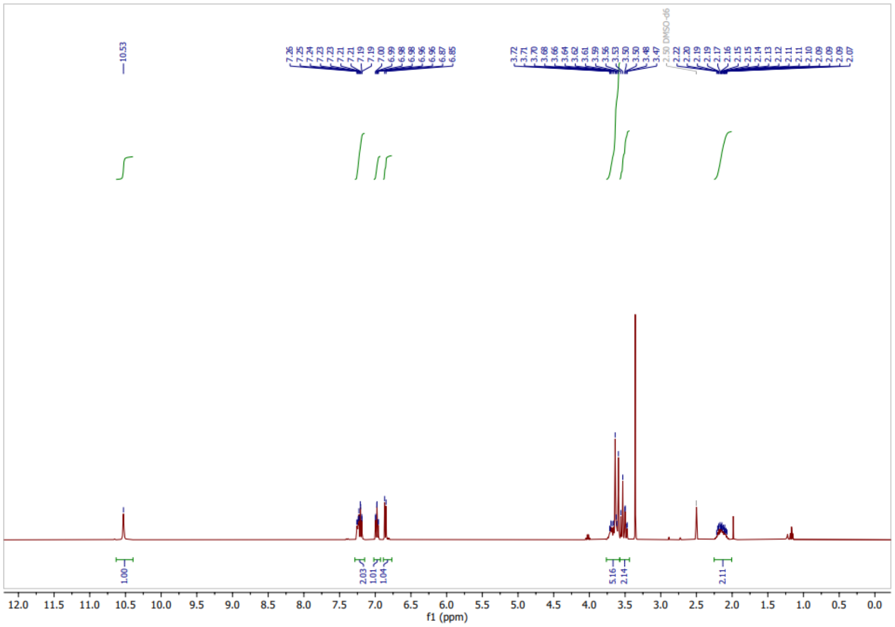

^{13}C-NMR (DMSO-d6)

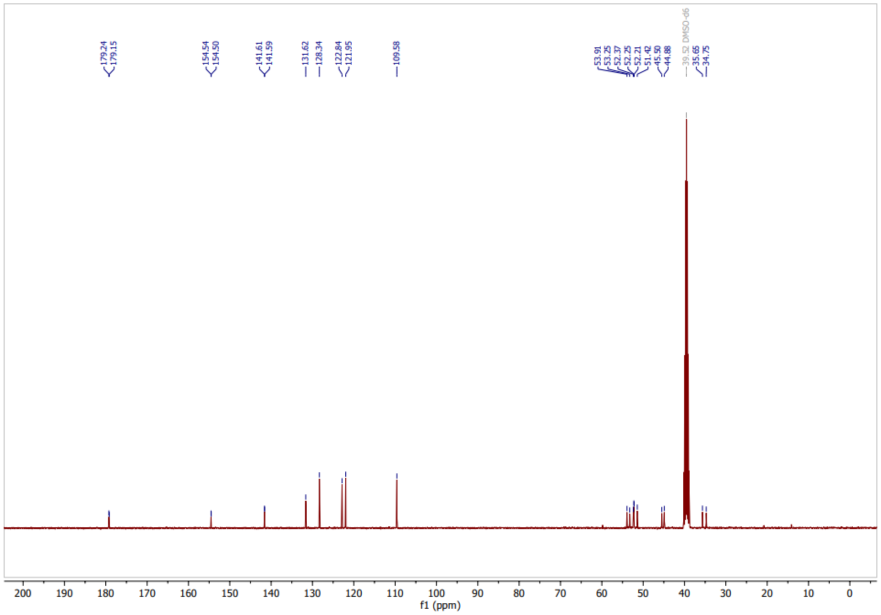

1'-acetyl-1-methylspiro[indoline-3,3'-pyrrolidin]-2-one (24)

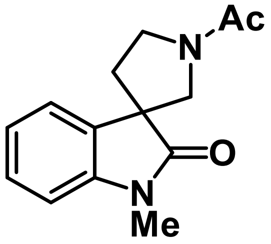

^{1}H-NMR (DMSO-d6)

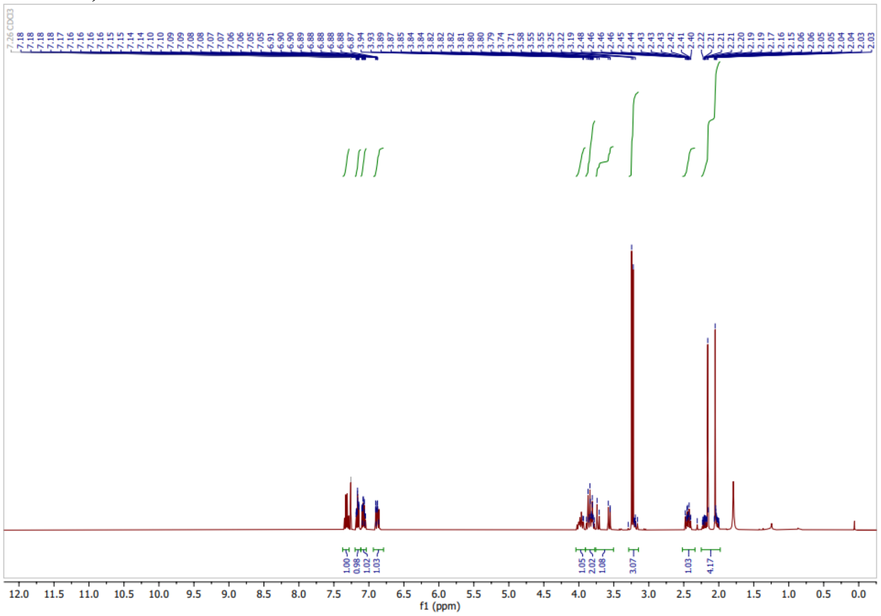

^{13}C-NMR (DMSO-d6)

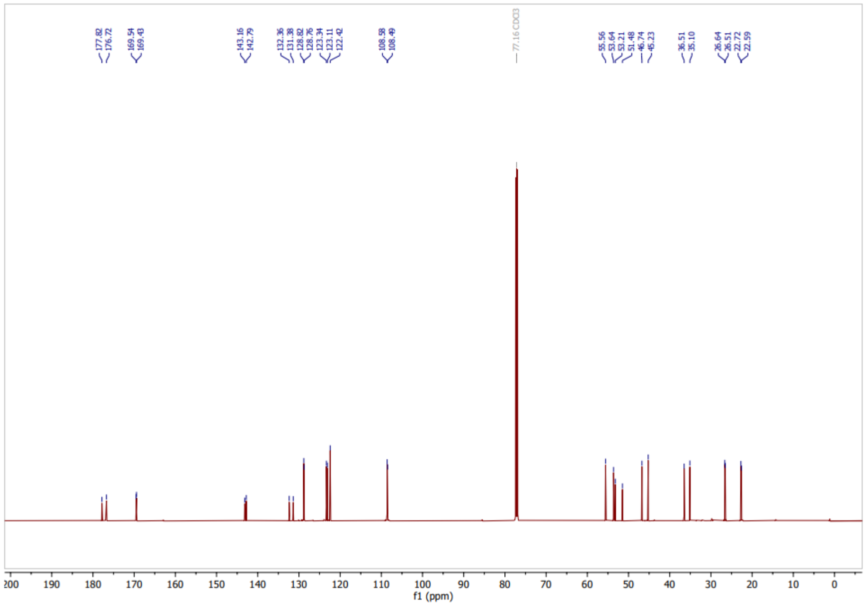

dimethyl (5'S)-2-oxospiro[indoline-3,3'-pyrrolidine]-1',5'-dicarboxylate (25)

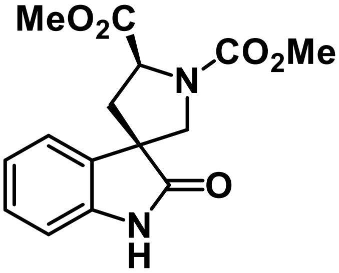

^{1}H-NMR (DMSO-d6)

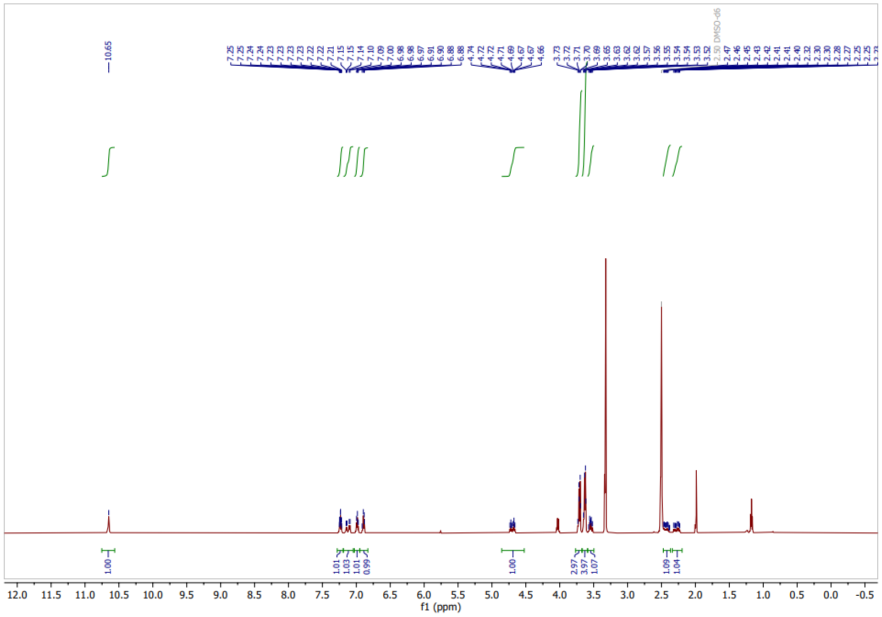

^{13}C-NMR (DMSO-d6)

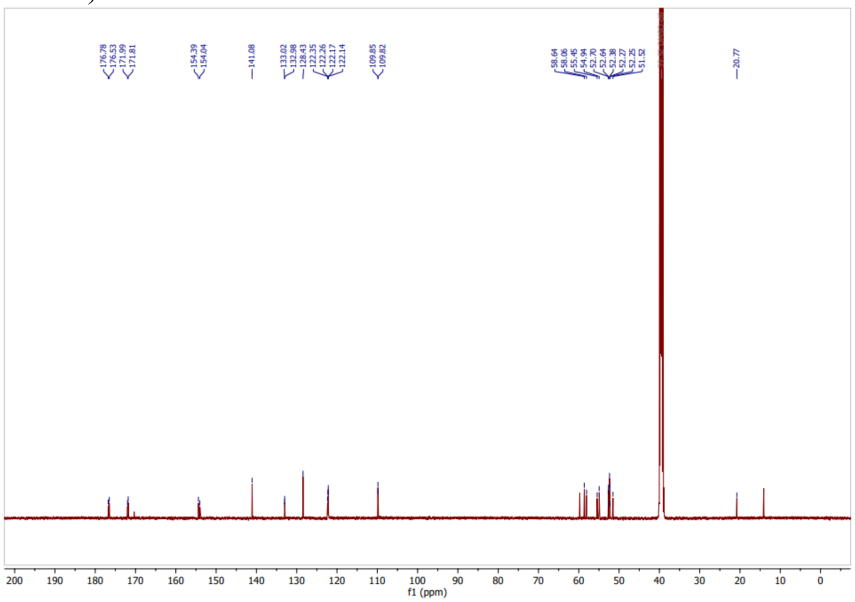

2,2-dimethyl-4,5-dihydro-2H-spiro[furan-3,3'-indolin]-2'-one (26)

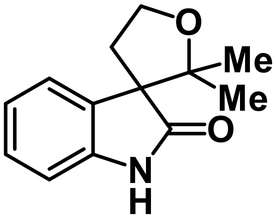

^{1}H-NMR (DMSO-d6)

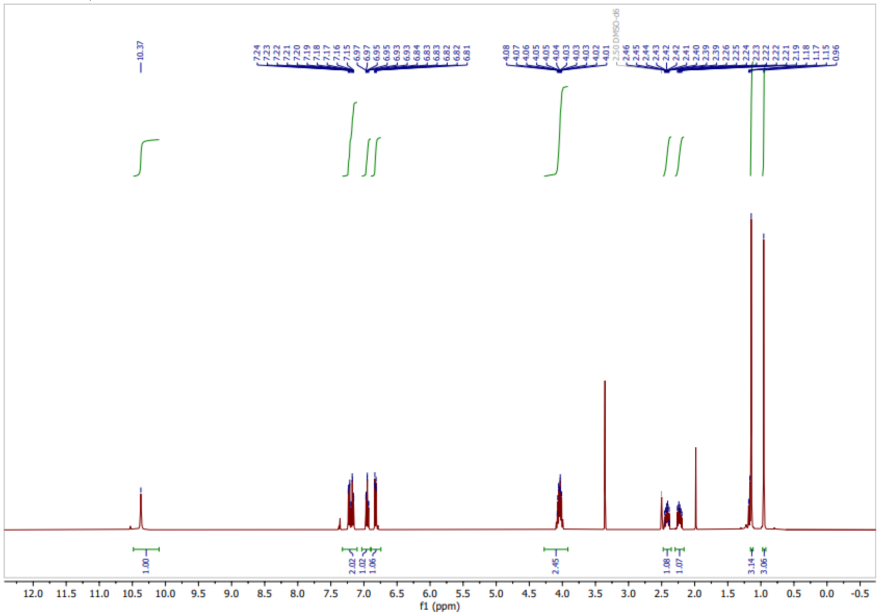

^{13}C-NMR (DMSO-d6)

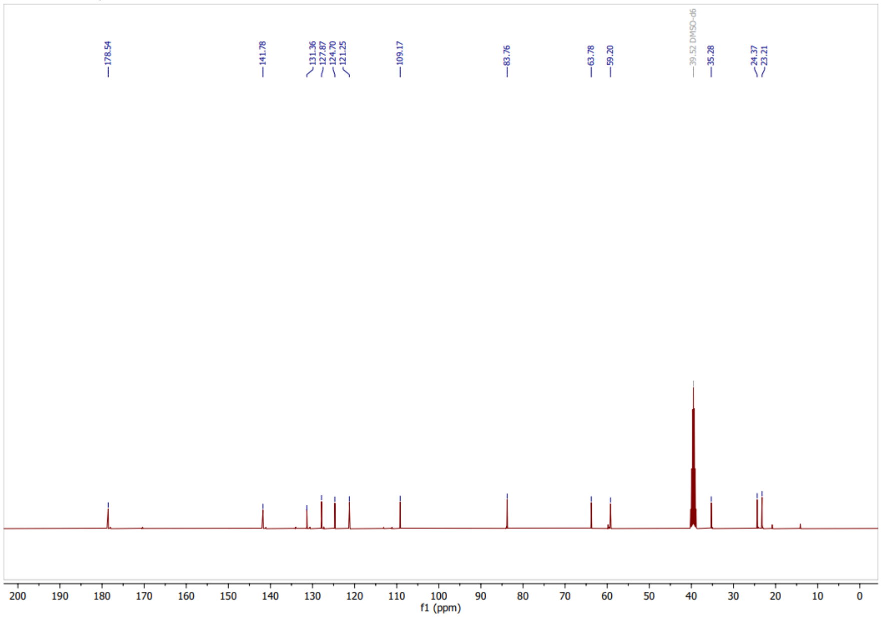

4',5'-dihydrodispiro[cyclohexane-1,2'-furan-3',3''-indolin]-2''-one (27)

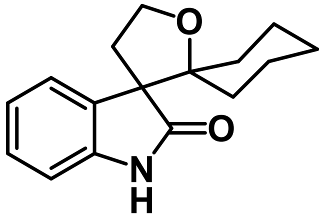

^{1}H-NMR ($CDCl_3$)

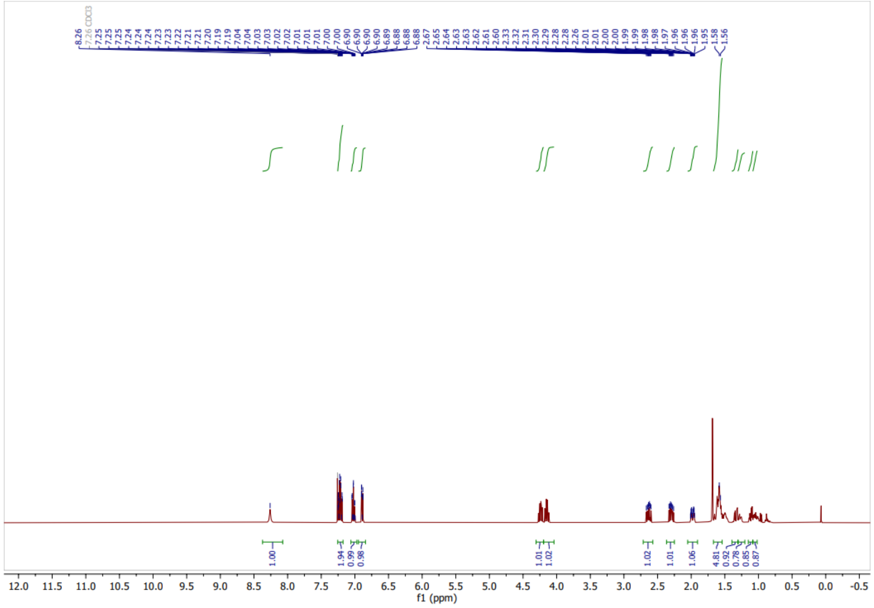

^{13}C-NMR ($CDCl_3$)

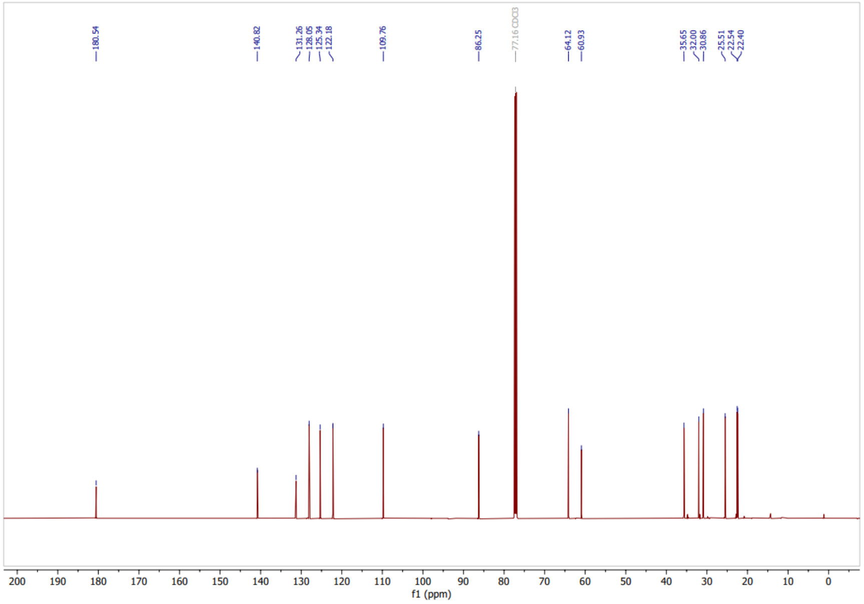

tert-butyl 2-oxo-4',5'-dihydrodispiro[indoline-3,3'-furan-2',4''-piperidine]-1''-carboxylate (28)

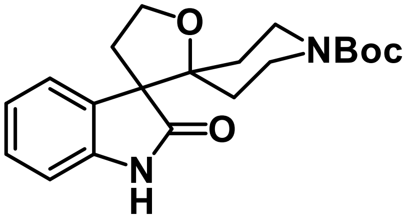

^{1}H-NMR (DMSO-d6)

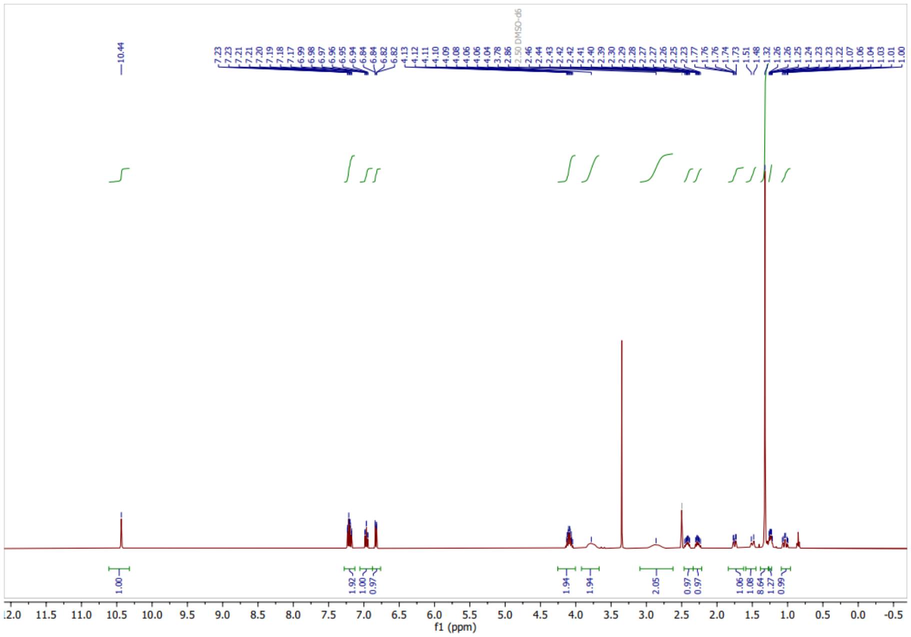

^{13}C-NMR (DMSO-d6)

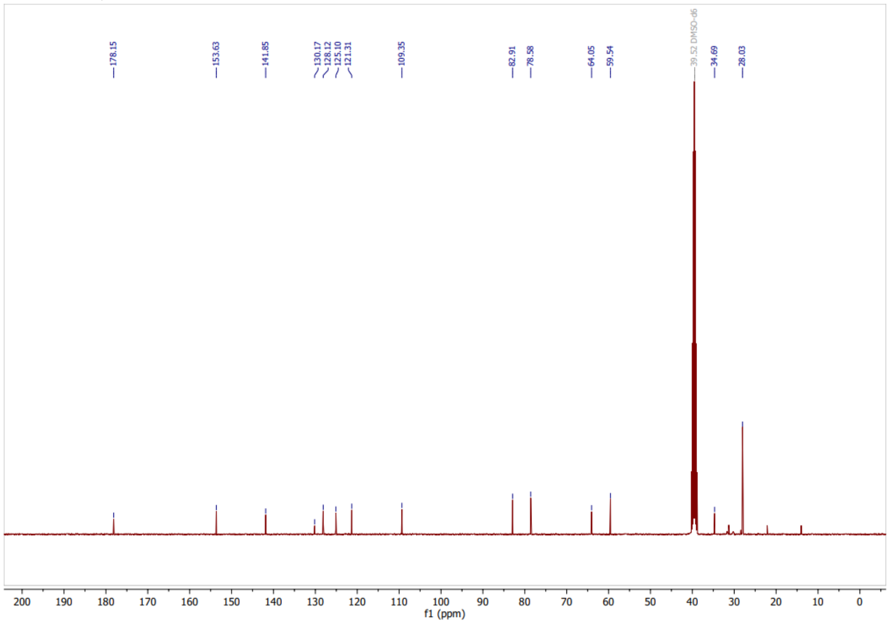

3-(hydroxymethyl)-3-methylindolin-2-one (29)

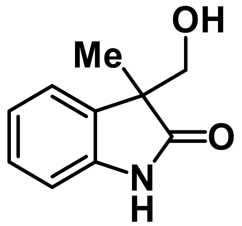

^{1}H-NMR (DMSO-d6)

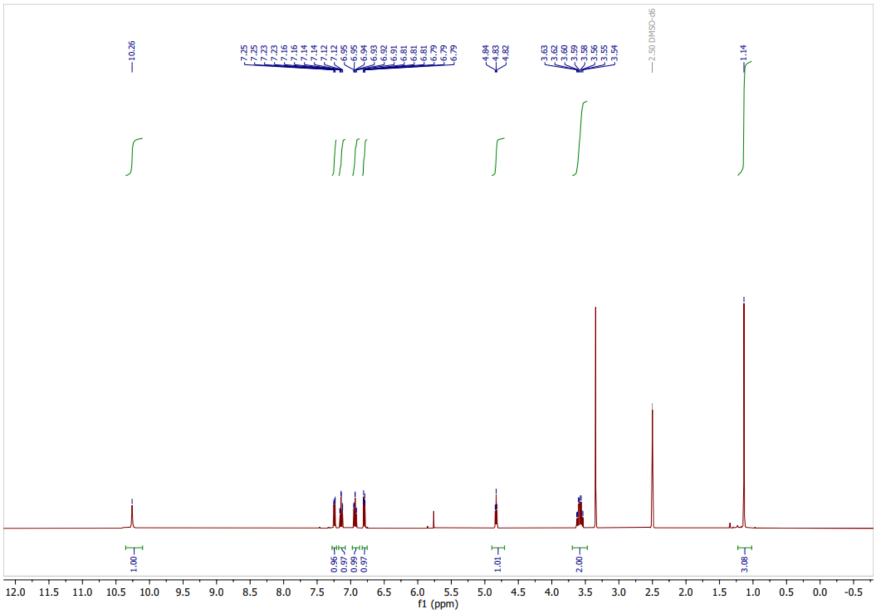

^{13}C-NMR (DMSO-d6)

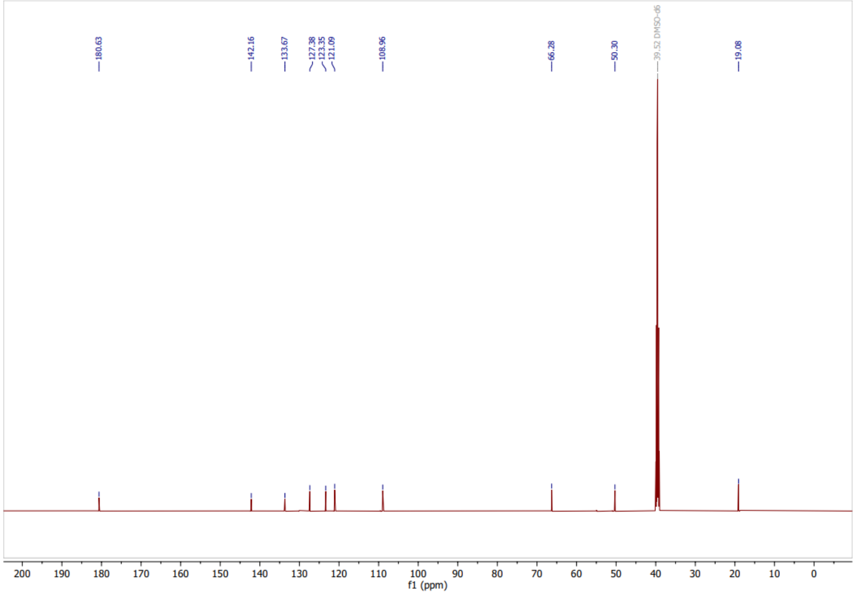

ethyl 3-methyl-2-oxoindoline-3-carboxylate (30)

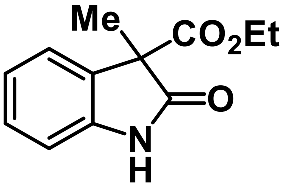

^{1}H-NMR (DMSO-d6)

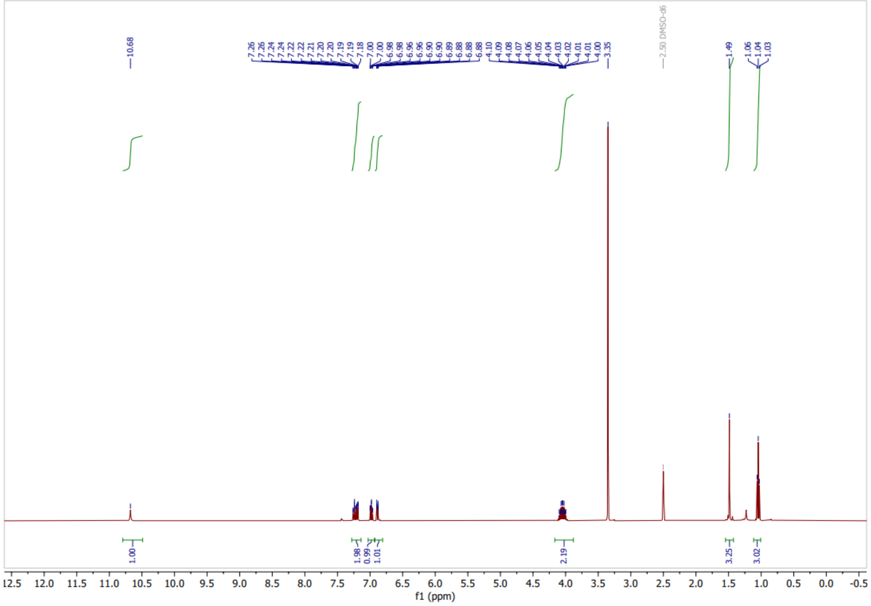

^{13}C-NMR (DMSO-d6)

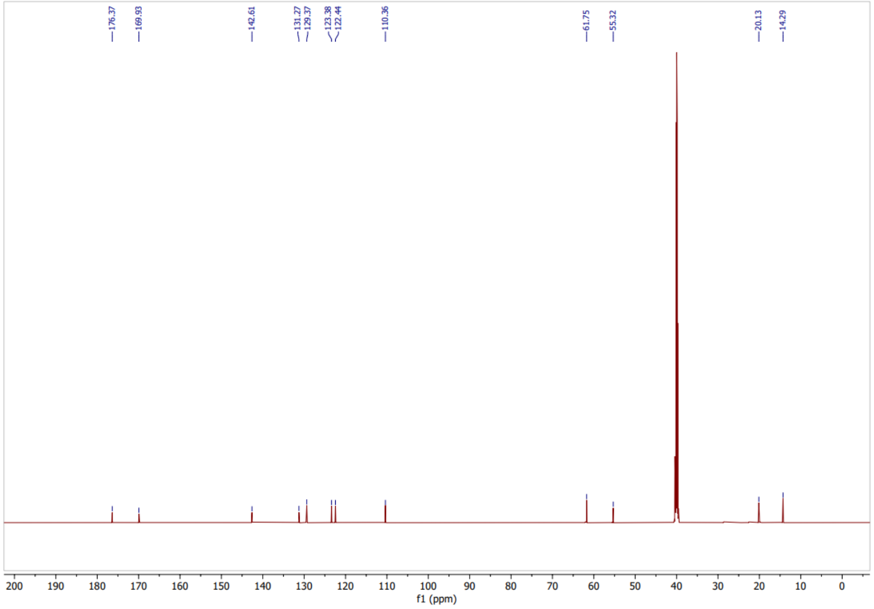

2-(2-oxoindolin-3-yl)acetic acid (33)

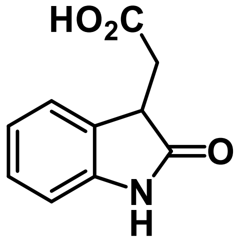

^{1}H-NMR (DMSO-d6)

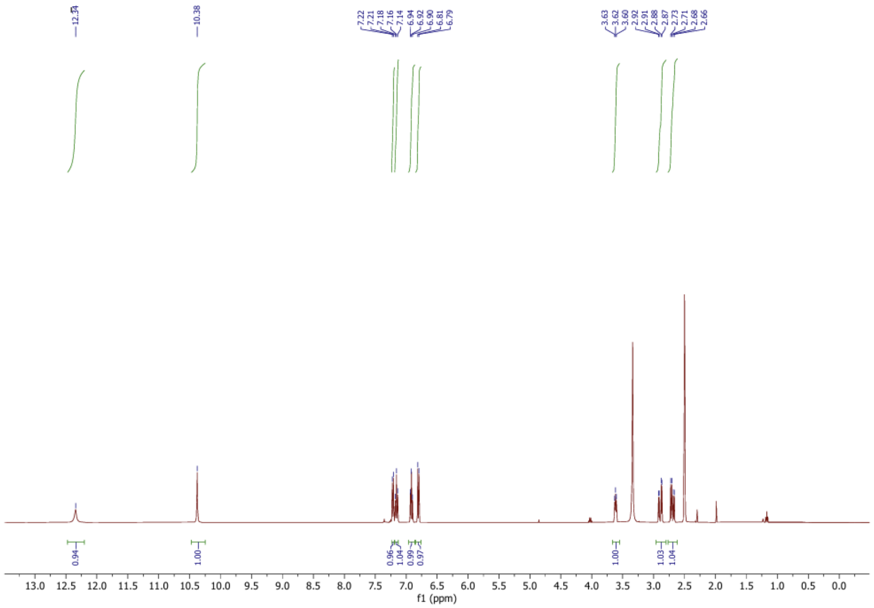

^{13}C-NMR (DMSO-d6)

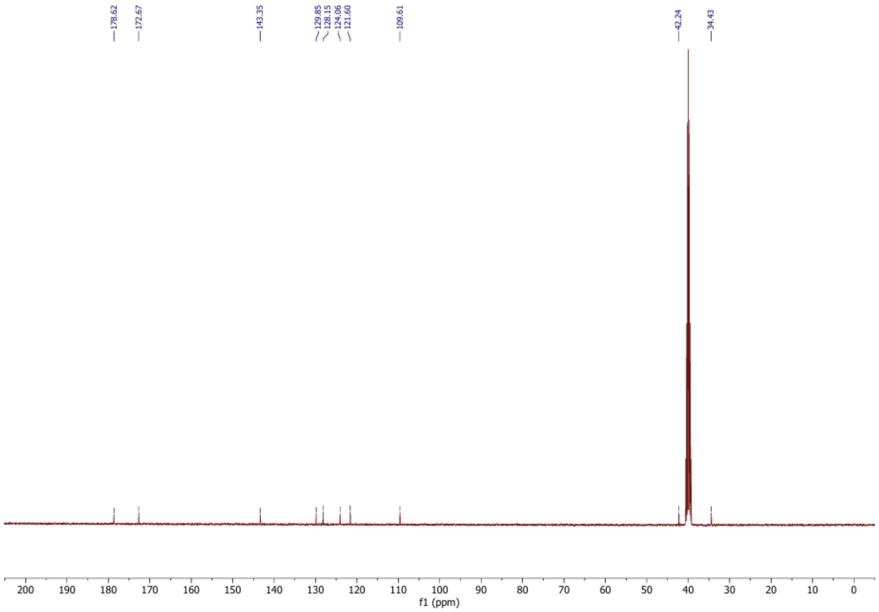

methyl 2-phenylquinoline-4-carboxylate (35)

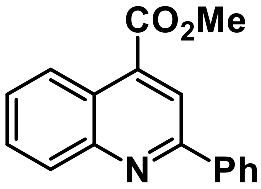

^{1}H-NMR ($CDCl_3$)

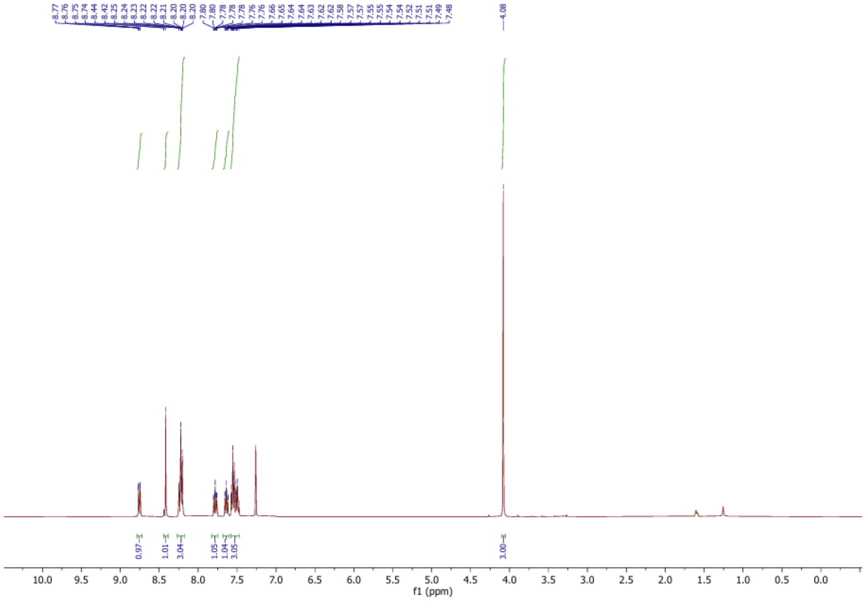

^{13}C-NMR ($CDCl_3$)

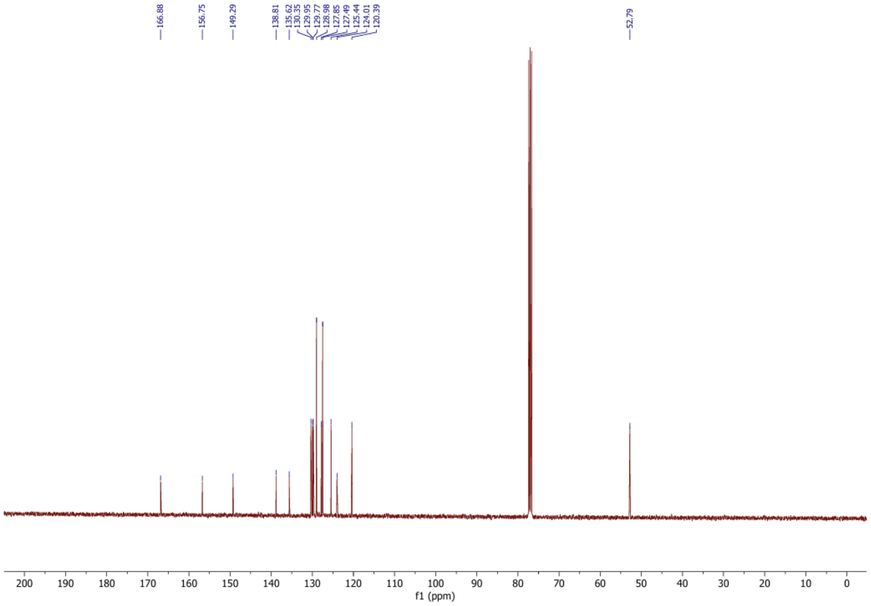

methyl (5'S)-2-oxospiro[indoline-3,3'-pyrrolidine]-5'-carboxylate (37)

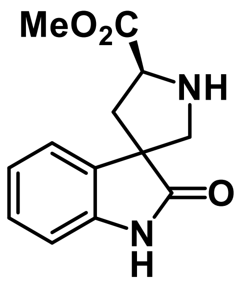

^{1}H-NMR ($CDCl_3$)

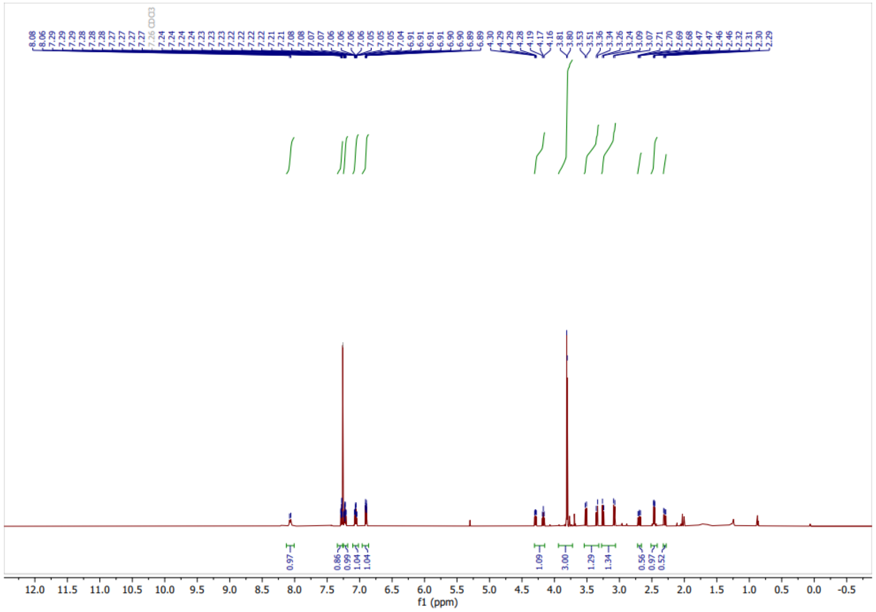

^{13}C-NMR ($CDCl_3$)

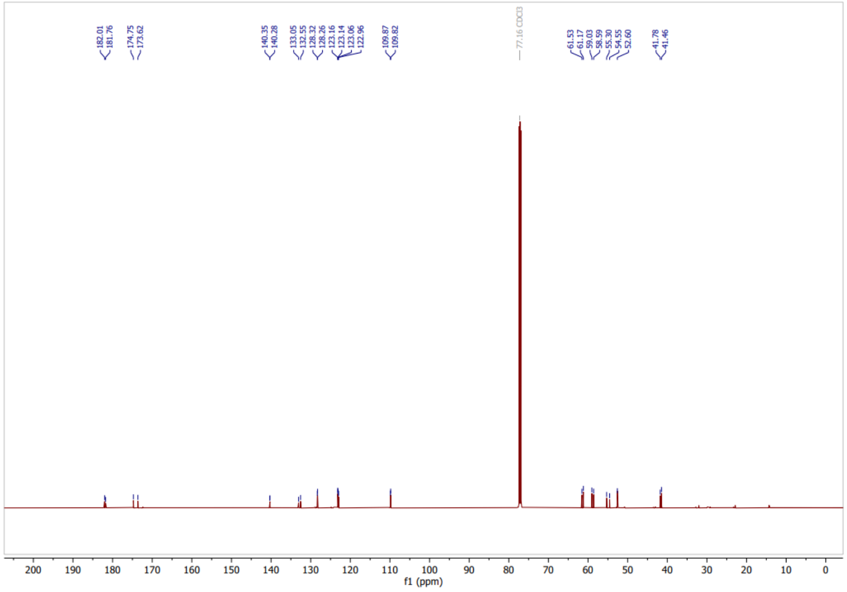

methyl 9H-pyrido[3,4-b]indole-3-carboxylate (38)

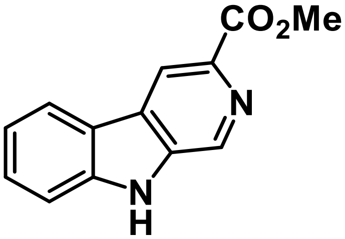

^{1}H-NMR (DMSO-d6)

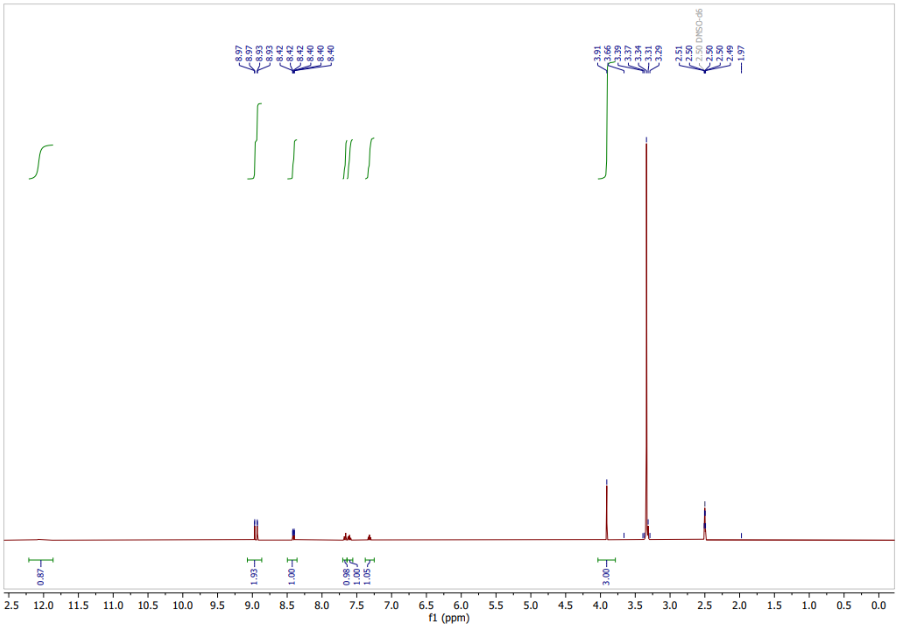

^{13}C-NMR (DMSO-d6)

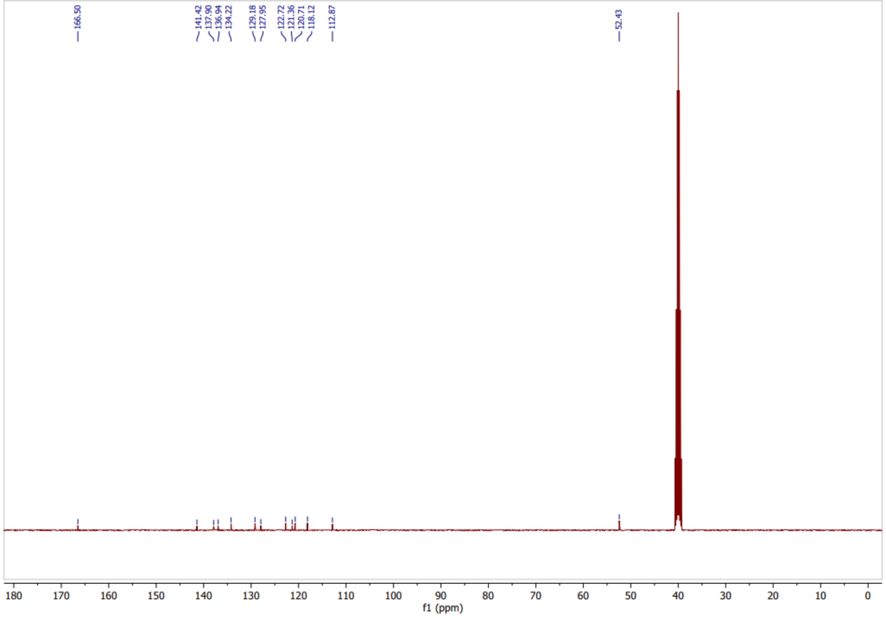

1'-methylspiro[indoline-3,3'-pyrrolidin]-2-one (44)

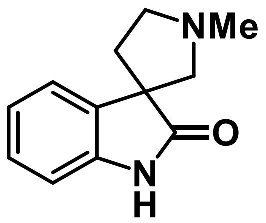

^{1}H-NMR (CDCl$_3$)

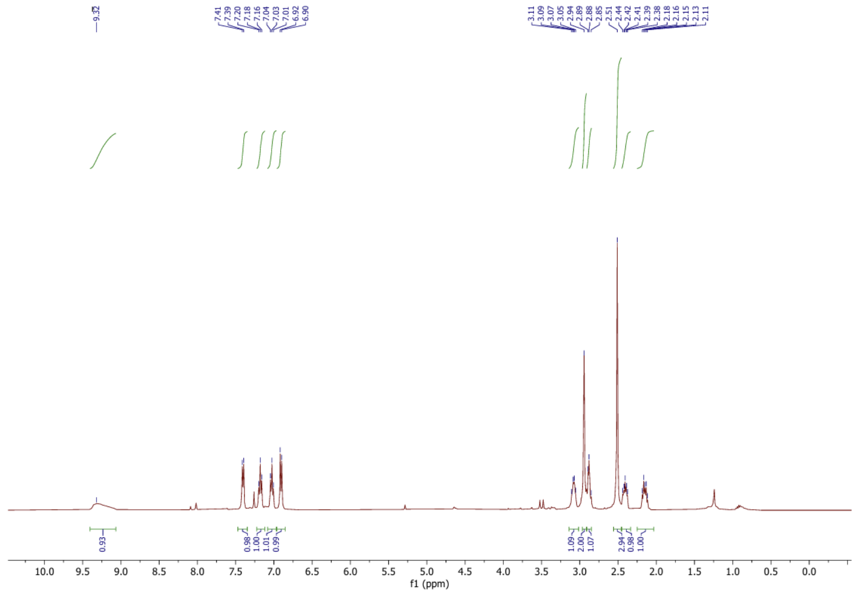

^{13}C-NMR (CDCl$_3$)

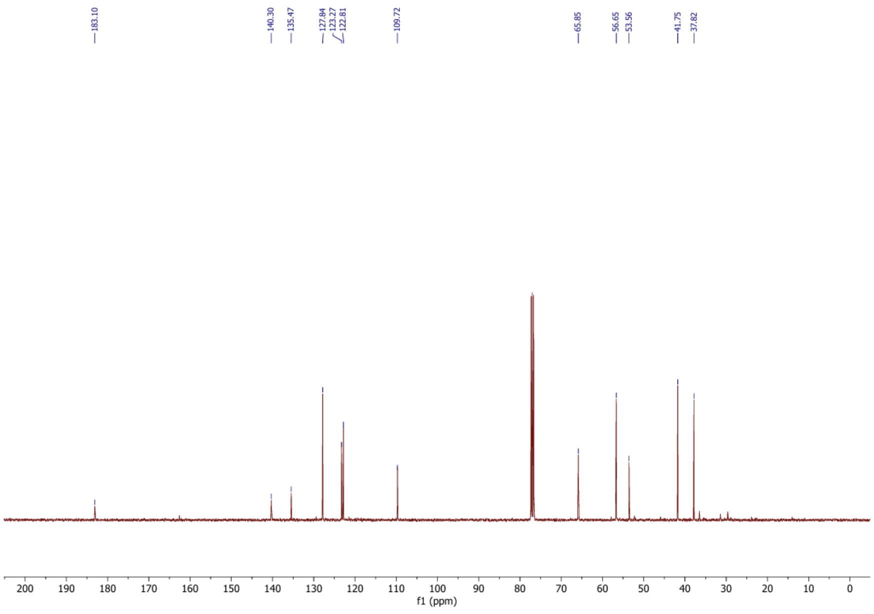

5-methoxy-1'-methylspiro[indoline-3,3'-pyrrolidin]-2-one (46)

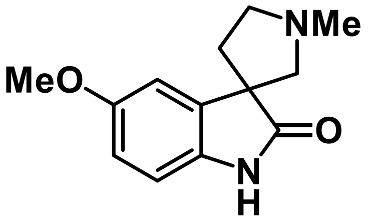

^{1}H-NMR (DMSO-d6)

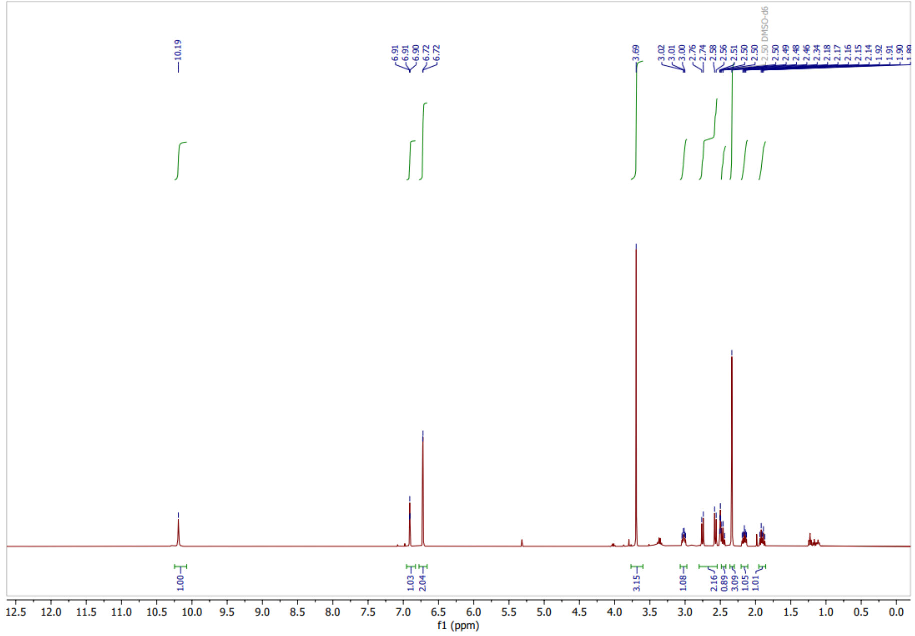

^{13}C-NMR (DMSO-d6)

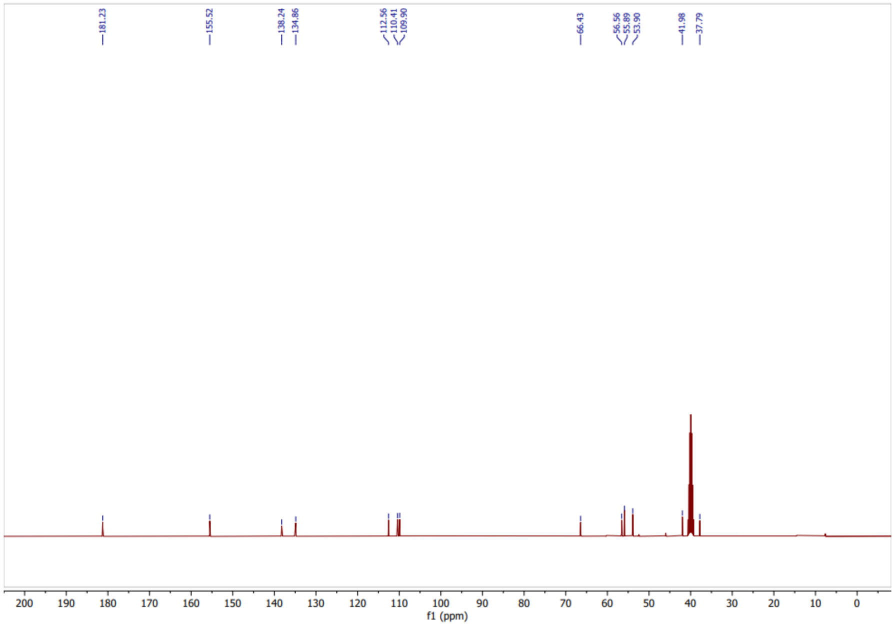

Appendix A: Crystal Structure Information

N-(2-(5-methoxy-2-oxoindolin-3-yl)ethyl)acetamide (20)

Crystal Data and Experimental

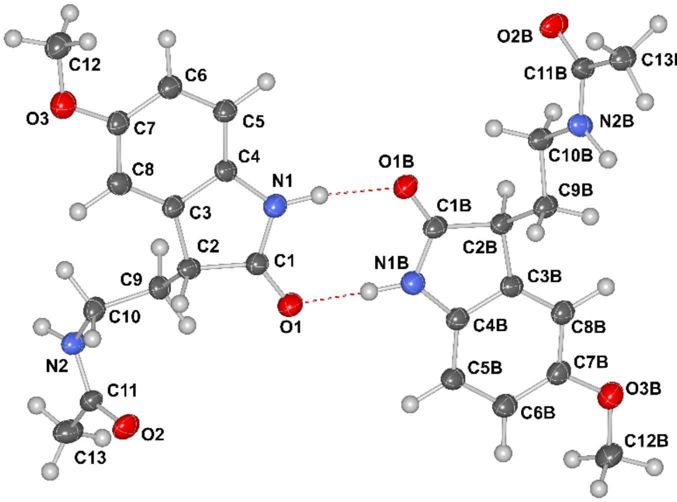

Experimental. Single colorless needle-shaped crystals of CB-123 recrystallized from a mixture of hexane and dimethyl carbonate by solvent layering. A suitable crystal with dimensions 0.18 × 0.06 × 0.03 mm^3 was selected and mounted on a loop with paratone on a XtaLAB Synergy-S diffractometer. The crystal was kept at a constant T = 100.0(1) K during data collection. The structure was solved with the ShelXT 2018/2 (Sheldrick, 2018) and Olex2 1.5-alpha (Dolomanov et al., 2009). The model was refined with olex2.refine 1.5-alpha (Bourhis et al., 2015) using full matrix least squares minimisation on $\boldsymbol{F^2}$.

Crystal Data. $C_{13}H_{16}N_2O_3$, M_r = 248.284, triclinic, P-1 (No. 2), a = 9.4287(9) Å, b = 9.7354(13) Å, c = 13.7668(18) Å, α = 82.277(11)°, β = 82.921(9)°, γ = 81.419(9)°, V = 1231.3(3) Å^3, T = 100.00(10) K, Z = 4, Z' = 2, μ(Cu K$_\alpha$) = 0.793, 16482 reflections measured, 4573 unique (R_{int} = 0.0663) which were used in all calculations. The final wR_2 was 0.2009 (all data) and R_1 was 0.0714 (I≥2 σ(I)).

**Compound**	CB-123
Formula	$C_{13}H_{16}N_2O_3$
$D_{calc.}$/ g cm^{-3}	1.339
μ/mm^{-1}	0.793
Formula Weight	248.284
Color	colorless
Shape	needle-shaped
Size/mm^3	0.18×0.06×0.03
T/K	100.00(10)
Crystal System	triclinic
Space Group	P-1
a/Å	9.4287(9)
b/Å	9.7354(13)
c/Å	13.7668(18)
α/°	82.277(11)
β/°	82.921(9)
γ/°	81.419(9)
V/Å^3	1231.3(3)
Z	4
Z'	2
Wavelength/Å	1.54184
Radiation type	Cu K$_\alpha$
Θ_{min}/°	3.26
Θ_{max}/°	73.95
Measured Refl's.	16482
Indep't Refl's	4573
Refl's I≥2 σ(I)	2772
R_{int}	0.0663
Parameters	381
Restraints	2
Largest Peak	1.0425
Deepest Hole	-0.4749
GooF	1.0415
wR_2 (all data)	0.2009
wR_2	0.1714
R_1 (all data)	0.1235
R_1	0.0714

Structure Quality Indicators

**Reflections:**	d min (CuKα) 2Θ=147.9° 0.80	I/σ(I) 14.7	Rint m=3.60 6.63%	Full 135.4° 91% to 147.9° 97.6
**Refinement:**	Shift -0.004	Max Peak 1.0	Min Peak -0.5	GooF 1.042

A colorless needle-shaped-shaped crystal with dimensions 0.18 × 0.06 × 0.03 mm^3 was mounted on a loop

with paratone. Data were collected using a XtaLAB Synergy, Dualflex, HyPix diffractometer operating at T = 100.00(10) K. Data were measured using ω scans with Cu K_α radiation. The diffraction pattern was indexed and the total number of runs and images was based on the strategy calculation from the program CrysAlisPro system (CCD 44.57a 64-bit (release 20-06-2024)). The maximum resolution that was achieved was Θ = 73.95° (0.83 Å). The unit cell was refined using CrysAlisPro 1.171.44.57a (Rigaku OD, 2024) on 2619 reflections, 16% of the observed reflections.

Data reduction, scaling and absorption corrections were performed using CrysAlisPro 1.171.44.57a (Rigaku OD, 2024). The final completeness is 97.58 % out to 73.95° in Θ. A numerical absorption correction based on gaussian integration over a multifaceted crystal model was performed using CrysAlisPro 1.171.42.74a (Rigaku Oxford Diffraction, 2022). An empirical absorption correction using spherical harmonics, implemented in SCALE3 ABSPACK scaling algorithm was also applied. The absorption coefficient μ of this material is 0.793 mm^{-1} at this wavelength (λ = 1.54184Å) and the minimum and maximum transmissions are 0.837 and 1.000.

The structure was solved, and the space group P-1 (# 2) determined by the ShelXT 2018/2 (Sheldrick, 2018) and refined by full matrix least squares minimisation on $\boldsymbol{F^2}$ using version of olex2.refine 1.5-alpha (Bourhis et al., 2015). All non-hydrogen atoms were refined anisotropically. Some hydrogen atom positions were calculated geometrically and refined using the riding model, but other hydrogen atoms were refined freely with distance restraints. Residual, secondary peaks appear near the main molecule and match the positions of the partner molecule overlaid on the first one but displaced by 0.2 Å from the main molecule. The positions of the peaks correspond to the same structure, indicating that the crystal is pure, but consists of domains like twin domains. This can be considered unresolved disorder that affects the entire molecule, specifically stacking or translational disorder along one of the crystal axes. It can also be described as a minor daughter domains within the crystal, with a population much smaller than the main component (approximately 10%, or about 1 $eÅ^{-3}$ for the oxygen atom). The repeating unit consists of two independent molecules with small differences in the conformations of the acetamide side chain, but minimal differences in the indole group. These two molecules hydrogen bond to each other, and interestingly, the best planes of the indole groups are displaced by a small amount (around 0.2 A) in the direction perpendicular to the planes. Refinement was by using NoSpherA2, an implementation of non-spherical atom-form-factors (F. Kleemiss, H. Puschmann, O. Dolomanov, S.Grabowsky - https://doi.org/10.1039/D0SC05526C – 2020). NoSpherA2 implementation of HAR makes use of tailor-made aspherical atomic form factors calculated from a Hirshfeld-partitioned electron density (ED) not from spherical-atom form factors. The ED was calculated from a Gaussian basis set single determinant SCF wavefunction from DFT using selected functionals for a fragment of this crystal. This fragment was embedded in an electrostatic crystal field by employing cluster charges. The following options were used. SOFTWARE: ORCA 5.0 PARTITIONING: NoSpherA2 INT ACCURACY: Normal METHOD: PBE BASIS SET: def2-SVP CHARGE: 0 MULTIPLICITY: 1 DATE: 2025-07-24_11-10-14

The value of Z' is 2. This means that there are two independent molecules in the asymmetric unit. The moiety formula is C13 H16 N2 O3.

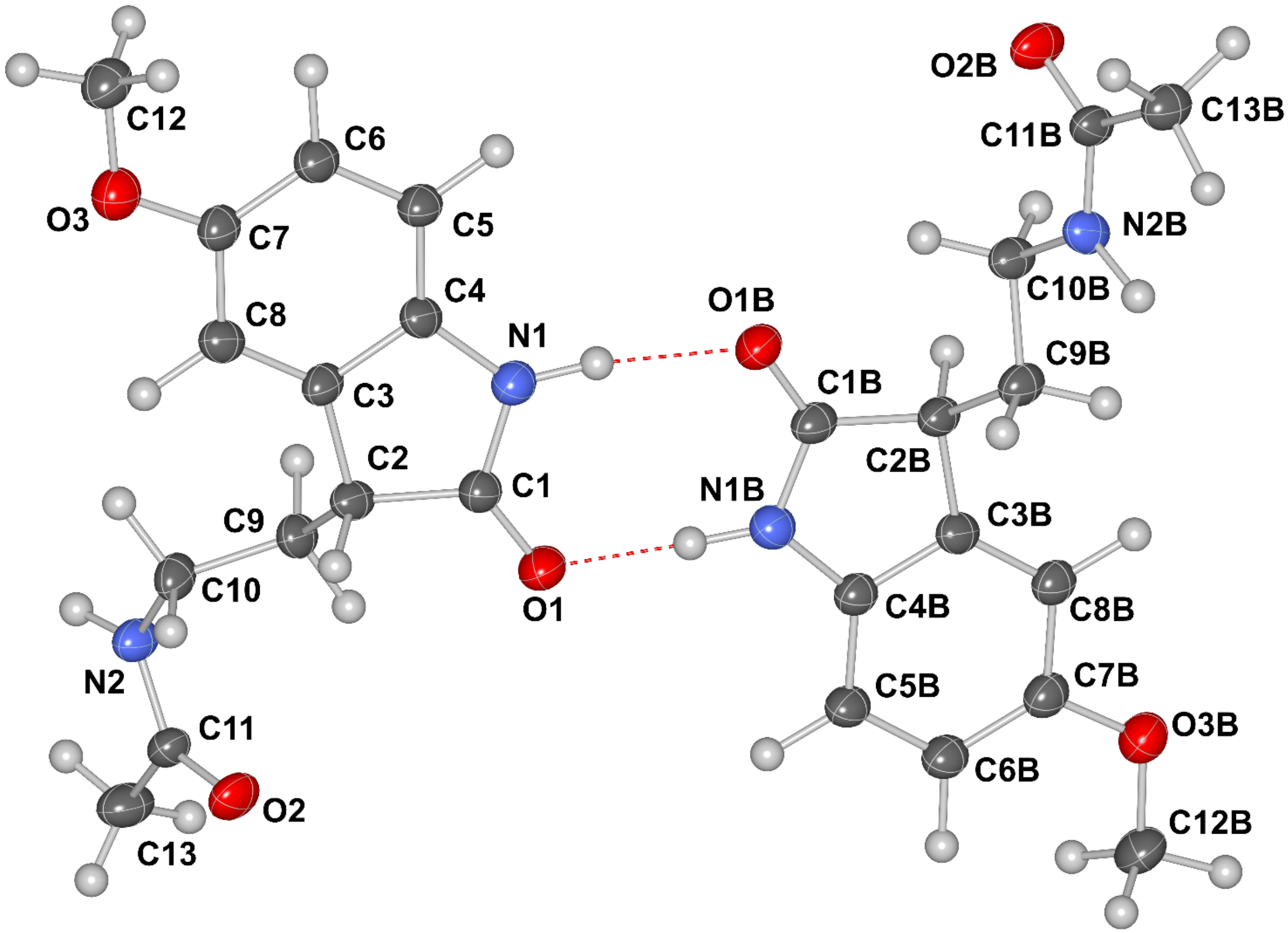

Figure 1 Thermal ellipsoidal representation of the asymmetric unit. The asymmetric unit consists of a hydrogen bonded pair of molecules with small differences in the conformations of the acetamide side chain, but minimal differences in the indole group. The two molecules are offset from each other along the stacking direction by a distance of 0.2 Å.

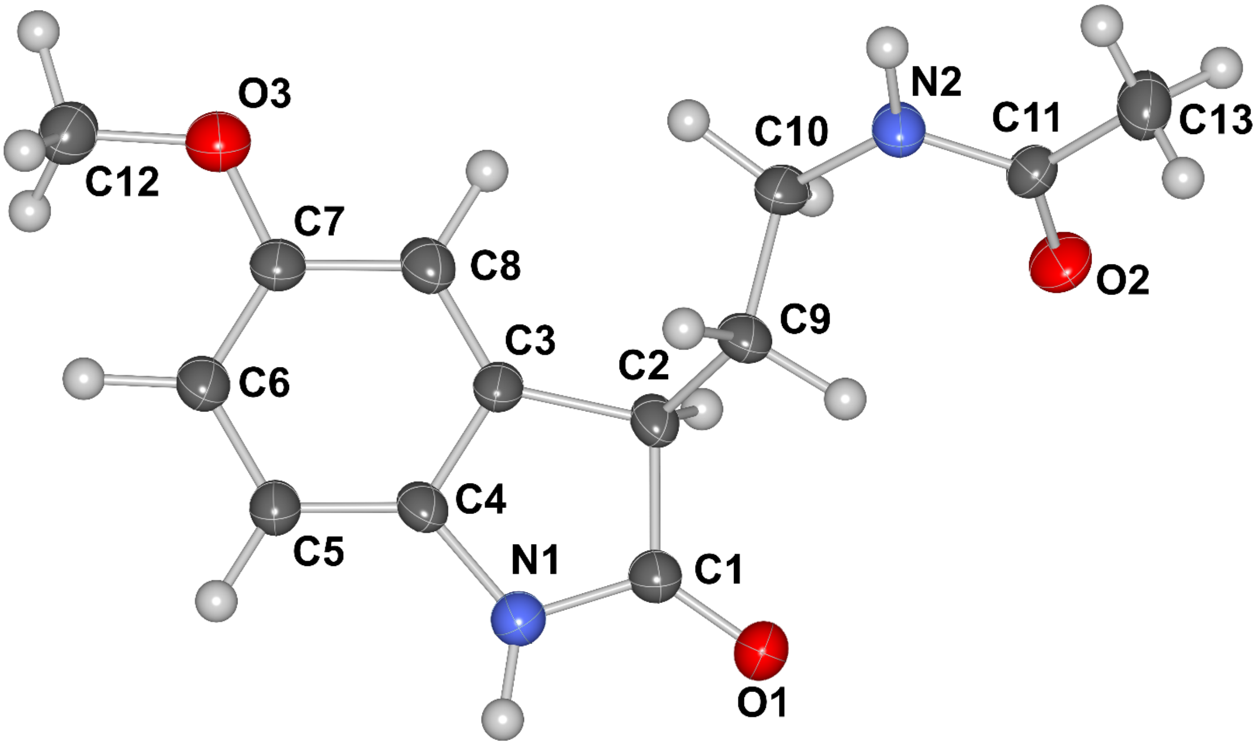

Figure 2 Thermal ellipsoidal representation of one of the two independent molecules in the asymmetric unit.

Data Plots: Diffraction Data

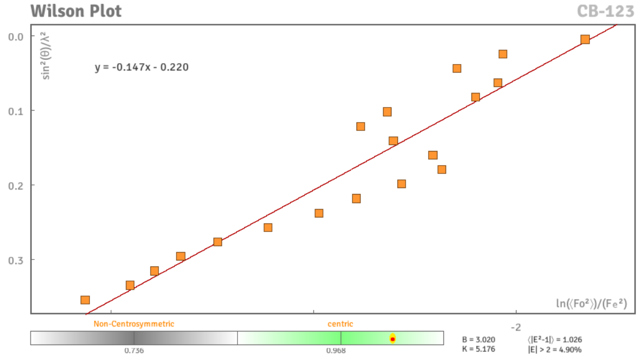

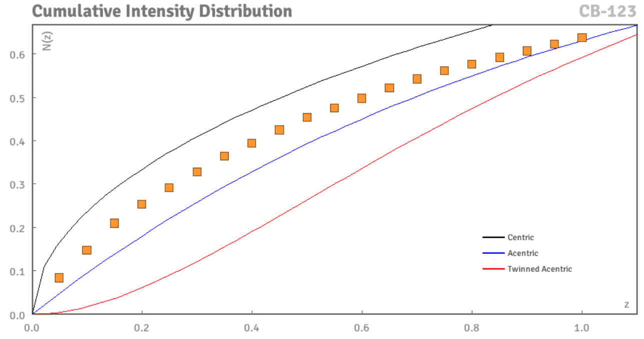

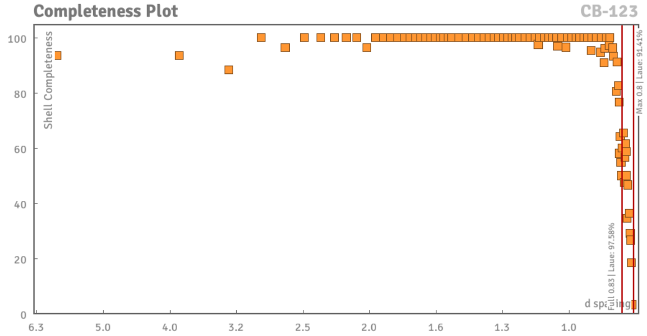

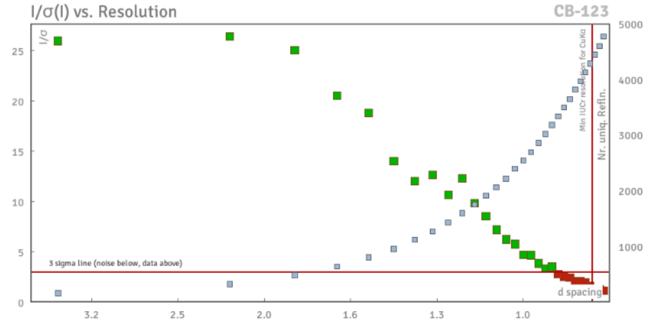

Data Plots: Refinement and Data

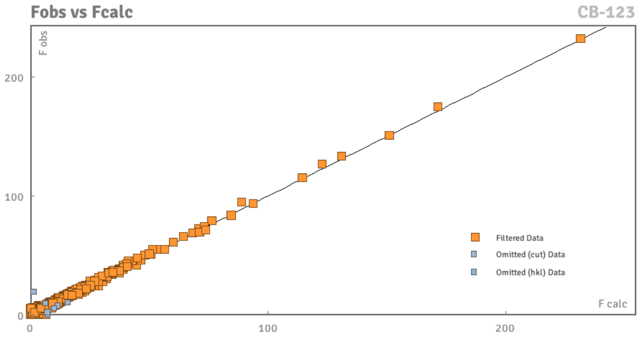

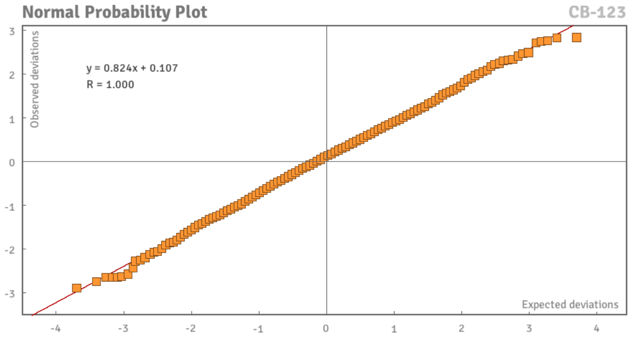

Reflection Statistics

Total reflections (after filtering)	16467	Unique reflections	4573
Completeness	0.914	Mean I/σ	9.08
hkl_{max} collected	(7, 12, 16)	hkl_{min} collected	(-11, -12, -16)
hkl_{max} used	(11, 12, 16)	hkl_{min} used	(-11, -11, 0)
Lim d_{max} collected	100.0	Lim d_{min} collected	0.77
d_{max} used	13.57	d_{min} used	0.8
Friedel pairs	1760	Friedel pairs merged	1
Inconsistent equivalents	2	R_{int}	0.0663
R_{sigma}	0.0678	Intensity transformed	0
Omitted reflections	0	Omitted by user (OMIT hkl)	15
Multiplicity	(2277, 1598, 1041, 570, 314, 218, 120, 86, 50, 27, 14, 15, 8, 3)	Maximum multiplicity	14
Removed systematic absences	0	Filtered off (Shel/OMIT)	0

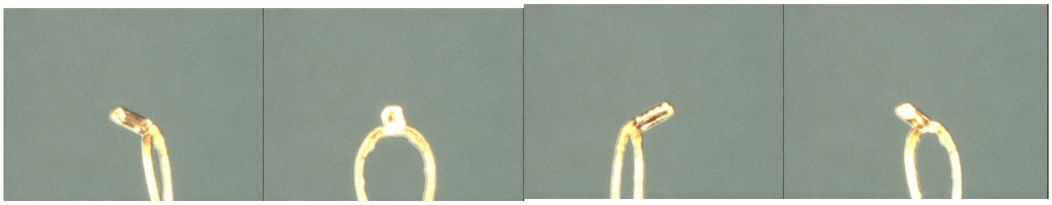

Table 1: Fractional Atomic Coordinates (×10^4^) and Equivalent Isotropic Displacement Parameters (Å^2×10^3) for CB-123. U_{eq} is defined as 1/3 of the trace of the orthogonalised U_{ij}.

Atom	x	y	z	U_{eq}
O1B	6923(2)	5553(3)	6458.8(18)	37.9(6)
O3B	2722(2)	-116(3)	7642.5(19)	42.1(7)
O2B	6012(2)	7663(3)	9686.6(19)	43.3(7)
N2B	4239(3)	6759(3)	9124(2)	32.9(7)
N1B	5978(3)	3902(3)	5787(2)	33.5(7)
C4B	5153(3)	2823(4)	6120(3)	32.3(8)
C11B	4706(3)	7667(4)	9620(3)	35.5(9)
C2B	5518(3)	3851(4)	7506(3)	32.9(8)
C7B	3509(3)	835(4)	7102(3)	36.8(9)
C3B	4843(3)	2732(4)	7144(3)	33.8(8)
C5B	4642(3)	1938(4)	5570(3)	36.1(9)
C8B	4016(3)	1747(4)	7647(3)	35.1(8)

Atom	x	y	z	U_{eq}
C10B	5263(3)	5772(4)	8608(3)	35.0(9)
C1B	6235(3)	4545(4)	6547(3)	34.1(8)
C6B	3803(3)	937(4)	6078(3)	36.8(9)
C9B	4455(3)	4871(4)	8104(3)	35.2(9)
C13B	3582(4)	8698(4)	10092(3)	39.9(9)
C12B	2209(4)	-1092(4)	7137(3)	46.8(10)
O1	7425(2)	4480(3)	3946.7(18)	38.1(6)
O2	8821(2)	3250(3)	801.4(19)	41.6(7)
O3	12133(2)	9669(3)	2456.8(19)	42.5(7)
N1	8617(3)	6027(3)	4562(2)	33.4(7)
N2	11092(3)	3600(3)	942(2)	33.7(7)
C1	8261(3)	5356(4)	3836(3)	34.3(8)
C2	9096(3)	5849(4)	2868(3)	33.0(8)
C3	9883(3)	6944(4)	3158(3)	31.7(8)
C4	9582(3)	6992(4)	4172(3)	32.6(8)
C5	10165(4)	7903(4)	4655(3)	35.5(9)
C6	11057(4)	8813(4)	4088(3)	36.5(9)
C7	11326(3)	8801(4)	3073(3)	34.9(8)
C8	10741(3)	7851(4)	2598(3)	36.1(9)
C9	10016(4)	4572(4)	2465(3)	35.2(9)
C10	10586(4)	4892(4)	1385(3)	35.8(9)
C11	10145(3)	2864(4)	679(3)	32.1(8)
C12	12711(4)	10702(4)	2859(3)	43.7(10)
C13	10757(4)	1548(4)	242(3)	47.6(11)

Table 2: Anisotropic Displacement Parameters (×10[4]) for CB-123. The anisotropic displacement factor exponent takes the form: $-2\pi^2[h^2a^{*2} \times U_{11} + ... + 2hka^* \times b^* \times U_{12}]$

Atom	U_{11}	U_{22}	U_{33}	U_{23}	U_{13}	U_{12}
O1B	33.8(12)	44.4(16)	38.9(15)	-14.9(12)	1.7(11)	-11.3(12)
O3B	38.5(13)	46.7(16)	43.2(16)	-14.2(12)	1.5(11)	-8.5(13)
O2B	22.4(12)	57.9(18)	53.8(17)	-6.7(11)	1.3(11)	-25.5(14)
N2B	23.6(14)	42.8(18)	33.7(17)	-0.2(12)	-1.0(12)	-16.1(14)
N1B	30.1(15)	38.5(18)	33.7(18)	-6.7(13)	-0.4(13)	-11.3(14)
C4B	26.9(16)	36(2)	36(2)	-5.1(15)	-0.7(14)	-9.9(17)
C11B	23.5(17)	50(2)	36(2)	-6.5(16)	-0.7(14)	-15.0(18)
C2B	26.0(16)	40(2)	36(2)	-8.6(15)	1.3(15)	-14.3(17)
C7B	28.0(17)	40(2)	43(2)	-7.2(15)	1.6(15)	-11.6(18)
C3B	25.5(16)	42(2)	36(2)	-4.2(15)	1.6(14)	-16.4(18)
C5B	32.3(17)	41(2)	38(2)	-8.6(16)	0.8(15)	-16.2(18)
C8B	26.9(16)	39(2)	40(2)	-4.9(15)	1.3(15)	-11.6(18)
C10B	26.1(16)	40(2)	42(2)	-3.2(15)	0.5(15)	-19.0(18)
C1B	27.5(16)	36(2)	41(2)	-6.1(15)	0.0(15)	-14.8(17)
C6B	32.2(18)	40(2)	41(2)	-7.1(16)	-0.1(16)	-14.2(18)
C9B	24.8(16)	39(2)	43(2)	-4.2(15)	0.6(15)	-15.2(18)
C13B	28.6(18)	44(2)	49(2)	-2.5(16)	-0.4(16)	-17.9(19)
C12B	45(2)	48(3)	52(3)	-21(2)	-2.9(19)	-8(2)
O1	37.0(13)	43.3(15)	37.9(15)	-16.1(12)	0.8(11)	-11.7(12)
O2	22.9(12)	56.4(18)	48.4(16)	-8.9(11)	-0.1(10)	-16.0(14)
O3	36.6(13)	49.2(17)	43.0(16)	-9.2(12)	2.9(11)	-12.4(13)
N1	30.0(14)	37.7(18)	34.5(17)	-9.0(13)	1.4(13)	-11.1(15)
N2	25.3(14)	40.4(18)	38.1(17)	-6.6(13)	1.8(12)	-16.5(14)
C1	30.1(17)	40(2)	34(2)	-7.6(16)	0.0(14)	-10.5(17)
C2	25.2(16)	40(2)	37(2)	-4.2(15)	0.5(14)	-18.4(17)
C3	25.8(16)	35(2)	37(2)	-6.6(14)	3.9(14)	-14.0(17)
C4	26.5(16)	41(2)	33(2)	-4.3(15)	1.9(14)	-15.8(17)

Atom	U_{11}	U_{22}	U_{33}	U_{23}	U_{13}	U_{12}
C5	32.6(18)	38(2)	38(2)	-9.1(16)	0.4(15)	-12.9(18)
C6	31.1(18)	41(2)	40(2)	-6.5(16)	1.8(15)	-15.2(18)
C7	28.7(17)	38(2)	38(2)	-5.3(15)	3.0(15)	-11.3(17)
C8	28.9(17)	44(2)	37(2)	-2.9(16)	-1.3(15)	-14.6(18)
C9	29.4(17)	43(2)	34(2)	-5.8(16)	4.4(15)	-15.1(17)
C10	31.4(18)	37(2)	40(2)	-7.6(16)	7.7(16)	-14.0(17)
C11	25.2(17)	38(2)	34.9(19)	-8.6(15)	-3.0(14)	-7.3(16)
C12	40(2)	41(2)	52(3)	-9.5(18)	0.3(19)	-11(2)
C13	39(2)	53(3)	58(3)	-10.9(19)	-5.6(19)	-23(2)

Table 3: Bond Lengths in Å for CB-123.

Atom	Atom	Length/Å
O1B	C1B	1.240(4)
O3B	C7B	1.359(4)
O3B	C12B	1.421(4)
O2B	C11B	1.246(4)
N2B	C11B	1.339(4)
N2B	C10B	1.448(4)
N2B	H2B	1.08(4)
N1B	H1B	0.990(18)
N1B	C4B	1.395(4)
N1B	C1B	1.353(5)
C4B	C3B	1.398(5)
C4B	C5B	1.392(5)
C11B	C13B	1.498(5)
C2B	H2Ba	1.12(4)
C2B	C3B	1.506(5)
C2B	C1B	1.527(5)
C2B	C9B	1.546(5)
C7B	C8B	1.405(5)
C7B	C6B	1.396(5)
C3B	C8B	1.385(5)
C5B	H5B	1.1030
C5B	C6B	1.405(5)
C8B	H8B	1.1030
C10B	H10a	1.09(3)
C10B	H10b	1.09(3)
C10B	C9B	1.524(5)
C6B	H6B	1.1030
C9B	H9Ba	1.05(3)
C9B	H9Bb	1.05(3)
C13B	H13a	1.11(3)
C13B	H13b	1.11(3)
C13B	H13c	1.11(3)
C12B	H12a	1.13(2)
C12B	H12b	1.13(2)
C12B	H12c	1.13(2)

Atom	Atom	Length/Å
O1	C1	1.228(4)
O2	C11	1.246(4)
O3	C7	1.362(4)
O3	C12	1.415(4)
N1	H1	1.013(18)
N1	C1	1.364(5)
N1	C4	1.414(4)
N2	C10	1.462(4)
N2	C11	1.335(4)
N2	H2	1.05(5)
C1	C2	1.519(5)
C2	H2a	1.1180
C2	C3	1.506(5)
C2	C9	1.534(5)
C3	C4	1.395(5)
C3	C8	1.381(5)
C4	C5	1.387(5)
C5	H5	1.11(5)
C5	C6	1.406(5)
C6	H6	1.12(4)
C6	C7	1.390(5)
C7	C8	1.410(5)
C8	H8	1.1030
C9	H9a	1.13(2)
C9	H9b	1.13(2)
C9	C10	1.524(5)
C10	H10c	1.10(3)
C10	H10d	1.10(3)
C11	C13	1.496(5)
C12	H12d	1.05(2)
C12	H12e	1.05(2)
C12	H12f	1.05(2)
C13	H13d	1.00(3)
C13	H13e	1.00(3)
C13	H13f	1.00(3)

Table 4: Bond Angles in ° for CB-123.

Atom	Atom	Atom	Angle/°
C12B	O3B	C7B	117.9(3)

Atom	Atom	Atom	Angle/°
C10B	N2B	C11B	120.2(3)

Atom	Atom	Atom	Angle/°
H2B	N2B	C11B	118(2)
H2B	N2B	C10B	122(2)
C4B	N1B	H1B	128(2)
C1B	N1B	H1B	121(2)
C1B	N1B	C4B	110.9(3)
C3B	C4B	N1B	110.0(3)
C5B	C4B	N1B	128.4(3)
C5B	C4B	C3B	121.6(3)
N2B	C11B	O2B	122.0(3)
C13B	C11B	O2B	121.1(3)
C13B	C11B	N2B	116.9(3)
C3B	C2B	H2Ba	108.6(2)
C1B	C2B	H2Ba	108.58(18)
C1B	C2B	C3B	101.9(3)
C9B	C2B	H2Ba	108.58(19)
C9B	C2B	C3B	114.8(3)
C9B	C2B	C1B	114.1(3)
C8B	C7B	O3B	115.2(3)
C6B	C7B	O3B	123.8(3)
C6B	C7B	C8B	121.0(3)
C2B	C3B	C4B	108.2(3)
C8B	C3B	C4B	120.8(3)
C8B	C3B	C2B	131.0(3)
H5B	C5B	C4B	121.1(2)
C6B	C5B	C4B	117.8(3)
C6B	C5B	H5B	121.1(2)
C3B	C8B	C7B	118.2(3)
H8B	C8B	C7B	120.9(2)
H8B	C8B	C3B	120.9(2)
H10a	C10B	N2B	109.75(19)
H10b	C10B	N2B	109.75(19)
H10b	C10B	H10a	108.22084693(2)
C9B	C10B	N2B	109.6(3)
C9B	C10B	H10a	109.8(2)
C9B	C10B	H10b	109.8(2)
N1B	C1B	O1B	124.4(3)
C2B	C1B	O1B	126.5(3)
C2B	C1B	N1B	109.1(3)
C5B	C6B	C7B	120.6(3)
H6B	C6B	C7B	119.7(2)
H6B	C6B	C5B	119.7(2)
C10B	C9B	C2B	111.0(3)
H9Ba	C9B	C2B	109.4(2)
H9Ba	C9B	C10B	109.4(2)
H9Bb	C9B	C2B	109.45(19)
H9Bb	C9B	C10B	109.4(2)
H9Bb	C9B	H9Ba	108.0
H13a	C13B	C11B	109.5
H13b	C13B	C11B	109.5
H13b	C13B	H13a	109.5
H13c	C13B	C11B	109.5
H13c	C13B	H13a	109.5
H13c	C13B	H13b	109.5
H12a	C12B	O3B	109.5
H12b	C12B	O3B	109.5
H12b	C12B	H12a	109.5
H12c	C12B	O3B	109.5
H12c	C12B	H12a	109.5

Atom	Atom	Atom	Angle/°
H12c	C12B	H12b	109.5
C12	O3	C7	118.6(3)
C1	N1	H1	117(3)
C4	N1	H1	132(3)
C4	N1	C1	110.8(3)
C11	N2	C10	120.1(3)
H2	N2	C10	124(3)
H2	N2	C11	115(3)
N1	C1	O1	125.8(3)
C2	C1	O1	125.5(3)
C2	C1	N1	108.7(3)
H2a	C2	C1	109.47(18)
C3	C2	C1	102.5(3)
C3	C2	H2a	109.5(2)
C9	C2	C1	108.3(3)
C9	C2	H2a	109.47(19)
C9	C2	C3	117.3(3)
C4	C3	C2	108.7(3)
C8	C3	C2	131.1(3)
C8	C3	C4	120.2(3)
C3	C4	N1	109.2(3)
C5	C4	N1	128.9(3)
C5	C4	C3	121.9(3)
H5	C5	C4	121(2)
C6	C5	C4	118.0(3)
C6	C5	H5	121(2)
H6	C6	C5	121.1(18)
C7	C6	C5	120.5(3)
C7	C6	H6	118.5(18)
C6	C7	O3	124.9(3)
C8	C7	O3	114.4(3)
C8	C7	C6	120.7(3)
C7	C8	C3	118.7(3)
H8	C8	C3	120.6(2)
H8	C8	C7	120.6(2)
H9a	C9	C2	109.22(19)
H9b	C9	C2	109.22(17)
H9b	C9	H9a	107.90780685(2)
C10	C9	C2	112.0(3)
C10	C9	H9a	109.22(19)
C10	C9	H9b	109.22(19)
C9	C10	N2	110.8(3)
H10c	C10	N2	109.48(17)
H10c	C10	C9	109.48(19)
H10d	C10	N2	109.48(19)
H10d	C10	C9	109.48(19)
H10d	C10	H10c	108.1
N2	C11	O2	121.5(3)
C13	C11	O2	122.0(3)
C13	C11	N2	116.5(3)
H12d	C12	O3	109.5
H12e	C12	O3	109.5
H12e	C12	H12d	109.5
H12f	C12	O3	109.5
H12f	C12	H12d	109.5
H12f	C12	H12e	109.5
H13d	C13	C11	109.5
H13e	C13	C11	109.5

Atom	Atom	Atom	Angle/˚
H13e	C13	H13d	109.5
H13f	C13	C11	109.5

Atom	Atom	Atom	Angle/˚
H13f	C13	H13d	109.5
H13f	C13	H13e	109.5

Table 5: Torsion Angles in ˚ for CB-123.

Atom	Atom	Atom	Atom	Angle/˚
O1B	C1B	N1B	C4B	-178.9(3)
O1B	C1B	C2B	C3B	179.0(4)
O1B	C1B	C2B	C9B	54.7(4)
O3B	C7B	C8B	C3B	179.2(3)
O3B	C7B	C6B	C5B	-179.2(3)
O2B	C11B	N2B	C10B	-2.7(5)
N2B	C10B	C9B	C2B	177.3(3)
N1B	C4B	C3B	C2B	0.2(3)
N1B	C4B	C3B	C8B	179.1(3)
N1B	C4B	C5B	C6B	-178.9(4)
N1B	C1B	C2B	C3B	0.3(3)
N1B	C1B	C2B	C9B	-124.0(3)
C4B	N1B	C1B	C2B	-0.1(3)
C4B	C3B	C2B	C1B	-0.3(3)
C4B	C3B	C2B	C9B	123.5(3)
C4B	C3B	C8B	C7B	0.5(4)
C4B	C5B	C6B	C7B	-0.5(4)
C11B	N2B	C10B	C9B	-179.7(4)
C2B	C3B	C4B	C5B	-178.6(3)
C2B	C3B	C8B	C7B	179.1(4)
C3B	C4B	N1B	C1B	-0.1(3)
C3B	C4B	C5B	C6B	-0.3(4)
C3B	C2B	C9B	C10B	168.7(3)
C3B	C8B	C7B	C6B	-1.3(4)
C5B	C4B	N1B	C1B	178.6(4)
C5B	C4B	C3B	C8B	0.3(4)
C5B	C6B	C7B	C8B	1.4(4)
C8B	C7B	O3B	C12B	-178.4(3)
C8B	C3B	C2B	C1B	-179.0(4)
C8B	C3B	C2B	C9B	-55.2(4)
C10B	N2B	C11B	C13B	177.2(3)
C10B	C9B	C2B	C1B	-74.3(3)
C6B	C7B	O3B	C12B	2.1(4)
O1	C1	N1	C4	-178.5(4)
O1	C1	C2	C3	177.8(4)
O1	C1	C2	C9	-57.6(4)
O2	C11	N2	C10	-0.0(4)
O3	C7	C6	C5	177.7(3)
O3	C7	C8	C3	-178.7(3)
N1	C1	C2	C3	-3.4(3)
N1	C1	C2	C9	121.2(3)
N1	C4	C3	C2	-1.6(3)
N1	C4	C3	C8	176.4(3)
N1	C4	C5	C6	-177.1(4)
N2	C10	C9	C2	-163.6(3)
C1	N1	C4	C3	-0.7(3)
C1	N1	C4	C5	178.0(3)
C1	C2	C3	C4	2.9(3)
C1	C2	C3	C8	-174.8(3)
C1	C2	C9	C10	165.3(3)

Atom	Atom	Atom	Atom	Angle/°
C2	C1	N1	C4	2.6(3)
C2	C3	C4	C5	179.7(3)
C2	C3	C8	C7	178.7(4)
C3	C2	C9	C10	-79.5(3)
C3	C4	C5	C6	1.4(4)
C3	C8	C7	C6	0.7(4)
C4	C3	C2	C9	-115.5(3)
C4	C3	C8	C7	1.2(4)
C4	C5	C6	C7	0.6(4)
C5	C4	C3	C8	-2.3(4)
C5	C6	C7	C8	-1.7(4)
C6	C7	O3	C12	-2.3(4)
C8	C3	C2	C9	66.8(4)
C8	C7	O3	C12	177.2(3)
C9	C10	N2	C11	77.4(3)
C10	N2	C11	C13	-179.3(3)

Table 6: Hydrogen Fractional Atomic Coordinates (×10[4]) and Equivalent Isotropic Displacement Parameters (Å^2×10^3) for CB-123. U_{eq} is defined as 1/3 of the trace of the orthogonalised U_{ij}.

Atom	x	y	z	U_{eq}
H1B	6470(40)	4130(40)	5117(17)	27(10)
H2Ba	6380(30)	3332(17)	7978(16)	38(10)
H5B	4888(3)	2019(4)	4761(3)	50(11)
H8B	3763(3)	1680(4)	8455(3)	52.7(13)
H10a	5969(18)	6333(14)	8061(13)	53(11)
H10b	5925(17)	5109(16)	9128(13)	41(10)
H6B	3373(3)	225(4)	5664(3)	51(11)
H9Ba	3778(19)	5517(17)	7632(13)	43(10)
H9Bb	3810(18)	4301(15)	8637(14)	66(13)
H13a	3655(4)	8570(5)	10900(19)	120(20)
H13b	3762(5)	9780(30)	9776(8)	55(12)
H13c	2490(30)	8512(5)	9952(4)	80(15)
H12a	1444(18)	-515(13)	6603(12)	53(11)
H12b	3150(20)	-1697(14)	6719(10)	89(16)
H12c	1626(14)	-1840(17)	7687(12)	75(14)
H1	8180(40)	5760(50)	5259(18)	50(13)
H2a	8323(3)	6351(4)	2330(3)	62(13)
H5	9990(40)	7870(50)	5470(30)	65(13)
H6	11550(40)	9580(40)	4440(30)	34(9)
H8	10966(3)	7834(4)	1794(3)	54.1(13)
H9a	10960(20)	4218(9)	2917(11)	37(9)
H9b	9344(15)	3690(20)	2543(3)	48(11)
H10c	11480(20)	5514(16)	1330(3)	51(11)
H10d	9730(20)	5510(16)	982(10)	53(12)
H12d	13312(14)	11280(13)	2293(12)	69(14)
H12e	11862(18)	11381(15)	3187(8)	62(12)
H12f	13389(15)	10216(11)	3397(12)	60(13)
H13d	10510(7)	730(20)	710(12)	100(20)
H13e	10345(11)	1542(5)	-390(16)	110(20)
H13f	11830(30)	1501(5)	114(5)	55(12)
H2B	3090(50)	6800(40)	9120(30)	51(7)
H2	12180(50)	3220(50)	760(40)	76(11)

Table 7: Hydrogen Bond information for CB-123.

D	H	A	d(D-H)/Å	d(H-A)/Å	d(D-A)/Å	D-H-A/deg
N1B	H1B	O1	0.990(18)	1.766(19)	2.755(4)	177(3)
N2B	H2B	O2[1]	1.08(4)	1.80(4)	2.876(3)	175(4)

––––

[1]1-x,1-y,1-z

Citations

CrysAlisPro (ROD), Rigaku Oxford Diffraction, Poland (?).

CrysAlisPro Software System, Rigaku Oxford Diffraction, (2024).

L.J. Bourhis and O.V. Dolomanov and R.J. Gildea and J.A.K. Howard and H. Puschmann, The Anatomy of a Comprehensive Constrained, Restrained, Refinement Program for the Modern Computing Environment - Olex2 Disected, *Acta Cryst. A*, (2015), **A71**, 59-71.

O.V. Dolomanov and L.J. Bourhis and R.J. Gildea and J.A.K. Howard and H. Puschmann, Olex2: A complete structure solution, refinement and analysis program, *J. Appl. Cryst.*, (2009), **42**, 339-341.

Sheldrick, G.M., ShelXT-Integrated space-group and crystal-structure determination, *Acta Cryst.*, (2015), **A71**, 3-8.

1'-methylspiro[indoline-3,3'-pyrrolidin]-2-one (44)

Crystal Data and Experimental

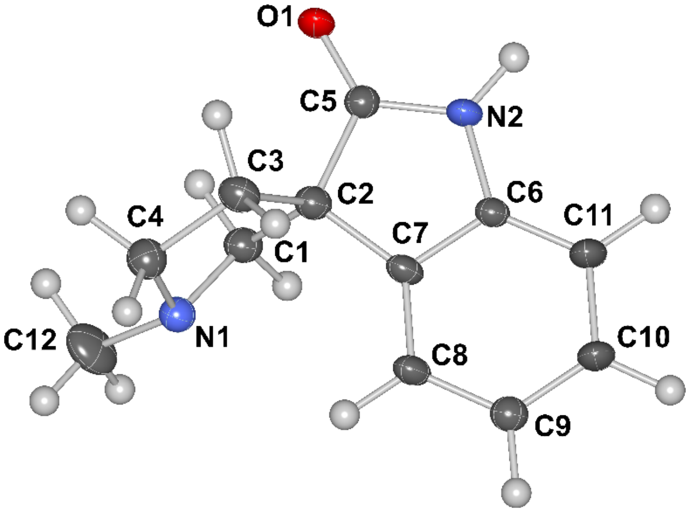

Experimental. Single colorless needle-shaped crystals of HJL-05-006-P were recrystallized from benzene by the oil drop method. A suitable crystal with dimensions 0.20 × 0.08 × 0.05 mm^3 was selected and mounted on a loop with paratone on a XtaLAB Synergy, Dualflex, HyPix diffractometer. The crystal was kept at a constant T = 102(3) K during data collection. The structure was solved with ShelXT (Sheldrick, 2015) and Olex2 1.5-alpha (Dolomanov et al., 2009). The model was refined with ShelXL 2018/3 (Sheldrick, 2015) using full matrix least squares minimisation on $\boldsymbol{F}^2$.

Crystal Data. $C_{12}H_{14}N_2O$, M_r = 202.25, triclinic, P-1 (No. 2), a = 5.7712(15) Å, b = 10.276(3) Å, c = 18.534(3) Å, α = 90.000(18)°, β = 93.696(16)°, γ = 104.78(2)°, V = 1060.4(5) Å^3, T = 102(3) K, Z = 4, Z' = 2, μ(Cu K$_\alpha$) = 0.656, 11008 reflections measured, 3216 unique (R_{int} = 0.1146) which were used in all calculations. The final wR_2 was 0.5491 (all data) and R_1 was 0.2107 (I≥2 σ(I)).

**Compound**	HJL-05-006-P
Formula	$C_{12}H_{14}N_2O$
$D_{calc.}$/ g cm^{-3}	1.267
μ/mm^{-1}	0.656
Formula Weight	202.25
Color	colorless
Shape	needle-shaped
Size/mm^3	0.20×0.08×0.05
T/K	102(3)
Crystal System	triclinic
Space Group	P-1
a/Å	5.7712(15)
b/Å	10.276(3)
c/Å	18.534(3)
α/°	90.000(18)
β/°	93.696(16)
γ/°	104.78(2)
V/Å^3	1060.4(5)
Z	4
Z'	2
Wavelength/Å	1.54184
Radiation type	Cu K$_\alpha$
Θ_{min}/°	4.451
Θ_{max}/°	65.065
Measured Refl's.	11008
Indep't Refl's	3216
Refl's I≥2 σ(I)	1019
R_{int}	0.1146
Parameters	455
Restraints	1234
Largest Peak	1.480
Deepest Hole	-0.547
GooF	1.523
wR_2 (all data)	0.5491
wR_2	0.4620
R_1 (all data)	0.3694
R_1	0.2107

Structure Quality Indicators

**Reflections:**	d min (CuKα) 2Θ=130.1° 0.85	I/σ(I) 6.6	Rint m=3.42 11.46%	Full 130.1° 89.0
**Refinement:**	Shift 0.003	Max Peak 1.5	Min Peak -0.6	GooF 1.523

A colorless needle-shaped-shaped crystal with dimensions 0.20 × 0.08 × 0.05 mm^3 was mounted on a loop with paratone. Data were collected using a XtaLAB Synergy, Dualflex, HyPix diffractometer equipped with an Oxford Cryosystems low-temperature device operating at T = 102(3) K.

Data were measured using ω and ϕ scans with Cu K_α radiation. The diffraction pattern was indexed and the total number of runs and images was based on the strategy calculation from the program CrysAlisPro system (CCD 44.57a 64-bit (release 20-06-2024)). The maximum resolution that was achieved was Θ = 65.065° (0.85 Å).

The unit cell was refined using CrysAlisPro 1.171.44.57a (Rigaku OD, 2024) on 841 reflections, 8% of the observed reflections.

Data reduction, scaling and absorption corrections were performed using CrysAlisPro 1.171.44.57a (Rigaku OD, 2024). The final completeness is 88.90 % out to 65.065° in Θ. A numerical absorption correction based on gaussian integration over a multifaceted crystal model was performed using CrysAlisPro 1.171.42.74a (Rigaku Oxford Diffraction, 2022). An empirical absorption correction using spherical harmonics, implemented in SCALE3 ABSPACK scaling algorithm was also applied.The absorption coefficient μ of this material is 0.656 mm^{-1} at this wavelength (λ = 1.54184Å) and the minimum and maximum transmissions are 0.789 and 1.000.

The structure was solved and the space group P-1 (# 2) determined by the ShelXT (Sheldrick, 2015) structure solution program and refined by full matrix least squares minimisation on $\boldsymbol{F^2}$ using version 2018/3 of ShelXL 2018/3 (Sheldrick, 2015). All non-hydrogen atoms were refined anisotropically. Hydrogen atom positions were calculated geometrically and refined using the riding model. Hydrogen atom positions were calculated geometrically and refined using the riding model.

The value of Z' is 2. This means that there are two independent molecules in the asymmetric unit. The moiety formula is C12 H14 N2 O.

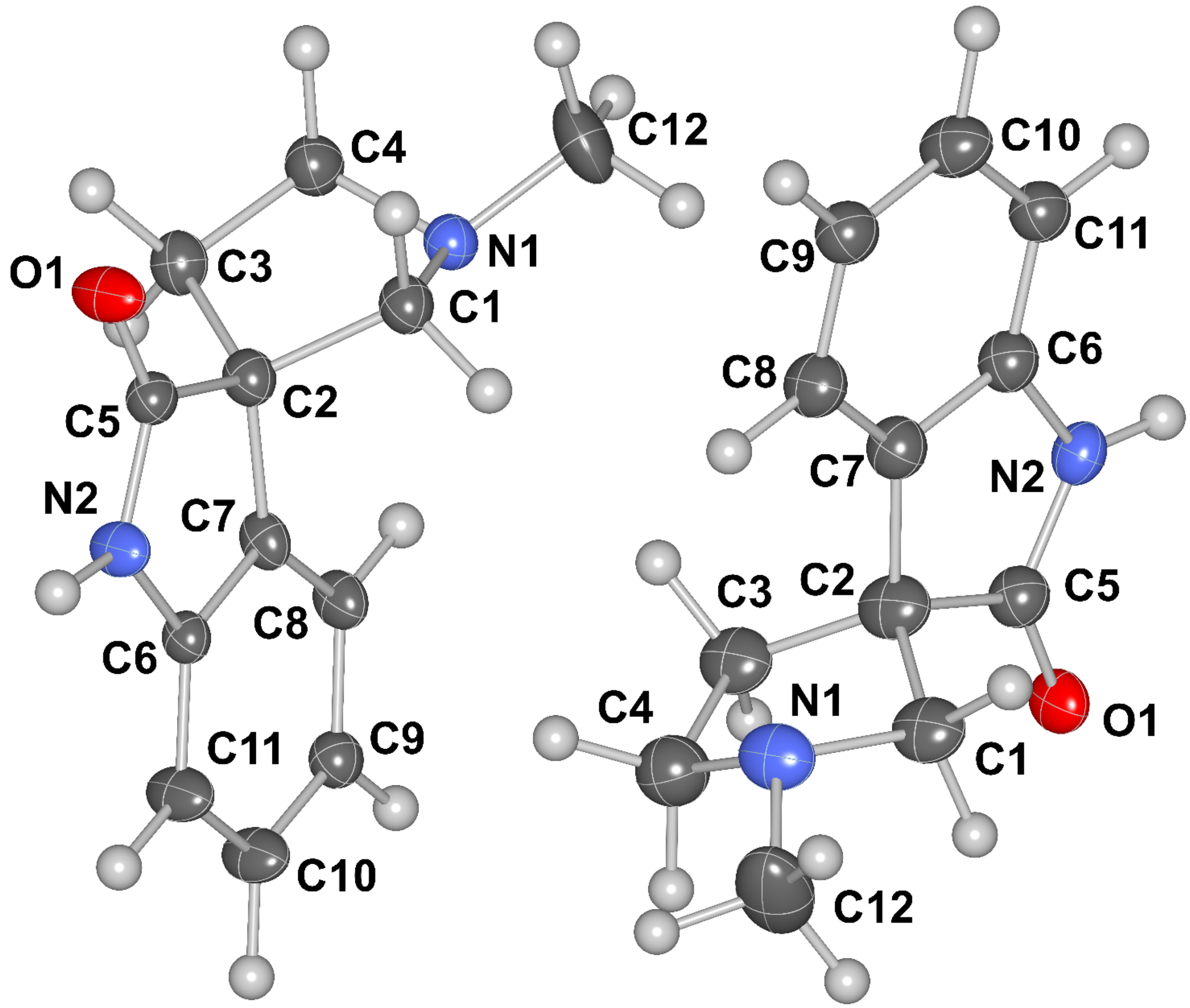

Figure 3 Thermal ellipsoidal representation of the asymmetric unit. The asymmetric unit consists of two disordered molecule (one disordered R-molecule and one disordered R/S-molecule). Only 1 component (one isomer is shown here).

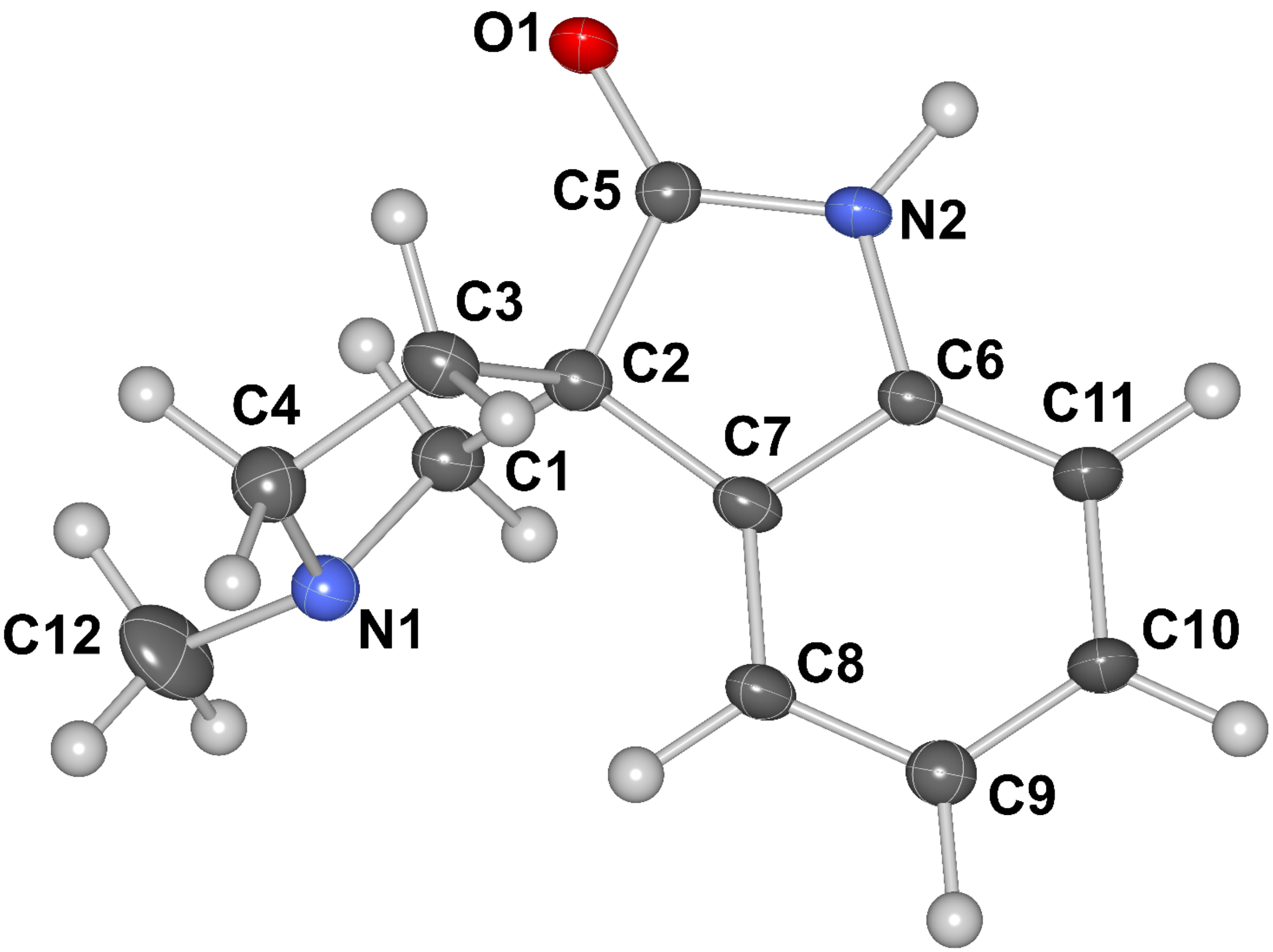

Figure 4

Data Plots: Diffraction Data

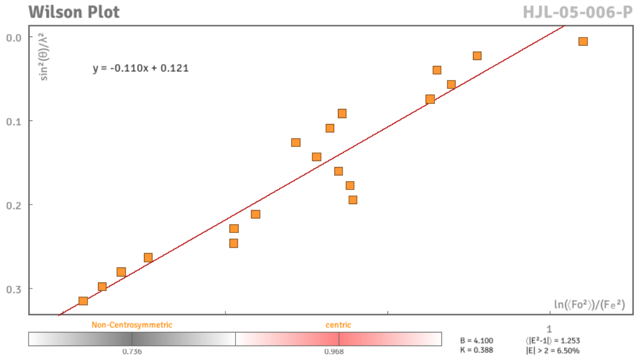

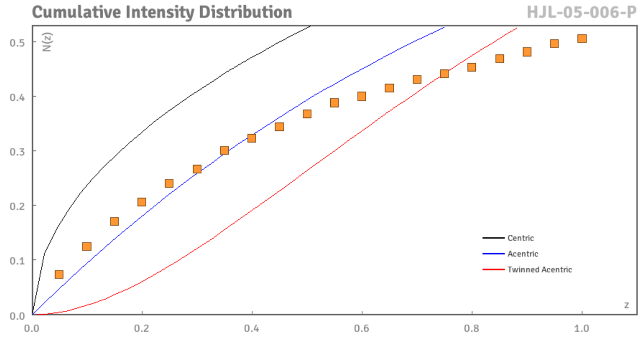

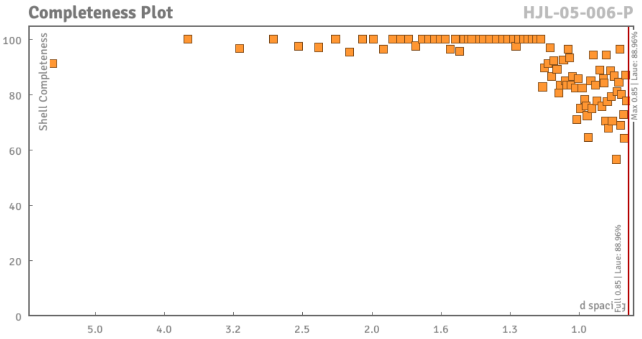

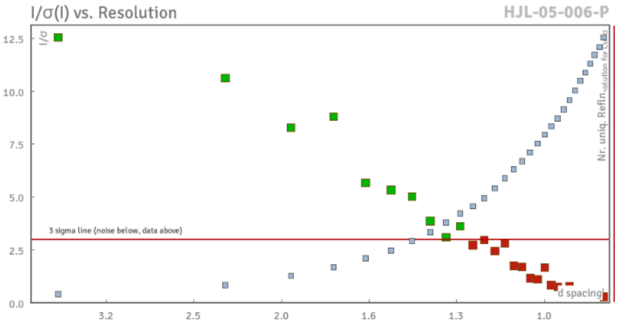

Data Plots: Refinement and Data

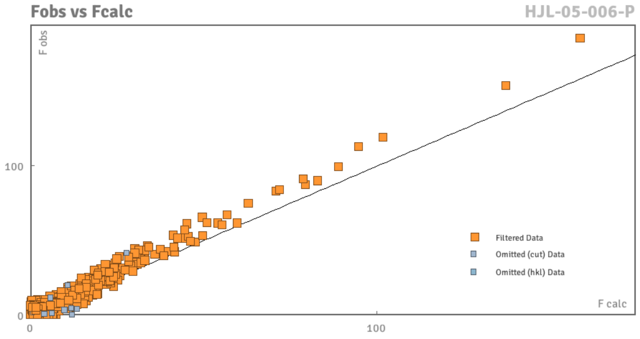

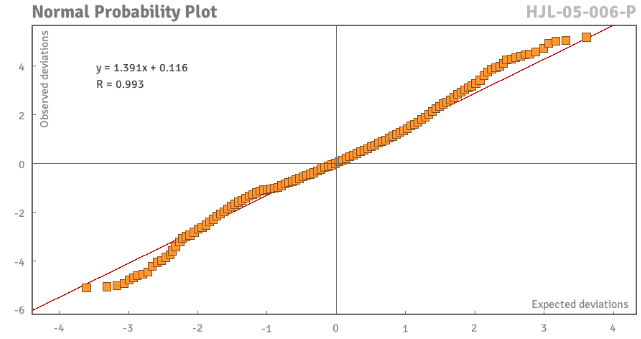

Reflection Statistics

Total reflections (after filtering)	11008	Unique reflections	3216
Completeness	0.89	Mean I/σ	3.28
hkl_{max} collected	(6, 12, 21)	hkl_{min} collected	(-6, -12, -15)
hkl_{max} used	(6, 12, 21)	hkl_{min} used	(-6, -12, 0)
Lim d_{max} collected	100.0	Lim d_{min} collected	0.77
d_{max} used	9.93	d_{min} used	0.85
Friedel pairs	1324	Friedel pairs merged	1
Inconsistent equivalents	0	R_{int}	0.1146
R_{sigma}	0.1515	Intensity transformed	0
Omitted reflections	0	Omitted by user (OMIT hkl)	48
Multiplicity	(1698, 1205, 707, 452, 231, 113, 52, 43, 25, 13, 10, 0, 1)	Maximum multiplicity	13
Removed systematic absences	0	Filtered off (Shel/OMIT)	0

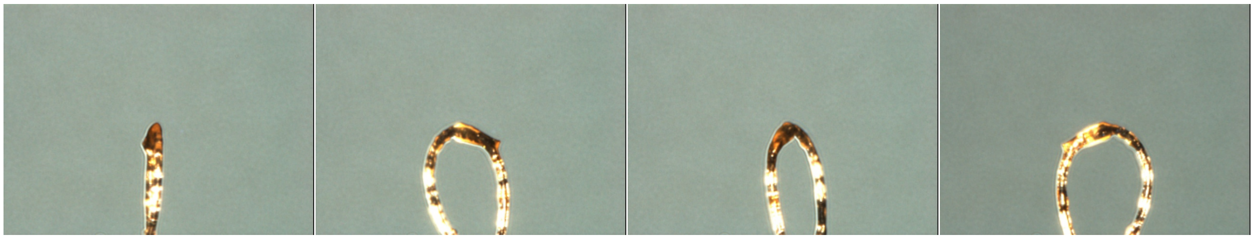

Table 8: Fractional Atomic Coordinates (×10^4) and Equivalent Isotropic Displacement Parameters (Å^2×10^3) for HJL-05-006-P. U_{eq} is defined as 1/3 of the trace of the orthogonalised U_{ij}.

Atom	x	y	z	U_{eq}
O1_3	4471(10)	3186(7)	106(3)	51.2(17)
N1_3	8500(13)	718(6)	1198(4)	62(2)

Atom	x	y	z	U_{eq}
N2_3	7572(10)	4910(5)	574(3)	46.0(11)
C1_3	8279(13)	1654(5)	619(3)	60.6(19)
C2_3	7470(9)	2758(4)	1019(3)	59.9(10)
C3_3	5741(12)	1917(6)	1556(4)	60.2(19)
C4_3	6342(18)	548(6)	1588(5)	71(3)
C5_3	6293(8)	3606(5)	510(2)	49.8(10)
C6_3	9551(10)	5057(4)	1081(3)	49.0(10)
C7_3	9580(9)	3800(5)	1365(3)	49.9(10)
C8_3	11412(11)	3668(6)	1852(3)	55.2(11)
C9_3	13245(15)	4825(7)	2058(6)	54.7(9)
C10_3	13187(15)	6080(7)	1784(6)	54.8(10)
C11_3	11324(12)	6204(5)	1282(4)	50.6(11)
C12_3	8840(20)	-542(7)	923(7)	103(6)
O1_5	10590(20)	1983(10)	4986(7)	56(3)
N1_5	7830(20)	4482(8)	3506(5)	42(2)
N2_5	7833(17)	266(7)	4369(6)	45(3)
C1_5	9141(16)	3450(8)	3630(5)	44(2)
C2_5	7509(13)	2456(6)	4117(4)	42(2)
C3_5	6550(20)	3416(8)	4598(5)	46(3)
C4_5	7080(30)	4766(8)	4210(6)	50(3)
C5_5	8853(15)	1575(8)	4547(5)	38(2)
C6_5	5889(17)	142(6)	3851(6)	35(2)
C7_5	5612(15)	1422(6)	3680(5)	39(3)
C8_5	3761(16)	1567(9)	3203(6)	44(4)
C9_5	2160(20)	401(11)	2894(9)	42(3)
C10_5	2390(30)	-873(9)	3087(14)	53(4)
C11_5	4310(20)	-1012(7)	3563(8)	55(4)
C12_5	9260(30)	5669(10)	3166(8)	77(5)
O1_6	10110(20)	1682(11)	4998(7)	56(3)
N1_6	6950(20)	4215(8)	3653(5)	42(2)
N2_6	7331(17)	-28(7)	4385(6)	45(3)
C1_6	8334(17)	3206(8)	3716(5)	44(2)
C2_6	6843(13)	2153(6)	4207(4)	42(2)
C3_6	5920(20)	3049(9)	4738(5)	47(3)
C4_6	6330(30)	4428(8)	4384(6)	49(3)
C5_6	8317(15)	1275(8)	4582(5)	37(2)
C6_6	5293(17)	-151(6)	3904(6)	35(2)
C7_6	4916(14)	1124(7)	3778(5)	39(3)
C8_6	3017(16)	1271(9)	3322(6)	43(4)
C9_6	1470(20)	112(11)	2988(9)	42(3)
C10_6	1830(40)	-1158(10)	3122(14)	53(4)
C11_6	3780(20)	-1298(7)	3584(8)	55(4)
C12_6	8260(30)	5444(10)	3321(8)	77(5)
O1_4	4340(30)	2746(17)	259(11)	51.2(17)
N1_4	6270(40)	404(10)	1024(10)	62(2)
N2_4	6910(20)	4679(12)	744(9)	46.0(11)
C1_4	7230(30)	1616(11)	1470(7)	60.6(19)
C2_4	8170(20)	2691(9)	908(6)	59.9(10)
C3_4	9130(30)	1880(13)	343(9)	60.2(19)
C4_4	8070(40)	396(12)	513(11)	71(3)
C5_4	6230(20)	3340(12)	595(7)	49.8(10)
C6_4	9170(20)	5037(9)	1136(8)	49.0(10)
C7_4	10000(20)	3887(10)	1250(8)	49.9(10)
C8_4	12080(20)	3950(14)	1679(10)	55.2(11)
C9_4	13320(40)	5197(17)	1999(16)	54.7(9)
C10_4	12390(40)	6317(16)	1927(15)	54.8(10)
C11_4	10320(30)	6257(10)	1468(11)	50.6(11)
C12_4	5680(60)	-808(12)	1453(14)	103(6)

Table 9: Anisotropic Displacement Parameters (×10⁴) for HJL-05-006-P. The anisotropic displacement factor exponent takes the form: $-2\pi^2[h^2a^{*2} \times U_{11} + ... + 2hka^* \times b^* \times U_{12}]$

Atom	U_{11}	U_{22}	U_{33}	U_{23}	U_{13}	U_{12}
O1_3	51(2)	49(3)	54(3)	12(2)	-12(2)	17(2)
N1_3	55(5)	57(4)	76(5)	2(3)	-17(4)	23(4)
N2_3	47.4(18)	54.6(16)	39(2)	4.5(17)	-1.5(17)	19.1(16)
C1_3	53(5)	63(3)	66(4)	0(2)	-17(3)	20(3)
C2_3	57.6(19)	59.0(16)	64(2)	0.5(16)	-19.3(17)	21.9(15)
C3_3	52(5)	63(3)	65(4)	2(2)	-18(3)	21(3)
C4_3	73(3)	67(3)	72(3)	0.4(19)	-1(2)	21(2)
C5_3	48.4(15)	52.7(15)	49.1(16)	1.5(13)	-2.8(13)	16.3(12)
C6_3	48.5(15)	51.3(14)	48.3(16)	2.5(13)	-1.7(13)	16.1(12)
C7_3	49.7(15)	52.4(14)	49.3(16)	4.0(13)	-0.9(13)	17.2(12)
C8_3	55.7(19)	56.1(18)	54(2)	5.1(18)	-8.7(18)	17.6(17)
C9_3	54.3(14)	56.2(14)	54.3(14)	0.3(13)	-0.4(13)	16.1(12)
C10_3	54.2(16)	56.7(15)	54.3(16)	-0.4(15)	-2.2(15)	17.0(15)
C11_3	50.1(14)	51.1(14)	50.7(15)	0.7(12)	2.2(11)	13.8(11)
C12_3	155(17)	63(5)	104(12)	11(6)	38(10)	45(7)
O1_5	56(5)	40(6)	68(5)	4(5)	-29(4)	14(5)
N1_5	43(3)	41(3)	42(3)	3.2(16)	0.3(16)	9.9(17)
N2_5	46(5)	39(3)	49(5)	6(3)	-19(4)	14(3)
C1_5	42(3)	45(3)	43(3)	4.1(18)	0.6(19)	10.3(18)
C2_5	40(3)	42(2)	42(3)	4.7(16)	-0.6(17)	11.0(17)
C3_5	44(9)	51(5)	48(5)	7(3)	3(4)	18(5)
C4_5	50(4)	49(3)	50(3)	2.1(19)	5(2)	13(2)
C5_5	36(3)	39(3)	37(3)	2.0(16)	0.2(16)	9.6(16)
C6_5	34(3)	37(3)	34(3)	7(2)	-4(3)	11(2)
C7_5	35(5)	39(3)	43(6)	12(3)	-2(5)	13(3)
C8_5	41(7)	41(5)	48(8)	11(5)	-9(6)	12(4)
C9_5	41(4)	44(4)	42(4)	5(3)	0(3)	12(3)
C10_5	53(8)	45(4)	56(7)	2(5)	-26(7)	11(4)
C11_5	56(7)	43(4)	60(7)	5(5)	-29(6)	10(4)
C12_5	76(13)	67(7)	87(10)	38(8)	22(9)	11(7)
O1_6	57(5)	40(6)	69(5)	3(5)	-30(4)	15(4)
N1_6	42(3)	41(3)	41(3)	3.2(16)	0.5(16)	9.7(17)
N2_6	47(5)	39(3)	49(5)	6(3)	-18(4)	14(3)
C1_6	42(3)	46(3)	43(3)	6(2)	0(3)	10(2)
C2_6	40(3)	42(3)	42(3)	6(2)	-1(2)	11(2)
C3_6	44(9)	51(5)	48(5)	7(3)	3(4)	18(5)
C4_6	50(4)	48(4)	49(3)	3(2)	6(3)	12(3)
C5_6	36(3)	39(3)	36(3)	2.2(16)	0.1(16)	9.6(16)
C6_6	34(3)	36(3)	34(3)	5.3(18)	-2.2(19)	10.5(18)
C7_6	35(5)	39(3)	43(6)	12(3)	-2(5)	13(3)
C8_6	41(7)	41(5)	48(8)	11(5)	-8(6)	12(4)
C9_6	41(3)	44(3)	42(3)	4.1(19)	1(2)	11(2)
C10_6	53(8)	45(4)	56(7)	2(5)	-26(7)	11(4)
C11_6	57(7)	43(4)	60(7)	4(5)	-29(6)	10(4)
C12_6	77(13)	67(7)	87(10)	38(8)	23(9)	11(7)
O1_4	51(2)	49(3)	54(3)	12(2)	-12(2)	17(2)
N1_4	55(5)	57(4)	76(5)	2(3)	-17(4)	23(4)
N2_4	47.4(18)	54.6(16)	39(2)	4.5(17)	-1.5(17)	19.1(16)
C1_4	53(5)	63(3)	66(4)	0(2)	-17(3)	20(3)
C2_4	57.6(19)	59.0(16)	64(2)	0.5(16)	-19.3(17)	21.9(15)
C3_4	52(5)	63(3)	65(4)	2(2)	-18(3)	21(3)
C4_4	73(3)	67(3)	72(3)	0.4(19)	-1(2)	21(2)
C5_4	48.4(15)	52.7(15)	49.1(16)	1.5(13)	-2.8(13)	16.3(12)

Atom	U_{11}	U_{22}	U_{33}	U_{23}	U_{13}	U_{12}
C6_4	48.5(15)	51.3(14)	48.3(16)	2.5(13)	-1.7(13)	16.1(12)
C7_4	49.7(15)	52.4(14)	49.3(16)	4.0(13)	-0.9(13)	17.2(12)
C8_4	55.7(19)	56.1(18)	54(2)	5.1(18)	-8.7(18)	17.6(17)
C9_4	54.3(14)	56.2(14)	54.3(14)	0.3(13)	-0.4(13)	16.1(12)
C10_4	54.2(16)	56.7(15)	54.3(16)	-0.4(15)	-2.2(15)	17.0(15)
C11_4	50.1(14)	51.1(14)	50.7(15)	0.7(12)	2.2(11)	13.8(11)
C12_4	155(17)	63(5)	104(12)	11(6)	38(10)	45(7)

Table 10: Bond Lengths in Å for HJL-05-006-P.

Atom	Atom	Length/Å
O1_3	C5_3	1.234(2)
N1_3	C1_3	1.463(2)
N1_3	C4_3	1.453(2)
N1_3	C12_3	1.455(2)
N2_3	H2_3	1.0000
N2_3	C5_3	1.356(2)
N2_3	C6_3	1.409(2)
C1_3	H1A_3	1.0980
C1_3	H1B_3	1.0980
C1_3	C2_3	1.539(2)
C2_3	C3_3	1.556(2)
C2_3	C5_3	1.526(2)
C2_3	C7_3	1.509(2)
C3_3	H3A_3	1.0980
C3_3	H3B_3	1.0980
C3_3	C4_3	1.533(2)
C4_3	H4A_3	1.0980
C4_3	H4B_3	1.0980
C6_3	C7_3	1.399(2)
C6_3	C11_3	1.382(2)
C7_3	C8_3	1.379(2)
C8_3	H8_3	1.0800
C8_3	C9_3	1.409(2)
C9_3	H9_3	1.0800
C9_3	C10_3	1.393(2)
C10_3	H10_3	1.0800
C10_3	C11_3	1.407(2)
C11_3	H11_3	1.0800
C12_3	H12A_3	1.0981
C12_3	H12B_3	1.0981
C12_3	H12C_3	1.0981
O1_5	C5_5	1.233(2)
N1_5	C1_5	1.461(2)
N1_5	C4_5	1.454(2)
N1_5	C12_5	1.456(2)
N2_5	H2_5	1.0000
N2_5	C5_5	1.355(2)
N2_5	C6_5	1.409(2)
C1_5	H1A_5	1.0980
C1_5	H1B_5	1.0980
C1_5	C2_5	1.538(2)
C2_5	C3_5	1.554(2)
C2_5	C5_5	1.528(2)
C2_5	C7_5	1.508(2)
C3_5	H3A_5	1.0980

Atom	Atom	Length/Å
C3_5	H3B_5	1.0980
C3_5	C4_5	1.532(2)
C4_5	H4B_5	1.0980
C4_5	H4A_5	1.0980
C6_5	C7_5	1.399(2)
C6_5	C11_5	1.382(2)
C7_5	C8_5	1.379(2)
C8_5	H8_5	1.0800
C8_5	C9_5	1.409(2)
C9_5	H9_5	1.0800
C9_5	C10_5	1.393(2)
C10_5	H10_5	1.0800
C10_5	C11_5	1.407(2)
C11_5	H11_5	1.0800
C12_5	H12A_5	1.0981
C12_5	H12B_5	1.0981
C12_5	H12C_5	1.0981
O1_6	C5_6	1.233(2)
N1_6	C1_6	1.461(2)
N1_6	C4_6	1.454(2)
N1_6	C12_6	1.456(2)
N2_6	H2_6	1.0000
N2_6	C5_6	1.355(2)
N2_6	C6_6	1.408(2)
C1_6	H1A_6	1.0980
C1_6	H1B_6	1.0980
C1_6	C2_6	1.538(2)
C2_6	C3_6	1.555(2)
C2_6	C5_6	1.529(2)
C2_6	C7_6	1.508(2)
C3_6	H3A_6	1.0980
C3_6	H3B_6	1.0980
C3_6	C4_6	1.532(2)
C4_6	H4A_6	1.0980
C4_6	H4B_6	1.0980
C6_6	C7_6	1.399(2)
C6_6	C11_6	1.382(2)
C7_6	C8_6	1.379(2)
C8_6	H8_6	1.0780
C8_6	C9_6	1.409(2)
C9_6	H9_6	1.0780
C9_6	C10_6	1.393(2)
C10_6	H10_6	1.0780
C10_6	C11_6	1.407(2)
C11_6	H11_6	1.0780

Atom	Atom	Length/Å
C12_6	H12C_6	1.0981
C12_6	H12B_6	1.0981
C12_6	H12A_6	1.0981
O1_4	C5_4	1.234(2)
N1_4	C1_4	1.460(2)
N1_4	C4_4	1.454(2)
N1_4	C12_4	1.456(2)
N2_4	H2_4	1.0000
N2_4	C5_4	1.355(2)
N2_4	C6_4	1.410(2)
C1_4	H1A_4	1.0980
C1_4	H1B_4	1.0980
C1_4	C2_4	1.538(2)
C2_4	C3_4	1.554(2)
C2_4	C5_4	1.527(2)
C2_4	C7_4	1.510(2)
C3_4	H3A_4	1.0980
C3_4	H3B_4	1.0980
C3_4	C4_4	1.533(2)
C4_4	H4A_4	1.0980
C4_4	H4B_4	1.0980
C6_4	C7_4	1.398(2)
C6_4	C11_4	1.383(2)
C7_4	C8_4	1.380(2)
C8_4	H8_4	1.0780
C8_4	C9_4	1.409(2)
C9_4	H9_4	1.0780
C9_4	C10_4	1.394(2)
C10_4	H10_4	1.0780
C10_4	C11_4	1.407(2)
C11_4	H11_4	1.0780
C12_4	H12A_4	1.0981
C12_4	H12B_4	1.0981
C12_4	H12C_4	1.0981

Table 11: Bond Angles in ° for HJL-05-006-P.

Atom	Atom	Atom	Angle/°
C4_3	N1_3	C1_3	105.4(3)
C4_3	N1_3	C12_3	113.4(3)
C12_3	N1_3	C1_3	112.3(3)
C5_3	N2_3	H2_3	124.5
C5_3	N2_3	C6_3	111.0(2)
C6_3	N2_3	H2_3	124.5
N1_3	C1_3	H1A_3	111.3
N1_3	C1_3	H1B_3	111.3
N1_3	C1_3	C2_3	102.3(2)
H1A_3	C1_3	H1B_3	109.2
C2_3	C1_3	H1A_3	111.3
C2_3	C1_3	H1B_3	111.3
C1_3	C2_3	C3_3	101.76(19)
C5_3	C2_3	C1_3	112.9(2)
C5_3	C2_3	C3_3	113.8(2)
C7_3	C2_3	C1_3	111.7(2)
C7_3	C2_3	C3_3	115.2(2)
C7_3	C2_3	C5_3	102.04(16)
C2_3	C3_3	H3A_3	110.7
C2_3	C3_3	H3B_3	110.7
H3A_3	C3_3	H3B_3	108.8
C4_3	C3_3	C2_3	105.31(19)
C4_3	C3_3	H3A_3	110.7
C4_3	C3_3	H3B_3	110.7
N1_3	C4_3	C3_3	105.2(2)
N1_3	C4_3	H4A_3	110.7
N1_3	C4_3	H4B_3	110.7
C3_3	C4_3	H4A_3	110.7
C3_3	C4_3	H4B_3	110.7
H4A_3	C4_3	H4B_3	108.8
O1_3	C5_3	N2_3	125.0(3)
O1_3	C5_3	C2_3	126.0(3)
N2_3	C5_3	C2_3	109.01(18)
C7_3	C6_3	N2_3	109.39(19)
C11_3	C6_3	N2_3	129.0(2)
C11_3	C6_3	C7_3	121.6(2)
C6_3	C7_3	C2_3	108.56(17)
C8_3	C7_3	C2_3	130.8(2)
C8_3	C7_3	C6_3	120.5(2)
C7_3	C8_3	H8_3	120.7
C7_3	C8_3	C9_3	118.6(3)
C9_3	C8_3	H8_3	120.7
C8_3	C9_3	H9_3	119.6
C10_3	C9_3	C8_3	120.7(3)
C10_3	C9_3	H9_3	119.6
C9_3	C10_3	H10_3	119.8
C9_3	C10_3	C11_3	120.3(3)
C11_3	C10_3	H10_3	119.8
C6_3	C11_3	C10_3	118.2(3)
C6_3	C11_3	H11_3	120.9
C10_3	C11_3	H11_3	120.9
N1_3	C12_3	H12A_3	109.5
N1_3	C12_3	H12B_3	109.5
N1_3	C12_3	H12C_3	109.5
H12A_3	C12_3	H12B_3	109.5
H12A_3	C12_3	H12C_3	109.5
H12B_3	C12_3	H12C_3	109.5
C4_5	N1_5	C1_5	105.5(3)
C4_5	N1_5	C12_5	113.3(3)
C12_5	N1_5	C1_5	112.3(3)
C5_5	N2_5	H2_5	124.5
C5_5	N2_5	C6_5	111.0(2)
C6_5	N2_5	H2_5	124.5
N1_5	C1_5	H1A_5	111.2
N1_5	C1_5	H1B_5	111.2
N1_5	C1_5	C2_5	102.6(2)
H1A_5	C1_5	H1B_5	109.2
C2_5	C1_5	H1A_5	111.2
C2_5	C1_5	H1B_5	111.2
C1_5	C2_5	C3_5	102.01(19)

Atom	Atom	Atom	Angle/°
C5_5	C2_5	C1_5	112.6(2)
C5_5	C2_5	C3_5	113.7(2)
C7_5	C2_5	C1_5	111.7(2)
C7_5	C2_5	C3_5	115.3(3)
C7_5	C2_5	C5_5	101.99(16)
C2_5	C3_5	H3A_5	110.7
C2_5	C3_5	H3B_5	110.7
H3A_5	C3_5	H3B_5	108.8
C4_5	C3_5	C2_5	105.41(19)
C4_5	C3_5	H3A_5	110.7
C4_5	C3_5	H3B_5	110.7
N1_5	C4_5	C3_5	105.2(2)
N1_5	C4_5	H4B_5	110.7
N1_5	C4_5	H4A_5	110.7
C3_5	C4_5	H4B_5	110.7
C3_5	C4_5	H4A_5	110.7
H4B_5	C4_5	H4A_5	108.8
O1_5	C5_5	N2_5	125.3(3)
O1_5	C5_5	C2_5	125.7(3)
N2_5	C5_5	C2_5	109.00(18)
C7_5	C6_5	N2_5	109.37(19)
C11_5	C6_5	N2_5	128.9(2)
C11_5	C6_5	C7_5	121.6(2)
C6_5	C7_5	C2_5	108.60(17)
C8_5	C7_5	C2_5	130.8(2)
C8_5	C7_5	C6_5	120.5(2)
C7_5	C8_5	H8_5	120.7
C7_5	C8_5	C9_5	118.6(3)
C9_5	C8_5	H8_5	120.7
C8_5	C9_5	H9_5	119.7
C10_5	C9_5	C8_5	120.7(3)
C10_5	C9_5	H9_5	119.7
C9_5	C10_5	H10_5	119.9
C9_5	C10_5	C11_5	120.3(3)
C11_5	C10_5	H10_5	119.9
C6_5	C11_5	C10_5	118.2(3)
C6_5	C11_5	H11_5	120.9
C10_5	C11_5	H11_5	120.9
N1_5	C12_5	H12A_5	109.5
N1_5	C12_5	H12B_5	109.5
N1_5	C12_5	H12C_5	109.5
H12A_5	C12_5	H12B_5	109.5
H12A_5	C12_5	H12C_5	109.5
H12B_5	C12_5	H12C_5	109.5
C4_6	N1_6	C1_6	105.4(3)
C4_6	N1_6	C12_6	113.3(3)
C12_6	N1_6	C1_6	112.3(3)
C5_6	N2_6	H2_6	124.5
C5_6	N2_6	C6_6	111.0(2)
C6_6	N2_6	H2_6	124.5
N1_6	C1_6	H1A_6	111.2
N1_6	C1_6	H1B_6	111.2
N1_6	C1_6	C2_6	102.7(2)
H1A_6	C1_6	H1B_6	109.1
C2_6	C1_6	H1A_6	111.2
C2_6	C1_6	H1B_6	111.2
C1_6	C2_6	C3_6	102.05(19)
C5_6	C2_6	C1_6	112.6(2)

Atom	Atom	Atom	Angle/°
C5_6	C2_6	C3_6	113.6(2)
C7_6	C2_6	C1_6	111.8(2)
C7_6	C2_6	C3_6	115.3(3)
C7_6	C2_6	C5_6	101.97(16)
C2_6	C3_6	H3A_6	110.7
C2_6	C3_6	H3B_6	110.7
H3A_6	C3_6	H3B_6	108.8
C4_6	C3_6	C2_6	105.42(19)
C4_6	C3_6	H3A_6	110.7
C4_6	C3_6	H3B_6	110.7
N1_6	C4_6	C3_6	105.1(2)
N1_6	C4_6	H4A_6	110.7
N1_6	C4_6	H4B_6	110.7
C3_6	C4_6	H4A_6	110.7
C3_6	C4_6	H4B_6	110.7
H4A_6	C4_6	H4B_6	108.8
O1_6	C5_6	N2_6	125.3(3)
O1_6	C5_6	C2_6	125.7(3)
N2_6	C5_6	C2_6	109.00(18)
C7_6	C6_6	N2_6	109.38(19)
C11_6	C6_6	N2_6	129.0(2)
C11_6	C6_6	C7_6	121.6(2)
C6_6	C7_6	C2_6	108.61(17)
C8_6	C7_6	C2_6	130.9(2)
C8_6	C7_6	C6_6	120.5(2)
C7_6	C8_6	H8_6	120.7
C7_6	C8_6	C9_6	118.6(3)
C9_6	C8_6	H8_6	120.7
C8_6	C9_6	H9_6	119.6
C10_6	C9_6	C8_6	120.7(3)
C10_6	C9_6	H9_6	119.6
C9_6	C10_6	H10_6	119.8
C9_6	C10_6	C11_6	120.3(3)
C11_6	C10_6	H10_6	119.8
C6_6	C11_6	C10_6	118.2(3)
C6_6	C11_6	H11_6	120.9
C10_6	C11_6	H11_6	120.9
N1_6	C12_6	H12C_6	109.5
N1_6	C12_6	H12B_6	109.5
N1_6	C12_6	H12A_6	109.5
H12C_6	C12_6	H12B_6	109.5
H12C_6	C12_6	H12A_6	109.5
H12B_6	C12_6	H12A_6	109.5
C4_4	N1_4	C1_4	105.4(3)
C4_4	N1_4	C12_4	113.3(4)
C12_4	N1_4	C1_4	112.3(3)
C5_4	N2_4	H2_4	124.5
C5_4	N2_4	C6_4	111.0(2)
C6_4	N2_4	H2_4	124.5
N1_4	C1_4	H1A_4	111.2
N1_4	C1_4	H1B_4	111.2
N1_4	C1_4	C2_4	102.8(2)
H1A_4	C1_4	H1B_4	109.1
C2_4	C1_4	H1A_4	111.2
C2_4	C1_4	H1B_4	111.2
C1_4	C2_4	C3_4	102.14(19)
C5_4	C2_4	C1_4	112.6(2)
C5_4	C2_4	C3_4	113.8(2)

Atom	Atom	Atom	Angle/°
C7_4	C2_4	C1_4	111.5(2)
C7_4	C2_4	C3_4	115.2(3)
C7_4	C2_4	C5_4	101.97(16)
C2_4	C3_4	H3A_4	110.7
C2_4	C3_4	H3B_4	110.7
H3A_4	C3_4	H3B_4	108.8
C4_4	C3_4	C2_4	105.45(19)
C4_4	C3_4	H3A_4	110.7
C4_4	C3_4	H3B_4	110.7
N1_4	C4_4	C3_4	105.0(2)
N1_4	C4_4	H4A_4	110.7
N1_4	C4_4	H4B_4	110.7
C3_4	C4_4	H4A_4	110.7
C3_4	C4_4	H4B_4	110.7
H4A_4	C4_4	H4B_4	108.8
O1_4	C5_4	N2_4	125.2(3)
O1_4	C5_4	C2_4	125.8(3)
N2_4	C5_4	C2_4	109.04(19)
C7_4	C6_4	N2_4	109.35(19)
C11_4	C6_4	N2_4	128.5(3)
C11_4	C6_4	C7_4	121.5(2)

Atom	Atom	Atom	Angle/°
C6_4	C7_4	C2_4	108.60(18)
C8_4	C7_4	C2_4	130.5(3)
C8_4	C7_4	C6_4	120.6(2)
C7_4	C8_4	H8_4	120.7
C7_4	C8_4	C9_4	118.6(3)
C9_4	C8_4	H8_4	120.7
C8_4	C9_4	H9_4	119.7
C10_4	C9_4	C8_4	120.6(3)
C10_4	C9_4	H9_4	119.7
C9_4	C10_4	H10_4	119.9
C9_4	C10_4	C11_4	120.3(3)
C11_4	C10_4	H10_4	119.9
C6_4	C11_4	C10_4	118.2(3)
C6_4	C11_4	H11_4	120.9
C10_4	C11_4	H11_4	120.9
N1_4	C12_4	H12A_4	109.5
N1_4	C12_4	H12B_4	109.5
N1_4	C12_4	H12C_4	109.5
H12A_4	C12_4	H12B_4	109.5
H12A_4	C12_4	H12C_4	109.5
H12B_4	C12_4	H12C_4	109.5

Table 12: Torsion Angles in ° for HJL-05-006-P.

Atom	Atom	Atom	Atom	Angle/°
N1_3	C1_3	C2_3	C3_3	-38.0(4)
N1_3	C1_3	C2_3	C5_3	-160.3(4)
N1_3	C1_3	C2_3	C7_3	85.4(4)
N2_3	C6_3	C7_3	C2_3	-0.04(10)
N2_3	C6_3	C7_3	C8_3	177.2(5)
N2_3	C6_3	C11_3	C10_3	-177.4(6)
C1_3	N1_3	C4_3	C3_3	-34.1(7)
C1_3	C2_3	C3_3	C4_3	17.7(6)
C1_3	C2_3	C5_3	O1_3	60.0(3)
C1_3	C2_3	C5_3	N2_3	-120.1(3)
C1_3	C2_3	C7_3	C6_3	120.9(3)
C1_3	C2_3	C7_3	C8_3	-56.0(6)
C2_3	C3_3	C4_3	N1_3	8.9(7)
C2_3	C7_3	C8_3	C9_3	176.6(6)
C3_3	C2_3	C5_3	O1_3	-55.3(3)
C3_3	C2_3	C5_3	N2_3	124.6(3)
C3_3	C2_3	C7_3	C6_3	-123.7(3)
C3_3	C2_3	C7_3	C8_3	59.4(6)
C4_3	N1_3	C1_3	C2_3	45.8(5)
C5_3	N2_3	C6_3	C7_3	-0.06(13)
C5_3	N2_3	C6_3	C11_3	177.6(5)
C5_3	C2_3	C3_3	C4_3	139.4(5)
C5_3	C2_3	C7_3	C6_3	0.11(10)
C5_3	C2_3	C7_3	C8_3	-176.8(5)
C6_3	N2_3	C5_3	O1_3	-179.98(11)
C6_3	N2_3	C5_3	C2_3	0.13(14)
C6_3	C7_3	C8_3	C9_3	0.04(15)
C7_3	C2_3	C3_3	C4_3	-103.3(6)
C7_3	C2_3	C5_3	O1_3	179.97(9)
C7_3	C2_3	C5_3	N2_3	-0.15(12)
C7_3	C6_3	C11_3	C10_3	0.04(15)

Atom	Atom	Atom	Atom	Angle/°
C7_3	C8_3	C9_3	C10_3	1.1(10)
C8_3	C9_3	C10_3	C11_3	-1.7(14)
C9_3	C10_3	C11_3	C6_3	1.1(10)
C11_3	C6_3	C7_3	C2_3	-177.9(5)
C11_3	C6_3	C7_3	C8_3	-0.6(5)
C12_3	N1_3	C1_3	C2_3	169.7(5)
C12_3	N1_3	C4_3	C3_3	-157.3(6)
N1_5	C1_5	C2_5	C3_5	-36.7(5)
N1_5	C1_5	C2_5	C5_5	-158.9(4)
N1_5	C1_5	C2_5	C7_5	87.1(5)
N2_5	C6_5	C7_5	C2_5	-0.01(10)
N2_5	C6_5	C7_5	C8_5	-177.5(8)
N2_5	C6_5	C11_5	C10_5	175.4(12)
C1_5	N1_5	C4_5	C3_5	-34.6(7)
C1_5	C2_5	C3_5	C4_5	16.1(6)
C1_5	C2_5	C5_5	O1_5	60.1(3)
C1_5	C2_5	C5_5	N2_5	-119.9(3)
C1_5	C2_5	C7_5	C6_5	120.5(3)
C1_5	C2_5	C7_5	C8_5	-62.4(9)
C2_5	C3_5	C4_5	N1_5	10.2(8)
C2_5	C7_5	C8_5	C9_5	-176.9(10)
C3_5	C2_5	C5_5	O1_5	-55.2(3)
C3_5	C2_5	C5_5	N2_5	124.7(3)
C3_5	C2_5	C7_5	C6_5	-123.6(3)
C3_5	C2_5	C7_5	C8_5	53.5(9)
C4_5	N1_5	C1_5	C2_5	45.2(5)
C5_5	N2_5	C6_5	C7_5	-0.01(13)
C5_5	N2_5	C6_5	C11_5	-175.9(11)
C5_5	C2_5	C3_5	C4_5	137.5(6)
C5_5	C2_5	C7_5	C6_5	0.03(10)
C5_5	C2_5	C7_5	C8_5	177.2(9)
C6_5	N2_5	C5_5	O1_5	-179.99(11)
C6_5	N2_5	C5_5	C2_5	0.03(14)
C6_5	C7_5	C8_5	C9_5	0.01(15)
C7_5	C2_5	C3_5	C4_5	-105.2(6)
C7_5	C2_5	C5_5	O1_5	179.98(9)
C7_5	C2_5	C5_5	N2_5	-0.03(13)
C7_5	C6_5	C11_5	C10_5	0.00(15)
C7_5	C8_5	C9_5	C10_5	2.5(18)
C8_5	C9_5	C10_5	C11_5	-4(3)
C9_5	C10_5	C11_5	C6_5	2.5(18)
C11_5	C6_5	C7_5	C2_5	176.2(10)
C11_5	C6_5	C7_5	C8_5	-1.3(9)
C12_5	N1_5	C1_5	C2_5	169.1(5)
C12_5	N1_5	C4_5	C3_5	-157.9(7)
N1_6	C1_6	C2_6	C3_6	-36.5(5)
N1_6	C1_6	C2_6	C5_6	-158.6(4)
N1_6	C1_6	C2_6	C7_6	87.3(5)
N2_6	C6_6	C7_6	C2_6	-0.02(10)
N2_6	C6_6	C7_6	C8_6	-180.0(8)
N2_6	C6_6	C11_6	C10_6	179.4(12)
C1_6	N1_6	C4_6	C3_6	-34.9(7)
C1_6	C2_6	C3_6	C4_6	15.7(6)
C1_6	C2_6	C5_6	O1_6	60.1(3)
C1_6	C2_6	C5_6	N2_6	-119.9(3)
C1_6	C2_6	C7_6	C6_6	120.5(3)
C1_6	C2_6	C7_6	C8_6	-59.5(9)
C2_6	C3_6	C4_6	N1_6	10.7(8)

Atom	Atom	Atom	Atom	Angle/°
C2_6	C7_6	C8_6	C9_6	-179.9(10)
C3_6	C2_6	C5_6	O1_6	-55.3(3)
C3_6	C2_6	C5_6	N2_6	124.7(3)
C3_6	C2_6	C7_6	C6_6	-123.6(3)
C3_6	C2_6	C7_6	C8_6	56.4(9)
C4_6	N1_6	C1_6	C2_6	45.3(5)
C5_6	N2_6	C6_6	C7_6	-0.01(13)
C5_6	N2_6	C6_6	C11_6	-179.5(11)
C5_6	C2_6	C3_6	C4_6	137.1(6)
C5_6	C2_6	C7_6	C6_6	0.04(10)
C5_6	C2_6	C7_6	C8_6	180.0(9)
C6_6	N2_6	C5_6	O1_6	-179.99(11)
C6_6	N2_6	C5_6	C2_6	0.04(14)
C6_6	C7_6	C8_6	C9_6	0.01(15)
C7_6	C2_6	C3_6	C4_6	-105.7(6)
C7_6	C2_6	C5_6	O1_6	179.98(9)
C7_6	C2_6	C5_6	N2_6	-0.04(13)
C7_6	C6_6	C11_6	C10_6	0.00(15)
C7_6	C8_6	C9_6	C10_6	0.9(19)
C8_6	C9_6	C10_6	C11_6	-1(3)
C9_6	C10_6	C11_6	C6_6	0.9(19)
C11_6	C6_6	C7_6	C2_6	179.5(10)
C11_6	C6_6	C7_6	C8_6	-0.5(9)
C12_6	N1_6	C1_6	C2_6	169.0(5)
C12_6	N1_6	C4_6	C3_6	-158.0(6)
N1_4	C1_4	C2_4	C3_4	-36.0(5)
N1_4	C1_4	C2_4	C5_4	86.4(7)
N1_4	C1_4	C2_4	C7_4	-159.6(6)
N2_4	C6_4	C7_4	C2_4	-0.02(10)
N2_4	C6_4	C7_4	C8_4	-174.0(11)
N2_4	C6_4	C11_4	C10_4	169.9(12)
C1_4	N1_4	C4_4	C3_4	-35.3(7)
C1_4	C2_4	C3_4	C4_4	14.9(7)
C1_4	C2_4	C5_4	O1_4	-60.3(3)
C1_4	C2_4	C5_4	N2_4	119.7(3)
C1_4	C2_4	C7_4	C6_4	-120.4(3)
C1_4	C2_4	C7_4	C8_4	52.7(12)
C2_4	C3_4	C4_4	N1_4	11.4(8)
C2_4	C7_4	C8_4	C9_4	-172.5(13)
C3_4	C2_4	C5_4	O1_4	55.3(3)
C3_4	C2_4	C5_4	N2_4	-124.7(3)
C3_4	C2_4	C7_4	C6_4	123.7(3)
C3_4	C2_4	C7_4	C8_4	-63.1(12)
C4_4	N1_4	C1_4	C2_4	45.2(5)
C5_4	N2_4	C6_4	C7_4	0.02(13)
C5_4	N2_4	C6_4	C11_4	-170.9(11)
C5_4	C2_4	C3_4	C4_4	-106.7(8)
C5_4	C2_4	C7_4	C6_4	0.01(10)
C5_4	C2_4	C7_4	C8_4	173.2(12)
C6_4	N2_4	C5_4	O1_4	179.99(11)
C6_4	N2_4	C5_4	C2_4	-0.01(14)
C6_4	C7_4	C8_4	C9_4	0.01(15)
C7_4	C2_4	C3_4	C4_4	136.0(8)
C7_4	C2_4	C5_4	O1_4	180.00(9)
C7_4	C2_4	C5_4	N2_4	0.00(13)
C7_4	C6_4	C11_4	C10_4	-0.01(15)
C7_4	C8_4	C9_4	C10_4	5(2)
C8_4	C9_4	C10_4	C11_4	-7(3)

Atom	Atom	Atom	Atom	Angle/°
C9_4	C10_4	C11_4	C6_4	5(2)
C11_4	C6_4	C7_4	C2_4	171.6(10)
C11_4	C6_4	C7_4	C8_4	-2.3(11)
C12_4	N1_4	C1_4	C2_4	169.0(5)
C12_4	N1_4	C4_4	C3_4	-158.4(7)

Table 13: Hydrogen Fractional Atomic Coordinates (×10[4]) and Equivalent Isotropic Displacement Parameters (Å[2]×10[3]) for HJL-05-006-P. U_{eq} is defined as 1/3 of the trace of the orthogonalised U_{ij}.

Atom	x	y	z	U_{eq}
H2_3	7164.84	5667.13	297.84	55
H1A_3	6933.35	1167.72	190.67	73
H1B_3	10006.23	2063.5	381.74	73
H3A_3	6036.77	2406.49	2092.24	72
H3B_3	3860.54	1799.37	1360.21	72
H4A_3	6666.2	272.23	2151.12	85
H4B_3	4864.9	-240.16	1330.46	85
H8_3	11440.05	2698.41	2072.09	66
H9_3	14710.83	4738.58	2431.71	66
H10_3	14578.8	6962.13	1958.87	66
H11_3	11280.09	7169.51	1059.33	61
H12A_3	7719.69	-837.3	419.33	154
H12B_3	8324.06	-1330.04	1325.87	154
H12C_3	10737.19	-410.84	818.04	154
H2_5	8387.37	-508.87	4576.77	54
H1A_5	10918.63	3876.61	3903.77	52
H1B_5	9356.75	2963.53	3120.08	52
H3A_5	4608.84	3026.31	4648.49	56
H3B_5	7465.77	3526.88	5140.25	56
H4B_5	5468.49	5148.74	4151.04	59
H4A_5	8514.6	5515.24	4511.15	59
H8_5	3542.52	2554.31	3070.48	52
H9_5	747.98	495.67	2502.66	51
H10_5	1083.94	-1758.38	2871.07	64
H11_5	4542.6	-1995.04	3698.62	66
H12A_5	11171.52	5783.47	3321.53	116
H12B_5	8728.84	6563.61	3343.36	116
H12C_5	8960.58	5554.18	2575.3	116
H2_6	7965.31	-799.84	4557.74	55
H1A_6	10146.39	3640.39	3965.79	53
H1B_6	8475.45	2767.16	3186.13	53
H3A_6	4004.15	2624.22	4814.39	56
H3B_6	6926.59	3130.25	5266.42	56
H4A_6	4687.94	4788.14	4373.09	59
H4B_6	7788.88	5169.91	4678.9	59
H8_6	2721.03	2251.96	3223.82	52
H9_6	-6.3	208.98	2623.59	51
H10_6	610.6	-2037.19	2871.98	64
H11_6	4095.02	-2274.86	3685.47	66
H12C_6	9930.06	5883.13	3651.93	116
H12B_6	7151.86	6166.04	3284.73	116
H12A_6	8703.78	5208.73	2776.47	116
H2_4	5947.02	5327.38	596.5	55
H1A_4	8692.5	1493.01	1852.87	73
H1B_4	5828.85	1871.76	1771.38	73
H3A_4	8547.79	2102.76	-208.67	72

Atom	x	y	z	U_{eq}
H3B_4	11104.44	2128.24	392.4	72
H4A_4	7255.12	-176.36	19.87	85
H4B_4	9468.27	-54.72	753.28	85
H8_4	12727.84	3064.88	1767.64	66
H9_4	15013.85	5284.95	2302.6	66
H10_4	13245.17	7231.95	2224.92	66
H11_4	9659.84	7137.79	1376.28	61
H12A_4	5461.68	-536.67	2012.61	154
H12B_4	7145.18	-1314.06	1445.25	154
H12C_4	4008.06	-1488.96	1225	154

Table 14: Hydrogen Bond information for HJL-05-006-P.

D	H	A	d(D-H)/Å	d(H-A)/Å	d(D-A)/Å	D-H-A/deg
N2_3	H2_3	O1_3[1]	1.00	1.82	2.791(8)	162.5
N2_6	H2_6	O1_6[2]	1.00	1.77	2.726(14)	159.6

––––

[1]1-x,1-y,-z; [2]2-x,-y,1-z

Table 15: Atomic Occupancies for all atoms that are not fully occupied in HJL-05-006-P.

Atom	Occupancy	Atom	Occupancy	Atom	Occupancy
O1_3	0.834(7)	H2_5	0.5	H1B_6	0.5
N1_3	0.834(7)	C1_5	0.5	C2_6	0.5
N2_3	0.834(7)	H1A_5	0.5	C3_6	0.5
H2_3	0.834(7)	H1B_5	0.5	H3A_6	0.5
C1_3	0.834(7)	C2_5	0.5	H3B_6	0.5
H1A_3	0.834(7)	C3_5	0.5	C4_6	0.5
H1B_3	0.834(7)	H3A_5	0.5	H4A_6	0.5
C2_3	0.834(7)	H3B_5	0.5	H4B_6	0.5
C3_3	0.834(7)	C4_5	0.5	C5_6	0.5
H3A_3	0.834(7)	H4B_5	0.5	C6_6	0.5
H3B_3	0.834(7)	H4A_5	0.5	C7_6	0.5
C4_3	0.834(7)	C5_5	0.5	C8_6	0.5
H4A_3	0.834(7)	C6_5	0.5	H8_6	0.5
H4B_3	0.834(7)	C7_5	0.5	C9_6	0.5
C5_3	0.834(7)	C8_5	0.5	H9_6	0.5
C6_3	0.834(7)	H8_5	0.5	C10_6	0.5
C7_3	0.834(7)	C9_5	0.5	H10_6	0.5
C8_3	0.834(7)	H9_5	0.5	C11_6	0.5
H8_3	0.834(7)	C10_5	0.5	H11_6	0.5
C9_3	0.834(7)	H10_5	0.5	C12_6	0.5
H9_3	0.834(7)	C11_5	0.5	H12C_6	0.5
C10_3	0.834(7)	H11_5	0.5	H12B_6	0.5
H10_3	0.834(7)	C12_5	0.5	H12A_6	0.5
C11_3	0.834(7)	H12A_5	0.5	O1_4	0.166(7)
H11_3	0.834(7)	H12B_5	0.5	N1_4	0.166(7)
C12_3	0.834(7)	H12C_5	0.5	N2_4	0.166(7)
H12A_3	0.834(7)	O1_6	0.5	H2_4	0.166(7)
H12B_3	0.834(7)	N1_6	0.5	C1_4	0.166(7)
H12C_3	0.834(7)	N2_6	0.5	H1A_4	0.166(7)
O1_5	0.5	H2_6	0.5	H1B_4	0.166(7)
N1_5	0.5	C1_6	0.5	C2_4	0.166(7)
N2_5	0.5	H1A_6	0.5	C3_4	0.166(7)

Atom	Occupancy
H3A_4	0.166(7)
H3B_4	0.166(7)
C4_4	0.166(7)
H4A_4	0.166(7)
H4B_4	0.166(7)
C5_4	0.166(7)
C6_4	0.166(7)
C7_4	0.166(7)
C8_4	0.166(7)
H8_4	0.166(7)
C9_4	0.166(7)
H9_4	0.166(7)
C10_4	0.166(7)
H10_4	0.166(7)
C11_4	0.166(7)
H11_4	0.166(7)
C12_4	0.166(7)
H12A_4	0.166(7)
H12B_4	0.166(7)
H12C_4	0.166(7)

Citations

CrysAlisPro (ROD), Rigaku Oxford Diffraction, Poland (?).

CrysAlisPro Software System, Rigaku Oxford Diffraction, (2024).

O.V. Dolomanov and L.J. Bourhis and R.J. Gildea and J.A.K. Howard and H. Puschmann, Olex2: A complete structure solution, refinement and analysis program, *J. Appl. Cryst.*, (2009), **42**, 339-341.

Sheldrick, G.M., Crystal structure refinement with ShelXL, *Acta Cryst.*, (2015), **C71**, 3-8.

Sheldrick, G.M., ShelXT-Integrated space-group and crystal-structure determination, *Acta Cryst.*, (2015), **A71**, 3-8.

5-methoxy-1'-methylspiro[indoline-3,3'-pyrrolidin]-2-one (46)

Crystal Data and Experimental

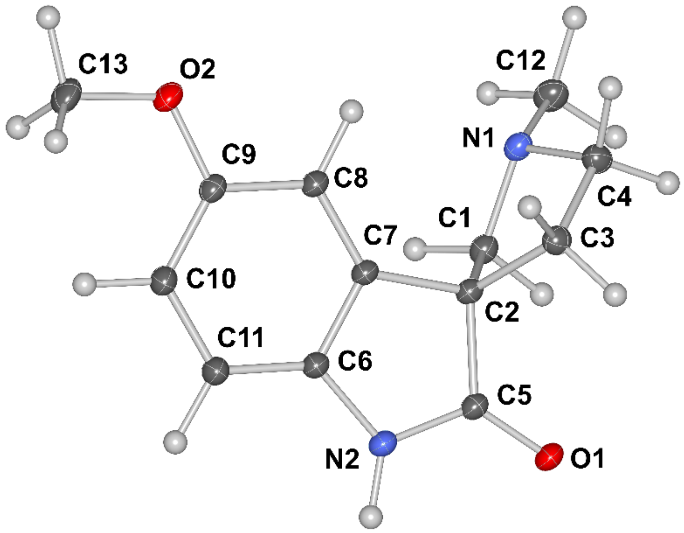

**Compound**	CB-122
Formula	$C_{13}H_{16}N_2O_2$
$D_{calc.}$/ g cm^{-3}	1.341
μ/mm^{-1}	0.743
Formula Weight	232.284
Color	colorless
Shape	needle-shaped
Size/mm^3	0.22×0.08×0.03
T/K	100.00(10)
Crystal System	triclinic
Space Group	P-1
a/Å	5.61760(19)
b/Å	9.9395(4)
c/Å	11.4591(4)
α/°	109.784(4)
β/°	91.652(3)
γ/°	105.485(3)
V/Å^3	575.18(4)
Z	2
Z'	1
Wavelength/Å	1.54184
Radiation type	Cu K_α
Θ_{min}/°	4.14
Θ_{max}/°	76.44
Measured Refl's.	9590
Indep't Refl's	2261
Refl's I≥2 σ(I)	2029
R_{int}	0.0219
Parameters	280
Restraints	297
Largest Peak	0.2760
Deepest Hole	-0.1060
GooF	1.3716
wR_2 (all data)	0.0411
wR_2	0.0403
R_1 (all data)	0.0225
R_1	0.0189

Experimental. Single colorless needle-shaped crystals of CB-122 were chosen from the sample as supplied. A suitable crystal with dimensions 0.22 × 0.08 × 0.03 mm^3 was selected and mounted on a loop with paratone on a XtaLAB Synergy-S diffractometer. The crystal was kept at a constant T = 100.00(10) K during data collection. The structure was solved with the ShelXT 2018/2 (Sheldrick, 2018) and Olex2 1.5-alpha (Dolomanov et al., 2009). The structure was refined with olex2.refine 1.5-alpha (Bourhis et al., 2015) using full matrix least squares minimisation on F^2.

Crystal Data. $C_{13}H_{16}N_2O_2$, M_r = 232.284, triclinic, P-1 (No. 2), a = 5.61760(19) Å, b = 9.9395(4) Å, c = 11.4591(4) Å, α = 109.784(4)°, β = 91.652(3)°, γ = 105.485(3)°, V = 575.18(4) Å^3, T = 100.00(10) K, Z = 2, Z' = 1, μ(Cu K_α) = 0.743, 9590 reflections measured, 2261 unique (R_{int} = 0.0219) which were used in all calculations. The final wR_2 was 0.0411 (all data) and R_1 was 0.0189 (I≥2 σ(I)).

Structure Quality Indicators

**Reflections:**	d min (Cu$K\alpha$) $2\Theta=152.9°$ 0.79	$I/\sigma(I)$ 50.4	Rint m=4.13 2.19%	Full 135.4° 94% to 152.9° 98.3
**Refinement:**	Shift -0.001	Max Peak 0.3	Min Peak -0.1	GooF 1.372

A colourless needle-shaped-shaped crystal with dimensions 0.22 × 0.08 × 0.03 mm^3 was mounted on a loop with paratone. Data were collected using a XtaLAB Synergy, Dualflex, HyPix diffractometer operating at T = 100.0(1) K.

Data were measured using ω scans with Cu K_α radiation. The diffraction pattern was indexed and the total number of runs and images was based on the strategy calculation from the program CrysAlisPro system (CCD 44.57a 64-bit (release 20-06-2024)). The maximum resolution that was achieved was Θ = 76.44° (0.79 Å).

The unit cell was refined using CrysAlisPro 1.171.44.57a (Rigaku OD, 2024) on 5990 reflections, 62% of the observed reflections.

Data reduction, scaling and absorption corrections were performed using CrysAlisPro 1.171.44.57a (Rigaku OD, 2024). The final completeness is 98.31 % out to 76.44° in Θ. A numerical absorption correction based on gaussian integration over a multifaceted crystal model was performed using CrysAlisPro 1.171.42.74a (Rigaku Oxford Diffraction, 2022). An empirical absorption correction using spherical harmonics, implemented in SCALE3 ABSPACK scaling algorithm was also applied. The absorption coefficient μ of this material is 0.743 mm^{-1} at this wavelength (λ = 1.54184Å) and the minimum and maximum transmissions are 0.755 and 1.000.

The structure was solved, and the space group *P*-1 (# 2) determined by the ShelXT 2018/2 (Sheldrick, 2018) and refined by full matrix least squares minimisation on $\boldsymbol{F^2}$ using version of olex2.refine 1.5-alpha (Bourhis et al., 2015). All atoms including hydrogens were refined anisotropically. Hydrogen atom positions were located from the electron density and refined using Hirshfeld scattering factors. Refinement was by using NoSpherA2, an implementation of non-spherical atom-form-factors (F. Kleemiss, H. Puschmann, O. Dolomanov, S.Grabowsky - https://doi.org/10.1039/D0SC05526C – 2020). NoSpherA2 implementation of HAR makes use of tailor-made aspherical atomic form factors calculated from a Hirshfeld-partitioned electron density (ED) not from spherical-atom form factors. The ED was calculated from a Gaussian basis set single determinant SCF wavefunction from DFT using selected functionals for a fragment of this crystal. This fragment was embedded in an electrostatic crystal field by employing cluster charges. The following options were used. SOFTWARE: ORCA 5.0 PARTITIONING: NoSpherA2 INT ACCURACY: Normal METHOD: PBE BASIS SET: def2-TZVP CHARGE: 0 MULTIPLICITY: 1 DATE: 2025-07-25_17-29-11

There is a single formula unit in the asymmetric unit, which is represented by the reported formula. The number of formula units in the unit cell, Z is 2 and the number of formula units, Z' in the symmetry independent unit is 1. The moiety formula is C13 H16 N2 O2.

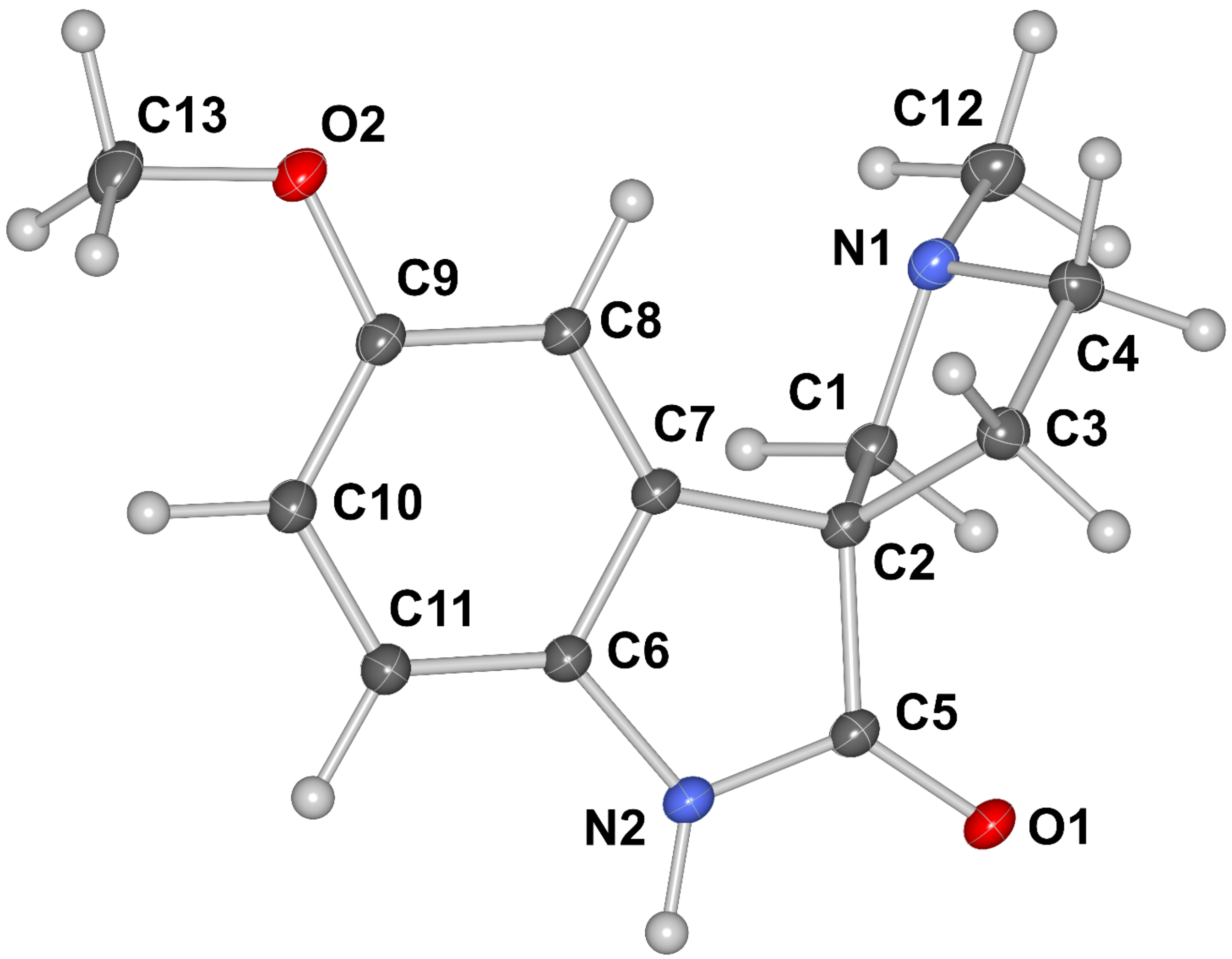

Figure 5 Thermal ellipsoidal representation of the asymmetric unit. The asymmetric unit consists of a one molecule

Data Plots: Diffraction Data

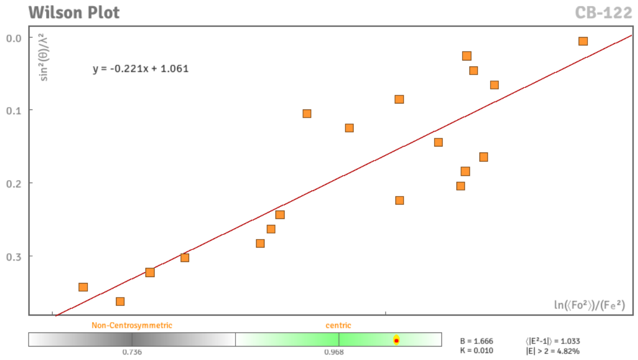

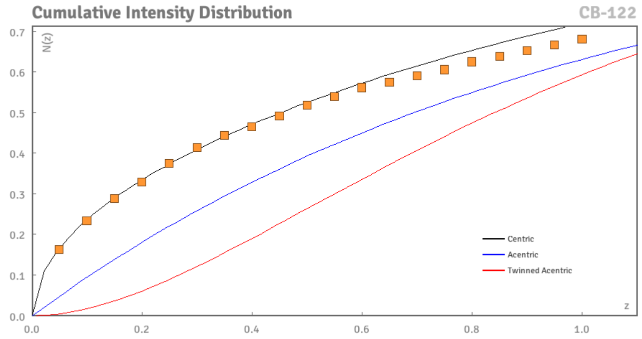

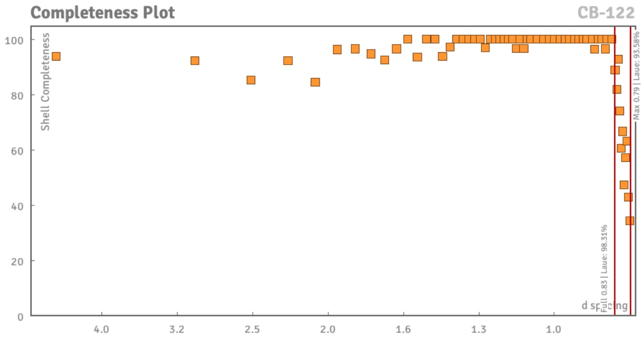

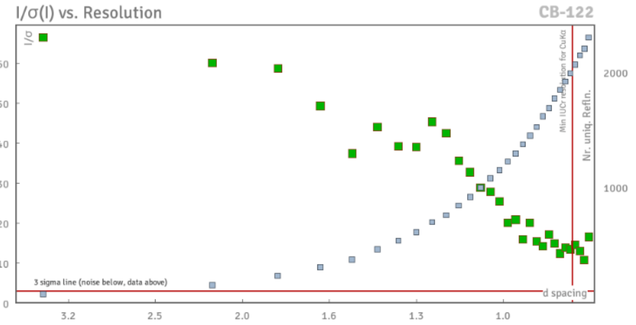

Data Plots: Refinement and Data

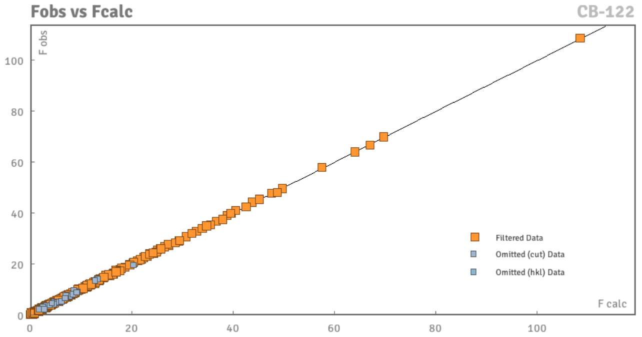

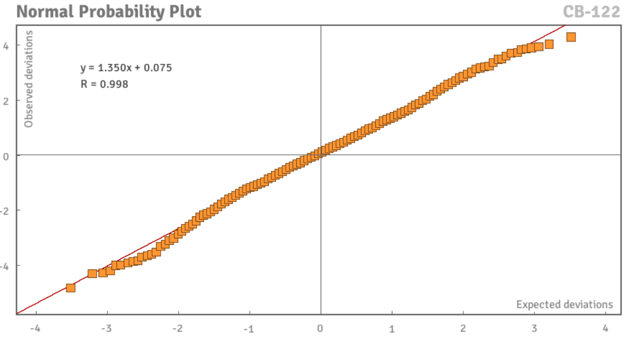

Reflection Statistics

Total reflections (after filtering)	9348	Unique reflections	2261
Completeness	0.936	Mean I/σ	29.45
hkl_{max} collected	(6, 12, 12)	hkl_{min} collected	(-7, -12, -14)
hkl_{max} used	(7, 11, 14)	hkl_{min} used	(-7, -12, 0)
Lim d_{max} collected	100.0	Lim d_{min} collected	0.77
d_{max} used	10.69	d_{min} used	0.79
Friedel pairs	1050	Friedel pairs merged	1
Inconsistent equivalents	1	R_{int}	0.0219
R_{sigma}	0.0198	Intensity transformed	0
Omitted reflections	0	Omitted by user (OMIT hkl)	242
Multiplicity	(1023, 835, 523, 337, 270, 165, 76, 59, 25, 14, 10, 9, 3, 1)	Maximum multiplicity	14
Removed systematic absences	0	Filtered off (Shel/OMIT)	0

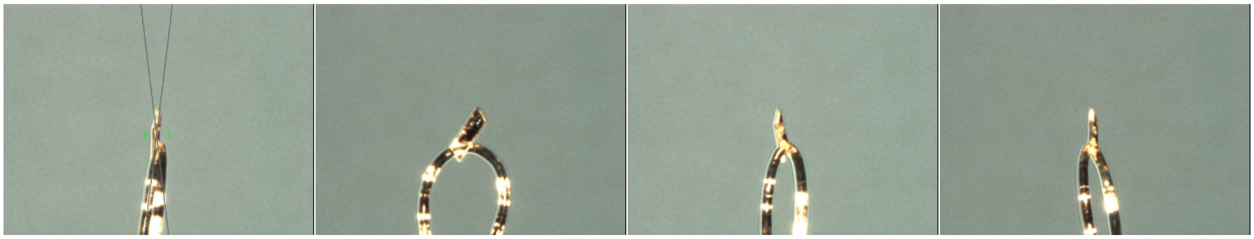

Table 16: Fractional Atomic Coordinates (×10^4) and Equivalent Isotropic Displacement Parameters (Å^2×10^3) for CB-122. U_{eq} is defined as 1/3 of the trace of the orthogonalised U_{ij}.

Atom	x	y	z	U_{eq}
O1	-702.0(7)	3316.3(5)	439.6(4)	18.40(11)
O2	10868.6(7)	6776.2(5)	4505.2(4)	19.10(11)

Atom	x	y	z	U_{eq}
N1	3790.2(9)	1594.4(6)	2235.8(4)	16.12(12)
N2	2774.4(9)	5373.5(6)	1016.5(5)	16.28(12)
C1	3481.4(11)	2181.4(7)	1250.0(6)	15.85(13)
C2	2747.1(10)	3603.0(7)	1957.1(5)	13.72(13)
C3	1137.8(11)	3121.8(7)	2906.7(6)	16.65(14)
C4	1562.5(11)	1639.0(7)	2852.4(6)	18.43(14)
C5	1354.8(10)	4054.8(7)	1052.6(5)	14.73(13)
C6	4978.5(10)	5933.9(7)	1864.9(5)	14.61(13)
C7	5007.3(10)	4949.9(7)	2493.0(5)	13.79(13)
C8	6971.2(10)	5262.4(7)	3388.8(5)	14.75(13)
C9	8967.5(10)	6570.5(7)	3630.7(5)	15.43(13)
C10	8948.1(11)	7536.5(7)	2990.9(6)	17.63(14)
C11	6922.5(11)	7221.6(7)	2091.2(6)	17.49(14)
C12	4136.5(12)	115.9(7)	1753.3(6)	21.68(15)
C13	12907.1(12)	8094.4(8)	4828.8(7)	23.24(15)

Table 17: Anisotropic Displacement Parameters (×10[4]) for CB-122. The anisotropic displacement factor exponent takes the form: $-2\pi^2[h^2a^{*2} \times U_{11} + ... + 2hka^* \times b^* \times U_{12}]$

Atom	U_{11}	U_{22}	U_{33}	U_{23}	U_{13}	U_{12}
O1	13.4(2)	20.6(2)	19.8(2)	2.03(18)	-3.22(17)	8.38(19)
O2	14.7(2)	22.1(2)	17.7(2)	2.19(18)	-3.04(17)	6.47(19)
N1	13.3(2)	15.7(3)	18.5(3)	4.2(2)	0.0(2)	5.3(2)
N2	13.4(2)	18.3(3)	17.0(3)	3.2(2)	-1.5(2)	7.6(2)
H2	32(5)	40(4)	54(5)	0(3)	-15(3)	31(2)
C1	14.2(3)	15.7(3)	15.9(3)	4.3(3)	0.9(2)	3.6(3)
H1a	27(3)	24(3)	25(3)	4.4(18)	-5.9(16)	2.5(19)
H1b	30(3)	31(4)	31(4)	10.8(18)	13.8(15)	12(2)
C2	12.3(3)	14.5(3)	13.7(3)	4.2(2)	0.1(2)	4.2(2)
C3	15.4(3)	18.4(3)	17.2(3)	5.6(3)	2.8(2)	7.1(3)
H3a	41(4)	30(3)	23(3)	9(2)	1.6(16)	3.5(15)
H3b	21(2)	42(4)	40(4)	11.5(15)	3.6(15)	18(3)
C4	17.1(3)	20.0(3)	20.8(3)	6.3(3)	3.1(3)	9.9(3)
H4a	25(3)	27(3)	47(4)	2.6(16)	-3.6(18)	9.5(19)
H4b	26(4)	48(4)	24(3)	19(3)	8.1(16)	22.8(15)
C5	12.6(3)	16.5(3)	14.3(3)	4.0(2)	-0.2(2)	4.9(2)
C6	13.1(3)	15.7(3)	14.7(3)	3.4(2)	-0.1(2)	5.8(2)
C7	12.5(3)	14.8(3)	13.3(3)	3.7(2)	-0.2(2)	4.5(2)
C8	13.1(3)	15.9(3)	14.2(3)	3.4(2)	-0.7(2)	4.9(2)
H8	33(4)	26(4)	33(4)	5(3)	-3(3)	18.0(18)
C9	12.8(3)	16.9(3)	14.2(3)	2.8(2)	-0.3(2)	4.0(2)
C10	14.8(3)	18.1(3)	18.3(3)	1.6(3)	-0.6(2)	7.1(3)
H10	23(3)	35(3)	53(5)	-8.2(18)	-10(2)	25(2)
C11	15.4(3)	17.4(3)	19.2(3)	2.2(3)	-0.8(2)	8.2(3)
H11	32(4)	28(3)	31(4)	-1(3)	-9(3)	19.2(18)
C12	20.9(3)	18.6(3)	25.4(4)	8.3(3)	0.4(3)	6.2(3)
H12a	30(3)	32(4)	41(4)	0.9(18)	-7.5(18)	7(2)
H12b	30(3)	29(4)	46(4)	13(2)	12.6(17)	11(2)
H12c	45(4)	38(4)	34(3)	16(3)	1(2)	16.7(17)
C13	16.3(3)	25.8(4)	21.4(4)	0.1(3)	-3.2(3)	6.0(3)
H13a	32(4)	51(5)	35(3)	1(2)	6.1(17)	14(2)
H13b	32(4)	42(4)	41(4)	1(2)	-12.2(18)	15(2)
H13c	33(4)	27(3)	44(4)	4.5(16)	-2(3)	7(2)

Table 18: Bond Lengths in Å for CB-122.

Atom	Atom	Length/Å
O1	C5	1.2323(7)
O2	C9	1.3673(7)
O2	C13	1.4181(8)
N1	C1	1.4613(8)
N1	C4	1.4583(8)
N1	C12	1.4533(8)
N2	H2	0.995(8)
N2	C5	1.3550(8)
N2	C6	1.4086(7)
C1	H1a	1.106(7)
C1	H1b	1.080(7)
C1	C2	1.5424(8)
C2	C3	1.5518(8)
C2	C5	1.5309(8)
C2	C7	1.5074(8)
C3	H3a	1.085(5)
C3	H3b	1.085(5)
C3	C4	1.5361(9)
C4	H4a	1.099(5)
C4	H4b	1.099(5)
C6	C7	1.3997(8)
C6	C11	1.3823(8)
C7	C8	1.3796(8)
C8	H8	1.078(7)
C8	C9	1.4088(9)
C9	C10	1.3933(9)
C10	H10	1.084(7)
C10	C11	1.4069(8)
C11	H11	1.088(7)
C12	H12a	1.107(4)
C12	H12b	1.107(4)
C12	H12c	1.107(4)
C13	H13a	1.079(8)
C13	H13b	1.085(7)
C13	H13c	1.125(8)

Table 19: Bond Angles in ° for CB-122.

Atom	Atom	Atom	Angle/°
C13	O2	C9	118.05(5)
C4	N1	C1	104.07(4)
C12	N1	C1	112.53(5)
C12	N1	C4	113.84(5)
C5	N2	H2	121.8(5)
C6	N2	H2	127.0(5)
C6	N2	C5	110.90(5)
H1a	C1	N1	111.5(4)
H1b	C1	N1	110.9(4)
H1b	C1	H1a	110.1(5)
C2	C1	N1	102.33(5)
C2	C1	H1a	108.7(4)
C2	C1	H1b	113.0(4)
C3	C2	C1	102.35(5)
C5	C2	C1	110.85(5)
C5	C2	C3	113.91(5)
C7	C2	C1	111.46(5)
C7	C2	C3	116.73(5)
C7	C2	C5	101.83(5)
H3a	C3	C2	110.73(3)
H3b	C3	C2	110.73(3)
H3b	C3	H3a	108.8
C4	C3	C2	105.07(5)
C4	C3	H3a	110.73(3)
C4	C3	H3b	110.73(3)
C3	C4	N1	104.39(5)
H4a	C4	N1	110.87(3)
H4a	C4	C3	110.87(3)
H4b	C4	N1	110.87(3)
H4b	C4	C3	110.87(3)
H4b	C4	H4a	108.9
N2	C5	O1	125.84(5)
C2	C5	O1	125.16(6)
C2	C5	N2	108.98(5)
C7	C6	N2	109.43(5)
C11	C6	N2	128.87(6)
C11	C6	C7	121.70(5)
C6	C7	C2	108.53(5)
C8	C7	C2	130.86(5)
C8	C7	C6	120.51(5)
H8	C8	C7	121.9(4)
C9	C8	C7	118.48(6)
C9	C8	H8	119.6(4)
C8	C9	O2	114.81(5)
C10	C9	O2	124.36(5)
C10	C9	C8	120.82(5)
H10	C10	C9	120.8(4)
C11	C10	C9	120.39(6)
C11	C10	H10	118.8(4)
C10	C11	C6	118.08(6)
H11	C11	C6	122.6(4)
H11	C11	C10	119.3(4)
H12a	C12	N1	109.5
H12b	C12	N1	109.5
H12b	C12	H12a	109.5
H12c	C12	N1	109.5
H12c	C12	H12a	109.5
H12c	C12	H12b	109.5
H13a	C13	O2	111.2(4)
H13b	C13	O2	105.6(4)
H13b	C13	H13a	111.1(5)
H13c	C13	O2	110.7(4)
H13c	C13	H13a	108.0(6)
H13c	C13	H13b	110.4(6)

Table 20: Torsion Angles in ° for CB-122.

Atom	Atom	Atom	Atom	Angle/°
O1	C5	N2	C6	178.14(6)
O1	C5	C2	C1	65.23(6)
O1	C5	C2	C3	-49.55(6)
O1	C5	C2	C7	-176.10(6)
O2	C9	C8	C7	-178.25(5)
O2	C9	C10	C11	179.04(6)
N1	C1	C2	C3	-35.67(5)
N1	C1	C2	C5	-157.50(4)
N1	C1	C2	C7	89.84(5)
N1	C4	C3	C2	16.27(5)
N2	C5	C2	C1	-113.36(5)
N2	C5	C2	C3	131.86(5)
N2	C5	C2	C7	5.31(5)
N2	C6	C7	C2	3.97(5)
N2	C6	C7	C8	-179.23(5)
N2	C6	C11	C10	-179.64(6)
C1	N1	C4	C3	-39.99(5)
C1	C2	C3	C4	11.75(5)
C1	C2	C7	C6	112.73(5)
C1	C2	C7	C8	-63.62(6)
C2	C1	N1	C4	47.77(5)
C2	C1	N1	C12	171.49(4)
C2	C5	N2	C6	-3.27(5)
C2	C7	C6	C11	-175.10(5)
C2	C7	C8	C9	174.35(6)
C3	C2	C7	C6	-130.18(5)
C3	C2	C7	C8	53.47(6)
C3	C4	N1	C12	-162.87(5)
C4	C3	C2	C5	131.46(5)
C4	C3	C2	C7	-110.23(5)
C5	N2	C6	C7	-0.42(5)
C5	N2	C6	C11	178.58(5)
C5	C2	C7	C6	-5.50(5)
C5	C2	C7	C8	178.15(4)
C6	C7	C8	C9	-1.63(6)
C6	C11	C10	C9	-0.16(7)
C7	C6	C11	C10	-0.76(7)
C7	C8	C9	C10	0.73(7)
C8	C7	C6	C11	1.69(7)
C8	C9	O2	C13	-177.94(5)
C8	C9	C10	C11	0.17(7)
C10	C9	O2	C13	3.12(7)

Table 21: Hydrogen Fractional Atomic Coordinates (×10^{4}) and Equivalent Isotropic Displacement Parameters (Å^{2}×10^{3}) for CB-122. U_{eq} is defined as 1/3 of the trace of the orthogonalised U_{ij}.

Atom	x	y	z	U_{eq}
H2	2170(15)	5889(9)	515(8)	41(2)
H1a	1933(12)	1404(8)	513(6)	28.1(17)
H1b	5186(13)	2412(8)	844(6)	29.6(17)
H3a	1728(3)	3958(4)	3841(4)	33.3(18)

Atom	x	y	z	U_{eq}
H3b	-816(8)	2966.0(9)	2644.0(12)	32.6(18)
H4a	-39(7)	685(4)	2311(2)	35.3(18)
H4b	1859.8(16)	1619.0(7)	3798(4)	28.2(17)
H8	7025(12)	4529(8)	3894(7)	29.8(19)
H10	10505(13)	8529(9)	3160(7)	38(2)
H11	6959(13)	7978(8)	1588(7)	30.6(19)
H12a	2470(6)	-680(3)	1099(2)	37.8(19)
H12b	5797(6)	161.9(7)	1253.4(19)	34.7(19)
H12c	4406.1(16)	-261.3(16)	2540(3)	37.0(19)
H13a	13893(13)	8120(9)	4038(7)	42(2)
H13b	14098(14)	8070(9)	5579(7)	40(2)
H13c	12247(13)	9119(9)	5157(7)	36.8(19)

Table 22: Hydrogen Bond information for CB-122.

D	H	A	d(D-H)/Å	d(H-A)/Å	d(D-A)/Å	D-H-A/deg
N2	H2	O1[1]	0.995(8)	1.839(8)	2.8291(7)	173.4(7)

––––

[1]-x,1-y,-

Citations

CrysAlisPro (ROD), Rigaku Oxford Diffraction, Poland (?).

CrysAlisPro Software System, Rigaku Oxford Diffraction, (2024).

L.J. Bourhis and O.V. Dolomanov and R.J. Gildea and J.A.K. Howard and H. Puschmann, The Anatomy of a Comprehensive Constrained, Restrained, Refinement Program for the Modern Computing Environment - Olex2 Disected, *Acta Cryst. A*, (2015), **A71**, 59-71.

O.V. Dolomanov and L.J. Bourhis and R.J. Gildea and J.A.K. Howard and H. Puschmann, Olex2: A complete structure solution, refinement and analysis program, *J. Appl. Cryst.*, (2009), **42**, 339-341.
